# Global, Regional, and National Burden of Endometriosis, PCOS, and Unexplained Infertility and Their Attribution to Infertility, 1990–2021: Global Burden of Disease Study 2021

**DOI:** 10.1111/jebm.70100

**Published:** 2025-12-23

**Authors:** Ye Xu, Yulong Jia, Qianrui Li, Hao Jiang, Yiquan Xiong, Wengxue Liang, Xuehong Liu, Yunxiang Huang, Kang Zou, Xin Sun, Jing Tan, Yan Ren

**Affiliations:** ^1^ General Practice Medical Center, Clinical Epidemiology and Evidence‐based Medicine Center West China Hospital Sichuan University Chengdu China; ^2^ NHC Key Laboratory of Clinical Epidemiology and Evidence‐Based Medicine West China Hospital Sichuan University Chengdu China; ^3^ Sichuan Center of Technology Innovation for Real World Data West China Hospital, Sichuan University Chengdu China; ^4^ Sichuan Provincial Center for Evidence‐Based Chinese Herbal Medicine Research West China Hospital, Sichuan University Chengdu China; ^5^ Department of Nuclear Medicine West China Hospital of Sichuan University Chengdu China

**Keywords:** Endometriosis, global Burden of Disease Study, infertility, polycystic ovarian syndrome, unexplained infertility, women of childbearing age

## Abstract

**Aim:**

Infertility is a growing global health issue affecting millions worldwide. While endometriosis, polycystic ovarian syndrome (PCOS) and unexplained infertility are recognized as the major contributors, their specific burden and impact on infertility among women of childbearing age (WCBA) remain inadequately quantified. This study aimed to evaluate the global burden of three diseases and infertility attributable to them.

**Methods:**

Using data from Global Burden of Disease Study 2021, we assessed the temporal trends of three diseases by average annual percentage change (AAPC) in age‐standardized prevalence rate (ASPR) and DALYs rate (ASDR), and evaluated their correlation with socio‐demographic index (SDI). We also calculated the age‐standardized prevalence and years lived with disability (YLDs) rates of infertility attributable to three diseases.

**Results:**

In 2021, the global ASPRs of endometriosis, PCOS, and unexplained infertility were 1070.7 (AAPC: –1.02%, declining), 3364.5 (AAPC: 0.74%, increasing), and 5586.2 (AAPC: 0.70%, increasing), and infertility attributable to three diseases were 60.6, 638.2, and 5586.2 per 100,000 population, respectively. Regionally, Oceania had the highest ASPR for endometriosis, East Asia for unexplained infertility, and high‐income Asia Pacific for PCOS. Infertility attributable to PCOS exhibited higher age‐standardized prevalence and YLDs rates compared to endometriosis in most regions. Notably, ASPRs and ASDRs for endometriosis and unexplained infertility decreased with SDI, while PCOS rates increased with SDI.

**Conclusions:**

The increasing global burden of endometriosis, PCOS, and unexplained infertility among WCBA has significantly contributed to rising infertility rates, with distinct regional and demographic disparities. Targeted public health strategies are needed to address these trends.

## 1 Introduction

The global total fertility rate (TFR) has declined by more than half, from 4.84 to 2.23, between 1950 and 2021 [1]. Infertility is a major factor affecting fertility and human health, and has become a worldwide public health issues, affecting 17.5% adults globally (1/6 of global population) [2, 3, 4]. Infertility is particularly devastating for women. The prevalence of infertility affects approximately 8%–12% of couples of reproductive ages. Although female factors account for approximately 50% of infertility cases, they are primarily responsible for 70%–80% of cases [5]. Moreover, it also elevates the risk of endometrial and ovarian cancers, and engenders other social and psychological issues [6, 7, 8]. Addressing infertility is central to achieving Sustainable Development Goal (SDG) 3 and 5 by the World Health Organization (WHO) [9]. And a substantial proportion of infertility cases are preventable through the effective management of underlying reproductive disorders, such as endometriosis and PCOS [10]. Therefore, identifying the burden of infertility‐related diseases and their attribution to infertility burden among WCBA is crucial for reducing the infertility incidence.

The major diseases associated with infertility in female include ovulatory dysfunction (e.g., PCOS), tubal disease, endometriosis, uterine/cervical factors and unexplained factors [11]. Among these, PCOS and endometriosis are the leading causes of infertility [12], and unexplained infertility is also a major cause of infertility among WCBA [13]. Endometriosis, PCOS, and unexplained infertility typically require long‐term management strategies [14, 15] and substantial healthcare expenditures [16, 17]. However, early detection and intervention for three conditions can reduce the infertility incidence. These diseases are strongly influenced by economic development, availability of medical resources, and access to healthcare services [18]. Therefore, it is crucial to systematically assess trend in the burden of these diseases from 1990 to 2021 to inform effective prevention and treatment strategies.

Although the global burden of endometriosis, PCOS, and unexplained infertility has been respectively assessed in several researches [19, 20, 21], these studies primarily focused on the burden of diseases themselves, ignoring the relationship among three diseases and the relationship between three diseases and infertility. Moreover, the burden of infertility attributable to three diseases in different regions is unclear. Therefore, we comprehensively estimated the burden of three diseases and infertility attributable to them in various regions, and compared the disparities in the burden of three diseases among WBCA using the GBD 2021 to provide evidence‐based support for the implementation of the strategies to mitigate infertility‐related diseases and infertility, with the aim of promoting the development of female reproductive health.

## 2 Methods

### 2.1 Study Population and Data Collection

Global Burden of Diseases Study (GBD) 2021 was produced and led by the Institute for the Health Metrics and Evaluation (IHME), and aimed to provide the comprehensive estimates of global disease burden using 100983 data sources, including census, surveys, disease registries, and other sources (https://www.healthdata.org/data‐tools‐practices/data‐sources). GBD 2021 reported the epidemiological indexes including incidence, prevalence, disability‐adjusted life‐years (DALYs), years of life lost (YLLs) and years lived with disability (YLDs) due to 371 diseases and injuries by sex, 25 age groups with 5 years as an interval, 204 countries and territories grouped into 7 super‐regions and 21 regions from 1990 to 2021 (https://vizhub.healthdata.org/gbd‐results/) [22]. We extracted data on prevalence, DALYs of three diseases and prevalence, YLDs of infertility attributable to them from the GBD 2021 among WCBA at five‐year intervals from 1990 to 2021. All estimates for this study are stratified by female, age from 15 to 49 years, 21 regions, and 204 countries and territories, covering the period from 1990 to 2021.

### 2.2 Definition

In GBD 2021, endometriosis is defined as growth of endometrial tissue outside the uterus, diagnosed via pelvic examination with laparoscopy or laparotomy confirmation according to ACOG guidelines. PCOS is characterized by hyperandrogenism, ovulatory dysfunction, and polycystic ovaries according to National Institutes of Health (NIH). Unexplained infertility refers to infertility excluding known causes such as pelvic inflammatory disease, PCOS, and endometriosis among WCBA [22]. The international classification of disease codes was defined in the supplementary appendix (Table S1). In addition, infertility is defined as failure to conceive and have a livebirth via regular unprotected sexual intercourse, encompassing primary infertility and secondary infertility. Infertility is an impairment caused by eight identified causes (e.g., chlamydia, gonorrhea, other STIs, endometriosis, PCOS, maternal sepsis, congenital Turner syndrome, congenital urogenital anomalies) and unknown causes in women according to GBD 2021 [23]. Primary infertility was defined the female has never been pregnant in a couple, while secondary infertility was defined the female who has been pregnant before and successfully delivered is unable to become pregnant again [24]. All rates were expressed per 100,000 persons. Women of childbearing age was defined as 15 to 49 years by WHO, denotes the period of females with the reproductive ability and cyclical sex hormones changes [25]. The socio‐demographic index (SDI) is a composite measure of total fertility rate (females age <25 years), income per capita, and average years of educational attainment (individuals age ≥15 years) [26].

### 2.3 Risk Attribution to Infertility Burden

The prevalence and YLDs of infertility attributable to endometriosis, PCOS, and unexplained infertility among WCBA from 1990 to 2021 were retrieved directly from the GBD 2021. For endometriosis and PCOS, the prevalence of infertility attributable to them was determined by their own prevalence multiply the risk attribution. The attributable fractions of endometriosis and PCOS were derived from the Australian Longitudinal Women's Health Study (ALWHS) (http://www.alswh.org.au/). For unexplained infertility, the attribution to infertility was calculated by subtracting the summed attribution to infertility prevalence of eight known causes (including endometriosis, PCOS, STIs) from overall prevalence of infertility according to GBD 2021 (https://vizhub.healthdata.org/gbd‐results/) [22]. The infertility attributable to three diseases did not lead to mortality, therefore the DALYs were equal to YLDs, so we only estimated the YLDs. The primary infertility and secondary infertility attributable to them were also estimated from the GBD disability weights survey. Additionally, we estimated the percentage contribution of these 3 diseases as risk factors in 2021.

### 2.4 Statistical Analysis

We calculated the age‐standardized rates (ASRs) and their average annual percent changes (AAPCs) to assess the global, regional and national trends of three diseases on prevalence and DALYs in women of childbearing age 15–49 years. We also estimated the ASRs of prevalence and YLDs of infertility attributable to three diseases, respectively. ASRs were standardized by the global age‐standard population, and rates are shown as per 100,000 persons. The 95% uncertainty interval (UI) was used to estimate the heterogeneity of ASR [13]. The AAPC was estimated to assess the temporal trends in age‐standardized prevalence and DALYs rates for three diseases. If both the AAPC and its 95% CI were above or below zero, it indicated the trend of ASR was increase or decline, respectively [26].

We used the smoothing spline models to evaluate the relationships between the burden of endometriosis, PCOS, and unexplained infertility among WCBA and SDI for 21 regions, 204 countries and territories. Additionally, we used Spearman correlation analysis to calculate *r* indices and *p* values for these relationships. Two‐tailed *p* < 0·05 was considered statistically significant. All statistical analyses and mapping were conducted using R software (version 4·4·1).

## 3 Results

### 3.1 The Global Diseases Burden of Endometriosis, PCOS, and Unexplained Infertility

**FIGURE 1:**
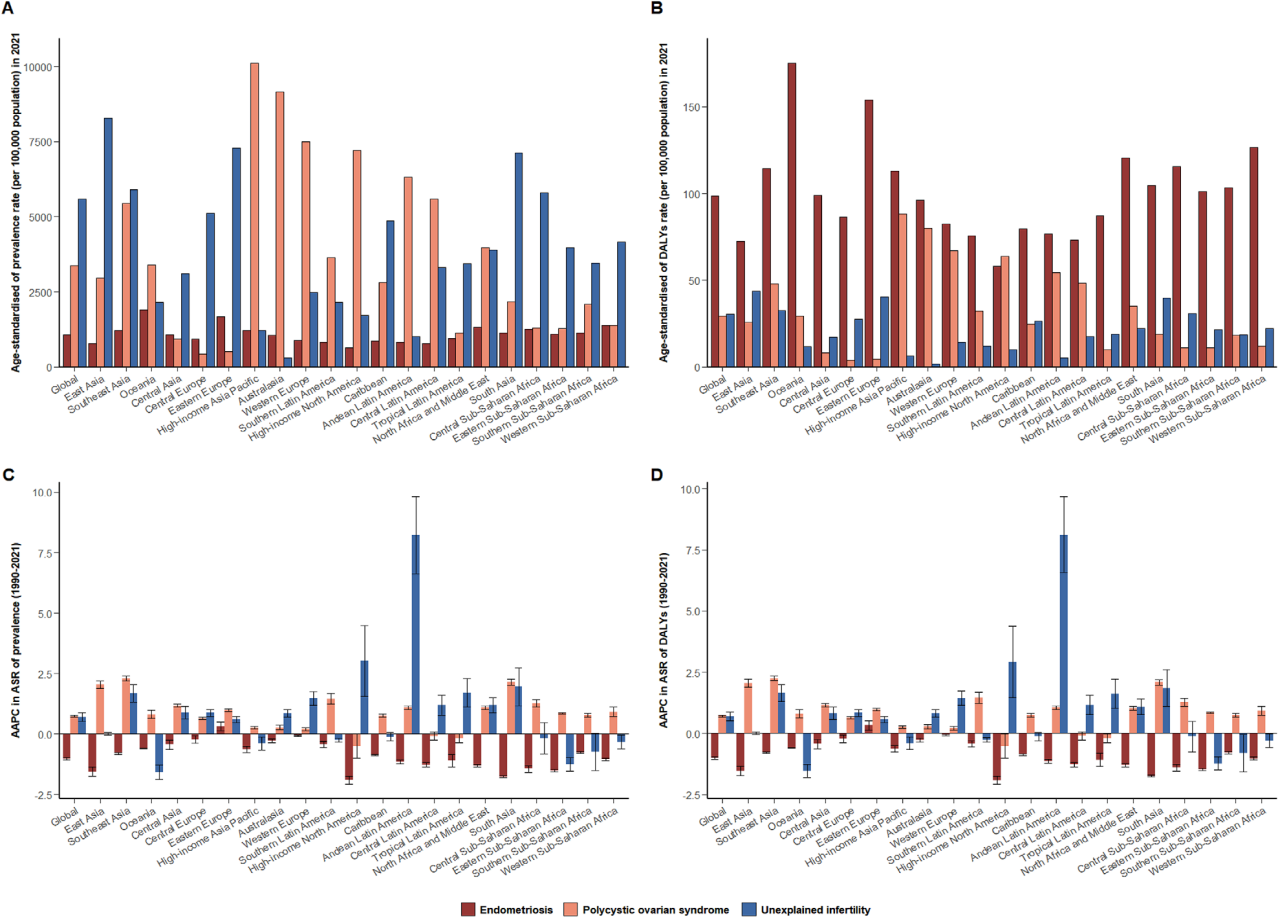
Age‐standardized prevalence and DALYs rates of endometriosis, PCOS, and unexplained infertility for WCBA in 2021, and their AAPC from 1990 to 2021 in global and 21 GBD regions. Age‐standardized rates of prevalence (A) and DALYs (B), and average annual percentage change of age‐standardized rate of prevalence (C) and DALYs (D). PCOS, polycystic ovarian syndrome; AAPC, average annual percentage change; ASR, age‐standardized rate; DALYs, disability‐adjusted life‐years; GBD, Global Burden of Diseases, Injuries, and Risk Factors Study; PCOS, polycystic ovarian syndrome; WCBA, women of childbearing age.

In 2021, the global age‐standardized prevalence rates (ASPRs) of endometriosis, PCOS, and unexplained infertility were 1070.7, 3364.5, and 5586.2 per 100,000 population, respectively (Table 1; Figure 1A). Between 1990 and 2021, the global ASPR of endometriosis significantly decreased by –1.02. In contrast, PCOS and unexplained infertility significantly increased by annual average of 0.74 and 0.70, respectively (Table 1; Figure 1C). The global age‐standardized DALYs rates (ASDRs) of DALYs for endometriosis, PCOS, and unexplained infertility were 98.69, 29.51, and 30.55, respectively (Table 1; Figure 1B). Trends in ASDRs for these diseases followed patterns similar to their respective ASPRs (Table 1; Figure 1D).

**TABLE 1:** Prevalence and DALYs of endometriosis, PCOS, and unexplained infertility for WCBA in 1990 and 2021, and their AAPC from 1990 to 2021 in global and 21 regions.

		Prevalence					DALYs (disability‐adjusted life‐years)				
Location	disease	Number of cases, 1990	Age‐standardized rate per 100,000 population, 1990	Number of cases, 2021	Age‐standardized rate per 100,000 population, 2021	Average annual percentage change, 1990–2021	Number of cases, 1990	Age‐standardized rate per 100,000 population, 1990	Number of cases, 2021	Age‐standardized rate per 100,000 population, 2021	Average annual percentage change, 1990–2021
Global											
	Endometriosis	19,083,472 (13,003,200 to 26,683,297)	1455.45 (1454.80 to 1456.11)	21,045,891 (14,631,681 to 29,054,373)	1070.70 (1070.25 to 1071.16)	−1.02 (−1.07 to −0.97)	1,758,975 (988,452 to 2,760,451)	133.98 (133.78 to 134.18)	1,939,180 (1,123,379 to 2,983,172)	98.69 (98.55 to 98.83)	−1.01 (−1.06 to −0.96)
	Polycystic ovarian syndrome	34,806,508 (24,932,017 to 47,919,343)	2628.48 (2627.60 to 2629.36)	65,767,553 (46,674,563 to 90,615,556)	3364.53 (3363.72 to 3365.35)	0.74 (0.70 to 0.77)	307,944 (136,789 to 642,229)	23.16 (23.08 to 23.25)	576,045 (258,040 to 1,201,745)	29.51 (29.43 to 29.58)	0.72 (0.68 to 0.76)
	Unexplained infertility	59,690,000 (32,625,584 to 104,614,493)	4581.22 (4580.05 to 4582.39)	110,089,459 (58,608,815 to 195,025,585)	5586.19 (5585.15 to 5587.24)	0.70 (0.52 to 0.87)	325,937 (114,823 to 807,747)	24.89 (24.80 to 24.98)	601,134 (213,158 to 1,468,475)	30.55 (30.47 to 30.63)	0.71 (0.53 to 0.89)
GBD regions											
East Asia	Endometriosis	3,825,408 (2,509,090 to 5,447,411)	1198.71 (1197.48 to 1199.93)	2,783,810 (1,995,049 to 3,696,574)	774.06 (773.12 to 775.00)	−1.55 (−1.74 to −1.36)	356,761 (197,508 to 582,328)	111.59 (111.22 to 111.97)	259,839 (152,524 to 415,697)	72.40 (72.11 to 72.69)	−1.53 (−1.71 to −1.35)
	Polycystic ovarian syndrome	5,386,011 (3,772,180 to 7,588,876)	1624.24 (1622.85 to 1625.64)	9,873,930 (6,960,735 to 14,017,692)	2967.58 (2965.65 to 2969.51)	2.04 (1.88 to 2.19)	46,320 (19,894 to 96,016)	13.91 (13.79 to 14.04)	84,711 (37,067 to 175,599)	25.64 (25.46 to 25.82)	2.05 (1.89 to 2.22)
	Unexplained infertility	25,455,243 (13,651,646 to 44,268,551)	7961.88 (7958.76 to 7965.00)	30,097,127 (14,985,652 to 53,555,149)	8289.89 (8286.88 to 8292.89)	0.01 (−0.04 to 0.05)	132,935 (44,100 to 341,116)	41.46 (41.24 to 41.69)	157,326 (51,989 to 406,568)	43.54 (43.33 to 43.76)	0.02 (−0.03 to 0.07)
Southeast Asia	Endometriosis	1,862,261 (1,287,458 to 2,594,969)	1600.25 (1597.91 to 1602.60)	2,282,870 (1,616,603 to 3,144,529)	1226.49 (1224.90 to 1228.08)	−0.80 (−0.83 to −0.76)	173,213 (98,237 to 267,369)	148.61 (147.89 to 149.32)	212,673 (123,354 to 329,093)	114.29 (113.81 to 114.78)	−0.78 (−0.81 to −0.74)
	Polycystic ovarian syndrome	3,506,301 (2,478,196 to 4,942,003)	2928.59 (2925.46 to 2931.72)	9,998,315 (7,055,378 to 14,098,619)	5444.39 (5441.01 to 5447.77)	2.30 (2.19 to 2.40)	31,337 (13,687 to 63,677)	26.01 (25.72 to 26.31)	88,125 (39,050 to 181,685)	48.06 (47.74 to 48.38)	2.26 (2.16 to 2.36)
	Unexplained infertility	5,036,961 (2,626,029 to 8,899,881)	4417.37 (4413.46 to 4421.28)	11,004,744 (5,574,141 to 19,376,109)	5898.53 (5895.05 to 5902.02)	1.68 (1.33 to 2.03)	28,085 (9537 to 69,716)	24.47 (24.18 to 24.76)	60,681 (21,015 to 150,690)	32.56 (32.30 to 32.82)	1.66 (1.32 to 2.00)
Oceania	Endometriosis	33,899 (22,823 to 48,109)	2295.12 (2270.18 to 2320.28)	65,016 (43,562 to 91,311)	1901.34 (1886.69 to 1916.08)	−0.59 (−0.61 to −0.58)	3124 (1756 to 4924)	211.00 (203.49 to 218.73)	5996 (3349 to 9557)	175.16 (170.74 to 179.68)	−0.58 (−0.60 to −0.56)
	Polycystic ovarian syndrome	37,969 (26,136 to 53,568)	2446.04 (2420.89 to 2471.41)	117,784 (81,922 to 168,231)	3384.68 (3365.30 to 3404.14)	0.81 (0.64 to 0.99)	333 (151 to 699)	21.32 (19.04 to 23.84)	1026 (444 to 2149)	29.44 (27.66 to 31.31)	0.81 (0.64 to 0.99)
	Unexplained infertility	48,370 (21,045 to 90,181)	3359.21 (3328.89 to 3389.76)	75,092 (42,007 to 115,416)	2159.76 (2144.29 to 2175.31)	−1.58 (−1.87 to −1.28)	267 (79 to 661)	18.36 (16.19 to 20.77)	416 (144 to 1001)	11.93 (10.81 to 13.14)	−1.53 (−1.81 to −1.25)
Central Asia	Endometriosis	228,992 (155,956 to 324,791)	1350.01 (1344.31 to 1355.73)	265,565 (185,561 to 376,049)	1066.26 (1062.19 to 1070.34)	−0.44 (−0.63 to −0.25)	21,275 (11,941 to 33,150)	125.17 (123.44 to 126.92)	24,611 (14,449 to 38,688)	98.84 (97.60 to 100.09)	−0.43 (−0.62 to −0.24)
	Polycystic ovarian syndrome	112,001 (75,285 to 163,264)	665.96 (661.94 to 670.00)	225,488 (155,209 to 316,467)	923.53 (919.70 to 927.37)	1.18 (1.11 to 1.25)	990 (412 to 2121)	5.86 (5.49 to 6.25)	1973 (832 to 4259)	8.10 (7.75 to 8.47)	1.16 (1.09 to 1.23)
	Unexplained infertility	515,912 (264,788 to 951,106)	2941.99 (2933.82 to 2950.19)	798,112 (353,395 to 1,591,761)	3113.71 (3106.86 to 3120.58)	0.89 (0.62 to 1.15)	2861 (964 to 7099)	16.23 (15.63 to 16.85)	4353 (1393 to 10,832)	17.05 (16.55 to 17.57)	0.83 (0.58 to 1.08)
Central Europe	Endometriosis	313,591 (223,384 to 442,644)	1020.60 (1017.01 to 1024.20)	243,625 (171,211 to 336,803)	931.75 (927.93 to 935.58)	−0.22 (−0.38 to −0.07)	29,126 (16,803 to 45,200)	94.85 (93.76 to 95.95)	22,546 (13,197 to 35,257)	86.45 (85.29 to 87.62)	−0.22 (−0.37 to −0.06)
	Polycystic ovarian syndrome	109,038 (72,301 to 162,996)	353.27 (351.16 to 355.38)	112,350 (76,721 to 158,461)	436.26 (433.61 to 438.92)	0.64 (0.59 to 0.70)	954 (399 to 1985)	3.10 (2.90 to 3.30)	974 (417 to 2048)	3.81 (3.57 to 4.07)	0.64 (0.59 to 0.69)
	Unexplained infertility	1,311,219 (598,614 to 2,598,281)	4151.08 (4143.94 to 4158.23)	1,334,989 (617,168 to 2,521,006)	5108.75 (5099.92 to 5117.59)	0.88 (0.73 to 1.02)	7097 (2214 to 18,258)	22.59 (22.06 to 23.12)	7124 (2268 to 18,324)	27.48 (26.83 to 28.14)	0.84 (0.71 to 0.98)
Eastern Europe	Endometriosis	969,978 (662,911 to 1,358,536)	1699.43 (1696.03 to 1702.84)	828,858 (568,343 to 1,158,821)	1665.13 (1661.37 to 1668.89)	0.33 (0.14 to 0.51)	89,730 (51,052 to 141,354)	157.28 (156.24 to 158.32)	76,348 (44,196 to 121,013)	153.88 (152.74 to 155.03)	0.33 (0.15 to 0.51)
	Polycystic ovarian syndrome	221,989 (150,604 to 321,108)	394.71 (393.05 to 396.37)	251,010 (175,471 to 363,218)	507.61 (505.52 to 509.70)	0.99 (0.94 to 1.04)	1967 (814 to 4144)	3.51 (3.36 to 3.67)	2199 (911 to 4610)	4.51 (4.31 to 4.71)	0.98 (0.93 to 1.03)
	Unexplained infertility	4,008,864 (2,013,408 to 7,351,356)	6713.72 (6707.11 to 6720.34)	3,640,758 (1,828,022 to 6,714,993)	7282.72 (7274.94 to 7290.51)	0.60 (0.48 to 0.73)	22,401 (7776 to 56,945)	37.77 (37.27 to 38.27)	20,006 (6812 to 52,654)	40.63 (40.04 to 41.22)	0.58 (0.45 to 0.71)
High‐income Asia Pacific	Endometriosis	658,015 (436,346 to 918,828)	1428.15 (1424.69 to 1431.63)	492,761 (351,034 to 642,759)	1217.46 (1213.93 to 1221.01)	−0.64 (−0.76 to −0.51)	61,111 (34,371 to 93,649)	132.74 (131.69 to 133.80)	45,670 (28,069 to 69,337)	113.10 (112.02 to 114.18)	−0.63 (−0.76 to −0.51)
	Polycystic ovarian syndrome	4,201,471 (3,030,276 to 5,851,092)	9136.96 (9128.19 to 9145.75)	3,894,790 (2,751,824 to 5,438,023)	10116.87 (10106.40 to 10127.34)	0.27 (0.22 to 0.32)	36,551 (16,146 to 74,038)	79.69 (78.87 to 80.51)	33,723 (15,178 to 68,444)	88.17 (87.19 to 89.15)	0.27 (0.22 to 0.31)
	Unexplained infertility	658,058 (58,320 to 1,978,968)	1320.08 (1316.87 to 1323.29)	555,824 (51,072 to 1,751,423)	1222.61 (1219.36 to 1225.88)	−0.39 (−0.66 to −0.12)	3524 (223 to 13,741)	7.09 (6.85 to 7.33)	2963 (191 to 11,210)	6.54 (6.31 to 6.79)	−0.40 (−0.66 to −0.14)
Australasia	Endometriosis	64,938 (44,155 to 91,896)	1199.83 (1190.60 to 1209.11)	76,354 (53,431 to 106,327)	1045.48 (1037.99 to 1053.02)	−0.27 (−0.36 to −0.19)	5934 (3407 to 9329)	109.67 (106.90 to 112.51)	7007 (4074 to 11,141)	96.04 (93.77 to 98.34)	−0.27 (−0.35 to −0.18)
	Polycystic ovarian syndrome	425,029 (313,039 to 559,106)	7885.92 (7862.19 to 7909.71)	665,142 (473,656 to 925,079)	9156.94 (9134.64 to 9179.29)	0.27 (0.18 to 0.37)	3709 (1688 to 7703)	68.88 (66.68 to 71.14)	5792 (2601 to 12,020)	79.97 (77.89 to 82.09)	0.28 (0.19 to 0.37)
	Unexplained infertility	14,969 (3920 to 63,777)	266.62 (262.36 to 270.94)	23,946 (5824 to 97,727)	308.63 (304.72 to 312.59)	0.86 (0.70 to 1.01)	85 (14 to 364)	1.51 (1.21 to 1.88)	135 (22 to 593)	1.75 (1.47 to 2.09)	0.83 (0.68 to 0.97)
Western Europe	Endometriosis	912,443 (619,279 to 1,284,577)	939.45 (937.52 to 941.38)	838,464 (604,693 to 1,124,385)	896.80 (894.84 to 898.76)	−0.07 (−0.10 to −0.04)	84,115 (47,743 to 130,830)	86.64 (86.05 to 87.23)	77,091 (45,223 to 118,774)	82.58 (81.99 to 83.18)	−0.07 (−0.10 to −0.04)
	Polycystic ovarian syndrome	6,457,417 (4,546,424 to 8,972,736)	6728.43 (6723.22 to 6733.64)	7,005,904 (4,935,409 to 9,775,583)	7493.55 (7487.88 to 7499.22)	0.21 (0.14 to 0.28)	57,717 (26,244 to 120,381)	60.15 (59.66 to 60.65)	62,191 (28,170 to 129,166)	66.97 (66.44 to 67.51)	0.22 (0.15 to 0.28)
	Unexplained infertility	1,725,906 (765,757 to 3,309,948)	1732.28 (1729.69 to 1734.87)	2,492,439 (748,783 to 5,485,462)	2474.19 (2471.09 to 2477.29)	1.48 (1.19 to 1.77)	9963 (3055 to 24,796)	10.01 (9.81 to 10.21)	14,102 (3264 to 39,770)	14.12 (13.89 to 14.36)	1.44 (1.15 to 1.73)
Southern Latin America	Endometriosis	120,883 (81,034 to 167,753)	988.40 (982.83 to 993.99)	148,402 (108,555 to 193,884)	825.80 (821.59 to 830.02)	−0.43 (−0.56 to −0.30)	11,131 (6326 to 17,161)	90.97 (89.28 to 92.67)	13,612 (8228 to 20,913)	75.78 (74.51 to 77.07)	−0.43 (−0.56 to −0.30)
	Polycystic ovarian syndrome	282,120 (195,051 to 409,782)	2281.39 (2272.97 to 2289.83)	637,611 (448,562 to 915,301)	3637.79 (3628.84 to 3646.76)	1.46 (1.24 to 1.69)	2506 (1122 to 5144)	20.23 (19.45 to 21.04)	5637 (2449 to 11,709)	32.22 (31.39 to 33.08)	1.46 (1.24 to 1.68)
	Unexplained infertility	282,045 (78,216 to 599,780)	2324.08 (2315.50 to 2332.67)	387,109 (94,046 to 864,584)	2142.36 (2135.61 to 2149.13)	−0.25 (−0.33 to −0.16)	1582 (328 to 4683)	13.00 (12.37 to 13.66)	2160 (370 to 6141)	11.98 (11.48 to 12.50)	−0.26 (−0.34 to −0.17)
High‐income North America	Endometriosis	761,179 (503,691 to 1,085,576)	971.34 (969.15 to 973.53)	551,615 (411,873 to 720,766)	634.60 (632.92 to 636.28)	−1.91 (−2.08 to −1.74)	70,030 (38,795 to 117,116)	89.37 (88.70 to 90.03)	50,355 (31,101 to 77,465)	57.98 (57.47 to 58.49)	−1.91 (−2.08 to −1.75)
	Polycystic ovarian syndrome	4,287,619 (3,024,720 to 6,002,205)	5695.27 (5689.84 to 5700.71)	6,071,380 (4,532,820 to 7,994,587)	7200.01 (7194.24 to 7205.77)	−0.50 (−1.01 to 0.01)	38,418 (16,995 to 79,943)	51.04 (50.52 to 51.55)	53,692 (24,467 to 108,624)	63.85 (63.31 to 64.40)	−0.51 (−1.02 to −0.01)
	Unexplained infertility	838,950 (80,704 to 2,240,173)	1039.75 (1037.52 to 1041.98)	1,478,922 (218,891 to 3,682,781)	1714.94 (1712.17 to 1717.71)	3.01 (1.55 to 4.49)	4977 (394 to 15,754)	6.18 (6.01 to 6.36)	8507 (1021 to 26,836)	9.90 (9.69 to 10.11)	2.91 (1.45 to 4.39)
Caribbean	Endometriosis	105,757 (70,995 to 149,734)	1154.89 (1147.85 to 1161.97)	105,254 (71,069 to 149,248)	866.00 (860.78 to 871.25)	−0.87 (−0.90 to −0.83)	9736 (5583 to 15,221)	106.16 (104.03 to 108.32)	9660 (5487 to 14,974)	79.50 (77.92 to 81.10)	−0.86 (−0.90 to −0.83)
	Polycystic ovarian syndrome	210,460 (141,599 to 302,722)	2256.95 (2247.18 to 2266.75)	339,902 (230,827 to 490,229)	2823.51 (2814.02 to 2833.03)	0.76 (0.70 to 0.83)	1877 (814 to 3907)	20.01 (19.10 to 20.96)	2982 (1295 to 6293)	24.79 (23.91 to 25.70)	0.75 (0.68 to 0.82)
	Unexplained infertility	428,429 (267,614 to 698,029)	4817.67 (4803.12 to 4832.26)	589,496 (342,122 to 997,994)	4863.01 (4850.60 to 4875.44)	−0.11 (−0.29 to 0.07)	2394 (903 to 5819)	26.71 (25.64 to 27.82)	3230 (1194 to 8225)	26.65 (25.74 to 27.59)	−0.13 (−0.29 to 0.04)
Andean Latin America	Endometriosis	111,333 (75,420 to 156,049)	1218.33 (1211.03 to 1225.67)	147,420 (100,385 to 208,479)	832.57 (828.32 to 836.83)	−1.14 (−1.22 to −1.07)	10,294 (5616 to 15,894)	112.41 (110.21 to 114.66)	13,598 (7791 to 20,791)	76.78 (75.50 to 78.09)	−1.13 (−1.21 to −1.06)
	Polycystic ovarian syndrome	433,323 (298,408 to 603,047)	4572.25 (4558.32 to 4586.21)	1,105,175 (756,783 to 1,547,626)	6305.75 (6293.99 to 6317.53)	1.09 (1.01 to 1.16)	3770 (1683 to 8162)	39.67 (38.38 to 40.99)	9546 (4192 to 19,999)	54.46 (53.37 to 55.56)	1.06 (0.99 to 1.14)
	Unexplained infertility	11,645 (6577 to 25,547)	139.48 (136.93 to 142.07)	180,844 (18,126 to 574,607)	1019.66 (1014.96 to 1024.37)	8.22 (6.63 to 9.82)	64 (20 to 170)	0.76 (0.58 to 0.98)	970 (83 to 3433)	5.47 (5.13 to 5.82)	8.11 (6.56 to 9.68)
Central Latin America	Endometriosis	482,039 (323,876 to 685,319)	1193.02 (1189.57 to 1196.48)	544,022 (368,948 to 768,535)	791.94 (789.83 to 794.05)	−1.27 (−1.36 to −1.17)	44,690 (25,337 to 69,950)	110.36 (109.31 to 111.42)	50,198 (28,688 to 77,952)	73.09 (72.45 to 73.73)	−1.27 (−1.37 to −1.17)
	Polycystic ovarian syndrome	2,128,215 (1,460,720 to 2,956,888)	5067.18 (5060.18 to 5074.18)	3,806,925 (2,645,029 to 5,321,795)	5580.86 (5575.25 to 5586.47)	−0.09 (−0.26 to 0.08)	18,714 (8297 to 39,324)	44.32 (43.67 to 44.98)	33,030 (14,361 to 69,177)	48.44 (47.91 to 48.96)	−0.10 (−0.27 to 0.06)
	Unexplained infertility	619,982 (225,562 to 1,276,674)	1681.74 (1677.50 to 1685.99)	2,270,205 (933,287 to 4,395,051)	3307.78 (3303.48 to 3312.09)	1.19 (0.78 to 1.59)	3368 (841 to 9450)	9.07 (8.76 to 9.39)	12,076 (3458 to 32,018)	17.60 (17.29 to 17.91)	1.17 (0.77 to 1.57)
Tropical Latin America	Endometriosis	454,603 (292,807 to 667,858)	1162.48 (1159.06 to 1165.91)	586,267 (403,001 to 822,883)	947.69 (945.25 to 950.13)	−1.10 (−1.36 to −0.84)	41,762 (23,468 to 67,636)	106.63 (105.59 to 107.67)	53,954 (31,178 to 82,793)	87.27 (86.53 to 88.01)	−1.07 (−1.33 to −0.81)
	Polycystic ovarian syndrome	418,592 (283,411 to 604,423)	1049.41 (1046.19 to 1052.64)	694,299 (471,531 to 984,784)	1140.94 (1138.24 to 1143.65)	−0.17 (−0.35 to 0.00)	3752 (1603 to 7919)	9.37 (9.07 to 9.68)	6114 (2607 to 12,902)	10.08 (9.83 to 10.34)	−0.19 (−0.37 to −0.01)
	Unexplained infertility	1,042,453 (543,526 to 1,869,616)	2710.47 (2705.23 to 2715.72)	2,215,723 (943,705 to 4,309,763)	3428.96 (3424.43 to 3433.49)	1.71 (1.11 to 2.31)	5989 (2095 to 15,567)	15.48 (15.08 to 15.88)	12,166 (3807 to 32,977)	18.90 (18.57 to 19.24)	1.62 (1.02 to 2.23)
North Africa and Middle East	Endometriosis	1,458,794 (986,186 to 2,053,663)	1939.75 (1936.53 to 1942.98)	2,110,649 (1,459,472 to 2,972,535)	1318.56 (1316.78 to 1320.34)	−1.30 (−1.37 to −1.24)	133,707 (77,683 to 208,514)	177.28 (176.31 to 178.26)	192,815 (111,805 to 296,595)	120.48 (119.94 to 121.02)	−1.30 (−1.36 to −1.24)
	Polycystic ovarian syndrome	2,314,896 (1,605,499 to 3,288,154)	2963.30 (2959.38 to 2967.23)	6,335,263 (4,447,536 to 8,950,145)	3968.60 (3965.51 to 3971.70)	1.09 (1.02 to 1.16)	21,107 (9225 to 44,527)	26.79 (26.42 to 27.16)	56,219 (25,110 to 119,097)	35.25 (34.96 to 35.54)	1.04 (0.96 to 1.11)
	Unexplained infertility	1,927,243 (1,224,847 to 2,787,806)	2600.04 (2596.30 to 2603.79)	6,357,159 (3,075,148 to 10,825,896)	3897.40 (3894.37 to 3900.44)	1.19 (0.88 to 1.50)	11,455 (4240 to 25,501)	15.23 (14.95 to 15.52)	35,930 (11,008 to 89,848)	22.08 (21.85 to 22.31)	1.09 (0.77 to 1.41)
South Asia	Endometriosis	4,768,052 (3,225,340 to 6,679,453)	1938.23 (1937.35 to 1939.11)	5,637,977 (3,885,331 to 7,896,451)	1141.27 (1140.80 to 1141.74)	−1.76 (−1.80 to −1.73)	435,105 (248,488 to 672,878)	176.52 (176.26 to 176.79)	517,015 (297,313 to 803,042)	104.58 (104.44 to 104.72)	−1.74 (−1.77 to −1.70)
	Polycystic ovarian syndrome	3,111,983 (2,216,403 to 4,330,484)	1229.14 (1228.44 to 1229.83)	10,749,370 (7,599,230 to 15,053,407)	2174.75 (2174.10 to 2175.40)	2.14 (2.01 to 2.27)	27,780 (12,092 to 58,787)	10.89 (10.82 to 10.95)	94,328 (40,875 to 197,626)	19.06 (19.00 to 19.12)	2.08 (1.97 to 2.20)
	Unexplained infertility	11,114,297 (5,715,754 to 19,378,720)	4481.16 (4479.83 to 4482.49)	35,555,258 (19,863,521 to 60,335,282)	7119.35 (7118.18 to 7120.52)	1.95 (1.17 to 2.75)	63,660 (19,959 to 148,994)	25.37 (25.27 to 25.47)	199,476 (71,920 to 470,290)	39.88 (39.79 to 39.97)	1.85 (1.10 to 2.60)
Central Sub‐Saharan Africa	Endometriosis	239,278 (163,650 to 339,235)	2028.75 (2020.39 to 2037.14)	393,437 (270,313 to 550,812)	1264.37 (1260.35 to 1268.40)	−1.44 (−1.58 to −1.29)	21,681 (12,492 to 34,160)	183.36 (180.85 to 185.89)	35,955 (20,385 to 56,652)	115.30 (114.08 to 116.52)	−1.40 (−1.54 to −1.26)
	Polycystic ovarian syndrome	104,326 (72,179 to 151,825)	854.52 (849.17 to 859.90)	418,511 (289,389 to 604,024)	1291.50 (1287.50 to 1295.51)	1.28 (1.13 to 1.43)	905 (384 to 1873)	7.35 (6.86 to 7.86)	3641 (1571 to 7479)	11.15 (10.78 to 11.53)	1.27 (1.11 to 1.44)
	Unexplained infertility	591,991 (313,522 to 1,069,334)	5403.15 (5389.09 to 5417.24)	1,705,616 (872,692 to 3,067,605)	5790.10 (5781.31 to 5798.90)	−0.17 (−0.80 to 0.47)	3163 (1051 to 7692)	28.56 (27.55 to 29.60)	9141 (2975 to 21,605)	30.77 (30.14 to 31.42)	−0.13 (−0.75 to 0.50)
Eastern Sub‐Saharan Africa	Endometriosis	718,201 (486,429 to 1,025,216)	1762.15 (1757.93 to 1766.39)	1,118,762 (753,328 to 1,589,080)	1101.33 (1099.24 to 1103.42)	−1.51 (−1.56 to −1.45)	65,626 (37,085 to 102,303)	160.60 (159.33 to 161.88)	102,871 (58,437 to 158,257)	101.02 (100.38 to 101.65)	−1.47 (−1.53 to −1.41)
	Polycystic ovarian syndrome	422,757 (295,395 to 609,315)	990.93 (987.81 to 994.05)	1,362,795 (957,526 to 1,946,441)	1282.62 (1280.40 to 1284.84)	0.86 (0.83 to 0.89)	3686 (1543 to 7733)	8.56 (8.27 to 8.85)	11,842 (5067 to 25,040)	11.07 (10.86 to 11.27)	0.85 (0.82 to 0.88)
	Unexplained infertility	1,956,158 (1,312,452 to 2,936,820)	5024.78 (5017.55 to 5032.02)	3,960,451 (2,299,441 to 6,526,195)	3974.09 (3970.12 to 3978.07)	−1.25 (−1.53 to −0.97)	10,614 (4134 to 23,829)	26.92 (26.39 to 27.45)	21,486 (7752 to 50,131)	21.36 (21.07 to 21.66)	−1.23 (−1.50 to −0.96)
Southern Sub‐Saharan Africa	Endometriosis	187,249 (127,732 to 262,696)	1462.41 (1455.63 to 1469.23)	249,659 (169,419 to 350,291)	1139.63 (1135.15 to 1144.12)	−0.75 (−0.79 to −0.72)	17,162 (9903 to 26,407)	133.68 (131.64 to 135.75)	22,666 (12,903 to 35,485)	103.41 (102.06 to 104.77)	−0.78 (−0.82 to −0.73)
	Polycystic ovarian syndrome	220,249 (151,839 to 316,995)	1667.09 (1659.93 to 1674.28)	456,796 (312,539 to 644,677)	2094.17 (2088.09 to 2100.27)	0.79 (0.70 to 0.87)	1940 (834 to 4237)	14.56 (13.90 to 15.25)	3957 (1695 to 8338)	18.14 (17.58 to 18.71)	0.75 (0.67 to 0.83)
	Unexplained infertility	691,791 (339,581 to 1,243,466)	5521.39 (5508.19 to 5534.62)	782,508 (260,859 to 1,743,635)	3466.06 (3458.38 to 3473.76)	−0.75 (−1.52 to 0.03)	3810 (1345 to 9669)	30.11 (29.15 to 31.10)	4197 (1096 to 11,247)	18.60 (18.04 to 19.18)	−0.80 (−1.57 to −0.03)
Western Sub‐Saharan Africa	Endometriosis	806,577 (548,667 to 1,135,225)	1945.51 (1941.11 to 1949.91)	1,575,104 (1,063,136 to 2,199,115)	1380.54 (1378.34 to 1382.75)	−1.05 (−1.11 to −0.99)	73,662 (42,078 to 114,956)	177.15 (175.82 to 178.48)	144,697 (82,221 to 223,588)	126.49 (125.83 to 127.16)	−1.02 (−1.08 to −0.96)
	Polycystic ovarian syndrome	414,740 (289,668 to 598,696)	962.89 (959.84 to 965.95)	1,644,814 (1,150,807 to 2,346,956)	1385.41 (1383.23 to 1387.59)	0.92 (0.74 to 1.11)	3612 (1530 to 7578)	8.30 (8.02 to 8.59)	14,344 (6144 to 30,582)	11.98 (11.78 to 12.18)	0.92 (0.74 to 1.11)
	Unexplained infertility	1,409,512 (720,926 to 2,539,665)	3486.84 (3480.90 to 3492.80)	4,583,137 (2,061,353 to 8,923,482)	4156.79 (4152.92 to 4160.66)	−0.32 (−0.61 to −0.03)	7643 (2645 to 18,891)	18.61 (18.19 to 19.05)	24,687 (7276 to 61,455)	22.16 (21.88 to 22.45)	−0.30 (−0.58 to −0.02)

DALYs: disability‐adjusted life‐years; PCOS: polycystic ovarian syndrome; WCBA: women of childbearing age; AAPC: average annual percentage change.

### 3.2 Regional and National Disparities in the Burden of Endometriosis, PCOS, and Unexplained Infertility

In 2021, the highest ASPRs of endometriosis were observed in Oceania, Eastern Europe, and Western Sub‐Saharan Africa, while the lowest rate was recorded in high‐income North America (Table 1; Figure 1A). Eastern Europe was only region to experience an increase in the ASPR, with an AAPC of 0.33 from 1990 to 2021 (Table 1; Figure 1C). Nationally, Niger had the highest ASPR of endometriosis. Notably, nine countries reported an increase in ASPRs, with Iceland showing the largest rise (AAPC: 1.24) (Table S2 online).

For PCOS, the highest ASPRs were observed in high‐income Asia Pacific, Australasia, and Western Europe, while the lowest rates were recorded in Central Europe and Eastern Europe (Table 1; Figure 1A). From 1990 to 2021, Tropical Latin America was only region to experience a decrease in the ASPR, with an AAPC of –0.17 (Table 1; Figure 1C). Nationally, the highest ASPRs were observed in Italy, and United States of America with an AAPC of –0.58 showed the largest decrease (Table S3 online).

Regarding unexplained infertility, the highest ASPRs in 2021 were recorded in East Asia and Eastern Europe, while the lowest rates were found in Australasia and Andean Latin America (Table 1; Figure 1A). From 1990 to 2021, Andean Latin America had the largest increase, with an AAPC of 8.22% (Table 1, Figure 1C). At the national level, the highest ASPR was observed in Central African Republic (Table S4 online). Notably, the trends of ASDRs for three diseases also showed consistent features with each own ASPRs (Tables 1 and S2–S4 online).

### 3.3 Age‐Group Disparities in the Burden of Endometriosis, PCOS, and Unexplained Infertility

**FIGURE 2:**
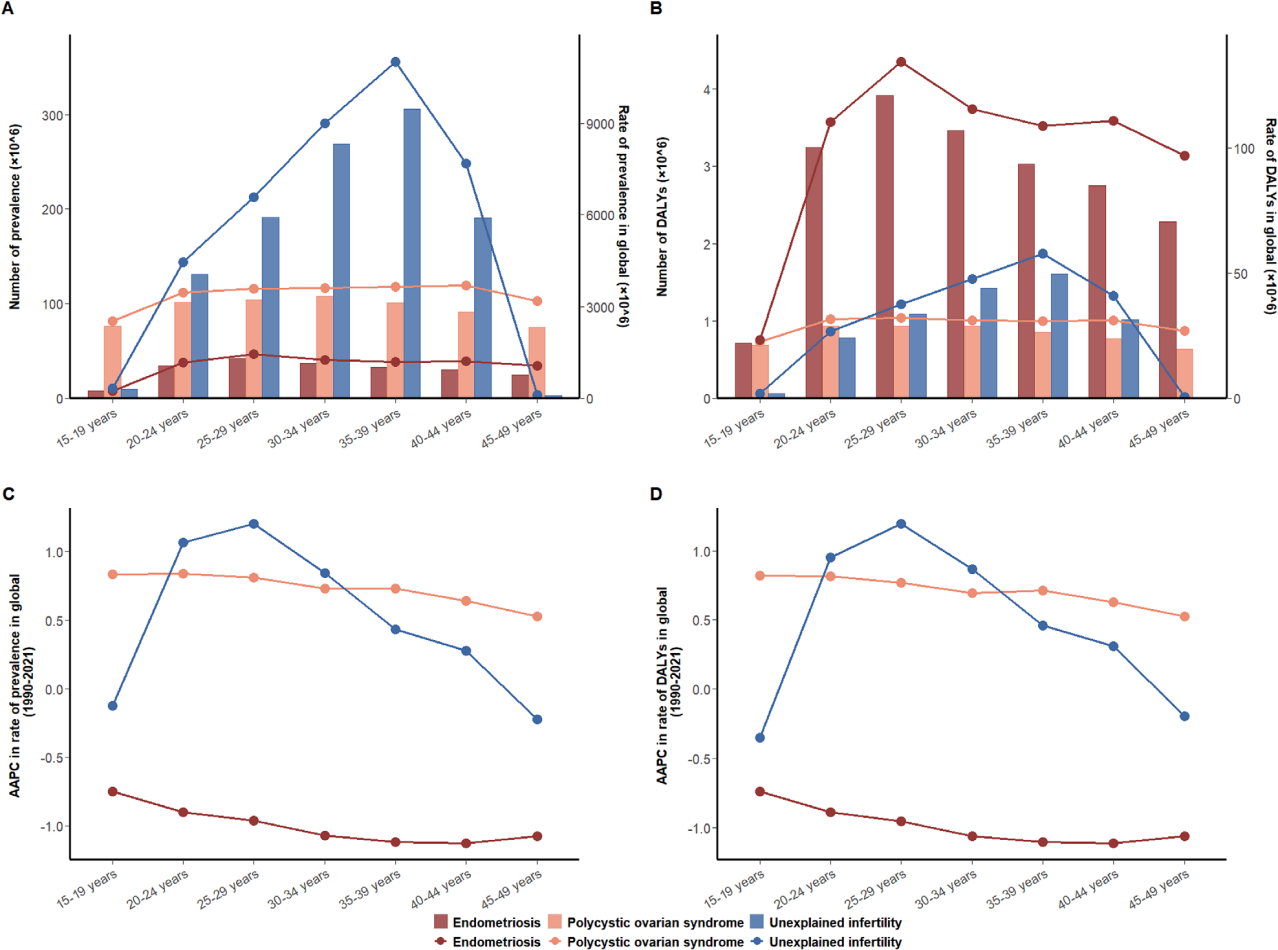
The global prevalence and DALYs of endometriosis, PCOS, and unexplained infertility for WCBA at 7 age groups in 2021, and their AAPC from 1990 to 2021. Numbers and rates of prevalence (A) and DALYs (B) and AAPC of prevalence rate (C) and DALYs rate (D) of endometriosis, PCOS, and unexplained infertility. AAPC, average annual percentage change; ASR, age‐standardized rate; GBD, Global Burden of Diseases, Injuries, and Risk Factors Study; PCOS, polycystic ovarian syndrome; WCBA, women of childbearing age.

In 2021, the global prevalence and DALYs rates for endometriosis showed a significant increase among individuals aged 15–29 years. The DALYs rate for endometriosis was higher than those for PCOS and unexplained infertility. For PCOS, the global burden rates remained relatively stable across the 15–49 years. In contrast, the global burden for unexplained infertility exhibited a marked increase with age, peaking at 35–39 years and subsequently declining in the 40–49 age group (Figure 2A, B). From 1990 to 2021, the AAPC in burden for endometriosis demonstrated a declining trend across all age groups contrasting with PCOS. For unexplained infertility, the trends in prevalence and DALYs showed an increase among individuals aged 20–44 years, with the largest rise occurring in the 20–29 age group. (Figure 2C, D).

### 3.4 SDI‐Group Disparities in the Burden of Endometriosis, PCOS, and Unexplained Infertility

**FIGURE 3:**
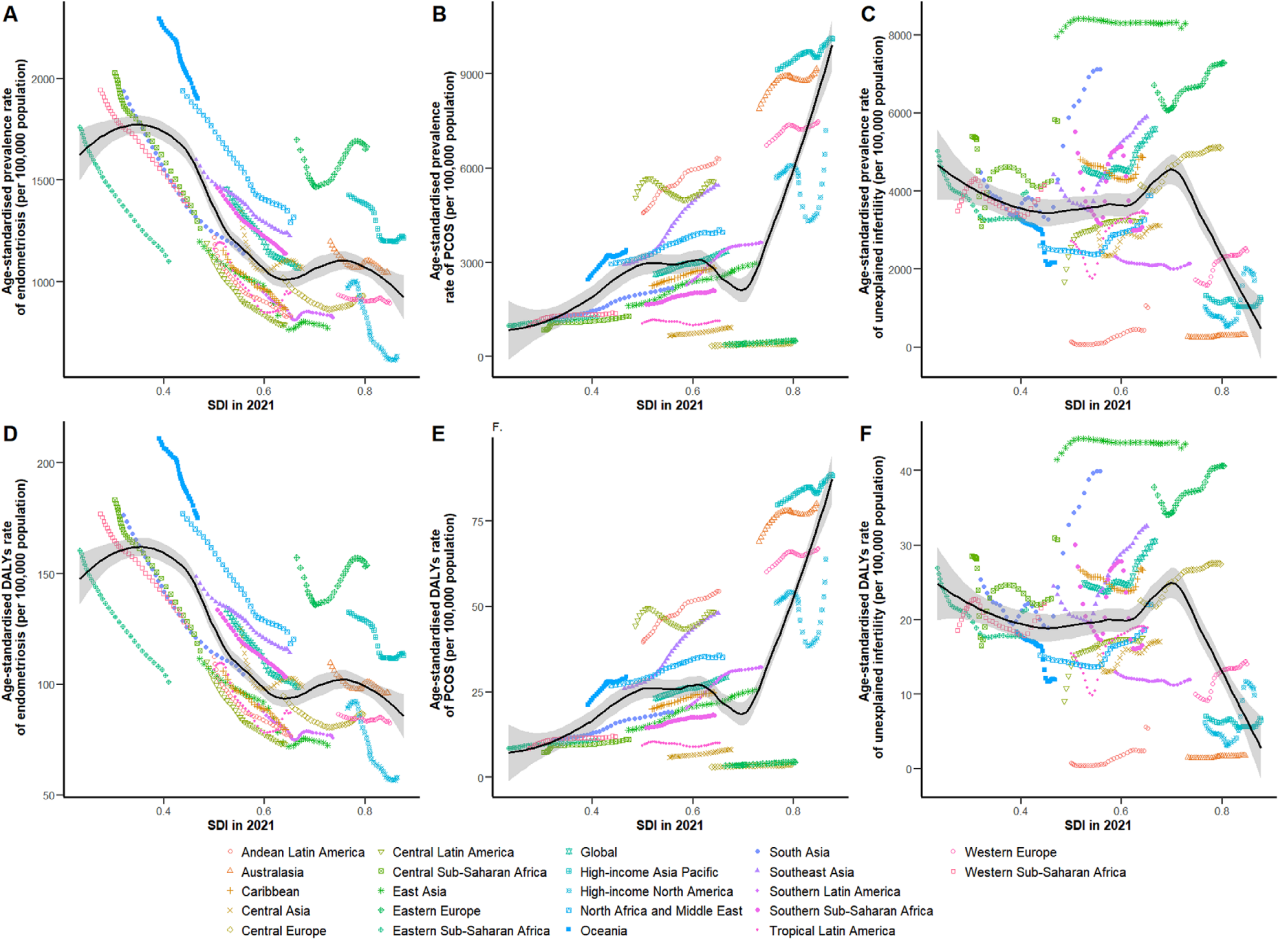
Age‐standardized prevalence and DALYs rates of endometriosis, PCOS, and unexplained infertility, globally and for 21 GBD regions, by SDI, 1990–2021. Age‐standardized prevalence rates of endometriosis (A), PCOS (B), unexplained infertility (C) by SDI. Age‐standardized DALYs rates of endometriosis (D), PCOS (E), unexplained infertility (F) by SDI. Expected values with 95% CI based on SDI and disease rates in 21 regions are shown as a solid line and shaded area. 32 points are plotted for each region and show the observed age‐standardized rate for each year from 1990 to 2021. Points above the solid line represent a higher‐than‐expected burden, while those below the line show a lower‐than‐expected burden. PCOS, polycystic ovarian syndrome; DALYs, disability‐adjusted life‐years; GBD, Global Burden of Diseases, Injuries, and Risk Factors Study; SDI, socio‐demographic index.

The ASPR of endometriosis was negatively correlated with the SDI at both regional (*r* = –0.61) and national levels (Figures 3A and S1A online). Niger had the highest higher‐than‐expected rates of endometriosis in 2021 (Figure S1A online). However, the ASPR of PCOS significantly increased with SDI (*r* = 0.41) among 21 regions (Figure 3B). Nationally, Italy, Japan and New Zealand had significantly higher‐than‐expected rates of PCOS in 2021 (Figure S2A online). The ASPR of unexplained infertility was negatively correlated (*r* = –0.29) (Figure 3C). The Central African Republic displayed significantly higher‐than‐expected rates in 2021 (Figure S3A online). Moreover, the relationship between ASDR of three diseases and SDI was consistent with their respective observed correlation (Figures 3 and S1–S3 online).

### 3.5 The Diseases Burden of Infertility Attributable to Endometriosis, PCOS, and Unexplained Infertility

**FIGURE 4:**
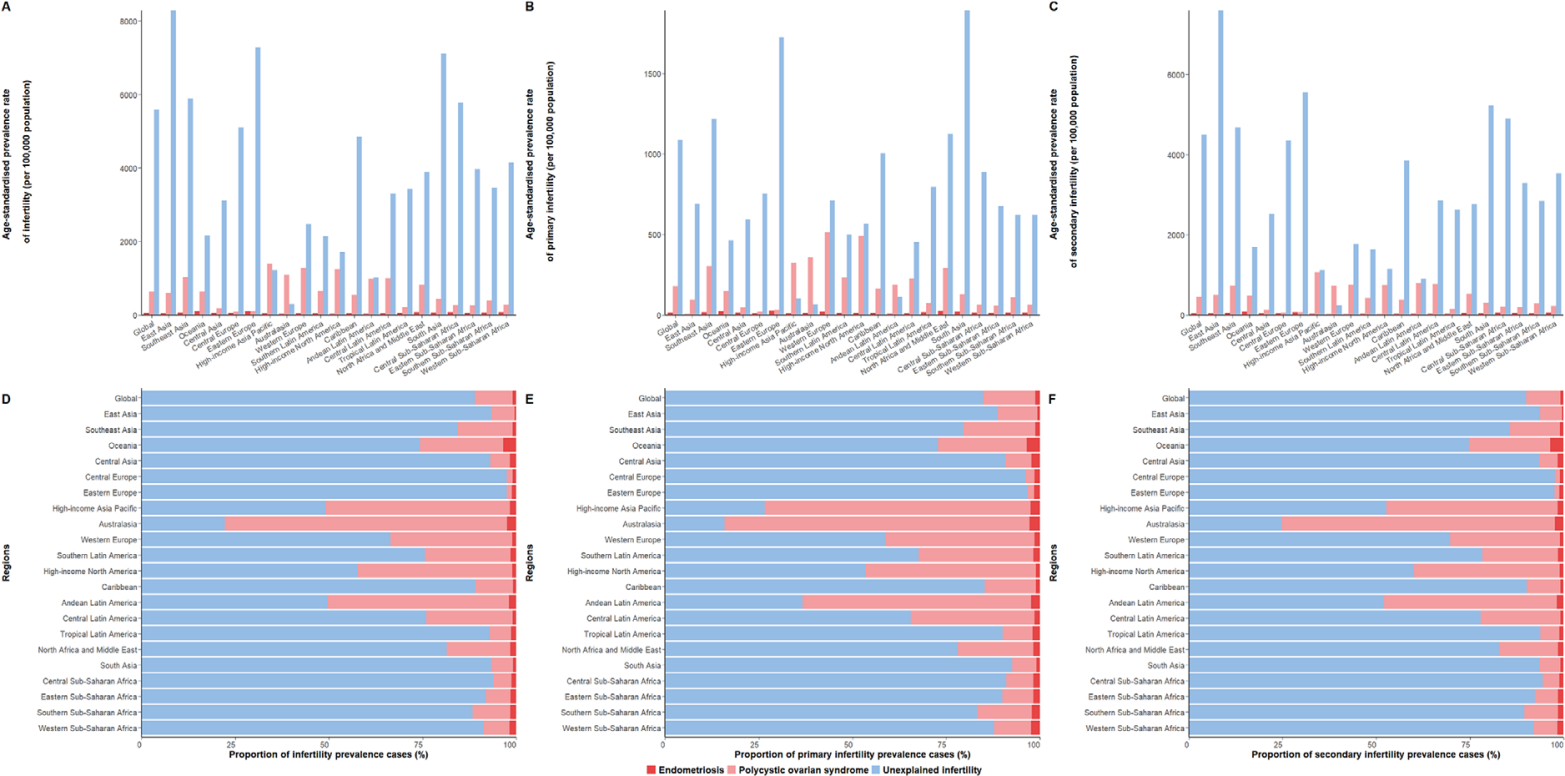
Age‐standardized prevalence rates and proportions of prevalence cases for infertility, primary infertility and secondary infertility attributable to endometriosis, PCOS, and unexplained infertility for WCBA globally and regionally in 2021. Age‐standardized prevalence rate of infertility (A), primary infertility (B), and secondary infertility (C). Proportions of prevalence cases of infertility (D), primary infertility (E), and secondary infertility (F). PCOS, polycystic ovarian syndrome; WCBA, women of childbearing age; YLDs, years lived with disability.

In 2021, the global ASPRs of infertility attributable to endometriosis, PCOS, and unexplained infertility were 60.6, 638.2, and 5586.2, respectively (Table S5 online; Figure 4A). Meanwhile, the corresponding global age‐standardized YLDs rates for infertility due to three diseases were 0.4, 3.7, and 31.2, respectively(Table S5 online; Figure S4A). Furthermore, the global burden of primary and secondary infertility attributable to three diseases exhibited patterns consistent with overall infertility trends (Tables S5–S7 online; Figures 4 and S4 online).

Regionally, infertility attributable to endometriosis showed the lowest burden (prevalence and YLDs) among three conditions. Oceania had the highest regional burden for both infertility and secondary infertility, while Eastern Europe showed the highest burden for primary infertility attributable to endometriosis. Concurrently, high‐income Asia Pacific carried the highest burden for both infertility and primary infertility attributable to PCOS, with Australasia accounting for 75.37% of PCOS‐attributable cases. Additionally, infertility attributable to unexplained infertility had the highest burden in East Asia and South Asia. Furthermore, the proportion of the burden of secondary infertility attributable to endometriosis, PCOS, and unexplained infertility was higher than that for primary infertility globally and in most regions in 2021 (Tables S5–S7 online; Figures 4 and S5 online).

## 4 Discussion

### 4.1 Main Findings

This study provides a comprehensive analysis of the geographical and temporal trends in the burden of endometriosis, PCOS, unexplained infertility, and their contribution to infertility among WCBA. The main findings are summarized as follows: (1) There are significant disparities in burden of these diseases, as well as regional disparities. From 1990 to 2021, the burden of unexplained infertility and PCOS increased. Although the burden of endometriosis decreased during this period, there were significant regional disparities. (2) The global burden of endometriosis and unexplained infertility characterized by an initial increase followed by a decline with age. In contrast, the global age‐specific prevalence and DALYs rates of PCOS maintained relatively stable across various age groups. (3) The ASPR and ASDR for endometriosis and unexplained infertility decreased with SDI, whereas the burden of PCOS increased with SDI. (4) Infertility attributable to PCOS exhibited higher age‐standardized prevalence and YLDs rates compared to endometriosis in most regions.

### 4.2 Comparison With Previous Studies and Potential Explanations

Our study identified a significant decline in global ASPR and ASDR of endometriosis from 1990 to 2021. Although the trend aligns with a systematic review revealing a decrease in prevalence over the past 30 years using health/insurance data [27], it should be interpreted with caution. Previous evidence has demonstrated that the true prevalence of endometriosis is likely underestimated due to diagnostic delays and barriers [28, 29]. The observed decline may partly be influenced by persistent barriers to healthcare access and diagnostic inertia, particularly in low‐resource settings. This hypothesis is supported by a report from a large US health system's electronic medical records database, which documented a decline in endometriosis incidence from 30.2 to 17.4 per 100,000 between 2006 and 2015, while concurrent diagnoses of chronic pelvic pain increased from 3.0% to 5.6% [30]. This highlights the need for considering this potentially affected population when estimating the burden and implementing health strategies. Regionally, high‐SDI regions (e.g., high‐income North America) recorded lower prevalence than low‐ and middle‐SDI region (e.g., Oceania and Sub‐Saharan Africa), aligning with prior findings [31]. This disparity likely stems from a complex combination of factors, including disparities in universal health coverage, cultural and socioeconomic differences, as well as environmental issues [32]. Additionally, our study demonstrated that the global prevalence of endometriosis was highest at 25–29 years among WCBA, contrasting with previous reports of 35–49 years [33, 34]. This shift could result from increased awareness of endometriosis management in adolescents [35, 36].

For PCOS, our findings revealed a global increase in ASPR and ASDR from 1990 to 2021, with high‐income Asia Pacific showing the highest burden in 2021. This aligns with previous studies [37, 38], highlighting the need for improved prevention and management among WCBA. This increase may be partly attributable to the rising prevalence of obesity [39], as evidence shows a positive correlation between PCOS and obesity [40]. Regionally, Tropical Latin America exhibited a declining trend in prevalence and DALYs, contrasting with another study [20]. This discrepancy could result from Brazil's expanded Family Health Strategy (FHS) since the 1990s, which reduced inequalities in primary healthcare services [41, 42, 43]. High‐income regions exhibited the higher ASPR of PCOS, consistent with previous study [44], reflecting disparities across regions, particularly higher prevalence in Europe and North America compared to Asia. These disparities may reflect regional differences in diagnostic intensity, healthcare‐seeking behavior, and environmental or lifestyle [45, 46].

Regarding unexplained infertility, global ASPR and ASDR increased from 1990 to 2021, consistent with previous reports [47, 48]. This trend may stem from environmental and lifestyle changes [49] and delayed childbearing age [50]. Female age is a critical factor, with fertility declining due to irreversible changes in ovarian reserve [51]. We found that Sub‐Saharan Africa showed a significant decline in the prevalence of unexplained infertility and higher prevalence compared other regions in 2021. Similarly, a previous study regarding the global perspective on infertility in the 21st century summarized that, despite infertility rates appeared to decline, Sub‐Saharan Africa still remained high rates of infertility and fertility [10]. The high prevalence may be explained by the unique social and political context as well as limited resource [52], while the decline may relate to reduced unsafe abortions and limited contraception access in Sub‐Saharan Africa [53, 54]. Australasia and high‐income North America recorded lower ASPR of unexplained infertility compared to low‐income regions. This may be attributed to investments in infertility services and affordable assisted reproductive technologies (e.g., IVF) [55, 56], which highly relying on the socioeconomic development.

For infertility attributable to endometriosis, PCOS, and unexplained infertility, our study demonstrated that unexplained infertility contributed the highest burden of infertility among most regions, partly due to unknown causes such as genetics, behavior, and socioeconomic factors excluding nine identified factors in GBD 2021 [22, 57]. Endometriosis contributed the lowest burden of infertility likely due to the effective treatments like surgery and assisted reproductive technologies (ART) in treating endometriosis‐associated infertility [58, 59]. High‐income Asia Pacific and Australasia exhibited higher burdens of infertility attributable to PCOS than unexplained infertility, linked to high PCOS incidence in these regions [27], indicating an urgent need for prevention and improvement in the treatment of PCOS and PCOS‐related infertility. In addition, our analysis revealed that three diseases contributed more to secondary infertility than primary infertility. Similarly, previous studies have reported that these diseases mainly lead to the structural or anatomic alterations, ovulatory dysfunction, and disruption of the fertilization microenvironment in WCBA [60, 61, 62]. These results highlight the dual burden of gynecological disorders, requiring targeted interventions for disease management and fertility preservation.

### 4.3 Clinical Implications

Our study revealed that persistent disparities in healthcare infrastructure and socioeconomic development worldwide are critical drivers of burden of these diseases. Comprehensive strategies integrating multi‐sectoral approaches are urgently needed. In low‐ and middle‐income countries (LMICs), investment should prioritize strengthening primary‐care diagnostic capacity [63, 64] (e.g., ultrasound and basic hormone testing), expanding reproductive health service [65], and improving insurance coverage to enhance early detection and treatment [66]. In high‐income countries (HICs), public health strategies that integrate lifestyle interventions [67] and standardize PCOS management [68]. Additionally, affordability and accessibility of ART should be improved through subsidies, technology transfer, and public–private collaboration [69]. National health systems should expand coverage for early detection of reproductive diseases and infertility prevention, with particular attention to underserved populations.

Meanwhile, Clinical practice must adapt to address persistent diagnostic and management gaps. In low‐resource environments, simplified diagnostic criteria and awareness of nonspecific symptoms (e.g., chronic pelvic pain) can reduce underdiagnosis of conditions like endometriosis [70], especially among younger women [71]. PCOS management should extend beyond fertility concerns to include long‐term monitoring and mitigation of metabolic and cardiovascular risks, supported by comprehensive lifestyle interventions encompassing diet, exercise, and weight modifications [72]. Finally, implementing evidence‐based, standardized care pathways will help ensure consistent, high‐quality treatment across diverse healthcare environments.

### 4.4 Limitations

However, our study has several limitations. First, the accuracy of burden estimates depends on the quality and completeness of primary data, with sparse data in LMICs likely leading to underestimation. Second, heterogeneity in diagnostic criteria, particularly the evolving definitions of PCOS, limits comparability across regions. Third, cultural factors can influence healthcare‐seeking behavior, thereby affecting diagnosis rates. Fourth, significant difference in healthcare systems and population characteristics across countries, require cautious interpretation of the results differences. Fifth, our analysis focused solely on prevalence and DALYs of these diseases, as GBD 2021 only provided these critical indicators for unexplained infertility. Sixth, the lack of specific phenotype data limited our ability to explore the burden of clinical subtypes. Finally, detection bias may overestimate the burdens in high‐income regions with advanced healthcare systems and higher healthcare seeking behaviors, so lower‐reported regions should not be overlooked in resource allocation.

## 5 Conclusion

In summary, the global burden of endometriosis, unexplained infertility and PCOS have resulted in significant public health challenges and influence on infertility. The burden of infertility attributable to these conditions exhibited notable regional differences between high‐ and low‐income settings. Addressing these disparities requires policies tailored to demographic, regional, and disease‐specific factors. In LMICs, strategies should prioritize strengthening diagnostic capacity for these diseases, expanding access to ART, and integrating screening into routine gynecological care. In HICs, efforts should emphasize weight management strategies to prevent PCOS. The implementation of these targeted measures can help alleviate the global burden of these conditions and improve reproductive health outcomes.

## Conflicts of Interest

The authors declare no conflicts of interest.

## Supporting information




**Figure S1**: Age‐standardized prevalence and DALYs rates of endometriosis for 204 countries and territories in 2021, by SDI. Age‐standardized rates of prevalence (A) and DALYs (B) by SDI. Expected values are shown as a solid line. 204 points are plotted for each country and territory. Points above the solid line represent a higher‐than‐expected burden, while those below the line show a lower‐than‐expected burden. SDI: socio‐demographic index.
**Figure S2**: Age‐standardized prevalence and DALYs rates of PCOS for 204 countries and territories in 2021, by SDI. Age‐standardized rates of prevalence (A) and DALYs (B) by SDI. Expected values are shown as a solid line. 204 points are plotted for each country and territory. Points above the solid line represent a higher‐than‐expected burden, while those below the line show a lower‐than‐expected burden. PCOS: polycystic ovarian syndrome; SDI: socio‐demographic index.
**Figure S3**: Age‐standardized prevalence and DALYs rates of unexplained infertility for 204 countries and territories in 2021, by SDI. Age‐standardized rates of prevalence (A) and DALYs (B) by SDI. Expected values are shown as a solid line. 204 points are plotted for each country and territory. Points above the solid line represent a higher‐than‐expected burden, while those below the line show a lower‐than‐expected burden. SDI, socio‐demographic index.
**Figure S4**: Age‐standardized YLDs rates and proportions of YLDs cases for infertility, primary infertility and secondary infertility attributable to endometriosis, PCOS, and unexplained infertility for WCBA in 2021. Age‐standardized YLDs rates of infertility (A), primary infertility (B), and secondary infertility (C). Proportions of YLDs cases of infertility (D), primary infertility (E), and secondary infertility (F). PCOS: polycystic ovarian syndrome; WCBA: women of childbearing age; YLDs: years lived with disability.
**Figure S5**: Proportion of prevalence and YLDs for primary infertility and secondary infertility attributable to endometriosis, PCOS, and unexplained infertility by global and 21 GBD regions in 2021. The proportion of age‐standardized prevalence rate for primary infertility and secondary infertility attributable to endometriosis(A), unexplained infertility (B), and polycystic ovarian syndrome (C). The proportion of age‐standardized YLDs rate for primary infertility and secondary infertility attributable to endometriosis (D), unexplained infertility (E), and polycystic ovarian syndrome (F). PCOS: polycystic ovarian syndrome; WCBA: women of childbearing age; YLDs: years lived with disability.
**Table S1**: International Classification of Diseases (ICD) codes mapped to endometriosis and PCOS in GBD 2021.
**Table S2**: Prevalence and DALYs cases and age‐standardized rate of endometriosis for WCBA in 1990 and 2021, and their average annual percentage change from 1990 to 2021 by countries and territories.
**Table S3**: Prevalence and DALYs cases and age‐standardized rate of polycystic ovarian syndrome for WCBA in 1990 and 2021, and their average annual percentage change from 1990 to 2021 by countries and territories.
**Table S4**: Prevalence and DALYs cases and age‐standardized rate of unexplained infertility for WCBA in 1990 and 2021, and their average annual percentage change from 1990 to 2021 by countries and territories.
**Table S5**: Prevalence and YLDs cases and age‐standardized rate of infertility attributable to endometriosis, PCOS, and unexplained infertility for WCBA in 2021 by location.
**Table S6**: Prevalence and YLDs cases and age‐standardized rate of primary infertility attributable to endometriosis, PCOS, and unexplained infertility for WCBA in 2021 by location.
**Table S7**: Prevalence and YLDs cases and age‐standardized rate of secondary infertility attributable to endometriosis, PCOS, and unexplained infertility for WCBA in 2021 by location.

## References

[1] GBD 2021 Fertility and Forecasting Collaborators , “Global Fertility in 204 Countries and Territories, 1950–2021, With Forecasts to 2100: A Comprehensive Demographic Analysis for the Global Burden of Disease Study 2021,” Lancet 403, no. 10440 (2024): 2057–2099. 38521087 10.1016/S0140-6736(24)00550-6 PMC11122687

[2] The Lancet Global Health , “Infertility—Why the Silence?,” Lancet Global Health 10, no. 6 (2022): e773. 35561706 10.1016/S2214-109X(22)00215-7

[3] World Health Organization . Infertility (World Health Organization, 2024).

[4] P. Njagi , W. Groot , J. Arsenijevic , S. Dyer , G. Mburu , and J. Kiarie , “Financial Costs of Assisted Reproductive Technology for Patients in Low‐ and Middle‐Income Countries: A Systematic Review,” Human Reproduction Open 2023, no. 2 (2023): hoad007. 36959890 10.1093/hropen/hoad007 PMC10029849

[5] X. Liu , J. Zhang , and S. Wang , “Global, Regional, and National Burden of Infertility Attributable to PCOS, 1990–2019,” Human Reproduction 39, no. 1 (2024): 108–118. 38011904 10.1093/humrep/dead241

[6] H. P. Yang , L. S. Cook , E. Weiderpass , et al., “Infertility and Incident Endometrial Cancer Risk: A Pooled Analysis From the Epidemiology of Endometrial Cancer Consortium (E2C2),” British Journal of Cancer 112, no. 5 (2015): 925–933. 25688738 10.1038/bjc.2015.24 PMC4453954

[7] Z. Fan , H. Song , R. Yuan , Y. Peng , and Y. Jiang , “Genetic Predisposition to Female Infertility in Relation to Epithelial Ovarian and Endometrial Cancers,” Postgraduate Medical Journal 99, no. 1168 (2023): 63–68. 36856662 10.1093/postmj/qgad009

[8] Y. Wang , Y. Fu , P. Ghazi , et al., “Prevalence of Intimate Partner Violence Against Infertile Women in Low‐Income and Middle‐income Countries: A Systematic Review and Meta‐Analysis,” The Lancet Global Health 10, no. 6 (2022): e820–e830. 35561719 10.1016/S2214-109X(22)00098-5 PMC9115867

[9] World Health Organization . Infertility Prevalence Estimates, 1990–2021 (World Health Organization, 2023).

[10] M. C. Inhorn and P. Patrizio , “Infertility Around the Globe: New Thinking on Gender, Reproductive Technologies and Global Movements in the 21st Century,” Human Reproduction Update 21, no. 4 (2015): 411–426. 25801630 10.1093/humupd/dmv016

[11] Infertility Workup for the Women's Health Specialist: ACOG Committee Opinion, Number 781. Obstetrics and Gynecology 133, no. 6 (2019): e377–e384. 31135764 10.1097/AOG.0000000000003271

[12] S. A. Carson and A. N. Kallen , “Diagnosis and Management of Infertility: A Review,” Jama 326, no. 1 (2021): 65–76. 34228062 10.1001/jama.2021.4788 PMC9302705

[13] P. Sun , C. Yu , L. Yin , et al., “Global, Regional, and National Burden of Female Cancers in Women of Child‐Bearing Age, 1990–2021: Analysis of Data From the Global Burden of Disease Study 2021,” EClinicalMedicine 74 (2024): 102713. 39050105 10.1016/j.eclinm.2024.102713 PMC11268131

[14] H. F. Escobar‐Morreale , “Polycystic Ovary Syndrome: Definition, Aetiology, Diagnosis and Treatment,” Nature Reviews Endocrinology 14, no. 5 (2018): 270–284. 10.1038/nrendo.2018.24 29569621

[15] C. Chapron , L. Marcellin , B. Borghese , and P. Santulli , “Rethinking Mechanisms, Diagnosis and Management of Endometriosis,” Nature Reviews Endocrinology 15, no. 11 (2019): 666–682. 10.1038/s41574-019-0245-z 31488888

[16] J. Applebaum , E. K. Kim , M. Sharp , A. Dokras , and D. K. Shah , “Racial and Socioeconomic Disparities in Fertility Treatment Provision for Patients With Polycystic Ovary Syndrome,” Fertility and Sterility 122, no. 5 (2024): 928–937. 38909670 10.1016/j.fertnstert.2024.06.014

[17] B. Swift , B. Taneri , C. M. Becker , et al., “Prevalence, Diagnostic Delay and Economic Burden of Endometriosis and Its Impact on Quality of Life: Results From an Eastern Mediterranean Population,” European Journal of Public Health 34, no. 2 (2024): 244–252. 38070492 10.1093/eurpub/ckad216 PMC10990517

[18] C. M. Cox , M. E. Thoma , N. Tchangalova , et al., “Infertility Prevalence and the Methods of Estimation From 1990 to 2021: A Systematic Review and Meta‐Analysis,” Human Reproduction Open 2022, no. 4 (2022): hoac051. 36483694 10.1093/hropen/hoac051 PMC9725182

[19] D. Wijeratne , J. F. E. Gibson , A. Fiander , E. Rafii‐Tabar , and R. Thakar , “The Global Burden of Disease due to Benign Gynecological Conditions: A Call to Action,” International Journal of Gynaecology and Obstetrics 164, no. 3 (2024): 1151–1159. 37987165 10.1002/ijgo.15211

[20] S. Safiri , M. Noori , S. A. Nejadghaderi , et al., “Prevalence, Incidence and Years Lived With Disability due to Polycystic Ovary Syndrome in 204 Countries and territories, 1990–2019,” Human Reproduction 37, no. 8 (2022): 1919–1931. 35586937 10.1093/humrep/deac091

[21] S. Zhang , T. T. Gong , H. Y. Wang , Y. H. Zhao , and Q. J. Wu , “Global, Regional, and National Endometriosis Trends From 1990 to 2017,” Annals of the New York Academy of Sciences 1484, no. 1 (2021): 90–101. 32909625 10.1111/nyas.14468

[22] GBD 2021 Diseases and Injuries Collaborators . Global Incidence, Prevalence, Years Lived With Disability (YLDs), Disability‐Adjusted Life‐Yyears (DALYs), and Healthy Life Expectancy (HALE) for 371 Diseases and Injuries in 204 Countries and Territories and 811 Subnational Locations, 1990–2021: A Systematic Analysis for the Global Burden of Disease Study 2021. Lancet 403, no. 10440 (2024): 2133–2161. 38642570 10.1016/S0140-6736(24)00757-8 PMC11122111

[23] GBD 2019 Diseases and Injuries Collaborators . Global Burden of 369 Diseases and Injuries in 204 Countries and territories, 1990–2019: A Systematic Analysis for the Global Burden of Disease Study 2019. Lancet 396, no. 10258 (2020): 1204–1222. 33069326 10.1016/S0140-6736(20)30925-9 PMC7567026

[24] Y. Liang , J. Huang , Q. Zhao , et al., “Global, Regional, and National Prevalence and Trends of Infertility Among Individuals of Reproductive Age (15‐49 years) From 1990 to 2021, With Projections to 2040,” Human Reproduction 40, no. 3 (2025): 529–544. 39752330 10.1093/humrep/deae292

[25] World Health Organization . Women of Reproductive Age (15‐49 years) Population (World Health Organization).

[26] L. Fu , T. Tian , B. Wang , et al., “Global, Regional, and National Burden of HIV and Other Sexually Transmitted Infections in Older Adults Aged 60–89 Years From 1990 to 2019: Results From the Global Burden of Disease Study 2019,” Lancet Healthy Longev 5, no. 1 (2024): e17–e30. 38183996 10.1016/S2666-7568(23)00214-3

[27] M. Ghiasi , M. T. Kulkarni , and S. A. Missmer , “Is Endometriosis More Common and More Severe Than It Was 30 Years Ago?,” Journal of Minimally Invasive Gynecology 27, no. 2 (2020): 452–461. 31816389 10.1016/j.jmig.2019.11.018

[28] A. W. Horne and S. A. Missmer , “Pathophysiology, Diagnosis, and Management of Endometriosis,” Bmj 379 (2022): e070750. 36375827 10.1136/bmj-2022-070750

[29] S. K. Agarwal , C. Chapron , L. C. Giudice , et al., “Clinical Diagnosis of Endometriosis: A Call to Action,” *American Journal of Obstetrics and Gynecology* 220, no. 4 (2019): 354.e1–.e12. 10.1016/j.ajog.2018.12.039 30625295

[30] J. P. Christ , O. Yu , R. Schulze‐Rath , J. Grafton , K. Hansen , and S. D. Reed , “Incidence, Prevalence, and Trends in Endometriosis Diagnosis: A United States Population‐based Study From 2006 to 2015,” American Journal of Obstetrics and Gynecology 225, no. 5 (2021): 500.e1–.e9. 10.1016/j.ajog.2021.06.067 34147493

[31] D. Y. Shen , J. Li , P. Hu , C. Qi , and H. Yang , “Global, Regional, and National Prevalence and Disability‐Adjusted Life‐Years for Endometriosis in 204 Countries and Territories, 1990–2019: Findings From a Global Burden of Disease Study,” European Journal of Obstetrics & Gynecology and Reproductive Biology: X 25 (2025): 100363. 39850250 10.1016/j.eurox.2024.100363 PMC11754495

[32] T. E. Collins , S. Akselrod , R. Atun , et al., “Converging Global Health Agendas and Universal Health Coverage: Financing Whole‐of‐Government Action Through UHC,” The Lancet Global Health 11, no. 12 (2023): e1978–e1985. 37973345 10.1016/S2214-109X(23)00489-8 PMC10664822

[33] D. Mirkin , C. Murphy‐Barron , and K. Iwasaki , “Actuarial Analysis of Private Payer Administrative Claims Data for Women With Endometriosis,” Journal of Managed Care Pharmacy 13, no. 3 (2007): 262–272. 17407392 10.18553/jmcp.2007.13.3.262 PMC10437570

[34] V. H. Eisenberg , C. Weil , G. Chodick , and V. Shalev , “Epidemiology of Endometriosis: A Large Population‐Based Database Study From a Healthcare Provider With 2 Million Members,” Bjog 125, no. 1 (2018): 55–62. 28444957 10.1111/1471-0528.14711

[35] J. Y. Shim and M. R. Laufer , “Adolescent Endometriosis: An Update,” Journal of Pediatric and Adolescent Gynecology 33, no. 2 (2020): 112–119. 31812704 10.1016/j.jpag.2019.11.011

[36] M. Hirsch , R. Dhillon‐Smith , A. S. Cutner , M. Yap , and S. M. Creighton , “The Prevalence of Endometriosis in Adolescents With Pelvic Pain: A Systematic Review,” Journal of Pediatric and Adolescent Gynecology 33, no. 6 (2020): 623–630. 32736134 10.1016/j.jpag.2020.07.011

[37] B. Jiang , “The Global Burden of Polycystic Ovary Syndrome in Women of Reproductive Age: Findings From the GBD 2019 Study,” International Journal of Women's Health 17 (2025): 153–165. 10.2147/IJWH.S490836 PMC11776423 39882398

[38] R. Deswal , V. Narwal , A. Dang , and C. S. Pundir , “The Prevalence of Polycystic Ovary Syndrome: A Brief Systematic Review,” Journal of Human Reproductive Sciences 13, no. 4 (2020): 261–271. 33627974 10.4103/jhrs.JHRS_95_18 PMC7879843

[39] M. Blüher , “Obesity: Global Epidemiology and Pathogenesis,” Nature reviews Endocrinology 15, no. 5 (2019): 288–298. 10.1038/s41574-019-0176-8 30814686

[40] S. S. Venkatesh , T. Ferreira , S. Benonisdottir , et al., “Obesity and Risk of Female Reproductive Conditions: A Mendelian Randomisation Study,” PLoS Medicine 19, no. 2 (2022): e1003679. 35104295 10.1371/journal.pmed.1003679 PMC8806071

[41] M. Kessler , E. Thumé , M. Marmot , et al., “Family Health Strategy, Primary Health Care, and Social Inequalities in Mortality Among Older Adults in Bagé, Southern Brazil,” American Journal of Public Health 111, no. 5 (2021): 927–936. 33734851 10.2105/AJPH.2020.306146 PMC8034023

[42] T. Hone , V. Saraceni , C. Medina Coeli , et al., “Primary Healthcare Expansion and Mortality in Brazil's Urban Poor: A Cohort Analysis of 1.2 Million Adults,” PLoS Medicine 17, no. 10 (2020): e1003357. 33125387 10.1371/journal.pmed.1003357 PMC7598481

[43] J. Macinko and M. J. Harris , “Brazil's Family Health Strategy–Delivering Community‐Based Primary Care in a Universal Health System,” New England Journal of Medicine 372, no. 23 (2015): 2177–2181. 26039598 10.1056/NEJMp1501140

[44] F. Chiaffarino , S. Cipriani , M. Dalmartello , et al., “Prevalence of Polycystic Ovary Syndrome in European Countries and USA: A Systematic Review and Meta‐Analysis,” European Journal of Obstetrics, Gynecology, and Reproductive Biology 279 (2022): 159–170. 36343588 10.1016/j.ejogrb.2022.10.020

[45] W. Kopp , “How Western Diet and Lifestyle Drive the Pandemic of Obesity and Civilization Diseases,” Diabetes, Metabolic Syndrome, and Obesity 12 (2019): 2221–2236. 10.2147/DMSO.S216791 PMC6817492 31695465

[46] K. Motlagh Asghari , S. A. Nejadghaderi , and M. Alizadeh , “Burden of Polycystic Ovary Syndrome in the Middle East and North Africa Region, 1990–2019,” Scientific Reports 12, no. 1 (2022). 10.1038/s41598-022-11006-0 PMC9052181 35488014

[47] Y. Wang , W. Wang , H. Li , and Q. Du , “Trends in the Burden of Female Infertility Among Adults Aged 20–49 Years During 1990–2019: An Analysis of Data From the Global Burden of Disease Study 2019,” BMJ Open 14, no. 7 (2024): e084755. 10.1136/bmjopen-2024-084755 PMC11293414 39079919

[48] Y. Wei , Z. Lin , Q. Huang , H. Wu , R. Wang , and J. Wang , “Burden of Female Infertility in 204 Countries and territories, 1990–2021: Results From the Global Burden of Disease Study 2021,” Journal of Psychosomatic Obstetrics and Gynaecology 46, no. 1 (2025): 2459618. 39936646 10.1080/0167482X.2025.2459618

[49] R. Bala , V. Singh , S. Rajender , and K. Singh , “Environment, Lifestyle, and Female Infertility,” Reproductive Sciences 28, no. 3 (2021): 617–638. 32748224 10.1007/s43032-020-00279-3

[50] C. M. Farquhar , S. Bhattacharya , S. Repping , et al., “Female Subfertility,” Nature Reviews Disease Primers 5, no. 1 (2019): 7. 10.1038/s41572-018-0058-8 30679436

[51] S. J. Chua , N. A. Danhof , M. H. Mochtar , et al., “Age‐related Natural Fertility Outcomes in Women Over 35 Years: A Systematic Review and Individual Participant Data Meta‐Analysis,” Human Reproduction 35, no. 8 (2020): 1808–1820. 32696041 10.1093/humrep/deaa129

[52] S. Sharma , S. Mittal , and P. Aggarwal , “Management of Infertility in Low Resource Countries,” Bjog 116, no. Suppl 1 (2009): 77–83. 19740177 10.1111/j.1471-0528.2009.02311.x

[53] B. O. Ahinkorah , E. K. Ameyaw , and A. A. Seidu , “Socio‐Economic and Demographic Predictors of Unmet Need for Contraception Among Young Women in Sub‐Saharan Africa: Evidence From Cross‐Sectional Surveys,” Reproductive Health 17, no. 1 (2020): 163. 33097088 10.1186/s12978-020-01018-2 PMC7585192

[54] G. Sedgh , S. Singh , I. H. Shah , E. Ahman , S. K. Henshaw , and A. Bankole , “Induced Abortion: Incidence and Trends Worldwide From 1995 to 2008,” Lancet 379, no. 9816 (2012): 625–632. 22264435 10.1016/S0140-6736(11)61786-8

[55] H. Sun , T. T. Gong , Y. T. Jiang , S. Zhang , Y. H. Zhao , and Q. J. Wu , “Global, Regional, and National Prevalence and Disability‐adjusted Life‐Years for Infertility in 195 Countries and Territories, 1990–2017: Results From a Global Burden of Disease Study,” Aging (Albany NY) 11, no. 23 (2017): 10952–10991. 10.18632/aging.102497 PMC6932903 31790362

[56] K. Hammarberg and M. Kirkman , “Infertility in Resource‐Constrained Settings: Moving Towards Amelioration,” Reproductive Biomedicine Online 26, no. 2 (2013): 189–195. 23260034 10.1016/j.rbmo.2012.11.009

[57] J. Bellver and J. Donnez , “Introduction: Infertility Etiology and Offspring Health,” Fertility and Sterility 111, no. 6 (2019): 1033–1035. 31155112 10.1016/j.fertnstert.2019.04.043

[58] G. Bonavina and H. S. Taylor , “Endometriosis‐Associated Infertility: From Pathophysiology to Tailored Treatment,” Frontiers in Endocrinology (Lausanne) 13 (2022): 1020827. 10.3389/fendo.2022.1020827 PMC9643365 36387918

[59] D. de Ziegler , P. Pirtea , M. Carbonnel , et al., “Assisted Reproduction in Endometriosis,” Best Practice & Research Clinical Endocrinology & Metabolism 33, no. 1 (2019): 47–59. 30503728 10.1016/j.beem.2018.10.001

[60] D. de Ziegler , B. Borghese , and C. Chapron , “Endometriosis and Infertility: Pathophysiology and Management,” Lancet 376, no. 9742 (2010): 730–738. 20801404 10.1016/S0140-6736(10)60490-4

[61] Thessaloniki ESHRE/ASRM‐Sponsored PCOS Consensus Workshop Group . “Consensus on Infertility Treatment Related to Polycystic Ovary Syndrome.” Fertility and Sterility 89, no. 3 (2008): 505–522. 18243179 10.1016/j.fertnstert.2007.09.041

[62] S. Palomba , T. T. Piltonen , and L. C. Giudice , “Endometrial Function in Women With Polycystic Ovary Syndrome: A Comprehensive Review,” Human Reproduction Update 27, no. 3 (2021): 584–618. 33302299 10.1093/humupd/dmaa051

[63] D. L. Olive and S. L. B. Schwartz , “Endometriosis,” New England Journal of Medicine 328, no. 24 (1993): 1759–1769. 8110213 10.1056/NEJM199306173282407

[64] C. Bandala , J. P. Cifuentes‐Chacón , A. Cortes‐Vázquez , R. Ruz‐Barros , L. Garrocho‐Hernández , and A. Cortes‐Algara , “Efficacy Between Conventional Laparoscopy and Robotic Surgery in Mexican Patients With Endometriosis: A Comparative Study,” Journal of Clinical Medicine 13, no. 12 (2024): 3576. 38930105 10.3390/jcm13123576 PMC11205068

[65] R. G. Almquist , C. M. Barrera , R. Fried , S. L. Boulet , J. F. Kawwass , and H. S. Hipp , “Impact of Access to Care and Race/Ethnicity on IVF Care Discontinuation,” Reproductive Biomedicine Online 44, no. 6 (2022): 1159–1168. 35339366 10.1016/j.rbmo.2021.11.017

[66] B. S. Bedrick , K. Anderson , D. E. Broughton , B. Hamilton , and E. S. Jungheim , “Factors Associated With Early In Vitro Fertilization Treatment Discontinuation,” Fertility and Sterility 112, no. 1 (2019): 105–111. 31043233 10.1016/j.fertnstert.2019.03.007 PMC7299162

[67] R. Gautam , P. Maan , A. Jyoti , A. Kumar , N. Malhotra , and T. Arora , “The Role of Lifestyle Interventions in PCOS Management: A Systematic Review,” Nutrients 17, no. 2 (2025): 310. 39861440 10.3390/nu17020310 PMC11767734

[68] K. M. Hoeger , A. Dokras , and T. Piltonen , “Update on PCOS: Consequences, Challenges, and Guiding Treatment,” Journal of Clinical Endocrinology and Metabolism 106, no. 3 (2021): e1071. 33211867 10.1210/clinem/dgaa839

[69] J. J. Horns , K. Fendereski , J. M. Ramsay , et al., “The Impact of Socioeconomic Status on Bulk Semen Parameters, Fertility Treatment, and Fertility Outcomes in a Cohort of Subfertile Men,” Fertility and Sterility 120, no. 1 (2023): 72–79. 36813124 10.1016/j.fertnstert.2023.02.015 PMC10293094

[70] H. S. Taylor , A. M. Kotlyar , and V. A. Flores , “Endometriosis Is a Chronic Systemic Disease: Clinical Challenges and Novel Innovations,” Lancet 397, no. 10276 (2021): 839–852. 33640070 10.1016/S0140-6736(21)00389-5

[71] G. Ventolini , G. M. Horowitz , and R. Long , “Endometriosis in Adolescence: A Long‐Term Follow‐Up Fecundability Assessment,” Reproductive Biology and Endocrinology [Electronic Resource]: RB&E 3 (2005): 14. 15845149 10.1186/1477-7827-3-14 PMC1131922

[72] Y. Gu , G. Zhou , F. Zhou , et al., “Life Modifications and PCOS: Old Story but New Tales,” Frontiers in Endocrinology (Lausanne) 13 (2022): 808898. 10.3389/fendo.2022.808898 PMC9045543 35498415

