# Supplementary material for: Global, Regional, and National Burden of Endometriosis, PCOS, and Unexplained Infertility and Their Attribution to Infertility, 1990–2021: Global Burden of Disease Study 2021
Source: J Evid Based Med. 2025 Dec 23;18(4):e70100. doi: 10.1111/jebm.70100 (PMC12750491; doi:10.1111/jebm.70100)
Supplement: Supplementary file 1 — Figure S1: Age‐standardized prevalence and DALYs rates of endometriosis for 204 countries and territories in 2021, by SDI. Age‐standardized rates of prevalence (A) and DALYs (B) by SDI. Expected values are shown as a solid line. 204 points are plotted for each country and territory. Points above the solid line represent a higher‐than‐expected burden, while those below the line show a lower‐than‐expected burden. SDI: socio‐demographic index. Figure S2: Age‐standardized prevalence and DALYs rates of PCOS for 204 countries and territories in 2021, by SDI. Age‐standardized rates of prevalence (A) and DALYs (B) by SDI. Expected values are shown as a solid line. 204 points are plotted for each country and territory. Points above the solid line represent a higher‐than‐expected burden, while those below the line show a lower‐than‐expected burden. PCOS: polycystic ovarian syndrome; SDI: socio‐demographic index. Figure S3: Age‐standardized prevalence and DALYs rates of unexplained infertility for 204 countries and territories in 2021, by SDI. Age‐standardized rates of prevalence (A) and DALYs (B) by SDI. Expected values are shown as a solid line. 204 points are plotted for each country and territory. Points above the solid line represent a higher‐than‐expected burden, while those below the line show a lower‐than‐expected burden. SDI, socio‐demographic index. Figure S4: Age‐standardized YLDs rates and proportions of YLDs cases for infertility, primary infertility and secondary infertility attributable to endometriosis, PCOS, and unexplained infertility for WCBA in 2021. Age‐standardized YLDs rates of infertility (A), primary infertility (B), and secondary infertility (C). Proportions of YLDs cases of infertility (D), primary infertility (E), and secondary infertility (F). PCOS: polycystic ovarian syndrome; WCBA: women of childbearing age; YLDs: years lived with disability. Figure S5: Proportion of prevalence and YLDs for primary infertility and secondary infertility attributab [file JEBM-18-0-s001.docx]

**Supplementary Material**

1. **Supplementary Figures**
   1. **Figure. S1:** Age-standardised prevalence and DALYs rates of endometriosis for 204 countries and territories in 2021, by SDI. Age-standardised rates of prevalence (A) and DALYs (B) by SDI; Expected values are shown as a solid line; 204 points are plotted for each country and territory; Points above the solid line represent a higher-than-expected burden, while those below the line show a lower-than-expected burden. SDI: socio-demographic index.
   2. **Figure. S2:** Age-standardised prevalence and DALYs rates of PCOS for 204 countries and territories in 2021, by SDI. Age-standardised rates of prevalence (A) and DALYs (B) by SDI; Expected values are shown as a solid line; 204 points are plotted for each country and territory; Points above the solid line represent a higher-than-expected burden, while those below the line show a lower-than-expected burden. PCOS: polycystic ovarian syndrome; SDI: socio-demographic index.
   3. **Figure. S3:** Age-standardised prevalence and DALYs rates of unexplained infertility for 204 countries and territories in 2021, by SDI. Age-standardised rates of prevalence (A) and DALYs (B) by SDI; Expected values are shown as a solid line; 204 points are plotted for each country and territory; Points above the solid line represent a higher-than-expected burden, while those below the line show a lower-than-expected burden. SDI, socio-demographic index.
   4. **Figure. S4:** Age-standardised YLDs rates and proportions of YLDs cases for infertility, primary infertility and secondary infertility attributable to endometriosis, PCOS and unexplained infertility for WCBA in 2021. Age-standardised YLDs rates of infertility (A), primary infertility (B) and secondary infertility (C). Proportions of YLDs cases of infertility (D), primary infertility (E) and secondary infertility (F). PCOS: polycystic ovarian syndrome; WCBA: Women of childbearing age; YLDs: years lived with disability
   5. **Figure. S5:** Proportion of prevalence and YLDs for primary infertility and secondary infertility attributable to endometriosis, PCOS and unexplained infertility by global and 21 GBD regions in 2021. The proportion of age-standardised prevalence rate for primary infertility and secondary infertility attributable to endometriosis(A), unexplained infertility (B) and polycystic ovarian syndrome (C); The proportion of age-standardised YLDs rate for primary infertility and secondary infertility attributable to endometriosis (D), unexplained infertility (E) and polycystic ovarian syndrome (F). PCOS: polycystic ovarian syndrome; WCBA: Women of childbearing age; YLDs: years lived with disability.
2. **Supplementary Tables**
   1. **Table S1:** International Classification of Diseases (ICD) codes mapped to endometriosis and PCOS in GBD 2021
   2. **Table S2:** Prevalence and DALYs cases and age-standardised rate of endometriosis for WCBA in 1990 and 2021, and their average annual percentage change from 1990 to 2021 by countries and territories.
   3. **Table S3:** Prevalence and DALYs cases and age-standardised rate of polycystic ovarian syndrome for WCBA in 1990 and 2021, and their average annual percentage change from 1990 to 2021 by countries and territories.
   4. **Table S4:** Prevalence and DALYs cases and age-standardised rate of unexplained infertility for WCBA in 1990 and 2021, and their average annual percentage change from 1990 to 2021 by countries and territories.
   5. **Table S5:** Prevalence and YLDs cases and age-standardised rate of infertility attributable to endometriosis, PCOS and unexplained infertility for WCBA in 2021 by location.
   6. **Table S6:** Prevalence and YLDs cases and age-standardised rate of primary infertility attributable to endometriosis, PCOS and unexplained infertility for WCBA in 2021 by location.
   7. **Table S7:** Prevalence and YLDs cases and age-standardised rate of secondary infertility attributable to endometriosis, PCOS and unexplained infertility for WCBA in 2021 by location.

**Figure. S1:** Age-standardised prevalence and DALYs rates of endometriosis for 204 locations in 2021, by SDI. Age-standardised rates of prevalence (A) and DALYs (B) by SDI; Expected values are shown as a solid line; 204 points are plotted for each location; Points above the solid line represent a higher-than-expected burden, while those below the line show a lower-than-expected burden. SDI: socio-demographic index.

**
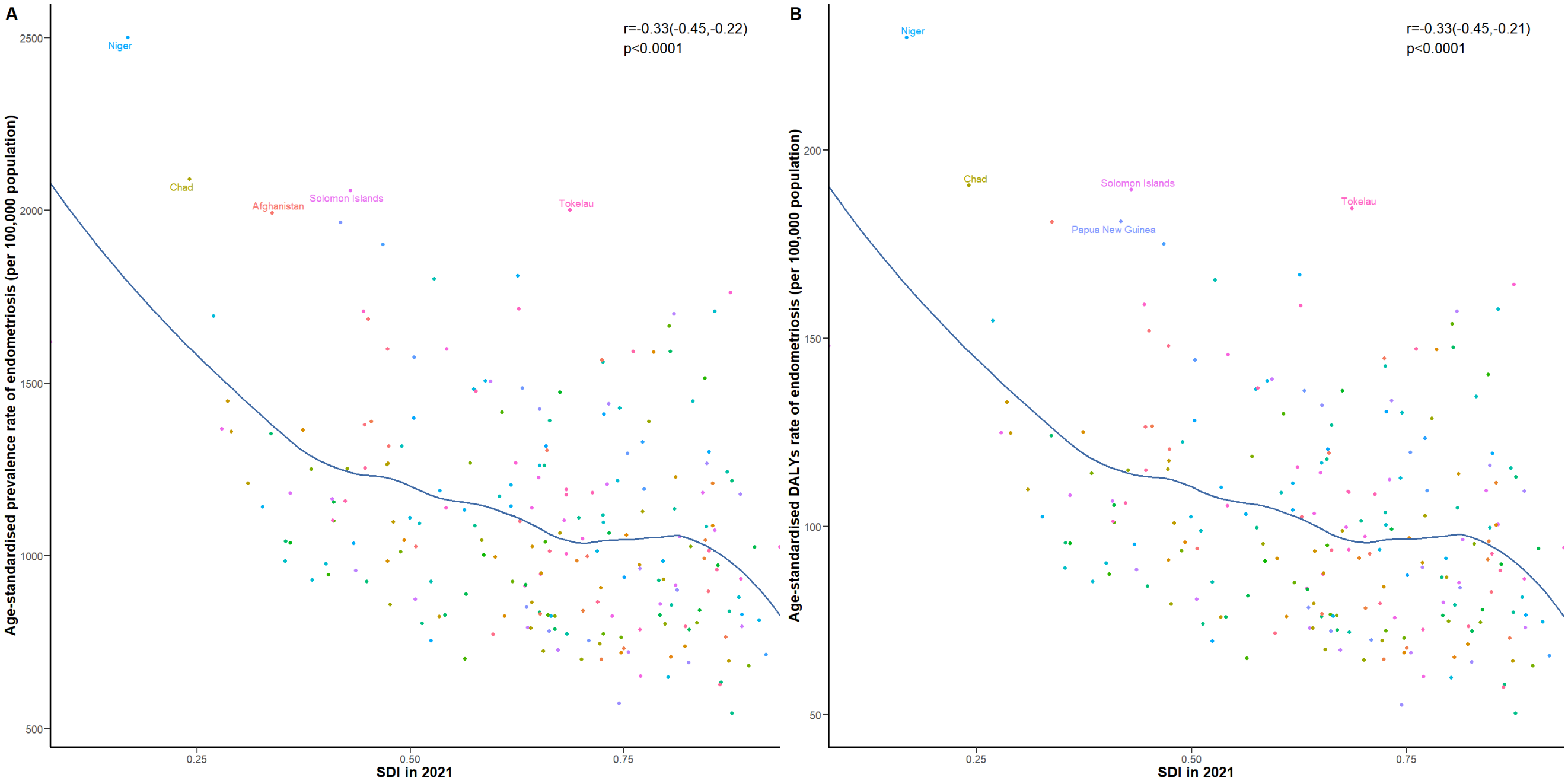
**

**Figure. S2:** Age-standardised prevalence and DALYs rates of PCOS for 204 locations in 2021, by SDI. Age-standardised rates of prevalence (A) and DALYs (B) by SDI; Expected values are shown as a solid line; 204 points are plotted for each location; Points above the solid line represent a higher-than-expected burden, while those below the line show a lower-than-expected burden. PCOS: polycystic ovarian syndrome; SDI: socio-demographic index.


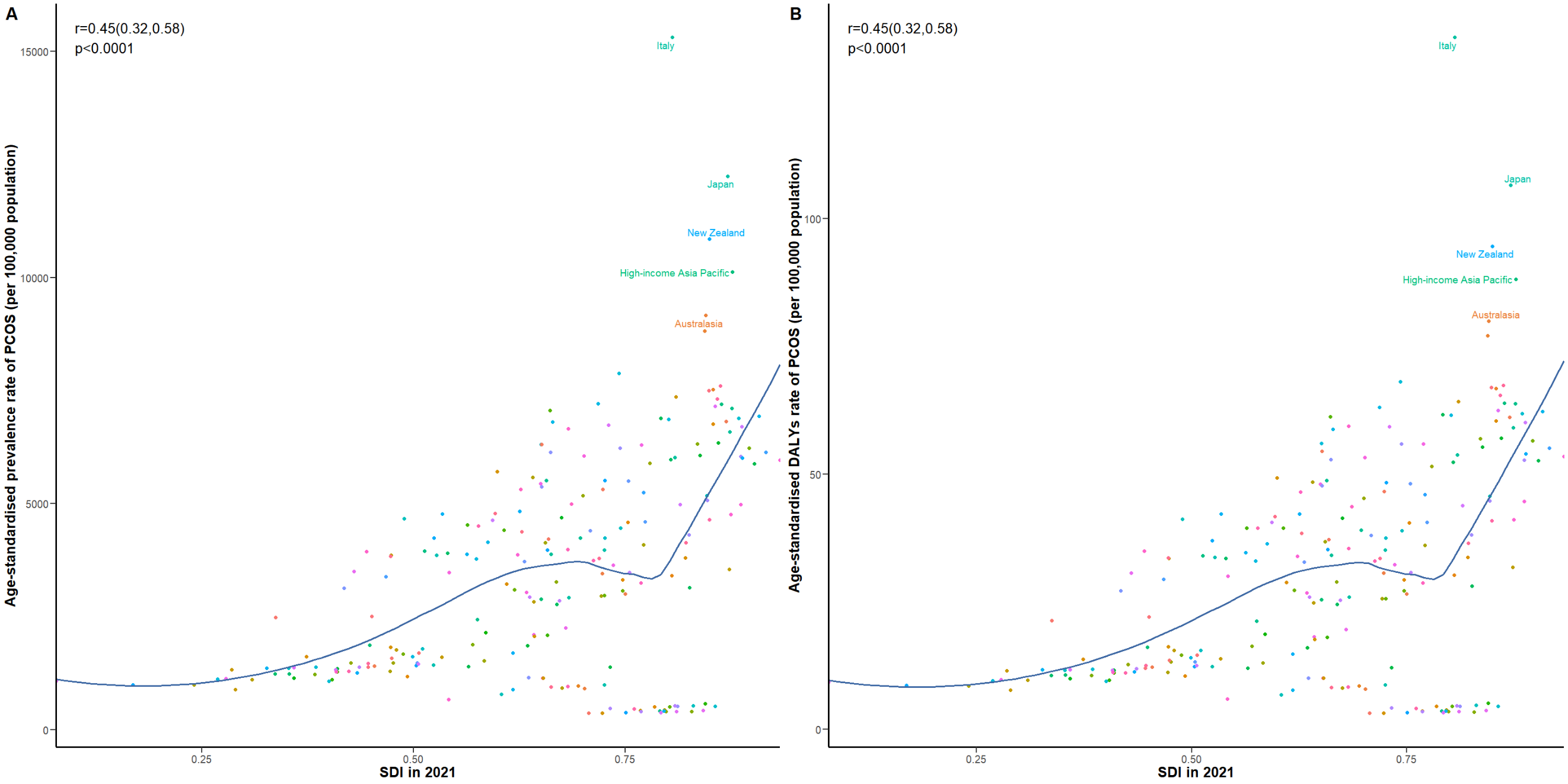


**Figure. S3** Age-standardised prevalence and DALYs rates of unexplained infertility for 204 locations in 2021, by SDI. Age-standardised rates of prevalence (A) and DALYs (B) by SDI; Expected values are shown as a solid line; 204 points are plotted for each location; Points above the solid line represent a higher-than-expected burden, while those below the line show a lower-than-expected burden. SDI, socio-demographic index.


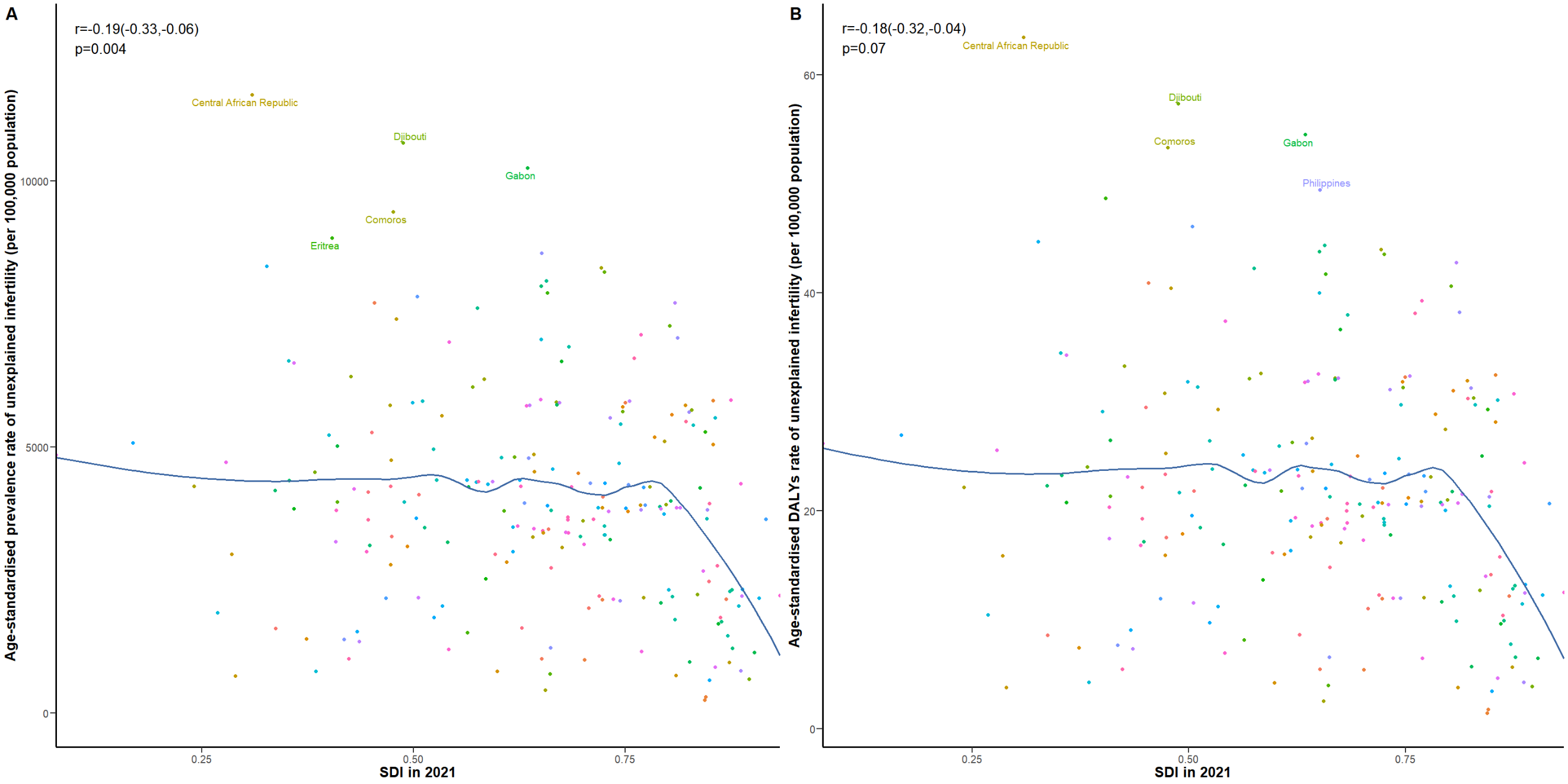


**Figure. S4:** Age-standardised YLDs rates and proportions of YLDs cases for infertility, primary infertility and secondary infertility attributable to endometriosis, PCOS and unexplained infertility for WCBA in 2021. Age-standardised YLDs rates of infertility (A), primary infertility (B) and secondary infertility (C). Proportions of YLDs cases of infertility (D), primary infertility (E) and secondary infertility (F). PCOS: polycystic ovarian syndrome; WCBA: Women of childbearing age; YLDs: years lived with disability


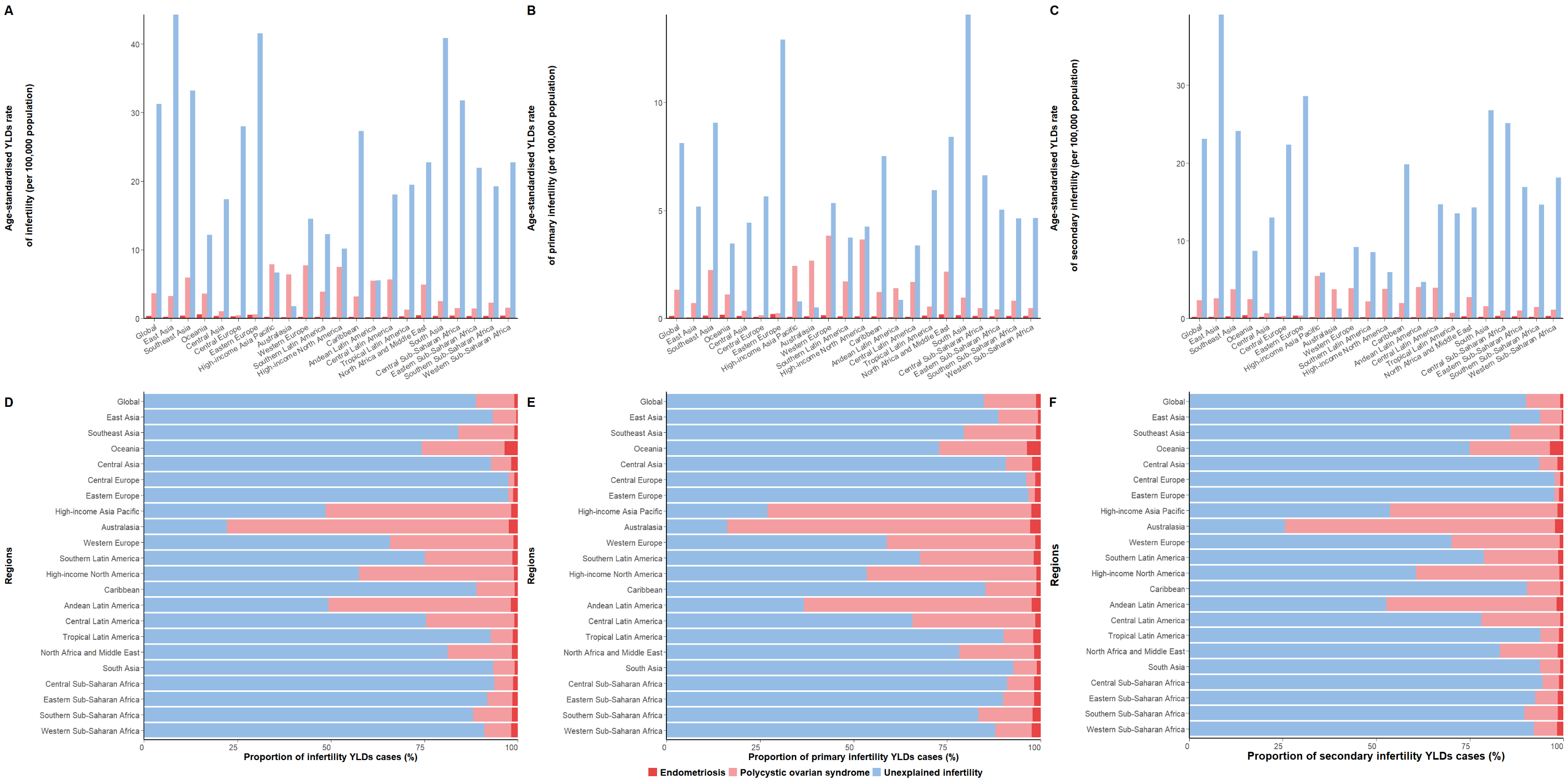


**Figure. S5:** Proportion of prevalence and YLDs for primary infertility and secondary infertility attributable to endometriosis, PCOS and unexplained infertility by global and 21 GBD regions in 2021. The proportion of age-standardised prevalence rate for primary infertility and secondary infertility attributable to endometriosis(A), unexplained infertility (B) and polycystic ovarian syndrome (C); The proportion of age-standardised YLDs rate for primary infertility and secondary infertility attributable to endometriosis (D), unexplained infertility (E) and polycystic ovarian syndrome (F). PCOS: polycystic ovarian syndrome; WCBA: Women of childbearing age; YLDs: years lived with disability.


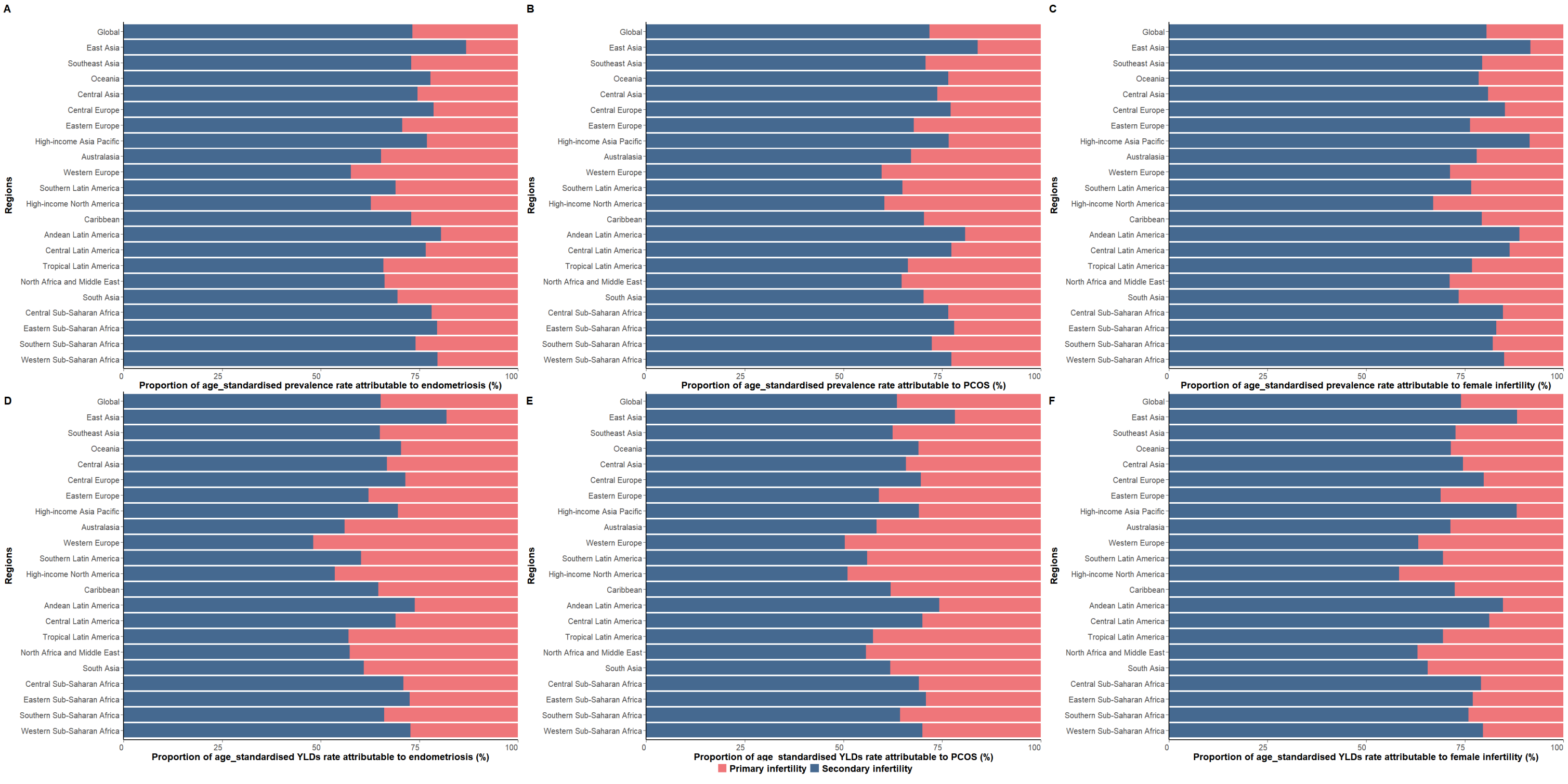


**Table S1:** International Classification of Diseases (ICD) codes mapped to endometriosis and PCOS in GBD 2021

| **Cause Name** | **Cause Hierarchy Level** | **ICD10** | **ICD10 Used in Hospital/Claims Analyses** | **ICD9** | **ICD9 Used in Hospital/Claims Analyses** |
| --- | --- | --- | --- | --- | --- |
| Endometriosis | 4 | N80-N80.9 | N80-N80.9 | 617-617.9 | 617-617.9 |
| Polycystic ovarian syndrome | 4 | E28.2 | E28.2 | 256.4 | 256.4 |

**Table S2:** Prevalence and DALYs cases and age-standardised rate of endometriosis for WCBA in 1990 and 2021, and their average annual percentage change from 1990 to 2021 by countries and territories.

| **Location** |  | **Prevalence** | | | | | | |  |  |  | **DALYs (Disability-Adjusted Life Years)** | | | | | | | | |
| --- | --- | --- | --- | --- | --- | --- | --- | --- | --- | --- | --- | --- | --- | --- | --- | --- | --- | --- | --- | --- |
|  |  | **Number of cases, 1990** |  | **Age-standardised** rate, 1990 |  | **Number of cases, 2021** |  | **Age-standardised** rate, 2021 |  | AAPC, 1990–2021 |  | **Number of cases, 1990** |  | **Age-standardised** rate, 1990 |  | **Number of cases, 2021** |  | **Age-standardised** rate, 2021 |  | AAPC, 1990–2021 |
| China |  | 3,583,587(2,345,186 to 5,133,213) |  | 1161.99(1160.76 to 1163.21) |  | 2,583,355(1,847,000 to 3,439,192) |  | 745.74(744.80 to 746.68) |  | -1.58(-1.77 to -1.39) |  | 334,191(185,052 to 546,554) |  | 108.17(107.79 to 108.54) |  | 241,181(142,227 to 387,507) |  | 69.76(69.48 to 70.05) |  | -1.56(-1.75 to -1.37) |
| Democratic People's Republic of Korea |  | 128,436(87,512 to 175,342) |  | 2326.13(2313.31 to 2339.00) |  | 87,191(60,445 to 120,970) |  | 1269.48(1261.02 to 1277.99) |  | -1.88(-1.99 to -1.77) |  | 11,976(6,828 to 18,725) |  | 216.72(212.82 to 220.68) |  | 8,135(4,829 to 13,014) |  | 118.59(116.01 to 121.21) |  | -1.87(-1.98 to -1.76) |
| Cambodia |  | 55,557(37,785 to 77,310) |  | 2313.70(2294.04 to 2333.50) |  | 57,668(40,116 to 79,561) |  | 1267.94(1257.56 to 1278.38) |  | -1.87(-2.05 to -1.69) |  | 5,121(2,938 to 7,983) |  | 213.02(207.09 to 219.10) |  | 5,350(3,133 to 8,540) |  | 117.53(114.39 to 120.75) |  | -1.82(-2.01 to -1.64) |
| Indonesia |  | 811,895(561,083 to 1,130,963) |  | 1755.82(1751.94 to 1759.72) |  | 966,977(678,093 to 1,337,306) |  | 1261.15(1258.64 to 1263.67) |  | -1.01(-1.07 to -0.95) |  | 75,716(42,817 to 117,838) |  | 163.49(162.30 to 164.68) |  | 90,367(53,161 to 142,137) |  | 117.89(117.12 to 118.66) |  | -0.98(-1.04 to -0.92) |
| Lao People's Democratic Republic |  | 20,229(14,033 to 28,773) |  | 2212.80(2181.78 to 2244.18) |  | 26,050(18,128 to 36,983) |  | 1318.23(1302.18 to 1334.44) |  | -1.83(-1.95 to -1.71) |  | 1,873(1,074 to 2,892) |  | 204.55(195.20 to 214.27) |  | 2,422(1,374 to 3,791) |  | 122.47(117.61 to 127.49) |  | -1.80(-1.92 to -1.69) |
| Malaysia |  | 66,581(45,521 to 95,895) |  | 1518.67(1506.95 to 1530.46) |  | 104,339(72,067 to 147,011) |  | 1218.34(1210.94 to 1225.77) |  | -0.86(-1.00 to -0.73) |  | 6,181(3,514 to 9,689) |  | 140.77(137.22 to 144.40) |  | 9,674(5,518 to 15,023) |  | 112.91(110.67 to 115.19) |  | -0.85(-0.99 to -0.72) |
| Maldives |  | 1,109(747 to 1,546) |  | 2523.75(2369.22 to 2686.96) |  | 1,524(1,068 to 2,132) |  | 1261.98(1198.52 to 1328.29) |  | -2.03(-2.34 to -1.73) |  | 102(58 to 159) |  | 231.75(186.70 to 285.86) |  | 141(81 to 225) |  | 116.92(98.17 to 138.62) |  | -2.01(-2.31 to -1.71) |
| Myanmar |  | 177,437(121,571 to 253,285) |  | 1767.19(1758.76 to 1775.66) |  | 178,950(125,369 to 249,106) |  | 1188.59(1183.09 to 1194.11) |  | -1.21(-1.27 to -1.15) |  | 16,459(9,248 to 25,718) |  | 163.63(161.07 to 166.22) |  | 16,622(9,896 to 25,880) |  | 110.40(108.73 to 112.09) |  | -1.19(-1.24 to -1.13) |
| Philippines |  | 248,629(174,192 to 344,239) |  | 1686.08(1679.31 to 1692.87) |  | 412,601(288,033 to 574,482) |  | 1425.02(1420.66 to 1429.39) |  | -0.54(-0.59 to -0.49) |  | 23,065(13,161 to 35,842) |  | 156.14(154.09 to 158.21) |  | 38,287(22,007 to 58,492) |  | 132.16(130.83 to 133.49) |  | -0.52(-0.57 to -0.48) |
| Sri Lanka |  | 55,185(38,825 to 76,269) |  | 1215.45(1205.26 to 1225.71) |  | 59,801(42,530 to 81,431) |  | 1050.83(1042.39 to 1059.32) |  | -0.36(-0.40 to -0.32) |  | 5,100(2,918 to 7,955) |  | 112.22(109.14 to 115.37) |  | 5,531(3,179 to 8,716) |  | 97.28(94.73 to 99.89) |  | -0.34(-0.39 to -0.30) |
| Thailand |  | 185,911(133,152 to 256,858) |  | 1173.80(1168.41 to 1179.22) |  | 170,908(121,541 to 226,253) |  | 1006.27(1001.38 to 1011.18) |  | -0.44(-0.48 to -0.40) |  | 17,265(10,077 to 26,461) |  | 108.90(107.26 to 110.56) |  | 15,910(9,121 to 25,490) |  | 93.86(92.37 to 95.37) |  | -0.42(-0.46 to -0.38) |
| Timor-Leste |  | 4,868(3,283 to 6,892) |  | 2655.09(2579.29 to 2732.78) |  | 5,510(3,716 to 7,992) |  | 1708.20(1662.07 to 1755.38) |  | -1.63(-1.77 to -1.49) |  | 450(259 to 710) |  | 245.21(222.58 to 269.78) |  | 514(285 to 808) |  | 159.02(145.18 to 173.92) |  | -1.59(-1.72 to -1.45) |
| Viet Nam |  | 228,388(156,420 to 317,756) |  | 1380.01(1374.09 to 1385.96) |  | 291,767(206,325 to 402,653) |  | 1099.30(1095.27 to 1103.34) |  | -0.56(-0.62 to -0.50) |  | 21,277(12,579 to 33,367) |  | 128.24(126.44 to 130.06) |  | 27,225(16,020 to 43,264) |  | 102.61(101.38 to 103.85) |  | -0.55(-0.60 to -0.49) |
| Fiji |  | 3,214(2,153 to 4,568) |  | 1687.42(1628.91 to 1747.65) |  | 3,369(2,293 to 4,736) |  | 1473.81(1424.44 to 1524.47) |  | -0.34(-0.40 to -0.28) |  | 298(169 to 464) |  | 156.22(138.79 to 175.39) |  | 311(178 to 477) |  | 136.12(121.41 to 152.14) |  | -0.35(-0.41 to -0.29) |
| Kiribati |  | 410(276 to 584) |  | 2249.91(2032.23 to 2487.04) |  | 568(382 to 797) |  | 1801.66(1655.87 to 1957.61) |  | -0.64(-0.67 to -0.61) |  | 38(22 to 59) |  | 206.38(144.47 to 289.01) |  | 52(29 to 82) |  | 165.56(123.57 to 218.17) |  | -0.63(-0.67 to -0.60) |
| Marshall Islands |  | 192(130 to 274) |  | 2128.83(1824.87 to 2479.97) |  | 215(145 to 301) |  | 1483.47(1291.68 to 1696.37) |  | -0.95(-1.06 to -0.85) |  | 18(10 to 28) |  | 196.31(112.58 to 333.72) |  | 20(11 to 31) |  | 136.44(83.06 to 212.13) |  | -0.97(-1.07 to -0.86) |
| Micronesia (Federated States of) |  | 552(373 to 775) |  | 2595.51(2376.59 to 2832.09) |  | 379(257 to 528) |  | 1507.13(1358.46 to 1668.10) |  | -1.63(-1.76 to -1.50) |  | 51(29 to 81) |  | 240.20(177.10 to 322.13) |  | 35(20 to 56) |  | 138.74(96.39 to 193.93) |  | -1.63(-1.76 to -1.50) |
| Papua New Guinea |  | 21,970(14,673 to 31,227) |  | 2403.76(2371.29 to 2436.62) |  | 50,712(34,087 to 71,310) |  | 1965.88(1948.71 to 1983.17) |  | -0.66(-0.69 to -0.63) |  | 2,019(1,131 to 3,230) |  | 220.36(210.62 to 230.49) |  | 4,676(2,584 to 7,512) |  | 181.02(175.84 to 186.32) |  | -0.64(-0.68 to -0.60) |
| Samoa |  | 549(380 to 778) |  | 1631.88(1494.01 to 1780.15) |  | 698(489 to 973) |  | 1504.74(1394.36 to 1621.90) |  | -0.05(-0.13 to 0.04) |  | 51(29 to 80) |  | 151.39(111.60 to 202.10) |  | 65(38 to 100) |  | 139.11(107.05 to 178.19) |  | -0.06(-0.15 to 0.03) |
| Solomon Islands |  | 2,253(1,507 to 3,110) |  | 3372.24(3229.15 to 3520.63) |  | 3,399(2,252 to 4,704) |  | 2058.36(1989.44 to 2129.15) |  | -1.63(-1.65 to -1.61) |  | 209(119 to 322) |  | 311.18(268.86 to 358.96) |  | 313(177 to 493) |  | 189.52(169.03 to 211.91) |  | -1.63(-1.65 to -1.61) |
| Tonga |  | 417(284 to 573) |  | 2037.82(1843.17 to 2248.43) |  | 416(282 to 588) |  | 1715.33(1553.98 to 1889.16) |  | -0.47(-0.50 to -0.43) |  | 39(22 to 61) |  | 188.96(133.20 to 261.46) |  | 39(23 to 61) |  | 158.73(112.49 to 217.94) |  | -0.47(-0.50 to -0.43) |
| Vanuatu |  | 802(545 to 1,147) |  | 2418.31(2249.10 to 2598.38) |  | 1,218(824 to 1,686) |  | 1599.52(1510.27 to 1692.90) |  | -1.35(-1.40 to -1.31) |  | 74(41 to 119) |  | 223.41(174.20 to 284.01) |  | 113(65 to 178) |  | 148.08(121.84 to 178.56) |  | -1.35(-1.40 to -1.31) |
| Armenia |  | 10,100(6,874 to 14,579) |  | 1122.20(1099.83 to 1144.97) |  | 6,415(4,543 to 8,597) |  | 841.98(820.95 to 863.50) |  | -0.72(-0.90 to -0.54) |  | 942(538 to 1,493) |  | 104.52(97.78 to 111.67) |  | 595(353 to 923) |  | 78.26(71.93 to 85.07) |  | -0.71(-0.89 to -0.54) |
| Azerbaijan |  | 24,297(16,564 to 34,682) |  | 1253.77(1237.25 to 1270.50) |  | 28,240(19,680 to 40,606) |  | 986.14(974.52 to 997.88) |  | -0.43(-0.58 to -0.27) |  | 2,269(1,316 to 3,572) |  | 116.77(111.77 to 121.97) |  | 2,622(1,535 to 4,107) |  | 91.59(88.07 to 95.23) |  | -0.41(-0.57 to -0.25) |
| Georgia |  | 16,386(11,283 to 23,511) |  | 1160.82(1143.03 to 1178.83) |  | 8,538(6,323 to 11,273) |  | 1066.45(1043.44 to 1089.89) |  | 0.04(-0.10 to 0.18) |  | 1,528(890 to 2,409) |  | 108.22(102.84 to 113.83) |  | 793(479 to 1,203) |  | 99.21(92.27 to 106.59) |  | 0.04(-0.10 to 0.18) |
| Kazakhstan |  | 49,833(34,195 to 69,869) |  | 1189.72(1179.16 to 1200.36) |  | 54,142(36,866 to 75,982) |  | 1118.51(1109.00 to 1128.09) |  | 0.26(0.05 to 0.47) |  | 4,641(2,585 to 7,399) |  | 110.63(107.43 to 113.91) |  | 5,018(2,849 to 7,758) |  | 103.73(100.85 to 106.69) |  | 0.27(0.06 to 0.48) |
| Kyrgyzstan |  | 14,530(9,789 to 21,126) |  | 1387.11(1363.75 to 1410.85) |  | 20,647(13,885 to 29,174) |  | 1173.29(1157.28 to 1189.48) |  | -0.09(-0.33 to 0.16) |  | 1,350(768 to 2,163) |  | 128.58(121.54 to 135.99) |  | 1,919(1,106 to 3,023) |  | 109.00(104.16 to 114.01) |  | -0.07(-0.32 to 0.18) |
| Mongolia |  | 7,860(5,281 to 11,097) |  | 1565.62(1529.42 to 1602.59) |  | 10,445(7,121 to 14,726) |  | 1205.58(1182.40 to 1229.13) |  | -0.22(-0.59 to 0.15) |  | 728(423 to 1,147) |  | 144.62(133.78 to 156.24) |  | 965(538 to 1,531) |  | 111.51(104.54 to 118.86) |  | -0.20(-0.57 to 0.17) |
| Tajikistan |  | 21,816(14,360 to 31,306) |  | 1846.32(1820.16 to 1872.84) |  | 29,468(19,967 to 42,099) |  | 1139.44(1126.36 to 1152.65) |  | -1.30(-1.47 to -1.13) |  | 2,025(1,146 to 3,224) |  | 170.92(163.04 to 179.16) |  | 2,731(1,535 to 4,196) |  | 105.48(101.53 to 109.56) |  | -1.28(-1.45 to -1.12) |
| Turkmenistan |  | 14,396(9,607 to 20,641) |  | 1635.57(1607.47 to 1664.13) |  | 14,878(9,975 to 20,944) |  | 1176.86(1158.01 to 1195.94) |  | -0.76(-0.99 to -0.54) |  | 1,332(739 to 2,149) |  | 150.87(142.44 to 159.77) |  | 1,382(787 to 2,126) |  | 109.27(103.58 to 115.20) |  | -0.74(-0.97 to -0.52) |
| Uzbekistan |  | 69,775(46,912 to 101,293) |  | 1435.59(1424.27 to 1446.99) |  | 92,792(64,431 to 131,029) |  | 1013.28(1006.75 to 1019.85) |  | -0.89(-1.09 to -0.68) |  | 6,459(3,564 to 10,039) |  | 132.55(129.13 to 136.05) |  | 8,587(5,064 to 13,881) |  | 93.79(91.81 to 95.80) |  | -0.87(-1.08 to -0.66) |
| Albania |  | 10,841(7,532 to 15,593) |  | 1289.25(1264.47 to 1314.45) |  | 6,275(4,386 to 8,920) |  | 998.22(973.59 to 1023.36) |  | -0.85(-1.00 to -0.71) |  | 1,016(580 to 1,600) |  | 120.64(113.15 to 128.55) |  | 583(349 to 933) |  | 92.82(85.41 to 100.74) |  | -0.87(-1.01 to -0.72) |
| Bosnia and Herzegovina |  | 11,689(8,045 to 16,325) |  | 988.32(970.45 to 1006.46) |  | 6,726(4,690 to 9,524) |  | 907.44(885.43 to 929.91) |  | -0.19(-0.28 to -0.10) |  | 1,090(613 to 1,714) |  | 92.11(86.71 to 97.77) |  | 621(362 to 977) |  | 84.03(77.42 to 91.11) |  | -0.19(-0.29 to -0.10) |
| Bulgaria |  | 21,359(14,999 to 29,917) |  | 1035.32(1021.36 to 1049.43) |  | 13,960(9,787 to 19,359) |  | 974.29(957.41 to 991.42) |  | 0.06(-0.04 to 0.17) |  | 1,980(1,143 to 3,100) |  | 96.07(91.84 to 100.44) |  | 1,291(736 to 2,011) |  | 90.38(85.28 to 95.73) |  | 0.07(-0.03 to 0.17) |
| Croatia |  | 11,440(7,819 to 16,068) |  | 936.39(919.23 to 953.80) |  | 7,259(5,360 to 9,629) |  | 803.13(784.39 to 822.26) |  | -0.56(-0.76 to -0.35) |  | 1,067(614 to 1,724) |  | 87.42(82.23 to 92.87) |  | 675(430 to 1,034) |  | 74.84(69.19 to 80.88) |  | -0.55(-0.76 to -0.35) |
| Czechia |  | 27,641(19,464 to 39,233) |  | 1095.04(1081.99 to 1108.22) |  | 23,693(16,314 to 32,875) |  | 1028.12(1014.36 to 1042.04) |  | -0.02(-0.25 to 0.22) |  | 2,567(1,503 to 4,096) |  | 101.88(97.92 to 105.96) |  | 2,190(1,269 to 3,424) |  | 95.34(91.18 to 99.67) |  | -0.01(-0.24 to 0.22) |
| Hungary |  | 26,055(18,024 to 36,491) |  | 1038.53(1025.76 to 1051.43) |  | 19,971(13,788 to 27,903) |  | 929.55(916.18 to 943.08) |  | -0.19(-0.29 to -0.09) |  | 2,429(1,351 to 3,764) |  | 96.95(93.07 to 100.96) |  | 1,853(1,050 to 2,872) |  | 86.46(82.41 to 90.68) |  | -0.19(-0.30 to -0.08) |
| North Macedonia |  | 5,680(3,930 to 8,084) |  | 1110.62(1081.92 to 1139.90) |  | 5,122(3,524 to 7,347) |  | 937.68(911.69 to 964.32) |  | -0.50(-0.61 to -0.39) |  | 529(311 to 833) |  | 103.34(94.72 to 112.55) |  | 475(275 to 745) |  | 87.04(79.25 to 95.51) |  | -0.49(-0.60 to -0.39) |
| Montenegro |  | 1,681(1,190 to 2,382) |  | 1069.71(1019.14 to 1122.21) |  | 1,430(966 to 2,013) |  | 985.73(934.63 to 1039.10) |  | -0.21(-0.25 to -0.18) |  | 157(90 to 245) |  | 99.67(84.67 to 116.65) |  | 133(78 to 208) |  | 91.49(76.39 to 108.94) |  | -0.22(-0.25 to -0.18) |
| Poland |  | 94,523(66,004 to 134,663) |  | 993.23(986.82 to 999.68) |  | 82,292(56,790 to 114,991) |  | 901.72(895.33 to 908.15) |  | -0.33(-0.53 to -0.14) |  | 8,756(5,101 to 13,696) |  | 92.08(90.13 to 94.06) |  | 7,617(4,510 to 11,836) |  | 83.69(81.74 to 85.67) |  | -0.32(-0.52 to -0.12) |
| Romania |  | 54,422(38,000 to 77,937) |  | 981.20(972.90 to 989.56) |  | 38,869(27,184 to 54,484) |  | 963.61(953.69 to 973.61) |  | 0.11(-0.00 to 0.21) |  | 5,046(2,904 to 7,878) |  | 91.00(88.48 to 93.57) |  | 3,590(2,049 to 5,674) |  | 89.17(86.17 to 92.25) |  | 0.11(0.01 to 0.22) |
| Serbia |  | 23,144(16,212 to 32,309) |  | 984.79(972.11 to 997.60) |  | 17,640(12,157 to 25,076) |  | 860.70(847.84 to 873.73) |  | -0.55(-0.78 to -0.32) |  | 2,157(1,251 to 3,344) |  | 91.83(87.98 to 95.80) |  | 1,631(956 to 2,568) |  | 79.78(75.89 to 83.82) |  | -0.55(-0.77 to -0.32) |
| Slovakia |  | 13,980(9,779 to 19,923) |  | 1052.28(1034.83 to 1069.95) |  | 11,793(8,253 to 16,054) |  | 915.89(898.81 to 933.25) |  | -0.24(-0.41 to -0.08) |  | 1,295(766 to 1,988) |  | 97.56(92.29 to 103.05) |  | 1,093(648 to 1,754) |  | 85.13(79.97 to 90.58) |  | -0.24(-0.40 to -0.08) |
| Slovenia |  | 6,122(4,265 to 8,501) |  | 1204.94(1174.88 to 1235.62) |  | 5,048(3,391 to 7,153) |  | 1183.32(1149.60 to 1217.91) |  | 0.18(0.07 to 0.29) |  | 571(332 to 896) |  | 112.32(103.27 to 122.00) |  | 466(264 to 739) |  | 109.60(99.49 to 120.59) |  | 0.17(0.06 to 0.28) |
| Belarus |  | 42,497(29,016 to 59,602) |  | 1627.30(1611.79 to 1642.94) |  | 34,881(23,573 to 50,268) |  | 1589.42(1572.03 to 1606.98) |  | 0.28(0.10 to 0.46) |  | 3,930(2,170 to 6,296) |  | 150.46(145.77 to 155.28) |  | 3,217(1,830 to 5,023) |  | 147.05(141.79 to 152.49) |  | 0.29(0.11 to 0.47) |
| Estonia |  | 6,535(4,447 to 9,253) |  | 1679.93(1639.21 to 1721.47) |  | 4,334(3,036 to 6,045) |  | 1514.79(1468.56 to 1562.27) |  | 0.11(-0.05 to 0.26) |  | 604(349 to 976) |  | 155.58(143.35 to 168.63) |  | 401(233 to 640) |  | 140.38(126.54 to 155.51) |  | 0.12(-0.03 to 0.28) |
| Latvia |  | 9,936(6,921 to 13,760) |  | 1498.78(1469.31 to 1528.74) |  | 5,773(3,889 to 8,097) |  | 1448.21(1409.59 to 1487.74) |  | 0.04(-0.26 to 0.35) |  | 920(518 to 1,435) |  | 138.87(129.99 to 148.23) |  | 534(309 to 845) |  | 134.48(122.87 to 147.02) |  | 0.05(-0.25 to 0.35) |
| Lithuania |  | 17,592(11,980 to 24,423) |  | 1869.41(1841.83 to 1897.32) |  | 10,155(7,050 to 14,335) |  | 1708.11(1674.45 to 1742.37) |  | -0.10(-0.37 to 0.17) |  | 1,625(970 to 2,565) |  | 172.78(164.46 to 181.43) |  | 936(544 to 1,450) |  | 157.78(147.65 to 168.51) |  | -0.09(-0.36 to 0.18) |
| Republic of Moldova |  | 20,427(13,859 to 29,101) |  | 1773.24(1748.80 to 1797.95) |  | 13,253(9,150 to 18,472) |  | 1440.47(1415.01 to 1466.35) |  | -0.38(-0.54 to -0.21) |  | 1,891(1,060 to 3,036) |  | 164.13(156.75 to 171.78) |  | 1,224(696 to 1,932) |  | 133.41(125.73 to 141.52) |  | -0.36(-0.52 to -0.20) |
| Russian Federation |  | 649,786(443,584 to 906,167) |  | 1693.08(1688.92 to 1697.24) |  | 593,269(406,498 to 829,919) |  | 1700.93(1696.38 to 1705.48) |  | 0.45(0.26 to 0.64) |  | 60,151(34,229 to 93,769) |  | 156.78(155.52 to 158.06) |  | 54,632(31,338 to 85,890) |  | 157.15(155.76 to 158.54) |  | 0.45(0.26 to 0.64) |
| Ukraine |  | 223,205(153,891 to 313,122) |  | 1723.92(1716.74 to 1731.12) |  | 167,193(114,592 to 230,082) |  | 1591.77(1583.76 to 1599.82) |  | 0.04(-0.12 to 0.20) |  | 20,608(12,082 to 32,253) |  | 159.32(157.14 to 161.53) |  | 15,403(8,961 to 24,566) |  | 147.16(144.73 to 149.63) |  | 0.04(-0.12 to 0.20) |
| Brunei Darussalam |  | 1,208(797 to 1,692) |  | 1818.28(1713.35 to 1929.06) |  | 1,581(1,065 to 2,211) |  | 1228.32(1168.24 to 1290.95) |  | -1.28(-1.40 to -1.17) |  | 113(64 to 182) |  | 168.99(138.13 to 205.99) |  | 147(83 to 225) |  | 114.03(96.25 to 134.46) |  | -1.29(-1.41 to -1.18) |
| Japan |  | 468,572(311,668 to 658,447) |  | 1444.17(1439.96 to 1448.39) |  | 328,746(238,589 to 425,093) |  | 1244.07(1239.63 to 1248.53) |  | -0.68(-0.84 to -0.51) |  | 43,458(24,644 to 67,830) |  | 134.13(132.85 to 135.43) |  | 30,460(18,753 to 45,893) |  | 115.57(114.22 to 116.94) |  | -0.67(-0.83 to -0.51) |
| Republic of Korea |  | 175,213(118,008 to 243,621) |  | 1403.02(1396.38 to 1409.68) |  | 145,541(100,569 to 198,690) |  | 1178.97(1172.70 to 1185.27) |  | -0.55(-0.62 to -0.48) |  | 16,330(9,120 to 26,154) |  | 130.57(128.55 to 132.61) |  | 13,485(8,032 to 20,730) |  | 109.41(107.51 to 111.35) |  | -0.54(-0.61 to -0.47) |
| Singapore |  | 13,021(8,526 to 18,655) |  | 1363.91(1340.43 to 1387.72) |  | 16,894(11,512 to 23,734) |  | 1074.16(1056.73 to 1091.88) |  | -0.76(-0.84 to -0.68) |  | 1,210(699 to 1,902) |  | 126.75(119.66 to 134.17) |  | 1,578(895 to 2,485) |  | 100.52(95.23 to 106.11) |  | -0.74(-0.83 to -0.66) |
| Australia |  | 49,967(33,938 to 69,890) |  | 1110.76(1101.03 to 1120.57) |  | 60,723(40,984 to 86,220) |  | 993.08(985.09 to 1001.12) |  | -0.24(-0.31 to -0.17) |  | 4,575(2,555 to 7,223) |  | 101.73(98.79 to 104.73) |  | 5,574(3,135 to 8,993) |  | 91.24(88.83 to 93.70) |  | -0.23(-0.30 to -0.17) |
| New Zealand |  | 14,971(9,969 to 21,283) |  | 1644.65(1618.39 to 1671.25) |  | 15,631(11,662 to 19,957) |  | 1300.68(1280.23 to 1321.40) |  | -0.42(-0.67 to -0.16) |  | 1,359(775 to 2,189) |  | 149.36(141.52 to 157.54) |  | 1,433(887 to 2,128) |  | 119.39(113.24 to 125.80) |  | -0.39(-0.64 to -0.14) |
| Andorra |  | 125(85 to 181) |  | 812.28(674.77 to 972.27) |  | 154(110 to 221) |  | 765.80(643.26 to 908.27) |  | -0.21(-0.24 to -0.18) |  | 12(7 to 19) |  | 75.09(37.91 to 137.15) |  | 14(8 to 23) |  | 70.40(37.09 to 126.10) |  | -0.21(-0.24 to -0.18) |
| Austria |  | 22,471(17,713 to 27,463) |  | 1114.82(1100.21 to 1129.60) |  | 24,242(17,987 to 31,428) |  | 1210.19(1194.71 to 1225.85) |  | 0.37(0.21 to 0.54) |  | 2,081(1,276 to 3,076) |  | 103.33(98.91 to 107.91) |  | 2,232(1,332 to 3,411) |  | 111.61(106.94 to 116.46) |  | 0.37(0.20 to 0.54) |
| Belgium |  | 29,043(19,513 to 40,885) |  | 1169.24(1155.77 to 1182.84) |  | 27,371(18,784 to 37,871) |  | 1088.29(1075.25 to 1101.46) |  | 0.06(-0.31 to 0.43) |  | 2,682(1,502 to 4,332) |  | 108.03(103.96 to 112.23) |  | 2,521(1,376 to 4,062) |  | 100.40(96.47 to 104.47) |  | 0.04(-0.33 to 0.40) |
| Cyprus |  | 2,011(1,367 to 2,843) |  | 1007.46(963.86 to 1052.59) |  | 2,976(2,073 to 4,233) |  | 805.81(775.79 to 836.93) |  | -0.66(-0.76 to -0.56) |  | 186(103 to 297) |  | 93.24(80.31 to 107.73) |  | 275(154 to 428) |  | 74.61(65.66 to 84.70) |  | -0.66(-0.76 to -0.56) |
| Denmark |  | 9,515(6,568 to 13,330) |  | 723.00(708.45 to 737.80) |  | 8,710(6,102 to 12,228) |  | 682.86(668.45 to 697.52) |  | -0.25(-0.38 to -0.12) |  | 879(492 to 1,439) |  | 66.89(62.51 to 71.51) |  | 803(457 to 1,292) |  | 63.00(58.68 to 67.59) |  | -0.25(-0.38 to -0.11) |
| Finland |  | 14,394(9,864 to 20,344) |  | 1115.57(1097.12 to 1134.29) |  | 11,207(7,660 to 15,887) |  | 972.85(954.70 to 991.27) |  | -0.95(-1.23 to -0.67) |  | 1,328(730 to 2,119) |  | 102.98(97.42 to 108.80) |  | 1,035(567 to 1,667) |  | 89.95(84.49 to 95.70) |  | -0.95(-1.22 to -0.67) |
| France |  | 128,025(88,474 to 185,782) |  | 875.55(870.74 to 880.38) |  | 119,643(83,100 to 167,402) |  | 842.59(837.77 to 847.44) |  | -0.05(-0.12 to 0.03) |  | 11,816(6,320 to 19,088) |  | 80.81(79.36 to 82.29) |  | 11,048(6,130 to 17,846) |  | 77.91(76.45 to 79.40) |  | -0.05(-0.13 to 0.02) |
| Germany |  | 211,606(146,100 to 302,359) |  | 1053.41(1048.88 to 1057.96) |  | 178,387(124,763 to 250,169) |  | 1025.25(1020.41 to 1030.11) |  | 0.04(-0.24 to 0.31) |  | 19,480(11,007 to 31,535) |  | 97.05(95.68 to 98.44) |  | 16,358(9,345 to 26,279) |  | 94.13(92.67 to 95.62) |  | 0.03(-0.25 to 0.31) |
| Greece |  | 21,388(14,652 to 30,604) |  | 845.49(834.18 to 856.92) |  | 18,056(12,430 to 25,342) |  | 829.45(816.88 to 842.18) |  | 0.02(-0.02 to 0.07) |  | 1,977(1,114 to 3,155) |  | 78.19(74.78 to 81.72) |  | 1,659(929 to 2,725) |  | 76.38(72.59 to 80.33) |  | 0.02(-0.03 to 0.06) |
| Iceland |  | 268(197 to 349) |  | 411.28(363.41 to 463.97) |  | 439(303 to 620) |  | 544.76(494.70 to 598.86) |  | 1.24(0.95 to 1.53) |  | 25(15 to 38) |  | 38.03(24.54 to 56.64) |  | 41(22 to 64) |  | 50.39(36.02 to 69.02) |  | 1.24(0.95 to 1.53) |
| Ireland |  | 8,215(5,597 to 11,493) |  | 942.22(921.93 to 962.86) |  | 9,792(6,760 to 13,791) |  | 840.45(823.45 to 857.74) |  | -0.27(-0.34 to -0.20) |  | 764(437 to 1,223) |  | 87.63(81.52 to 94.09) |  | 899(501 to 1,440) |  | 77.23(72.14 to 82.62) |  | -0.30(-0.37 to -0.23) |
| Israel |  | 13,241(8,918 to 18,652) |  | 1093.56(1074.92 to 1112.47) |  | 25,196(17,484 to 34,871) |  | 1136.90(1122.87 to 1151.06) |  | -0.02(-0.10 to 0.06) |  | 1,226(663 to 1,976) |  | 101.19(95.58 to 107.07) |  | 2,326(1,317 to 3,689) |  | 105.03(100.80 to 109.40) |  | -0.02(-0.10 to 0.05) |
| Italy |  | 131,728(86,251 to 186,707) |  | 912.75(907.82 to 917.70) |  | 102,593(80,656 to 125,884) |  | 858.34(852.90 to 863.82) |  | -0.10(-0.17 to -0.02) |  | 12,093(6,728 to 18,983) |  | 83.82(82.33 to 85.33) |  | 9,428(5,976 to 13,635) |  | 79.07(77.42 to 80.75) |  | -0.07(-0.15 to 0.00) |
| Luxembourg |  | 968(660 to 1,362) |  | 964.19(903.73 to 1028.23) |  | 1,406(963 to 1,986) |  | 880.09(833.54 to 928.96) |  | -0.87(-1.08 to -0.65) |  | 89(49 to 145) |  | 89.04(71.34 to 110.48) |  | 130(68 to 209) |  | 81.26(67.54 to 97.40) |  | -0.86(-1.07 to -0.65) |
| Malta |  | 720(500 to 1,042) |  | 754.91(700.04 to 813.21) |  | 626(440 to 887) |  | 649.74(598.42 to 704.97) |  | -0.32(-0.42 to -0.22) |  | 67(37 to 103) |  | 69.85(53.89 to 89.41) |  | 58(32 to 94) |  | 59.90(45.06 to 78.92) |  | -0.33(-0.42 to -0.23) |
| Netherlands |  | 36,681(25,237 to 51,969) |  | 910.61(901.28 to 920.02) |  | 30,751(21,209 to 43,673) |  | 830.91(821.56 to 840.34) |  | -0.21(-0.27 to -0.16) |  | 3,398(1,885 to 5,509) |  | 84.40(81.57 to 87.30) |  | 2,830(1,627 to 4,571) |  | 76.54(73.72 to 79.45) |  | -0.22(-0.28 to -0.17) |
| Norway |  | 8,023(5,022 to 11,910) |  | 748.94(732.60 to 765.56) |  | 8,818(5,873 to 12,629) |  | 713.61(698.65 to 728.84) |  | -0.09(-0.18 to -0.00) |  | 740(393 to 1,209) |  | 69.08(64.18 to 74.27) |  | 811(458 to 1,281) |  | 65.70(61.22 to 70.46) |  | -0.09(-0.18 to 0.00) |
| Portugal |  | 16,128(11,091 to 22,468) |  | 640.07(630.23 to 650.04) |  | 13,330(9,343 to 18,248) |  | 573.21(563.14 to 583.44) |  | -0.33(-0.36 to -0.29) |  | 1,487(848 to 2,435) |  | 59.01(56.05 to 62.10) |  | 1,221(684 to 1,912) |  | 52.62(49.60 to 55.80) |  | -0.32(-0.36 to -0.29) |
| Spain |  | 66,134(45,911 to 91,779) |  | 686.82(681.59 to 692.08) |  | 65,466(49,200 to 83,369) |  | 652.74(647.48 to 658.03) |  | -0.12(-0.16 to -0.08) |  | 6,139(3,434 to 9,657) |  | 63.75(62.17 to 65.37) |  | 6,016(3,524 to 9,087) |  | 60.11(58.52 to 61.73) |  | -0.14(-0.19 to -0.09) |
| Sweden |  | 16,737(11,788 to 23,421) |  | 794.00(781.87 to 806.28) |  | 20,935(14,205 to 29,749) |  | 934.01(921.22 to 946.94) |  | 0.60(0.42 to 0.79) |  | 1,549(877 to 2,449) |  | 73.57(69.91 to 77.39) |  | 1,928(1,119 to 3,024) |  | 86.10(82.25 to 90.11) |  | 0.59(0.41 to 0.78) |
| Switzerland |  | 19,805(13,322 to 27,806) |  | 1097.89(1082.51 to 1113.47) |  | 20,585(13,921 to 29,197) |  | 1026.45(1012.10 to 1040.98) |  | -0.10(-0.21 to 0.02) |  | 1,823(1,035 to 2,890) |  | 101.17(96.54 to 106.00) |  | 1,889(1,050 to 3,071) |  | 94.37(90.05 to 98.87) |  | -0.10(-0.21 to 0.02) |
| United Kingdom |  | 154,353(102,598 to 219,291) |  | 1062.83(1057.51 to 1068.17) |  | 146,927(105,269 to 196,313) |  | 960.28(955.32 to 965.27) |  | -0.26(-0.31 to -0.21) |  | 14,195(8,038 to 22,660) |  | 97.79(96.19 to 99.43) |  | 13,491(8,119 to 20,795) |  | 88.30(86.79 to 89.82) |  | -0.26(-0.31 to -0.21) |
| Argentina |  | 68,864(46,515 to 97,119) |  | 867.97(861.50 to 874.48) |  | 85,232(58,603 to 117,773) |  | 701.35(696.64 to 706.08) |  | -0.55(-0.63 to -0.48) |  | 6,340(3,598 to 10,004) |  | 79.90(77.94 to 81.89) |  | 7,852(4,494 to 12,435) |  | 64.63(63.21 to 66.08) |  | -0.55(-0.63 to -0.48) |
| Chile |  | 44,553(30,289 to 62,228) |  | 1260.15(1248.39 to 1272.00) |  | 55,742(42,518 to 68,315) |  | 1128.69(1119.29 to 1138.14) |  | -0.15(-0.54 to 0.25) |  | 4,102(2,323 to 6,437) |  | 115.86(112.32 to 119.50) |  | 5,079(3,083 to 7,480) |  | 102.91(100.09 to 105.80) |  | -0.15(-0.54 to 0.24) |
| Uruguay |  | 7,460(5,067 to 10,148) |  | 994.76(972.32 to 1017.60) |  | 7,420(5,121 to 10,321) |  | 866.65(846.95 to 886.71) |  | -0.47(-0.51 to -0.42) |  | 689(378 to 1,051) |  | 91.82(85.09 to 98.95) |  | 680(383 to 1,067) |  | 79.57(73.67 to 85.83) |  | -0.47(-0.52 to -0.43) |
| Canada |  | 63,369(43,809 to 88,539) |  | 821.83(815.40 to 828.30) |  | 60,716(42,908 to 84,798) |  | 695.74(690.15 to 701.38) |  | -1.46(-2.05 to -0.87) |  | 5,870(3,336 to 9,286) |  | 76.11(74.17 to 78.11) |  | 5,606(3,121 to 8,997) |  | 64.31(62.61 to 66.04) |  | -1.46(-2.05 to -0.87) |
| United States of America |  | 697,653(458,772 to 1,002,054) |  | 988.10(985.77 to 990.43) |  | 490,787(365,657 to 641,291) |  | 627.91(626.15 to 629.68) |  | -1.97(-2.19 to -1.75) |  | 64,146(35,456 to 108,198) |  | 90.85(90.15 to 91.56) |  | 44,739(27,623 to 68,286) |  | 57.28(56.75 to 57.82) |  | -1.97(-2.19 to -1.76) |
| Antigua and Barbuda |  | 153(102 to 221) |  | 942.35(797.29 to 1108.99) |  | 182(126 to 257) |  | 732.98(629.57 to 849.95) |  | -0.90(-0.94 to -0.85) |  | 14(8 to 22) |  | 87.06(47.39 to 150.50) |  | 17(9 to 27) |  | 67.71(39.10 to 111.06) |  | -0.89(-0.93 to -0.84) |
| Bahamas |  | 653(432 to 938) |  | 903.82(834.57 to 977.83) |  | 765(523 to 1,067) |  | 707.43(658.04 to 759.62) |  | -0.89(-0.95 to -0.84) |  | 60(34 to 97) |  | 83.45(63.40 to 108.49) |  | 71(41 to 110) |  | 65.24(50.87 to 82.52) |  | -0.89(-0.95 to -0.84) |
| Barbados |  | 604(411 to 860) |  | 874.10(805.26 to 947.68) |  | 524(362 to 720) |  | 721.00(659.85 to 786.69) |  | -0.58(-0.65 to -0.51) |  | 56(32 to 87) |  | 80.72(60.80 to 105.57) |  | 48(27 to 78) |  | 66.54(48.91 to 88.94) |  | -0.58(-0.66 to -0.51) |
| Belize |  | 535(362 to 757) |  | 1351.01(1233.18 to 1478.89) |  | 982(659 to 1,411) |  | 825.96(774.87 to 879.66) |  | -1.44(-1.53 to -1.36) |  | 49(28 to 77) |  | 124.55(90.73 to 169.17) |  | 91(53 to 143) |  | 76.11(61.18 to 93.72) |  | -1.44(-1.53 to -1.36) |
| Cuba |  | 29,396(19,372 to 41,500) |  | 960.99(949.92 to 972.16) |  | 21,220(14,475 to 30,245) |  | 826.66(815.37 to 838.09) |  | -0.28(-0.36 to -0.20) |  | 2,714(1,550 to 4,263) |  | 88.65(85.31 to 92.09) |  | 1,958(1,153 to 3,108) |  | 76.39(72.99 to 79.94) |  | -0.26(-0.34 to -0.19) |
| Dominica |  | 192(129 to 270) |  | 1174.49(1010.98 to 1358.66) |  | 125(86 to 179) |  | 763.44(635.44 to 910.09) |  | -1.41(-1.51 to -1.31) |  | 18(10 to 27) |  | 108.16(62.99 to 175.56) |  | 12(6 to 18) |  | 70.33(35.75 to 124.86) |  | -1.41(-1.51 to -1.31) |
| Dominican Republic |  | 23,054(15,721 to 32,345) |  | 1268.95(1252.04 to 1286.07) |  | 27,034(18,485 to 38,317) |  | 926.35(915.33 to 937.48) |  | -1.03(-1.10 to -0.97) |  | 2,128(1,215 to 3,370) |  | 116.81(111.73 to 122.10) |  | 2,487(1,395 to 3,914) |  | 85.18(81.86 to 88.60) |  | -1.04(-1.10 to -0.97) |
| Grenada |  | 199(136 to 288) |  | 1063.38(916.75 to 1229.68) |  | 205(137 to 291) |  | 787.98(683.43 to 904.70) |  | -0.88(-0.98 to -0.79) |  | 18(10 to 29) |  | 98.13(57.53 to 160.30) |  | 19(11 to 30) |  | 72.44(43.42 to 114.44) |  | -0.88(-0.97 to -0.79) |
| Guyana |  | 2,225(1,501 to 3,256) |  | 1126.31(1078.48 to 1176.01) |  | 1,720(1,154 to 2,450) |  | 836.60(797.27 to 877.45) |  | -0.86(-0.91 to -0.82) |  | 204(116 to 321) |  | 102.91(88.84 to 118.89) |  | 157(90 to 244) |  | 76.11(64.58 to 89.20) |  | -0.86(-0.90 to -0.82) |
| Haiti |  | 24,559(16,339 to 35,070) |  | 1656.18(1635.10 to 1677.49) |  | 32,568(21,983 to 46,059) |  | 926.05(915.98 to 936.21) |  | -1.88(-1.93 to -1.84) |  | 2,237(1,282 to 3,443) |  | 150.51(144.21 to 157.04) |  | 2,962(1,697 to 4,692) |  | 84.16(81.14 to 87.26) |  | -1.86(-1.91 to -1.82) |
| Jamaica |  | 6,120(4,031 to 8,670) |  | 1059.96(1032.64 to 1087.91) |  | 6,107(4,035 to 8,784) |  | 774.28(754.94 to 794.01) |  | -0.99(-1.03 to -0.95) |  | 568(324 to 876) |  | 98.20(90.02 to 107.02) |  | 567(319 to 902) |  | 71.86(66.06 to 78.07) |  | -0.98(-1.02 to -0.94) |
| Saint Lucia |  | 362(243 to 521) |  | 1105.04(990.44 to 1230.87) |  | 342(237 to 487) |  | 728.16(652.36 to 811.08) |  | -1.31(-1.39 to -1.22) |  | 33(19 to 52) |  | 101.68(69.20 to 146.23) |  | 31(18 to 50) |  | 67.18(45.63 to 96.31) |  | -1.30(-1.38 to -1.21) |
| Saint Vincent and the Grenadines |  | 262(175 to 374) |  | 1044.44(916.46 to 1187.95) |  | 222(152 to 312) |  | 792.24(691.18 to 904.33) |  | -0.85(-0.90 to -0.80) |  | 24(14 to 38) |  | 96.59(60.70 to 149.52) |  | 20(12 to 32) |  | 73.06(44.84 to 112.94) |  | -0.85(-0.90 to -0.80) |
| Suriname |  | 1,086(720 to 1,537) |  | 1142.17(1074.10 to 1213.67) |  | 1,335(906 to 1,851) |  | 913.31(864.90 to 963.79) |  | -0.71(-0.76 to -0.65) |  | 100(57 to 157) |  | 105.09(85.19 to 128.52) |  | 122(70 to 191) |  | 83.61(69.43 to 99.91) |  | -0.72(-0.77 to -0.66) |
| Trinidad and Tobago |  | 3,107(2,101 to 4,463) |  | 1012.94(977.28 to 1049.67) |  | 2,723(1,854 to 3,881) |  | 786.86(757.11 to 817.56) |  | -0.67(-0.77 to -0.58) |  | 288(162 to 455) |  | 93.95(83.32 to 105.67) |  | 251(141 to 386) |  | 72.57(63.73 to 82.38) |  | -0.68(-0.77 to -0.59) |
| Bolivia (Plurinational State of) |  | 23,278(15,769 to 32,259) |  | 1587.32(1566.66 to 1608.19) |  | 31,207(21,097 to 44,046) |  | 997.47(986.41 to 1008.63) |  | -1.34(-1.54 to -1.15) |  | 2,147(1,239 to 3,379) |  | 146.05(139.84 to 152.49) |  | 2,865(1,625 to 4,487) |  | 91.51(88.18 to 94.93) |  | -1.33(-1.52 to -1.14) |
| Ecuador |  | 29,491(20,055 to 41,862) |  | 1212.27(1198.14 to 1226.54) |  | 39,453(26,850 to 55,477) |  | 829.85(821.68 to 838.09) |  | -1.19(-1.26 to -1.12) |  | 2,729(1,530 to 4,237) |  | 112.00(107.74 to 116.41) |  | 3,645(2,089 to 5,645) |  | 76.63(74.16 to 79.16) |  | -1.18(-1.25 to -1.12) |
| Peru |  | 58,564(39,477 to 84,154) |  | 1116.54(1107.31 to 1125.83) |  | 76,760(52,946 to 110,431) |  | 781.55(776.03 to 787.11) |  | -1.07(-1.13 to -1.02) |  | 5,418(2,921 to 8,446) |  | 103.08(100.29 to 105.93) |  | 7,089(4,047 to 11,060) |  | 72.18(70.50 to 73.88) |  | -1.06(-1.11 to -1.00) |
| Colombia |  | 89,513(60,301 to 128,527) |  | 1043.24(1036.26 to 1050.27) |  | 96,946(66,250 to 137,476) |  | 725.36(720.80 to 729.95) |  | -1.17(-1.19 to -1.14) |  | 8,319(4,628 to 13,256) |  | 96.79(94.67 to 98.95) |  | 8,992(5,163 to 14,377) |  | 67.28(65.90 to 68.69) |  | -1.16(-1.19 to -1.14) |
| Costa Rica |  | 7,990(5,434 to 11,436) |  | 1030.76(1007.74 to 1054.24) |  | 9,288(6,378 to 13,238) |  | 700.84(686.62 to 715.30) |  | -1.18(-1.30 to -1.06) |  | 742(424 to 1,152) |  | 95.46(88.56 to 102.84) |  | 854(494 to 1,293) |  | 64.49(60.23 to 69.00) |  | -1.19(-1.31 to -1.07) |
| El Salvador |  | 13,558(9,268 to 19,559) |  | 1077.93(1059.37 to 1096.76) |  | 12,691(8,702 to 18,259) |  | 702.53(690.33 to 714.91) |  | -1.42(-1.61 to -1.24) |  | 1,253(726 to 1,998) |  | 99.43(93.86 to 105.29) |  | 1,175(651 to 1,848) |  | 64.99(61.32 to 68.84) |  | -1.40(-1.58 to -1.22) |
| Guatemala |  | 29,041(19,547 to 41,465) |  | 1672.98(1653.31 to 1692.85) |  | 36,074(24,521 to 50,875) |  | 829.08(820.46 to 837.78) |  | -2.31(-2.52 to -2.10) |  | 2,675(1,526 to 4,176) |  | 153.89(147.97 to 160.01) |  | 3,306(1,867 to 5,136) |  | 75.88(73.29 to 78.54) |  | -2.31(-2.52 to -2.10) |
| Honduras |  | 15,440(10,380 to 22,183) |  | 1546.10(1521.07 to 1571.49) |  | 22,694(15,418 to 32,325) |  | 804.48(793.94 to 815.13) |  | -2.16(-2.34 to -1.98) |  | 1,428(797 to 2,285) |  | 142.61(135.09 to 150.49) |  | 2,094(1,183 to 3,311) |  | 74.10(70.93 to 77.39) |  | -2.15(-2.33 to -1.96) |
| Mexico |  | 256,207(173,668 to 363,569) |  | 1230.28(1225.39 to 1235.18) |  | 291,624(200,280 to 411,098) |  | 826.34(823.34 to 829.35) |  | -1.23(-1.34 to -1.11) |  | 23,749(13,495 to 36,798) |  | 113.74(112.26 to 115.24) |  | 26,869(15,405 to 41,534) |  | 76.17(75.26 to 77.09) |  | -1.24(-1.35 to -1.12) |
| Nicaragua |  | 11,274(7,589 to 15,739) |  | 1315.52(1290.31 to 1341.18) |  | 13,767(9,376 to 19,866) |  | 755.25(742.66 to 768.01) |  | -1.56(-1.78 to -1.33) |  | 1,041(580 to 1,603) |  | 121.15(113.60 to 129.16) |  | 1,269(700 to 1,971) |  | 69.60(65.82 to 73.55) |  | -1.55(-1.77 to -1.33) |
| Panama |  | 5,647(3,853 to 8,023) |  | 939.61(914.83 to 964.95) |  | 8,127(5,521 to 11,514) |  | 755.57(739.22 to 772.20) |  | -0.62(-0.68 to -0.55) |  | 525(305 to 819) |  | 87.26(79.83 to 95.26) |  | 751(423 to 1,175) |  | 69.83(64.92 to 75.02) |  | -0.63(-0.70 to -0.56) |
| Venezuela (Bolivarian Republic of) |  | 53,369(36,910 to 77,572) |  | 1124.34(1114.61 to 1134.16) |  | 52,810(36,605 to 75,720) |  | 772.90(766.23 to 779.61) |  | -1.02(-1.13 to -0.91) |  | 4,958(2,822 to 7,629) |  | 104.28(101.33 to 107.30) |  | 4,888(2,816 to 7,572) |  | 71.68(69.66 to 73.75) |  | -1.02(-1.14 to -0.91) |
| Brazil |  | 440,439(282,973 to 649,166) |  | 1153.13(1149.68 to 1156.59) |  | 570,125(392,217 to 800,518) |  | 950.86(948.38 to 953.35) |  | -1.08(-1.35 to -0.81) |  | 40,456(22,736 to 65,619) |  | 105.76(104.72 to 106.81) |  | 52,465(30,332 to 80,564) |  | 87.56(86.81 to 88.31) |  | -1.05(-1.32 to -0.78) |
| Paraguay |  | 14,164(9,729 to 19,890) |  | 1554.22(1528.21 to 1580.59) |  | 16,143(10,713 to 22,909) |  | 851.62(838.50 to 864.91) |  | -1.85(-1.92 to -1.78) |  | 1,306(728 to 2,057) |  | 142.98(135.18 to 151.16) |  | 1,489(840 to 2,356) |  | 78.48(74.53 to 82.59) |  | -1.84(-1.91 to -1.77) |
| Algeria |  | 100,346(67,884 to 138,903) |  | 1795.47(1783.90 to 1807.11) |  | 150,399(100,262 to 213,721) |  | 1305.62(1298.98 to 1312.27) |  | -0.79(-0.93 to -0.66) |  | 9,232(5,179 to 14,219) |  | 164.53(161.04 to 168.08) |  | 13,758(7,862 to 21,259) |  | 119.56(117.55 to 121.59) |  | -0.80(-0.93 to -0.66) |
| Bahrain |  | 1,775(1,175 to 2,549) |  | 1477.04(1405.02 to 1552.65) |  | 3,535(2,443 to 4,982) |  | 1061.37(1026.60 to 1097.08) |  | -1.24(-1.34 to -1.14) |  | 163(91 to 266) |  | 135.45(114.31 to 160.36) |  | 323(185 to 506) |  | 96.97(86.66 to 108.24) |  | -1.24(-1.34 to -1.14) |
| Egypt |  | 232,872(155,612 to 328,444) |  | 1824.07(1816.59 to 1831.59) |  | 365,059(249,092 to 516,479) |  | 1415.88(1411.28 to 1420.50) |  | -0.67(-0.74 to -0.60) |  | 21,369(12,649 to 33,853) |  | 167.06(164.80 to 169.34) |  | 33,534(18,723 to 52,842) |  | 129.95(128.56 to 131.35) |  | -0.66(-0.73 to -0.59) |
| Iran (Islamic Republic of) |  | 183,652(126,240 to 257,711) |  | 1517.07(1509.90 to 1524.27) |  | 268,328(182,433 to 375,996) |  | 1110.71(1106.40 to 1115.03) |  | -0.91(-1.23 to -0.60) |  | 16,851(9,550 to 26,089) |  | 138.76(136.61 to 140.95) |  | 24,448(13,943 to 38,165) |  | 101.43(100.13 to 102.74) |  | -0.91(-1.22 to -0.60) |
| Iraq |  | 100,617(66,736 to 143,301) |  | 2624.50(2607.76 to 2641.34) |  | 144,426(98,077 to 206,660) |  | 1392.62(1385.42 to 1399.86) |  | -2.31(-2.40 to -2.21) |  | 9,213(5,180 to 14,524) |  | 239.58(234.54 to 244.71) |  | 13,176(7,447 to 21,196) |  | 126.88(124.71 to 129.07) |  | -2.31(-2.40 to -2.21) |
| Jordan |  | 17,705(11,893 to 25,555) |  | 2273.22(2238.08 to 2308.85) |  | 47,141(31,521 to 67,523) |  | 1561.07(1546.95 to 1575.29) |  | -1.28(-1.38 to -1.17) |  | 1,628(923 to 2,535) |  | 208.07(197.56 to 219.08) |  | 4,312(2,446 to 6,664) |  | 142.61(138.37 to 146.95) |  | -1.28(-1.38 to -1.18) |
| Kuwait |  | 5,725(3,794 to 8,383) |  | 1334.74(1298.99 to 1371.44) |  | 16,873(11,670 to 23,635) |  | 1084.45(1067.30 to 1101.87) |  | -1.05(-1.22 to -0.88) |  | 530(303 to 853) |  | 123.29(112.61 to 134.95) |  | 1,546(882 to 2,446) |  | 99.69(94.52 to 105.13) |  | -1.06(-1.23 to -0.89) |
| Lebanon |  | 14,270(9,615 to 20,490) |  | 1920.07(1888.41 to 1952.15) |  | 22,118(14,899 to 31,416) |  | 1428.85(1409.76 to 1448.16) |  | -0.93(-0.98 to -0.88) |  | 1,305(733 to 2,080) |  | 175.39(165.92 to 185.29) |  | 2,015(1,135 to 3,176) |  | 130.27(124.54 to 136.21) |  | -0.92(-0.97 to -0.87) |
| Libya |  | 18,905(12,636 to 27,183) |  | 2249.14(2215.59 to 2283.13) |  | 22,072(15,202 to 30,824) |  | 1096.56(1082.08 to 1111.20) |  | -2.07(-2.35 to -1.78) |  | 1,736(1,002 to 2,794) |  | 205.75(195.70 to 216.24) |  | 2,019(1,168 to 3,158) |  | 100.45(96.10 to 104.96) |  | -2.07(-2.35 to -1.79) |
| Morocco |  | 102,500(70,170 to 148,031) |  | 1667.05(1656.64 to 1677.52) |  | 110,453(76,314 to 159,780) |  | 1132.76(1126.08 to 1139.46) |  | -1.33(-1.39 to -1.27) |  | 9,416(5,351 to 15,095) |  | 152.66(149.53 to 155.85) |  | 10,074(5,851 to 15,432) |  | 103.37(101.36 to 105.41) |  | -1.33(-1.39 to -1.27) |
| Palestine |  | 11,790(7,848 to 16,860) |  | 2841.43(2788.00 to 2895.72) |  | 18,927(12,680 to 27,400) |  | 1485.66(1464.22 to 1507.36) |  | -2.44(-2.62 to -2.25) |  | 1,083(613 to 1,707) |  | 260.13(244.16 to 276.97) |  | 1,738(954 to 2,763) |  | 136.06(129.63 to 142.75) |  | -2.43(-2.61 to -2.25) |
| Oman |  | 7,976(5,352 to 11,228) |  | 2423.13(2368.89 to 2478.45) |  | 12,592(8,702 to 18,228) |  | 1193.39(1172.26 to 1214.84) |  | -2.19(-2.43 to -1.94) |  | 733(405 to 1,172) |  | 222.05(205.87 to 239.33) |  | 1,156(662 to 1,823) |  | 109.57(103.23 to 116.22) |  | -2.17(-2.41 to -1.93) |
| Qatar |  | 1,626(1,085 to 2,351) |  | 2010.07(1910.15 to 2114.95) |  | 7,541(5,154 to 10,922) |  | 1267.68(1237.38 to 1298.69) |  | -1.59(-1.64 to -1.55) |  | 149(83 to 235) |  | 184.15(154.83 to 218.67) |  | 690(393 to 1,087) |  | 116.28(107.21 to 126.06) |  | -1.59(-1.63 to -1.55) |
| Saudi Arabia |  | 64,247(43,107 to 93,343) |  | 2052.33(2035.94 to 2068.84) |  | 112,371(76,968 to 156,382) |  | 1056.24(1050.00 to 1062.52) |  | -2.30(-2.43 to -2.16) |  | 5,906(3,279 to 9,334) |  | 187.93(183.00 to 192.98) |  | 10,251(5,998 to 15,586) |  | 96.46(94.57 to 98.37) |  | -2.29(-2.42 to -2.15) |
| Syrian Arab Republic |  | 63,301(42,830 to 89,442) |  | 2446.47(2426.62 to 2466.47) |  | 45,460(31,104 to 63,893) |  | 1269.94(1257.54 to 1282.45) |  | -2.13(-2.23 to -2.04) |  | 5,802(3,364 to 9,147) |  | 223.42(217.46 to 229.52) |  | 4,144(2,439 to 6,459) |  | 115.78(112.06 to 119.62) |  | -2.13(-2.22 to -2.04) |
| Tunisia |  | 32,114(21,793 to 46,165) |  | 1599.18(1581.29 to 1617.23) |  | 37,512(25,831 to 52,774) |  | 1192.02(1179.84 to 1204.30) |  | -0.90(-0.99 to -0.82) |  | 2,964(1,731 to 4,511) |  | 147.22(141.84 to 152.78) |  | 3,428(1,957 to 5,348) |  | 109.18(105.51 to 112.95) |  | -0.91(-0.99 to -0.82) |
| Türkiye |  | 213,832(139,726 to 308,698) |  | 1538.43(1531.82 to 1545.07) |  | 261,095(178,196 to 367,167) |  | 1183.77(1179.21 to 1188.34) |  | -1.03(-1.11 to -0.95) |  | 19,706(11,253 to 30,921) |  | 141.42(139.42 to 143.44) |  | 23,907(13,555 to 37,615) |  | 108.54(107.16 to 109.93) |  | -1.03(-1.11 to -0.95) |
| United Arab Emirates |  | 5,893(3,899 to 8,489) |  | 1652.65(1608.09 to 1698.49) |  | 17,864(12,296 to 24,970) |  | 1014.73(997.69 to 1032.03) |  | -1.51(-1.67 to -1.35) |  | 542(309 to 850) |  | 151.60(138.34 to 166.18) |  | 1,626(969 to 2,591) |  | 92.80(87.69 to 98.18) |  | -1.51(-1.67 to -1.35) |
| Yemen |  | 97,550(64,766 to 138,886) |  | 3738.58(3714.44 to 3762.86) |  | 135,318(90,705 to 191,033) |  | 1685.44(1676.35 to 1694.57) |  | -2.86(-3.06 to -2.65) |  | 8,790(4,988 to 13,814) |  | 335.92(328.72 to 343.26) |  | 12,236(7,052 to 18,607) |  | 152.09(149.37 to 154.85) |  | -2.84(-3.04 to -2.63) |
| Afghanistan |  | 49,400(33,416 to 67,635) |  | 2548.82(2525.04 to 2572.81) |  | 134,067(89,454 to 189,185) |  | 1993.14(1982.01 to 2004.31) |  | -0.80(-1.01 to -0.58) |  | 4,481(2,570 to 6,959) |  | 230.49(223.39 to 237.81) |  | 12,222(6,832 to 18,882) |  | 180.97(177.64 to 184.36) |  | -0.78(-0.99 to -0.57) |
| Bangladesh |  | 426,737(286,451 to 615,345) |  | 1847.26(1841.49 to 1853.05) |  | 481,240(325,152 to 698,323) |  | 1044.84(1041.89 to 1047.81) |  | -1.71(-1.83 to -1.58) |  | 39,286(22,176 to 61,885) |  | 169.40(167.66 to 171.16) |  | 44,155(24,750 to 68,461) |  | 95.78(94.88 to 96.67) |  | -1.69(-1.81 to -1.57) |
| Bhutan |  | 2,415(1,624 to 3,455) |  | 1822.06(1746.95 to 1900.00) |  | 2,066(1,403 to 2,950) |  | 985.47(943.27 to 1029.19) |  | -2.04(-2.11 to -1.96) |  | 222(127 to 353) |  | 167.16(145.00 to 192.23) |  | 191(107 to 297) |  | 91.02(78.52 to 105.07) |  | -2.01(-2.09 to -1.94) |
| India |  | 3,585,604(2,422,800 to 5,010,458) |  | 1826.53(1824.62 to 1828.44) |  | 4,117,137(2,868,780 to 5,736,851) |  | 1087.22(1086.17 to 1088.28) |  | -1.75(-1.79 to -1.71) |  | 326,605(186,178 to 502,371) |  | 166.09(165.51 to 166.67) |  | 377,501(217,018 to 582,795) |  | 99.63(99.31 to 99.95) |  | -1.72(-1.76 to -1.69) |
| Nepal |  | 100,203(67,112 to 141,509) |  | 2286.72(2272.33 to 2301.19) |  | 93,834(63,297 to 134,137) |  | 1037.01(1030.34 to 1043.72) |  | -2.64(-2.77 to -2.52) |  | 9,142(5,255 to 14,286) |  | 208.25(203.93 to 212.66) |  | 8,628(4,959 to 13,633) |  | 95.21(93.20 to 97.26) |  | -2.61(-2.73 to -2.50) |
| Pakistan |  | 653,093(445,212 to 907,483) |  | 2960.54(2953.24 to 2967.86) |  | 943,700(633,305 to 1,325,555) |  | 1575.70(1572.50 to 1578.91) |  | -2.08(-2.18 to -1.98) |  | 59,849(34,982 to 92,866) |  | 270.69(268.49 to 272.91) |  | 86,540(49,967 to 134,842) |  | 144.29(143.33 to 145.27) |  | -2.07(-2.17 to -1.97) |
| Angola |  | 48,105(32,233 to 67,550) |  | 2186.05(2166.04 to 2206.21) |  | 101,314(69,187 to 145,758) |  | 1388.90(1380.21 to 1397.63) |  | -1.38(-1.48 to -1.28) |  | 4,396(2,516 to 6,719) |  | 199.35(193.35 to 205.51) |  | 9,257(5,370 to 14,369) |  | 126.63(124.01 to 129.28) |  | -1.36(-1.46 to -1.26) |
| Central African Republic |  | 10,577(7,097 to 15,185) |  | 1701.01(1667.84 to 1734.74) |  | 15,981(10,991 to 22,582) |  | 1210.63(1191.61 to 1229.89) |  | -1.07(-1.16 to -0.98) |  | 958(563 to 1,511) |  | 153.71(143.86 to 164.12) |  | 1,454(855 to 2,309) |  | 109.87(104.20 to 115.79) |  | -1.03(-1.12 to -0.94) |
| Congo |  | 8,231(5,672 to 11,572) |  | 1547.02(1512.52 to 1582.21) |  | 14,635(10,119 to 20,581) |  | 1045.67(1028.74 to 1062.81) |  | -0.98(-1.09 to -0.87) |  | 750(422 to 1,160) |  | 140.56(130.31 to 151.50) |  | 1,337(757 to 2,045) |  | 95.45(90.39 to 100.74) |  | -0.96(-1.07 to -0.84) |
| Democratic Republic of the Congo |  | 167,035(113,794 to 236,665) |  | 2052.28(2042.16 to 2062.44) |  | 253,435(174,743 to 357,836) |  | 1250.56(1245.60 to 1255.55) |  | -1.51(-1.68 to -1.33) |  | 15,091(8,533 to 24,148) |  | 184.93(181.90 to 188.00) |  | 23,171(12,972 to 36,013) |  | 114.08(112.59 to 115.60) |  | -1.46(-1.63 to -1.30) |
| Equatorial Guinea |  | 2,060(1,377 to 2,906) |  | 2192.40(2096.86 to 2291.58) |  | 3,696(2,517 to 5,308) |  | 1041.68(1007.73 to 1076.61) |  | -2.59(-2.67 to -2.51) |  | 187(103 to 288) |  | 198.40(170.43 to 230.09) |  | 338(196 to 532) |  | 95.03(84.99 to 106.09) |  | -2.55(-2.64 to -2.47) |
| Gabon |  | 3,270(2,237 to 4,644) |  | 1545.56(1490.46 to 1602.54) |  | 4,376(2,982 to 6,230) |  | 916.43(889.16 to 944.36) |  | -1.63(-1.69 to -1.56) |  | 299(169 to 458) |  | 140.62(124.38 to 158.79) |  | 398(227 to 614) |  | 83.24(75.18 to 91.99) |  | -1.61(-1.68 to -1.54) |
| Burundi |  | 22,442(14,932 to 31,658) |  | 1839.14(1814.34 to 1864.26) |  | 40,179(26,881 to 57,056) |  | 1359.62(1345.91 to 1373.45) |  | -0.91(-0.95 to -0.87) |  | 2,061(1,156 to 3,283) |  | 168.52(161.07 to 176.26) |  | 3,699(2,013 to 5,855) |  | 124.86(120.74 to 129.12) |  | -0.89(-0.93 to -0.84) |
| Comoros |  | 1,636(1,123 to 2,373) |  | 1655.10(1573.54 to 1740.20) |  | 1,652(1,134 to 2,368) |  | 858.72(817.65 to 901.39) |  | -2.07(-2.10 to -2.03) |  | 151(87 to 239) |  | 152.76(128.73 to 180.44) |  | 153(88 to 244) |  | 79.37(67.24 to 93.13) |  | -2.05(-2.08 to -2.01) |
| Djibouti |  | 1,724(1,156 to 2,409) |  | 1865.41(1775.29 to 1959.52) |  | 3,293(2,232 to 4,712) |  | 1012.08(977.69 to 1047.43) |  | -1.97(-2.07 to -1.87) |  | 159(91 to 254) |  | 171.94(145.39 to 202.62) |  | 305(174 to 489) |  | 93.60(83.35 to 104.83) |  | -1.95(-2.05 to -1.85) |
| Eritrea |  | 12,218(8,330 to 17,131) |  | 1628.87(1599.42 to 1658.77) |  | 15,273(10,132 to 22,109) |  | 945.25(930.15 to 960.54) |  | -1.77(-1.86 to -1.68) |  | 1,120(634 to 1,785) |  | 149.07(140.26 to 158.34) |  | 1,413(813 to 2,228) |  | 87.35(82.81 to 92.10) |  | -1.72(-1.81 to -1.64) |
| Ethiopia |  | 194,066(133,949 to 272,464) |  | 1800.15(1791.88 to 1808.44) |  | 273,591(185,831 to 385,219) |  | 1038.42(1034.42 to 1042.43) |  | -1.80(-1.86 to -1.73) |  | 17,824(10,157 to 27,878) |  | 164.86(162.37 to 167.39) |  | 25,241(14,538 to 38,910) |  | 95.57(94.36 to 96.79) |  | -1.76(-1.83 to -1.70) |
| Kenya |  | 81,990(56,578 to 115,104) |  | 1711.58(1699.23 to 1724.00) |  | 117,743(81,348 to 165,979) |  | 925.39(920.00 to 930.80) |  | -1.93(-1.96 to -1.91) |  | 7,561(4,298 to 11,746) |  | 157.27(153.55 to 161.07) |  | 10,863(6,123 to 16,973) |  | 85.18(83.55 to 86.83) |  | -1.91(-1.93 to -1.88) |
| Madagascar |  | 42,044(27,700 to 60,264) |  | 1647.49(1631.21 to 1663.91) |  | 67,555(44,708 to 96,052) |  | 977.63(970.12 to 985.18) |  | -1.71(-1.77 to -1.65) |  | 3,863(2,115 to 6,075) |  | 151.06(146.16 to 156.10) |  | 6,251(3,557 to 10,139) |  | 90.23(87.96 to 92.55) |  | -1.67(-1.73 to -1.61) |
| Malawi |  | 37,265(25,150 to 53,918) |  | 1728.57(1710.33 to 1746.98) |  | 43,450(29,055 to 62,706) |  | 930.17(921.15 to 939.27) |  | -2.20(-2.35 to -2.06) |  | 3,383(1,931 to 5,470) |  | 156.51(151.07 to 162.14) |  | 4,004(2,252 to 6,232) |  | 85.43(82.72 to 88.23) |  | -2.13(-2.27 to -1.99) |
| Mauritius |  | 3,549(2,498 to 4,934) |  | 1176.05(1137.17 to 1216.06) |  | 3,291(2,345 to 4,453) |  | 1013.22(978.66 to 1048.76) |  | -0.52(-0.57 to -0.47) |  | 331(192 to 515) |  | 109.63(97.98 to 122.42) |  | 304(177 to 471) |  | 93.79(83.48 to 105.10) |  | -0.53(-0.59 to -0.48) |
| Mozambique |  | 48,079(32,018 to 69,387) |  | 1606.29(1591.76 to 1620.94) |  | 81,380(54,325 to 115,805) |  | 1141.64(1133.54 to 1149.79) |  | -0.97(-1.07 to -0.86) |  | 4,374(2,487 to 6,967) |  | 145.85(141.50 to 150.32) |  | 7,335(4,059 to 11,575) |  | 102.57(100.16 to 105.04) |  | -0.97(-1.07 to -0.86) |
| Rwanda |  | 26,403(17,683 to 37,926) |  | 1712.26(1690.85 to 1733.91) |  | 32,618(22,149 to 46,477) |  | 957.35(946.86 to 967.94) |  | -2.09(-2.18 to -1.99) |  | 2,432(1,357 to 3,834) |  | 157.29(150.85 to 163.96) |  | 3,021(1,708 to 4,782) |  | 88.51(85.34 to 91.78) |  | -2.05(-2.14 to -1.96) |
| Seychelles |  | 229(156 to 315) |  | 1302.99(1133.62 to 1493.67) |  | 299(211 to 416) |  | 1207.53(1073.42 to 1354.54) |  | -0.07(-0.17 to 0.03) |  | 21(12 to 34) |  | 121.23(73.85 to 191.86) |  | 28(16 to 45) |  | 112.53(74.39 to 164.32) |  | -0.07(-0.16 to 0.03) |
| Somalia |  | 31,888(21,083 to 45,123) |  | 1982.07(1960.12 to 2004.23) |  | 72,678(48,116 to 100,906) |  | 1619.59(1607.56 to 1631.70) |  | -0.58(-0.64 to -0.53) |  | 2,901(1,678 to 4,575) |  | 180.16(173.59 to 186.94) |  | 6,657(3,709 to 10,302) |  | 148.03(144.41 to 151.72) |  | -0.55(-0.62 to -0.49) |
| United Republic of Tanzania |  | 100,822(66,591 to 143,844) |  | 1789.77(1778.25 to 1801.35) |  | 177,752(118,802 to 251,564) |  | 1255.04(1249.10 to 1261.01) |  | -1.00(-1.07 to -0.92) |  | 9,068(5,123 to 14,363) |  | 160.61(157.18 to 164.11) |  | 16,329(9,140 to 25,664) |  | 115.02(113.22 to 116.83) |  | -0.92(-1.01 to -0.84) |
| Uganda |  | 68,841(45,796 to 99,717) |  | 1912.96(1897.88 to 1928.14) |  | 113,221(75,231 to 162,772) |  | 1158.97(1151.97 to 1166.01) |  | -1.75(-1.88 to -1.63) |  | 6,271(3,518 to 10,055) |  | 173.71(169.20 to 178.33) |  | 10,409(5,859 to 16,453) |  | 106.23(104.12 to 108.37) |  | -1.71(-1.83 to -1.59) |
| Zambia |  | 29,141(19,784 to 41,673) |  | 1724.54(1703.74 to 1745.55) |  | 48,198(32,256 to 69,042) |  | 1026.80(1017.37 to 1036.31) |  | -1.65(-1.76 to -1.54) |  | 2,660(1,515 to 4,133) |  | 157.14(150.91 to 163.59) |  | 4,428(2,564 to 7,194) |  | 94.12(91.28 to 97.04) |  | -1.61(-1.72 to -1.51) |
| Botswana |  | 4,231(2,793 to 6,137) |  | 1385.63(1342.58 to 1429.90) |  | 7,088(4,908 to 10,115) |  | 1027.08(1003.22 to 1051.38) |  | -0.92(-0.94 to -0.89) |  | 389(219 to 604) |  | 127.07(114.29 to 141.10) |  | 645(383 to 1,000) |  | 93.39(86.30 to 100.94) |  | -0.93(-0.97 to -0.89) |
| Lesotho |  | 5,824(3,852 to 8,455) |  | 1602.46(1561.23 to 1644.56) |  | 5,430(3,743 to 7,679) |  | 1093.59(1064.19 to 1123.69) |  | -1.26(-1.35 to -1.17) |  | 534(302 to 841) |  | 146.69(134.41 to 159.84) |  | 493(281 to 788) |  | 98.90(90.21 to 108.29) |  | -1.31(-1.40 to -1.22) |
| Namibia |  | 5,051(3,399 to 7,147) |  | 1574.78(1530.05 to 1620.64) |  | 7,498(5,004 to 10,509) |  | 1143.12(1117.20 to 1169.54) |  | -0.95(-1.02 to -0.88) |  | 465(268 to 750) |  | 144.65(131.33 to 159.12) |  | 686(382 to 1,102) |  | 104.47(96.74 to 112.69) |  | -0.94(-1.02 to -0.87) |
| South Africa |  | 129,288(89,774 to 180,017) |  | 1378.27(1370.61 to 1385.98) |  | 174,720(118,998 to 244,085) |  | 1103.65(1098.46 to 1108.86) |  | -0.73(-0.74 to -0.72) |  | 11,846(6,887 to 18,148) |  | 125.95(123.64 to 128.29) |  | 15,811(9,058 to 24,613) |  | 99.89(98.34 to 101.47) |  | -0.76(-0.78 to -0.74) |
| Eswatini |  | 2,884(1,968 to 4,043) |  | 1563.61(1504.89 to 1624.32) |  | 3,146(2,126 to 4,578) |  | 1002.47(967.16 to 1038.91) |  | -1.40(-1.43 to -1.38) |  | 267(154 to 418) |  | 144.25(126.84 to 163.71) |  | 286(160 to 450) |  | 90.81(80.42 to 102.35) |  | -1.47(-1.50 to -1.43) |
| Zimbabwe |  | 39,971(27,045 to 56,838) |  | 1783.16(1764.94 to 1801.55) |  | 51,777(34,372 to 73,087) |  | 1318.23(1306.76 to 1329.80) |  | -0.67(-0.80 to -0.53) |  | 3,661(2,093 to 5,677) |  | 162.89(157.43 to 168.53) |  | 4,745(2,717 to 7,680) |  | 120.46(117.01 to 123.99) |  | -0.66(-0.80 to -0.52) |
| Benin |  | 20,798(13,949 to 28,950) |  | 1944.15(1916.84 to 1971.81) |  | 42,346(28,584 to 58,722) |  | 1365.32(1352.01 to 1378.75) |  | -0.88(-0.99 to -0.77) |  | 1,899(1,089 to 2,988) |  | 176.96(168.80 to 185.46) |  | 3,888(2,273 to 6,088) |  | 125.03(121.03 to 129.15) |  | -0.86(-0.96 to -0.75) |
| Burkina Faso |  | 42,215(28,536 to 61,563) |  | 2116.02(2095.47 to 2136.74) |  | 75,870(50,637 to 107,369) |  | 1448.60(1438.02 to 1459.25) |  | -1.29(-1.32 to -1.26) |  | 3,849(2,169 to 6,161) |  | 192.55(186.39 to 198.88) |  | 6,988(4,005 to 10,738) |  | 133.03(129.84 to 136.28) |  | -1.25(-1.29 to -1.22) |
| Cameroon |  | 39,191(26,621 to 56,366) |  | 1715.06(1697.57 to 1732.71) |  | 83,035(55,555 to 116,504) |  | 1098.15(1090.52 to 1105.83) |  | -1.24(-1.41 to -1.06) |  | 3,578(2,020 to 5,600) |  | 156.15(150.92 to 161.55) |  | 7,647(4,438 to 11,887) |  | 100.88(98.58 to 103.23) |  | -1.20(-1.37 to -1.03) |
| Cabo Verde |  | 970(653 to 1,355) |  | 1271.76(1188.27 to 1360.49) |  | 1,256(863 to 1,730) |  | 824.20(778.98 to 871.53) |  | -1.50(-1.55 to -1.45) |  | 90(50 to 144) |  | 117.51(93.17 to 147.39) |  | 116(67 to 180) |  | 75.99(62.71 to 91.45) |  | -1.50(-1.56 to -1.45) |
| Chad |  | 28,432(19,169 to 40,391) |  | 2256.72(2229.95 to 2283.76) |  | 74,573(48,955 to 102,921) |  | 2090.52(2075.03 to 2106.11) |  | -0.19(-0.24 to -0.15) |  | 2,599(1,422 to 4,201) |  | 205.75(197.73 to 214.04) |  | 6,821(3,699 to 10,444) |  | 190.59(185.94 to 195.35) |  | -0.18(-0.23 to -0.14) |
| Côte d'Ivoire |  | 49,864(33,227 to 69,984) |  | 1887.99(1870.65 to 1905.48) |  | 81,597(54,906 to 114,663) |  | 1252.20(1243.47 to 1260.98) |  | -1.19(-1.24 to -1.15) |  | 4,540(2,573 to 7,235) |  | 171.25(166.06 to 176.58) |  | 7,502(4,246 to 11,523) |  | 114.93(112.30 to 117.61) |  | -1.15(-1.20 to -1.11) |
| Gambia |  | 4,718(3,156 to 6,781) |  | 2152.03(2087.52 to 2218.42) |  | 6,829(4,639 to 9,740) |  | 1156.72(1128.62 to 1185.44) |  | -2.08(-2.17 to -1.99) |  | 433(245 to 685) |  | 196.59(177.47 to 217.64) |  | 625(357 to 1,005) |  | 105.59(97.24 to 114.59) |  | -2.07(-2.16 to -1.98) |
| Ghana |  | 51,961(35,219 to 75,159) |  | 1515.57(1502.19 to 1529.07) |  | 80,902(56,110 to 113,501) |  | 890.15(883.96 to 896.37) |  | -1.67(-1.70 to -1.63) |  | 4,770(2,759 to 7,482) |  | 138.74(134.71 to 142.87) |  | 7,434(4,216 to 11,379) |  | 81.67(79.80 to 83.57) |  | -1.65(-1.68 to -1.61) |
| Guinea |  | 27,333(18,396 to 39,247) |  | 2043.18(2018.71 to 2067.91) |  | 43,348(28,763 to 60,699) |  | 1354.54(1341.50 to 1367.69) |  | -1.32(-1.36 to -1.29) |  | 2,500(1,410 to 3,939) |  | 186.56(179.22 to 194.15) |  | 3,985(2,259 to 6,423) |  | 124.17(120.25 to 128.20) |  | -1.31(-1.34 to -1.27) |
| Guinea-Bissau |  | 3,712(2,500 to 5,193) |  | 1667.46(1612.81 to 1723.72) |  | 5,338(3,630 to 7,909) |  | 1041.91(1013.52 to 1071.00) |  | -1.52(-1.56 to -1.48) |  | 339(192 to 533) |  | 151.94(135.78 to 169.74) |  | 491(281 to 765) |  | 95.64(87.18 to 104.81) |  | -1.50(-1.54 to -1.46) |
| Liberia |  | 10,123(6,786 to 14,457) |  | 1877.87(1839.88 to 1916.57) |  | 13,233(8,934 to 18,981) |  | 984.54(967.66 to 1001.65) |  | -2.09(-2.17 to -2.01) |  | 909(507 to 1,432) |  | 168.15(156.93 to 180.08) |  | 1,198(672 to 1,862) |  | 88.96(83.95 to 94.22) |  | -2.06(-2.14 to -1.99) |
| Mali |  | 39,664(26,754 to 54,935) |  | 2140.71(2119.27 to 2162.33) |  | 86,302(57,576 to 120,839) |  | 1693.84(1682.15 to 1705.60) |  | -0.76(-0.80 to -0.73) |  | 3,634(2,071 to 5,699) |  | 195.70(189.26 to 202.32) |  | 7,910(4,417 to 12,335) |  | 154.70(151.18 to 158.28) |  | -0.74(-0.78 to -0.71) |
| Mauritania |  | 8,273(5,544 to 11,639) |  | 1834.17(1793.79 to 1875.35) |  | 11,380(7,694 to 16,194) |  | 1110.96(1090.25 to 1131.99) |  | -1.55(-1.62 to -1.47) |  | 759(437 to 1,202) |  | 167.95(155.90 to 180.80) |  | 1,054(604 to 1,635) |  | 102.65(96.44 to 109.20) |  | -1.52(-1.60 to -1.44) |
| Niger |  | 36,911(24,833 to 52,026) |  | 2243.96(2220.44 to 2267.69) |  | 119,710(77,365 to 166,346) |  | 2500.23(2485.47 to 2515.06) |  | 0.39(0.33 to 0.44) |  | 3,376(1,898 to 5,369) |  | 204.71(197.66 to 211.98) |  | 11,053(6,108 to 17,273) |  | 229.88(225.43 to 234.41) |  | 0.42(0.36 to 0.47) |
| Nigeria |  | 377,698(258,748 to 528,387) |  | 1990.64(1984.01 to 1997.29) |  | 755,577(513,311 to 1,047,460) |  | 1399.95(1396.71 to 1403.19) |  | -1.09(-1.17 to -1.01) |  | 34,453(19,610 to 53,613) |  | 180.97(178.98 to 182.99) |  | 69,360(39,490 to 108,163) |  | 128.16(127.19 to 129.15) |  | -1.06(-1.14 to -0.97) |
| Sao Tome and Principe |  | 363(241 to 514) |  | 1515.13(1357.39 to 1688.22) |  | 473(326 to 665) |  | 874.50(796.82 to 958.20) |  | -1.85(-1.91 to -1.79) |  | 34(19 to 52) |  | 139.98(95.18 to 201.21) |  | 44(25 to 69) |  | 80.72(58.41 to 109.33) |  | -1.85(-1.90 to -1.79) |
| Senegal |  | 31,548(21,279 to 43,730) |  | 1939.80(1917.74 to 1962.08) |  | 43,954(29,979 to 63,907) |  | 1164.42(1153.37 to 1175.56) |  | -1.42(-1.53 to -1.32) |  | 2,888(1,642 to 4,594) |  | 177.04(170.43 to 183.87) |  | 4,037(2,254 to 6,443) |  | 106.77(103.45 to 110.19) |  | -1.40(-1.50 to -1.29) |
| Sierra Leone |  | 17,804(11,869 to 25,038) |  | 1791.86(1764.60 to 1819.51) |  | 25,999(17,901 to 37,684) |  | 1182.17(1167.39 to 1197.12) |  | -1.38(-1.51 to -1.25) |  | 1,636(872 to 2,518) |  | 164.13(155.97 to 172.68) |  | 2,390(1,325 to 3,684) |  | 108.34(103.91 to 112.95) |  | -1.37(-1.50 to -1.24) |
| Togo |  | 14,973(10,172 to 21,830) |  | 1830.60(1800.45 to 1861.19) |  | 23,364(16,160 to 32,797) |  | 1102.61(1088.44 to 1116.94) |  | -1.46(-1.57 to -1.36) |  | 1,375(781 to 2,178) |  | 167.63(158.61 to 177.10) |  | 2,152(1,206 to 3,324) |  | 101.42(97.16 to 105.84) |  | -1.44(-1.54 to -1.35) |
| American Samoa |  | 264(178 to 364) |  | 2272.43(1998.27 to 2578.30) |  | 178(122 to 246) |  | 1567.71(1343.94 to 1819.43) |  | -1.21(-1.23 to -1.18) |  | 25(14 to 39) |  | 210.95(133.83 to 322.49) |  | 16(10 to 26) |  | 144.73(82.92 to 236.40) |  | -1.22(-1.25 to -1.19) |
| Bermuda |  | 154(103 to 216) |  | 859.98(728.21 to 1012.14) |  | 103(70 to 146) |  | 739.08(597.88 to 907.74) |  | -0.47(-0.54 to -0.40) |  | 14(8 to 23) |  | 80.05(43.79 to 139.08) |  | 10(5 to 15) |  | 68.69(31.08 to 137.40) |  | -0.47(-0.54 to -0.39) |
| Cook Islands |  | 79(55 to 111) |  | 1806.76(1426.26 to 2265.04) |  | 60(42 to 82) |  | 1388.68(1057.58 to 1794.18) |  | -0.82(-0.86 to -0.79) |  | 7(4 to 12) |  | 167.50(68.23 to 353.55) |  | 6(3 to 9) |  | 128.78(44.58 to 295.27) |  | -0.82(-0.86 to -0.79) |
| Greenland |  | 140(96 to 197) |  | 925.60(775.13 to 1100.09) |  | 103(70 to 143) |  | 787.55(642.16 to 957.86) |  | -0.58(-0.65 to -0.52) |  | 13(7 to 21) |  | 84.62(43.98 to 151.93) |  | 9(5 to 15) |  | 72.22(33.62 to 138.14) |  | -0.58(-0.64 to -0.52) |
| Guam |  | 612(424 to 857) |  | 1750.80(1612.42 to 1899.53) |  | 579(396 to 798) |  | 1591.21(1463.68 to 1727.13) |  | -0.33(-0.47 to -0.20) |  | 57(32 to 91) |  | 162.90(122.73 to 214.06) |  | 54(31 to 85) |  | 147.61(110.64 to 193.28) |  | -0.33(-0.47 to -0.20) |
| Monaco |  | 62(43 to 89) |  | 870.02(660.39 to 1140.13) |  | 58(41 to 83) |  | 813.99(612.78 to 1065.94) |  | -0.21(-0.23 to -0.19) |  | 6(3 to 9) |  | 80.47(27.34 to 207.12) |  | 5(3 to 8) |  | 74.72(24.32 to 183.12) |  | -0.22(-0.24 to -0.20) |
| Nauru |  | 67(45 to 93) |  | 2872.18(2217.43 to 3680.61) |  | 50(33 to 70) |  | 1811.27(1340.52 to 2404.78) |  | -1.52(-1.57 to -1.47) |  | 6(4 to 10) |  | 265.48(97.79 to 608.90) |  | 5(3 to 7) |  | 166.86(50.35 to 422.58) |  | -1.53(-1.58 to -1.48) |
| Niue |  | 9(7 to 13) |  | 1917.31(884.91 to 3645.07) |  | 5(4 to 8) |  | 1410.44(481.48 to 3249.97) |  | -0.96(-1.01 to -0.91) |  | 1(1 to 1) |  | 177.94(2.60 to 1155.91) |  | 1(0 to 1) |  | 130.48(0.12 to 1305.79) |  | -0.97(-1.02 to -0.92) |
| Northern Mariana Islands |  | 224(152 to 319) |  | 1604.32(1393.00 to 1845.17) |  | 153(107 to 208) |  | 1329.59(1120.13 to 1569.27) |  | -0.51(-0.55 to -0.46) |  | 21(12 to 33) |  | 148.78(89.88 to 240.83) |  | 14(8 to 23) |  | 123.39(66.04 to 213.55) |  | -0.50(-0.54 to -0.46) |
| Palau |  | 64(44 to 90) |  | 1585.71(1217.26 to 2040.04) |  | 50(35 to 67) |  | 1297.07(947.42 to 1745.19) |  | -0.60(-0.64 to -0.57) |  | 6(3 to 9) |  | 147.22(52.97 to 338.70) |  | 5(3 to 7) |  | 119.74(33.81 to 319.42) |  | -0.61(-0.64 to -0.58) |
| Puerto Rico |  | 9,127(6,165 to 12,763) |  | 956.98(937.45 to 976.83) |  | 5,282(3,669 to 7,423) |  | 691.35(672.63 to 710.49) |  | -1.07(-1.13 to -1.01) |  | 843(484 to 1,387) |  | 88.43(82.56 to 94.61) |  | 488(270 to 772) |  | 63.98(58.37 to 70.02) |  | -1.06(-1.13 to -0.99) |
| Saint Kitts and Nevis |  | 92(60 to 133) |  | 949.33(756.29 to 1187.77) |  | 115(79 to 165) |  | 721.21(594.83 to 868.47) |  | -0.85(-0.87 to -0.82) |  | 8(5 to 13) |  | 87.28(36.88 to 191.70) |  | 11(6 to 17) |  | 66.52(32.56 to 123.45) |  | -0.83(-0.86 to -0.80) |
| San Marino |  | 53(36 to 76) |  | 844.76(632.72 to 1107.42) |  | 56(40 to 80) |  | 795.24(594.85 to 1046.30) |  | -0.20(-0.22 to -0.17) |  | 5(3 to 8) |  | 78.45(25.17 to 187.90) |  | 5(3 to 8) |  | 73.20(23.16 to 180.80) |  | -0.21(-0.24 to -0.18) |
| Tokelau |  | 10(7 to 14) |  | 3035.85(1454.36 to 5734.36) |  | 6(4 to 9) |  | 2001.05(760.49 to 4316.30) |  | -1.29(-1.34 to -1.25) |  | 1(1 to 1) |  | 280.84(5.49 to 1901.08) |  | 1(0 to 1) |  | 184.60(0.48 to 1605.18) |  | -1.30(-1.35 to -1.25) |
| Tuvalu |  | 48(34 to 68) |  | 1983.18(1463.51 to 2633.82) |  | 42(29 to 57) |  | 1476.29(1060.69 to 2006.62) |  | -0.91(-0.94 to -0.88) |  | 4(3 to 7) |  | 183.77(54.90 to 458.60) |  | 4(2 to 6) |  | 136.81(35.88 to 366.46) |  | -0.87(-0.91 to -0.83) |
| United States Virgin Islands |  | 296(204 to 410) |  | 1039.88(924.02 to 1166.77) |  | 139(95 to 197) |  | 795.87(666.49 to 944.97) |  | -0.87(-0.92 to -0.82) |  | 27(16 to 41) |  | 96.22(63.40 to 140.67) |  | 13(7 to 20) |  | 73.50(38.31 to 130.29) |  | -0.87(-0.92 to -0.83) |
| South Sudan |  | 19,128(12,861 to 27,112) |  | 1561.10(1538.04 to 1584.48) |  | 29,204(19,142 to 40,723) |  | 1367.87(1351.99 to 1383.90) |  | -0.36(-0.41 to -0.31) |  | 1,750(979 to 2,751) |  | 142.28(135.39 to 149.49) |  | 2,672(1,494 to 4,137) |  | 125.02(120.25 to 129.93) |  | -0.35(-0.41 to -0.29) |
| Sudan |  | 131,900(85,805 to 185,887) |  | 2951.79(2935.45 to 2968.21) |  | 175,527(118,429 to 245,934) |  | 1598.62(1591.04 to 1606.22) |  | -2.26(-2.44 to -2.07) |  | 12,035(6,756 to 19,040) |  | 268.47(263.56 to 273.45) |  | 16,033(9,175 to 25,179) |  | 145.66(143.38 to 147.96) |  | -2.24(-2.43 to -2.06) |

**Table S3:** Prevalence and DALYs cases and age-standardised rate of polycystic ovarian syndrome for WCBA in 1990 and 2021, and their average annual percentage change from 1990 to 2021 by countries and territories.

| **Location** |  | **Prevalence** | | | | | | | | |  | **DALYs (Disability-Adjusted Life Years)** | | | | | | | | |
| --- | --- | --- | --- | --- | --- | --- | --- | --- | --- | --- | --- | --- | --- | --- | --- | --- | --- | --- | --- | --- |
|  |  | **Number of cases, 1990** |  | **Age-standardised** rate, 1990 |  | **Number of cases, 2021** |  | **Age-standardised** rate, 2021 |  | AAPC, 1990–2021 |  | **Number of cases, 1990** |  | **Age-standardised** rate, 1990 |  | **Number of cases, 2021** |  | **Age-standardised** rate, 2021 |  | AAPC, 1990–2021 |
| China |  | 5,127,459(3,585,816 to 7,237,269) |  | 1600.46(1599.05 to 1601.87) |  | 9,481,520(6,677,110 to 13,483,459) |  | 2959.21(2957.25 to 2961.18) |  | 2.07(1.91 to 2.24) |  | 44,078(18,967 to 91,450) |  | 13.70(13.57 to 13.84) |  | 81,338(35,559 to 168,663) |  | 25.57(25.39 to 25.75) |  | 2.09(1.92 to 2.26) |
| Democratic People's Republic of Korea |  | 94,970(66,984 to 137,221) |  | 1678.50(1667.75 to 1689.32) |  | 124,320(85,604 to 175,175) |  | 1882.44(1871.92 to 1893.01) |  | 0.32(0.23 to 0.41) |  | 824(342 to 1,738) |  | 14.52(13.54 to 15.56) |  | 1,072(458 to 2,217) |  | 16.28(15.31 to 17.29) |  | 0.33(0.25 to 0.42) |
| Cambodia |  | 55,630(38,353 to 79,708) |  | 2233.34(2214.37 to 2252.46) |  | 175,038(121,741 to 250,772) |  | 3852.40(3834.30 to 3870.56) |  | 1.88(1.85 to 1.92) |  | 485(213 to 964) |  | 19.34(17.62 to 21.21) |  | 1,522(678 to 3,118) |  | 33.48(31.82 to 35.22) |  | 1.94(1.89 to 1.99) |
| Indonesia |  | 1,377,477(975,187 to 1,933,703) |  | 2885.24(2880.32 to 2890.16) |  | 4,160,508(2,953,224 to 5,850,443) |  | 5510.68(5505.37 to 5515.99) |  | 2.37(2.28 to 2.46) |  | 12,331(5,372 to 25,213) |  | 25.63(25.17 to 26.10) |  | 36,682(16,344 to 75,309) |  | 48.69(48.20 to 49.20) |  | 2.32(2.24 to 2.40) |
| Lao People's Democratic Republic |  | 24,111(16,841 to 33,704) |  | 2516.51(2484.05 to 2549.33) |  | 92,988(65,716 to 133,535) |  | 4667.77(4637.66 to 4698.03) |  | 2.22(2.16 to 2.28) |  | 215(94 to 438) |  | 22.23(19.29 to 25.54) |  | 822(364 to 1,695) |  | 41.15(38.37 to 44.09) |  | 2.22(2.16 to 2.28) |
| Malaysia |  | 206,107(144,449 to 297,789) |  | 4597.16(4577.00 to 4617.39) |  | 668,843(466,905 to 958,652) |  | 7877.54(7858.62 to 7896.49) |  | 1.88(1.73 to 2.04) |  | 1,793(764 to 3,597) |  | 39.83(37.98 to 41.77) |  | 5,785(2,566 to 11,769) |  | 68.11(66.37 to 69.90) |  | 1.88(1.72 to 2.03) |
| Maldives |  | 1,284(884 to 1,796) |  | 2723.59(2567.25 to 2888.36) |  | 7,368(5,089 to 10,536) |  | 6307.02(6161.06 to 6455.88) |  | 3.39(3.10 to 3.67) |  | 12(5 to 24) |  | 24.14(11.67 to 46.62) |  | 65(29 to 141) |  | 55.95(42.96 to 72.03) |  | 3.39(3.11 to 3.68) |
| Myanmar |  | 241,142(166,889 to 345,394) |  | 2321.66(2312.15 to 2331.20) |  | 719,359(502,609 to 1,008,047) |  | 4766.35(4755.33 to 4777.38) |  | 2.76(2.62 to 2.90) |  | 2,146(935 to 4,501) |  | 20.50(19.62 to 21.42) |  | 6,367(2,807 to 13,208) |  | 42.16(41.13 to 43.21) |  | 2.77(2.63 to 2.91) |
| Philippines |  | 486,355(339,705 to 688,264) |  | 3159.86(3150.77 to 3168.98) |  | 1,573,841(1,099,718 to 2,239,764) |  | 5366.33(5357.92 to 5374.75) |  | 2.17(2.02 to 2.31) |  | 4,308(1,859 to 8,788) |  | 27.85(27.00 to 28.72) |  | 14,011(6,065 to 29,084) |  | 47.69(46.90 to 48.49) |  | 2.15(2.02 to 2.29) |
| Sri Lanka |  | 167,584(117,876 to 239,939) |  | 3642.38(3624.84 to 3659.98) |  | 341,198(240,311 to 478,607) |  | 6055.35(6034.97 to 6075.78) |  | 1.86(1.64 to 2.08) |  | 1,504(672 to 3,123) |  | 32.59(30.95 to 34.30) |  | 2,993(1,324 to 5,969) |  | 53.25(51.36 to 55.21) |  | 1.83(1.62 to 2.05) |
| Thailand |  | 551,221(383,818 to 787,443) |  | 3435.49(3426.33 to 3444.68) |  | 1,082,502(759,683 to 1,540,743) |  | 6659.42(6646.53 to 6672.34) |  | 2.24(2.08 to 2.40) |  | 5,041(2,211 to 10,313) |  | 31.30(30.44 to 32.19) |  | 9,580(4,274 to 19,995) |  | 59.34(58.12 to 60.57) |  | 2.20(2.05 to 2.35) |
| Timor-Leste |  | 4,457(3,104 to 6,218) |  | 2383.18(2312.23 to 2456.00) |  | 13,501(9,465 to 19,526) |  | 3938.39(3869.88 to 4007.91) |  | 1.96(1.85 to 2.07) |  | 40(18 to 81) |  | 21.10(14.94 to 29.30) |  | 121(53 to 247) |  | 34.96(28.81 to 42.17) |  | 1.97(1.86 to 2.08) |
| Viet Nam |  | 371,449(255,951 to 522,813) |  | 2181.30(2173.96 to 2188.66) |  | 1,124,756(783,395 to 1,602,433) |  | 4371.36(4363.17 to 4379.56) |  | 2.80(2.61 to 2.98) |  | 3,289(1,429 to 6,818) |  | 19.17(18.50 to 19.88) |  | 9,841(4,202 to 20,505) |  | 38.41(37.64 to 39.18) |  | 2.76(2.58 to 2.94) |
| Fiji |  | 6,013(4,137 to 8,699) |  | 3068.75(2990.78 to 3148.40) |  | 10,714(7,438 to 15,260) |  | 4688.94(4600.54 to 4778.62) |  | 1.24(1.09 to 1.38) |  | 53(23 to 108) |  | 27.12(20.27 to 35.77) |  | 94(42 to 193) |  | 41.37(33.45 to 50.62) |  | 1.24(1.09 to 1.38) |
| Kiribati |  | 492(335 to 698) |  | 2616.77(2385.59 to 2866.99) |  | 1,236(864 to 1,731) |  | 3855.72(3642.95 to 4078.48) |  | 1.12(0.90 to 1.34) |  | 4(2 to 9) |  | 22.86(6.41 to 63.17) |  | 11(5 to 22) |  | 33.73(16.69 to 62.07) |  | 1.12(0.90 to 1.34) |
| Marshall Islands |  | 218(150 to 313) |  | 2234.75(1933.69 to 2581.41) |  | 558(387 to 780) |  | 3772.05(3464.98 to 4099.80) |  | 1.47(1.29 to 1.65) |  | 2(1 to 4) |  | 19.59(1.89 to 105.16) |  | 5(2 to 10) |  | 32.97(10.49 to 79.20) |  | 1.46(1.28 to 1.64) |
| Micronesia (Federated States of) |  | 662(457 to 934) |  | 2867.47(2644.75 to 3107.22) |  | 1,080(756 to 1,538) |  | 4149.01(3903.46 to 4406.53) |  | 0.97(0.76 to 1.17) |  | 6(3 to 12) |  | 25.19(8.65 to 63.45) |  | 9(4 to 20) |  | 36.39(16.94 to 69.21) |  | 0.96(0.75 to 1.17) |
| Papua New Guinea |  | 20,456(14,006 to 28,720) |  | 2122.84(2093.08 to 2152.96) |  | 82,232(57,068 to 118,738) |  | 3128.83(3107.38 to 3150.41) |  | 1.00(0.83 to 1.17) |  | 178(80 to 377) |  | 18.38(15.71 to 21.43) |  | 714(308 to 1,494) |  | 27.12(25.16 to 29.21) |  | 1.01(0.83 to 1.19) |
| Samoa |  | 1,256(861 to 1,824) |  | 3434.84(3239.66 to 3639.98) |  | 2,247(1,556 to 3,191) |  | 4636.23(4444.56 to 4834.49) |  | 0.86(0.73 to 0.99) |  | 11(5 to 23) |  | 30.10(14.56 to 57.20) |  | 20(9 to 41) |  | 40.57(24.55 to 63.78) |  | 0.86(0.73 to 0.99) |
| Solomon Islands |  | 1,638(1,117 to 2,368) |  | 2220.19(2108.68 to 2336.80) |  | 5,974(4,151 to 8,299) |  | 3492.48(3403.94 to 3582.84) |  | 1.23(0.98 to 1.47) |  | 14(6 to 30) |  | 19.47(10.40 to 34.42) |  | 52(23 to 112) |  | 30.58(22.83 to 40.26) |  | 1.23(0.98 to 1.48) |
| Tonga |  | 863(593 to 1,258) |  | 3893.91(3630.59 to 4172.56) |  | 1,338(915 to 1,902) |  | 5318.19(5035.40 to 5613.12) |  | 0.69(0.48 to 0.91) |  | 8(3 to 16) |  | 34.12(13.88 to 72.09) |  | 12(5 to 24) |  | 46.50(23.70 to 82.64) |  | 0.69(0.48 to 0.90) |
| Vanuatu |  | 900(624 to 1,287) |  | 2566.13(2396.28 to 2746.53) |  | 3,008(2,043 to 4,412) |  | 3834.48(3697.56 to 3975.43) |  | 1.21(1.16 to 1.27) |  | 8(4 to 16) |  | 22.50(9.38 to 48.40) |  | 26(12 to 56) |  | 33.61(21.96 to 49.62) |  | 1.21(1.15 to 1.26) |
| Armenia |  | 5,385(3,670 to 7,898) |  | 614.06(597.37 to 631.17) |  | 6,787(4,589 to 9,859) |  | 912.99(890.65 to 935.81) |  | 1.43(1.38 to 1.49) |  | 47(19 to 100) |  | 5.34(3.89 to 7.24) |  | 59(25 to 124) |  | 7.91(5.96 to 10.39) |  | 1.41(1.36 to 1.47) |
| Azerbaijan |  | 11,830(7,987 to 17,586) |  | 625.79(614.03 to 637.76) |  | 26,694(17,952 to 38,849) |  | 965.52(953.75 to 977.42) |  | 1.73(1.61 to 1.85) |  | 106(43 to 232) |  | 5.55(4.50 to 6.82) |  | 234(96 to 511) |  | 8.53(7.45 to 9.74) |  | 1.74(1.61 to 1.86) |
| Georgia |  | 10,820(7,178 to 15,802) |  | 783.06(768.30 to 798.04) |  | 10,833(7,430 to 15,092) |  | 1379.37(1352.80 to 1406.38) |  | 2.31(2.06 to 2.56) |  | 95(38 to 205) |  | 6.89(5.57 to 8.44) |  | 94(41 to 196) |  | 12.10(9.72 to 14.95) |  | 2.30(2.05 to 2.55) |
| Kazakhstan |  | 29,144(19,707 to 42,970) |  | 703.53(695.38 to 711.77) |  | 47,129(31,933 to 66,861) |  | 992.21(983.16 to 1001.34) |  | 1.21(1.18 to 1.25) |  | 259(110 to 560) |  | 6.24(5.50 to 7.07) |  | 412(176 to 868) |  | 8.72(7.89 to 9.62) |  | 1.19(1.16 to 1.22) |
| Kyrgyzstan |  | 6,803(4,594 to 9,856) |  | 647.45(631.54 to 663.72) |  | 13,475(8,983 to 19,577) |  | 777.95(764.82 to 791.25) |  | 0.45(0.37 to 0.53) |  | 60(25 to 129) |  | 5.65(4.26 to 7.45) |  | 118(49 to 256) |  | 6.80(5.62 to 8.16) |  | 0.46(0.38 to 0.53) |
| Mongolia |  | 3,142(2,075 to 4,523) |  | 617.34(594.77 to 640.68) |  | 7,455(5,046 to 10,597) |  | 879.31(859.27 to 899.74) |  | 1.25(1.20 to 1.30) |  | 28(12 to 59) |  | 5.40(3.49 to 8.17) |  | 65(27 to 143) |  | 7.71(5.94 to 9.89) |  | 1.26(1.21 to 1.31) |
| Tajikistan |  | 6,197(4,213 to 9,230) |  | 516.73(503.00 to 530.82) |  | 17,041(11,274 to 24,728) |  | 665.58(655.55 to 675.74) |  | 0.90(0.83 to 0.96) |  | 55(23 to 124) |  | 4.56(3.37 to 6.17) |  | 152(62 to 332) |  | 5.94(5.03 to 6.98) |  | 0.93(0.86 to 1.00) |
| Turkmenistan |  | 5,795(3,932 to 8,472) |  | 654.65(636.96 to 672.81) |  | 11,993(8,107 to 17,568) |  | 953.55(936.55 to 970.79) |  | 1.26(1.23 to 1.28) |  | 51(21 to 110) |  | 5.74(4.20 to 7.79) |  | 105(44 to 224) |  | 8.33(6.81 to 10.09) |  | 1.25(1.23 to 1.28) |
| Uzbekistan |  | 32,884(21,976 to 47,832) |  | 671.08(663.37 to 678.88) |  | 84,081(56,778 to 117,757) |  | 938.68(932.31 to 945.09) |  | 1.15(1.02 to 1.28) |  | 290(120 to 628) |  | 5.87(5.17 to 6.66) |  | 733(311 to 1,605) |  | 8.20(7.62 to 8.82) |  | 1.12(0.99 to 1.25) |
| Albania |  | 2,312(1,497 to 3,531) |  | 276.79(265.33 to 288.68) |  | 2,242(1,495 to 3,389) |  | 364.43(349.41 to 379.97) |  | 0.86(0.76 to 0.95) |  | 20(8 to 44) |  | 2.43(1.47 to 3.88) |  | 20(8 to 44) |  | 3.23(1.96 to 5.07) |  | 0.87(0.77 to 0.98) |
| Bosnia and Herzegovina |  | 2,743(1,748 to 4,343) |  | 234.51(225.80 to 243.49) |  | 2,673(1,746 to 4,062) |  | 364.77(350.71 to 379.31) |  | 1.59(1.36 to 1.81) |  | 24(10 to 51) |  | 2.05(1.31 to 3.07) |  | 23(10 to 50) |  | 3.18(1.99 to 4.90) |  | 1.57(1.35 to 1.79) |
| Bulgaria |  | 6,742(4,295 to 10,169) |  | 325.35(317.55 to 333.29) |  | 5,926(3,936 to 8,823) |  | 417.23(406.11 to 428.61) |  | 0.82(0.78 to 0.86) |  | 59(24 to 126) |  | 2.84(2.15 to 3.68) |  | 51(21 to 106) |  | 3.62(2.65 to 4.87) |  | 0.83(0.79 to 0.87) |
| Croatia |  | 3,604(2,363 to 5,489) |  | 298.49(288.77 to 308.47) |  | 3,630(2,388 to 5,443) |  | 403.90(390.52 to 417.68) |  | 1.07(0.97 to 1.17) |  | 31(13 to 67) |  | 2.60(1.77 to 3.72) |  | 32(12 to 67) |  | 3.53(2.38 to 5.11) |  | 1.08(0.98 to 1.18) |
| Czechia |  | 7,927(5,051 to 11,828) |  | 308.40(301.56 to 315.37) |  | 9,042(5,977 to 13,427) |  | 396.05(387.44 to 404.82) |  | 0.78(0.73 to 0.84) |  | 69(28 to 145) |  | 2.68(2.07 to 3.41) |  | 78(33 to 166) |  | 3.44(2.68 to 4.37) |  | 0.76(0.70 to 0.82) |
| Hungary |  | 8,409(5,350 to 12,485) |  | 332.13(324.96 to 339.42) |  | 8,738(5,763 to 13,271) |  | 410.28(401.33 to 419.41) |  | 0.68(0.60 to 0.75) |  | 73(31 to 158) |  | 2.89(2.26 to 3.66) |  | 76(31 to 162) |  | 3.57(2.78 to 4.55) |  | 0.68(0.61 to 0.75) |
| North Macedonia |  | 1,370(881 to 2,132) |  | 268.59(254.55 to 283.21) |  | 1,996(1,296 to 3,004) |  | 373.65(356.99 to 390.99) |  | 1.16(1.09 to 1.24) |  | 12(5 to 26) |  | 2.36(1.22 to 4.13) |  | 17(7 to 36) |  | 3.25(1.87 to 5.41) |  | 1.16(1.08 to 1.23) |
| Montenegro |  | 503(326 to 758) |  | 321.13(293.64 to 350.56) |  | 594(391 to 879) |  | 412.15(379.14 to 447.46) |  | 1.03(0.94 to 1.11) |  | 4(2 to 9) |  | 2.79(0.82 to 7.02) |  | 5(2 to 11) |  | 3.60(1.17 to 8.76) |  | 1.04(0.95 to 1.12) |
| Poland |  | 46,199(30,690 to 69,111) |  | 487.05(482.56 to 491.58) |  | 45,296(32,290 to 61,237) |  | 513.10(508.14 to 518.09) |  | -0.01(-0.08 to 0.06) |  | 407(169 to 832) |  | 4.31(3.89 to 4.76) |  | 394(175 to 807) |  | 4.50(4.05 to 5.00) |  | -0.01(-0.08 to 0.05) |
| Romania |  | 15,630(10,005 to 23,504) |  | 278.08(273.71 to 282.51) |  | 16,025(10,599 to 24,176) |  | 396.30(389.95 to 402.73) |  | 1.24(1.19 to 1.29) |  | 137(55 to 286) |  | 2.43(2.04 to 2.88) |  | 139(58 to 294) |  | 3.46(2.89 to 4.12) |  | 1.24(1.19 to 1.30) |
| Serbia |  | 6,510(4,171 to 9,795) |  | 278.37(271.63 to 285.24) |  | 7,689(5,093 to 11,132) |  | 377.84(369.28 to 386.56) |  | 1.11(1.05 to 1.18) |  | 57(23 to 119) |  | 2.44(1.85 to 3.17) |  | 67(27 to 143) |  | 3.31(2.55 to 4.24) |  | 1.11(1.05 to 1.17) |
| Slovakia |  | 3,823(2,455 to 5,747) |  | 287.22(278.15 to 296.52) |  | 5,068(3,370 to 7,587) |  | 399.95(388.49 to 411.70) |  | 1.07(1.03 to 1.12) |  | 33(13 to 73) |  | 2.50(1.72 to 3.52) |  | 44(17 to 99) |  | 3.49(2.49 to 4.81) |  | 1.08(1.04 to 1.12) |
| Slovenia |  | 1,523(991 to 2,306) |  | 303.72(288.59 to 319.46) |  | 1,796(1,170 to 2,643) |  | 426.34(405.93 to 447.63) |  | 1.16(1.05 to 1.27) |  | 13(5 to 28) |  | 2.62(1.40 to 4.54) |  | 15(7 to 32) |  | 3.66(2.00 to 6.34) |  | 1.14(1.03 to 1.25) |
| Belarus |  | 9,777(6,608 to 14,508) |  | 381.79(374.21 to 389.49) |  | 10,945(7,454 to 15,969) |  | 508.97(498.98 to 519.14) |  | 1.12(1.04 to 1.20) |  | 87(35 to 185) |  | 3.40(2.72 to 4.21) |  | 96(40 to 206) |  | 4.50(3.60 to 5.59) |  | 1.11(1.03 to 1.19) |
| Estonia |  | 1,565(1,060 to 2,283) |  | 407.28(387.21 to 428.17) |  | 1,620(1,090 to 2,401) |  | 577.43(548.62 to 607.53) |  | 1.42(1.33 to 1.51) |  | 14(5 to 29) |  | 3.64(1.98 to 6.22) |  | 14(6 to 31) |  | 5.14(2.75 to 8.99) |  | 1.41(1.32 to 1.50) |
| Latvia |  | 2,635(1,779 to 4,015) |  | 404.08(388.70 to 419.95) |  | 2,085(1,426 to 2,989) |  | 530.92(507.38 to 555.39) |  | 1.08(1.00 to 1.15) |  | 23(10 to 50) |  | 3.60(2.29 to 5.45) |  | 18(7 to 39) |  | 4.67(2.71 to 7.72) |  | 1.05(0.98 to 1.12) |
| Lithuania |  | 3,408(2,241 to 5,049) |  | 367.88(355.60 to 380.50) |  | 2,956(2,030 to 4,389) |  | 511.61(492.84 to 530.99) |  | 1.30(1.21 to 1.38) |  | 30(12 to 64) |  | 3.27(2.20 to 4.69) |  | 26(10 to 54) |  | 4.49(2.89 to 6.79) |  | 1.26(1.18 to 1.34) |
| Republic of Moldova |  | 3,654(2,387 to 5,430) |  | 321.51(311.09 to 332.22) |  | 4,245(2,924 to 6,178) |  | 472.95(458.07 to 488.27) |  | 1.52(1.37 to 1.67) |  | 32(13 to 71) |  | 2.87(1.96 to 4.07) |  | 37(15 to 80) |  | 4.22(2.92 to 6.02) |  | 1.49(1.36 to 1.62) |
| Russian Federation |  | 152,314(103,930 to 219,218) |  | 403.46(401.42 to 405.52) |  | 181,822(126,785 to 262,061) |  | 523.36(520.83 to 525.91) |  | 1.00(0.96 to 1.04) |  | 1,345(558 to 2,897) |  | 3.58(3.39 to 3.78) |  | 1,588(657 to 3,312) |  | 4.63(4.39 to 4.88) |  | 1.00(0.95 to 1.04) |
| Ukraine |  | 48,637(32,850 to 70,928) |  | 379.14(375.76 to 382.54) |  | 47,338(32,439 to 69,218) |  | 454.13(449.81 to 458.49) |  | 0.79(0.72 to 0.87) |  | 435(181 to 865) |  | 3.41(3.10 to 3.75) |  | 420(177 to 887) |  | 4.08(3.68 to 4.53) |  | 0.77(0.70 to 0.85) |
| Brunei Darussalam |  | 3,193(2,190 to 4,648) |  | 4702.13(4535.11 to 4874.86) |  | 9,325(6,526 to 13,098) |  | 7355.42(7205.88 to 7507.52) |  | 1.51(1.35 to 1.67) |  | 28(12 to 58) |  | 41.20(27.01 to 61.89) |  | 81(37 to 167) |  | 64.19(50.91 to 80.18) |  | 1.50(1.34 to 1.65) |
| Japan |  | 3,663,284(2,626,778 to 5,105,376) |  | 11408.86(11396.98 to 11420.75) |  | 3,065,760(2,174,446 to 4,288,103) |  | 12234.24(12219.97 to 12248.53) |  | 0.21(0.16 to 0.26) |  | 31,814(13,981 to 64,360) |  | 99.57(98.46 to 100.69) |  | 26,525(12,010 to 53,721) |  | 106.56(105.23 to 107.91) |  | 0.21(0.16 to 0.26) |
| Republic of Korea |  | 492,742(343,127 to 711,866) |  | 3898.01(3887.02 to 3909.03) |  | 711,995(490,800 to 1,013,844) |  | 6038.89(6024.30 to 6053.52) |  | 1.11(0.77 to 1.45) |  | 4,338(1,936 to 8,869) |  | 34.18(33.16 to 35.22) |  | 6,184(2,822 to 12,404) |  | 52.77(51.41 to 54.16) |  | 1.10(0.76 to 1.44) |
| Singapore |  | 42,252(29,677 to 60,581) |  | 4448.39(4405.76 to 4491.35) |  | 107,710(75,887 to 155,805) |  | 7156.57(7109.90 to 7203.55) |  | 1.54(1.39 to 1.69) |  | 371(167 to 761) |  | 39.00(35.11 to 43.24) |  | 933(438 to 1,964) |  | 62.48(58.17 to 67.10) |  | 1.53(1.38 to 1.68) |
| Australia |  | 328,294(240,603 to 429,892) |  | 7319.76(7294.68 to 7344.90) |  | 534,957(377,206 to 748,835) |  | 8818.88(8794.89 to 8842.92) |  | 0.38(0.25 to 0.50) |  | 2,867(1,291 to 5,818) |  | 63.98(61.66 to 66.38) |  | 4,658(2,069 to 9,819) |  | 77.03(74.80 to 79.32) |  | 0.38(0.26 to 0.50) |
| New Zealand |  | 96,735(68,161 to 135,306) |  | 10676.30(10609.07 to 10743.86) |  | 130,185(93,691 to 178,019) |  | 10847.39(10788.07 to 10906.99) |  | -0.10(-0.22 to 0.01) |  | 842(374 to 1,744) |  | 92.98(86.80 to 99.49) |  | 1,133(517 to 2,361) |  | 94.60(89.12 to 100.34) |  | -0.09(-0.20 to 0.02) |
| Andorra |  | 833(572 to 1,184) |  | 5512.42(5140.29 to 5906.63) |  | 1,363(946 to 1,920) |  | 6819.98(6440.56 to 7218.79) |  | 0.64(0.54 to 0.75) |  | 8(3 to 16) |  | 49.65(20.49 to 104.62) |  | 12(5 to 25) |  | 61.14(30.19 to 114.93) |  | 0.63(0.53 to 0.74) |
| Austria |  | 141,884(100,446 to 195,369) |  | 7113.87(7076.63 to 7151.26) |  | 149,422(103,525 to 210,469) |  | 7515.50(7476.50 to 7554.68) |  | -0.05(-0.13 to 0.03) |  | 1,262(569 to 2,561) |  | 63.24(59.77 to 66.87) |  | 1,317(579 to 2,692) |  | 66.69(63.05 to 70.51) |  | -0.09(-0.18 to -0.00) |
| Belgium |  | 140,146(96,070 to 199,858) |  | 5706.21(5676.21 to 5736.35) |  | 168,223(117,768 to 234,027) |  | 6761.69(6728.89 to 6794.62) |  | 0.43(0.23 to 0.62) |  | 1,264(554 to 2,626) |  | 51.52(48.71 to 54.47) |  | 1,492(677 to 3,127) |  | 60.37(57.29 to 63.57) |  | 0.39(0.20 to 0.58) |
| Cyprus |  | 8,688(6,029 to 12,221) |  | 4377.30(4285.60 to 4470.53) |  | 22,901(15,867 to 32,918) |  | 6321.90(6235.84 to 6409.08) |  | 1.31(1.14 to 1.48) |  | 79(35 to 160) |  | 39.60(31.32 to 49.48) |  | 205(91 to 422) |  | 56.95(49.03 to 66.06) |  | 1.29(1.13 to 1.46) |
| Denmark |  | 63,407(44,325 to 88,294) |  | 4836.97(4799.09 to 4875.10) |  | 78,688(55,120 to 108,652) |  | 6227.72(6183.78 to 6271.91) |  | 0.79(0.66 to 0.92) |  | 575(264 to 1,171) |  | 43.91(40.37 to 47.70) |  | 712(326 to 1,451) |  | 56.46(52.34 to 60.84) |  | 0.79(0.66 to 0.92) |
| Finland |  | 63,928(44,105 to 90,348) |  | 5002.47(4963.02 to 5042.20) |  | 72,406(49,736 to 101,465) |  | 6342.16(6295.44 to 6389.17) |  | 0.70(0.65 to 0.74) |  | 574(255 to 1,195) |  | 45.06(41.39 to 49.02) |  | 648(293 to 1,345) |  | 57.06(52.70 to 61.72) |  | 0.69(0.65 to 0.74) |
| France |  | 700,300(477,472 to 1,001,461) |  | 4816.24(4804.92 to 4827.58) |  | 858,878(587,904 to 1,211,532) |  | 6058.46(6045.49 to 6071.46) |  | 0.70(0.66 to 0.74) |  | 6,428(2,865 to 13,394) |  | 44.25(43.17 to 45.35) |  | 7,780(3,527 to 16,133) |  | 55.25(54.02 to 56.51) |  | 0.68(0.64 to 0.72) |
| Germany |  | 922,890(657,638 to 1,265,860) |  | 4697.83(4688.11 to 4707.58) |  | 1,011,059(710,978 to 1,415,668) |  | 5882.79(5871.06 to 5894.54) |  | 0.65(0.59 to 0.70) |  | 8,372(3,867 to 17,372) |  | 42.60(41.68 to 43.54) |  | 8,990(4,142 to 18,212) |  | 52.57(51.47 to 53.70) |  | 0.60(0.55 to 0.65) |
| Greece |  | 138,880(95,524 to 198,093) |  | 5503.14(5474.19 to 5532.22) |  | 149,094(102,927 to 213,550) |  | 6886.58(6850.10 to 6923.22) |  | 0.52(0.34 to 0.70) |  | 1,247(556 to 2,564) |  | 49.48(46.77 to 52.31) |  | 1,321(594 to 2,786) |  | 61.67(58.25 to 65.26) |  | 0.51(0.33 to 0.69) |
| Iceland |  | 3,704(2,577 to 5,240) |  | 5680.88(5498.95 to 5867.54) |  | 5,690(4,008 to 8,045) |  | 7109.79(6924.86 to 7298.72) |  | 0.74(0.69 to 0.79) |  | 33(15 to 70) |  | 51.15(35.26 to 72.08) |  | 51(23 to 105) |  | 63.80(47.38 to 84.48) |  | 0.73(0.67 to 0.78) |
| Ireland |  | 46,608(32,072 to 66,641) |  | 5305.13(5256.98 to 5353.62) |  | 76,763(53,795 to 108,080) |  | 6587.16(6539.40 to 6635.21) |  | 0.66(0.56 to 0.75) |  | 419(183 to 868) |  | 47.74(43.27 to 52.56) |  | 682(304 to 1,403) |  | 59.04(54.58 to 63.80) |  | 0.64(0.55 to 0.74) |
| Israel |  | 56,541(39,461 to 81,324) |  | 4642.38(4603.99 to 4681.04) |  | 133,420(92,953 to 189,532) |  | 6012.38(5980.08 to 6044.81) |  | 0.73(0.65 to 0.82) |  | 508(227 to 1,056) |  | 41.71(38.14 to 45.55) |  | 1,189(525 to 2,450) |  | 53.71(50.69 to 56.86) |  | 0.72(0.63 to 0.80) |
| Italy |  | 2,300,244(1,644,619 to 3,187,278) |  | 16092.94(16072.10 to 16113.80) |  | 1,838,182(1,299,434 to 2,549,737) |  | 15307.74(15284.68 to 15330.84) |  | -0.42(-0.54 to -0.29) |  | 20,152(9,095 to 42,633) |  | 141.05(139.10 to 143.01) |  | 16,135(7,202 to 34,264) |  | 135.48(133.31 to 137.67) |  | -0.37(-0.49 to -0.25) |
| Luxembourg |  | 5,303(3,609 to 7,553) |  | 5382.48(5236.40 to 5532.12) |  | 10,820(7,623 to 15,313) |  | 6888.97(6755.86 to 7024.40) |  | 0.79(0.74 to 0.85) |  | 48(21 to 98) |  | 48.48(35.53 to 65.30) |  | 97(42 to 201) |  | 61.83(49.78 to 76.36) |  | 0.79(0.73 to 0.84) |
| Malta |  | 4,817(3,349 to 6,890) |  | 5062.75(4918.84 to 5210.06) |  | 6,526(4,549 to 9,284) |  | 6858.63(6687.61 to 7033.54) |  | 0.91(0.77 to 1.05) |  | 43(19 to 87) |  | 45.70(32.95 to 62.11) |  | 58(26 to 118) |  | 61.50(46.25 to 80.97) |  | 0.89(0.75 to 1.03) |
| Netherlands |  | 189,829(129,978 to 270,172) |  | 4754.12(4732.67 to 4775.66) |  | 220,659(152,819 to 311,862) |  | 6001.76(5976.49 to 6027.11) |  | 0.70(0.65 to 0.74) |  | 1,716(764 to 3,525) |  | 43.00(40.98 to 45.09) |  | 1,977(866 to 4,149) |  | 53.97(51.59 to 56.43) |  | 0.68(0.63 to 0.73) |
| Norway |  | 56,734(39,786 to 80,005) |  | 5353.83(5309.73 to 5398.21) |  | 74,513(52,487 to 106,371) |  | 6138.26(6093.72 to 6183.06) |  | 0.32(0.22 to 0.43) |  | 509(228 to 1,052) |  | 48.13(44.03 to 52.53) |  | 665(294 to 1,380) |  | 55.08(50.93 to 59.51) |  | 0.32(0.22 to 0.43) |
| Portugal |  | 115,805(80,094 to 164,381) |  | 4584.53(4558.14 to 4611.03) |  | 144,968(99,925 to 206,405) |  | 6230.59(6197.20 to 6264.13) |  | 0.74(0.55 to 0.92) |  | 1,041(465 to 2,127) |  | 41.27(38.80 to 43.86) |  | 1,287(591 to 2,577) |  | 55.90(52.77 to 59.19) |  | 0.73(0.55 to 0.91) |
| Spain |  | 476,732(330,562 to 668,783) |  | 4955.51(4941.45 to 4969.61) |  | 622,267(434,253 to 868,566) |  | 6294.89(6278.41 to 6311.41) |  | 0.66(0.53 to 0.80) |  | 4,280(1,967 to 8,939) |  | 44.48(43.16 to 45.84) |  | 5,464(2,466 to 11,567) |  | 55.87(54.32 to 57.46) |  | 0.63(0.50 to 0.76) |
| Sweden |  | 84,771(59,330 to 121,368) |  | 4116.45(4088.45 to 4144.60) |  | 109,846(76,645 to 155,591) |  | 4980.61(4950.72 to 5010.65) |  | 0.49(0.23 to 0.74) |  | 760(337 to 1,572) |  | 37.11(34.49 to 39.89) |  | 978(437 to 2,008) |  | 44.65(41.85 to 47.60) |  | 0.48(0.22 to 0.73) |
| Switzerland |  | 93,997(65,249 to 134,415) |  | 5289.81(5255.61 to 5324.20) |  | 117,520(81,335 to 166,909) |  | 5959.38(5924.29 to 5994.66) |  | 0.41(0.40 to 0.43) |  | 843(369 to 1,733) |  | 47.54(44.34 to 50.93) |  | 1,046(467 to 2,144) |  | 53.42(50.13 to 56.89) |  | 0.41(0.39 to 0.42) |
| United Kingdom |  | 835,303(583,559 to 1,190,025) |  | 5845.85(5833.25 to 5858.47) |  | 1,125,561(789,645 to 1,604,716) |  | 7306.96(7293.28 to 7320.66) |  | 0.48(0.35 to 0.61) |  | 7,500(3,338 to 15,454) |  | 52.51(51.32 to 53.72) |  | 10,022(4,504 to 20,704) |  | 65.39(64.10 to 66.70) |  | 0.47(0.34 to 0.60) |
| Argentina |  | 178,995(123,508 to 259,812) |  | 2239.19(2228.82 to 2249.59) |  | 411,903(289,126 to 588,383) |  | 3450.24(3439.69 to 3460.82) |  | 1.35(1.17 to 1.52) |  | 1,588(710 to 3,249) |  | 19.86(18.89 to 20.86) |  | 3,647(1,607 to 7,541) |  | 30.61(29.62 to 31.62) |  | 1.35(1.17 to 1.52) |
| Chile |  | 85,928(59,765 to 124,517) |  | 2372.70(2356.76 to 2388.74) |  | 194,117(135,114 to 282,208) |  | 4085.53(4067.24 to 4103.89) |  | 1.71(1.40 to 2.03) |  | 766(330 to 1,584) |  | 21.04(19.56 to 22.60) |  | 1,711(727 to 3,509) |  | 36.08(34.38 to 37.86) |  | 1.70(1.39 to 2.01) |
| Uruguay |  | 17,184(11,950 to 24,788) |  | 2291.62(2257.48 to 2326.15) |  | 31,556(22,137 to 46,176) |  | 3781.93(3740.12 to 3824.12) |  | 1.61(1.42 to 1.81) |  | 153(66 to 316) |  | 20.35(17.25 to 23.85) |  | 278(120 to 555) |  | 33.46(29.63 to 37.67) |  | 1.60(1.40 to 1.79) |
| Canada |  | 206,438(143,946 to 294,139) |  | 2766.41(2754.36 to 2778.49) |  | 295,926(207,514 to 431,710) |  | 3546.13(3533.15 to 3559.15) |  | 0.72(0.62 to 0.83) |  | 1,854(823 to 3,826) |  | 24.86(23.73 to 26.04) |  | 2,644(1,124 to 5,396) |  | 31.79(30.57 to 33.05) |  | 0.72(0.61 to 0.82) |
| United States of America |  | 4,080,725(2,884,086 to 5,719,265) |  | 6017.23(6011.36 to 6023.12) |  | 5,774,954(4,333,610 to 7,593,437) |  | 7599.79(7593.56 to 7606.02) |  | -0.58(-1.12 to -0.04) |  | 36,560(16,174 to 76,006) |  | 53.91(53.36 to 54.47) |  | 51,044(23,316 to 102,444) |  | 67.35(66.77 to 67.94) |  | -0.60(-1.13 to -0.06) |
| Antigua and Barbuda |  | 369(248 to 528) |  | 2251.21(2024.73 to 2498.82) |  | 723(493 to 1,023) |  | 2997.85(2781.27 to 3227.98) |  | 0.81(0.75 to 0.86) |  | 3(1 to 7) |  | 19.96(4.42 to 62.21) |  | 6(3 to 13) |  | 26.50(9.97 to 58.91) |  | 0.80(0.75 to 0.86) |
| Bahamas |  | 2,088(1,421 to 3,011) |  | 2857.94(2734.65 to 2985.89) |  | 3,661(2,478 to 5,349) |  | 3409.45(3299.60 to 3522.10) |  | 0.52(0.44 to 0.59) |  | 19(8 to 40) |  | 25.32(15.01 to 40.86) |  | 32(14 to 70) |  | 30.16(20.65 to 42.64) |  | 0.51(0.44 to 0.59) |
| Barbados |  | 1,946(1,331 to 2,781) |  | 2819.15(2694.50 to 2948.48) |  | 2,347(1,600 to 3,359) |  | 3318.26(3183.60 to 3457.48) |  | 0.47(0.44 to 0.51) |  | 17(8 to 37) |  | 24.97(14.56 to 40.57) |  | 21(9 to 44) |  | 29.32(17.93 to 45.71) |  | 0.47(0.43 to 0.50) |
| Belize |  | 942(634 to 1,332) |  | 2233.04(2084.85 to 2390.94) |  | 3,898(2,678 to 5,492) |  | 3225.15(3124.19 to 3328.67) |  | 0.97(0.70 to 1.24) |  | 9(4 to 18) |  | 20.09(8.49 to 43.79) |  | 35(15 to 75) |  | 28.77(19.98 to 40.27) |  | 0.94(0.68 to 1.20) |
| Cuba |  | 75,591(50,737 to 108,226) |  | 2460.42(2442.73 to 2478.21) |  | 80,618(54,535 to 114,988) |  | 3268.01(3245.03 to 3291.13) |  | 0.94(0.89 to 0.99) |  | 671(288 to 1,390) |  | 21.73(20.10 to 23.47) |  | 709(306 to 1,483) |  | 28.88(26.75 to 31.14) |  | 0.95(0.90 to 1.00) |
| Dominica |  | 381(257 to 548) |  | 2236.60(2012.38 to 2480.89) |  | 504(339 to 728) |  | 3067.91(2805.63 to 3348.52) |  | 0.86(0.74 to 0.98) |  | 3(1 to 7) |  | 19.85(4.45 to 59.89) |  | 4(2 to 9) |  | 27.17(8.08 to 67.20) |  | 0.85(0.73 to 0.97) |
| Dominican Republic |  | 36,972(25,138 to 53,183) |  | 1954.98(1934.37 to 1975.78) |  | 89,880(60,962 to 131,328) |  | 3092.81(3072.60 to 3113.12) |  | 1.55(1.46 to 1.64) |  | 333(146 to 678) |  | 17.51(15.61 to 19.60) |  | 792(340 to 1,650) |  | 27.21(25.35 to 29.18) |  | 1.53(1.44 to 1.63) |
| Grenada |  | 376(254 to 540) |  | 1927.57(1731.98 to 2142.20) |  | 708(474 to 1,012) |  | 2764.78(2563.88 to 2977.82) |  | 1.00(0.87 to 1.14) |  | 3(1 to 7) |  | 17.10(3.73 to 55.13) |  | 6(3 to 14) |  | 24.49(9.19 to 53.65) |  | 1.00(0.87 to 1.13) |
| Guyana |  | 4,089(2,744 to 6,035) |  | 1993.36(1930.71 to 2057.81) |  | 5,881(3,962 to 8,434) |  | 2886.18(2812.45 to 2961.43) |  | 1.17(1.07 to 1.27) |  | 36(16 to 78) |  | 17.59(12.18 to 24.99) |  | 52(22 to 112) |  | 25.37(18.90 to 33.44) |  | 1.18(1.06 to 1.30) |
| Haiti |  | 23,190(15,399 to 33,920) |  | 1504.48(1484.76 to 1524.43) |  | 66,178(45,397 to 93,347) |  | 1862.16(1847.95 to 1876.46) |  | 0.83(0.78 to 0.88) |  | 206(91 to 443) |  | 13.28(11.49 to 15.30) |  | 571(246 to 1,205) |  | 16.06(14.76 to 17.44) |  | 0.77(0.71 to 0.83) |
| Jamaica |  | 13,004(8,767 to 18,354) |  | 2172.46(2133.96 to 2211.57) |  | 22,664(15,359 to 32,580) |  | 2922.68(2884.65 to 2961.11) |  | 0.97(0.89 to 1.04) |  | 117(50 to 244) |  | 19.29(15.83 to 23.39) |  | 201(86 to 429) |  | 25.91(22.44 to 29.79) |  | 0.96(0.89 to 1.04) |
| Saint Lucia |  | 727(487 to 1,027) |  | 2125.05(1967.92 to 2293.09) |  | 1,300(886 to 1,816) |  | 2854.98(2700.23 to 3016.90) |  | 0.68(0.54 to 0.82) |  | 7(3 to 14) |  | 18.83(6.95 to 44.10) |  | 11(5 to 24) |  | 25.26(12.70 to 45.95) |  | 0.67(0.53 to 0.82) |
| Saint Vincent and the Grenadines |  | 513(344 to 729) |  | 1941.01(1768.83 to 2128.21) |  | 811(549 to 1,163) |  | 2926.26(2727.65 to 3135.83) |  | 1.37(1.27 to 1.47) |  | 5(2 to 10) |  | 17.25(4.89 to 49.07) |  | 7(3 to 15) |  | 25.92(10.52 to 53.64) |  | 1.36(1.26 to 1.46) |
| Suriname |  | 2,165(1,456 to 3,052) |  | 2222.39(2128.05 to 2320.10) |  | 4,392(2,978 to 6,270) |  | 3032.21(2943.07 to 3123.42) |  | 0.99(0.95 to 1.03) |  | 19(8 to 41) |  | 19.66(11.76 to 31.25) |  | 39(17 to 83) |  | 26.73(18.97 to 36.67) |  | 0.99(0.95 to 1.03) |
| Trinidad and Tobago |  | 6,916(4,642 to 9,729) |  | 2238.19(2185.29 to 2292.14) |  | 11,013(7,377 to 15,772) |  | 3245.88(3184.48 to 3308.23) |  | 1.32(1.17 to 1.46) |  | 62(27 to 131) |  | 20.12(15.40 to 25.96) |  | 97(42 to 201) |  | 28.70(23.18 to 35.23) |  | 1.29(1.15 to 1.44) |
| Bolivia (Plurinational State of) |  | 64,629(43,587 to 93,627) |  | 4239.97(4206.73 to 4273.42) |  | 178,507(120,329 to 252,009) |  | 5703.66(5677.18 to 5730.23) |  | 1.00(0.92 to 1.08) |  | 563(241 to 1,192) |  | 36.81(33.78 to 40.07) |  | 1,543(673 to 3,310) |  | 49.24(46.81 to 51.77) |  | 0.98(0.91 to 1.06) |
| Ecuador |  | 135,158(94,265 to 189,727) |  | 5349.53(5320.32 to 5378.87) |  | 333,593(234,116 to 462,511) |  | 7052.64(7028.70 to 7076.64) |  | 0.88(0.64 to 1.11) |  | 1,179(528 to 2,504) |  | 46.47(43.79 to 49.29) |  | 2,897(1,265 to 6,074) |  | 61.21(59.00 to 63.48) |  | 0.85(0.61 to 1.08) |
| Peru |  | 233,536(156,216 to 328,299) |  | 4304.77(4286.90 to 4322.71) |  | 593,075(404,289 to 857,160) |  | 6134.63(6119.00 to 6150.30) |  | 1.24(1.20 to 1.28) |  | 2,029(894 to 4,430) |  | 37.31(35.67 to 39.02) |  | 5,107(2,214 to 10,838) |  | 52.84(51.40 to 54.31) |  | 1.22(1.18 to 1.26) |
| Colombia |  | 275,131(184,450 to 393,294) |  | 3154.44(3142.40 to 3166.52) |  | 544,010(373,322 to 791,226) |  | 4133.29(4122.30 to 4144.31) |  | 0.79(0.73 to 0.85) |  | 2,470(1,071 to 5,283) |  | 28.18(27.06 to 29.35) |  | 4,842(2,099 to 10,268) |  | 36.77(35.74 to 37.82) |  | 0.79(0.74 to 0.85) |
| Costa Rica |  | 30,739(20,846 to 44,497) |  | 3912.89(3868.37 to 3957.88) |  | 67,302(45,653 to 97,529) |  | 5173.80(5134.59 to 5213.25) |  | 0.80(0.74 to 0.86) |  | 270(120 to 560) |  | 34.19(30.15 to 38.70) |  | 587(252 to 1,280) |  | 45.23(41.63 to 49.08) |  | 0.79(0.73 to 0.86) |
| El Salvador |  | 41,816(28,303 to 61,312) |  | 3195.13(3163.63 to 3226.89) |  | 80,763(55,365 to 115,886) |  | 4530.90(4499.61 to 4562.36) |  | 1.06(0.95 to 1.16) |  | 370(160 to 784) |  | 28.14(25.26 to 31.29) |  | 704(308 to 1,483) |  | 39.45(36.58 to 42.49) |  | 1.04(0.93 to 1.14) |
| Guatemala |  | 49,939(33,896 to 72,593) |  | 2725.46(2700.89 to 2750.22) |  | 172,330(113,759 to 251,050) |  | 3904.87(3886.27 to 3923.55) |  | 0.96(0.84 to 1.07) |  | 432(184 to 921) |  | 23.46(21.23 to 25.88) |  | 1,481(639 to 3,077) |  | 33.45(31.75 to 35.22) |  | 0.97(0.85 to 1.08) |
| Honduras |  | 27,670(18,779 to 40,542) |  | 2622.23(2590.33 to 2654.48) |  | 112,488(77,453 to 163,302) |  | 3942.76(3919.53 to 3966.11) |  | 1.27(1.16 to 1.38) |  | 241(105 to 522) |  | 22.75(19.88 to 25.98) |  | 973(428 to 2,064) |  | 34.01(31.88 to 36.25) |  | 1.24(1.12 to 1.36) |
| Mexico |  | 1,473,996(1,016,593 to 2,070,229) |  | 6742.59(6731.37 to 6753.84) |  | 2,379,832(1,670,385 to 3,278,940) |  | 6800.66(6792.02 to 6809.32) |  | -0.53(-0.77 to -0.28) |  | 12,922(5,713 to 27,108) |  | 58.76(57.72 to 59.82) |  | 20,532(8,964 to 43,600) |  | 58.71(57.91 to 59.52) |  | -0.55(-0.79 to -0.30) |
| Nicaragua |  | 26,900(18,100 to 39,332) |  | 2976.97(2939.85 to 3014.52) |  | 77,229(51,193 to 110,370) |  | 4240.35(4210.45 to 4270.42) |  | 1.05(0.96 to 1.14) |  | 236(101 to 499) |  | 25.99(22.64 to 29.79) |  | 673(293 to 1,455) |  | 36.92(34.18 to 39.83) |  | 1.03(0.94 to 1.13) |
| Panama |  | 16,939(11,459 to 24,684) |  | 2754.50(2712.44 to 2797.12) |  | 47,084(32,341 to 69,297) |  | 4403.74(4364.02 to 4443.73) |  | 1.36(1.27 to 1.45) |  | 147(64 to 320) |  | 23.83(20.07 to 28.17) |  | 407(175 to 884) |  | 38.02(34.41 to 41.91) |  | 1.35(1.25 to 1.44) |
| Venezuela (Bolivarian Republic of) |  | 185,085(125,291 to 271,448) |  | 3796.62(3778.98 to 3814.34) |  | 325,888(220,478 to 476,414) |  | 4777.65(4761.06 to 4794.28) |  | 0.67(0.60 to 0.74) |  | 1,625(703 to 3,462) |  | 33.21(31.58 to 34.91) |  | 2,831(1,236 to 5,927) |  | 41.67(40.13 to 43.26) |  | 0.66(0.59 to 0.73) |
| Brazil |  | 411,776(278,883 to 594,523) |  | 1057.06(1053.79 to 1060.34) |  | 672,524(457,339 to 952,793) |  | 1140.88(1138.13 to 1143.63) |  | -0.21(-0.39 to -0.03) |  | 3,691(1,576 to 7,785) |  | 9.44(9.13 to 9.75) |  | 5,923(2,525 to 12,494) |  | 10.08(9.83 to 10.35) |  | -0.23(-0.41 to -0.05) |
| Paraguay |  | 6,816(4,498 to 10,123) |  | 730.41(712.81 to 748.36) |  | 21,775(14,725 to 31,991) |  | 1145.87(1130.66 to 1161.25) |  | 1.67(1.61 to 1.73) |  | 61(25 to 129) |  | 6.47(4.91 to 8.41) |  | 192(81 to 399) |  | 10.07(8.70 to 11.62) |  | 1.65(1.59 to 1.71) |
| Algeria |  | 167,735(115,886 to 239,375) |  | 2879.72(2865.28 to 2894.22) |  | 473,938(322,019 to 677,264) |  | 4213.51(4201.43 to 4225.63) |  | 1.40(1.36 to 1.45) |  | 1,507(640 to 3,017) |  | 25.65(24.31 to 27.05) |  | 4,165(1,810 to 8,711) |  | 37.15(36.02 to 38.31) |  | 1.37(1.31 to 1.43) |
| Bahrain |  | 4,701(3,197 to 6,723) |  | 4017.43(3897.26 to 4141.19) |  | 14,957(10,285 to 21,235) |  | 4579.65(4506.28 to 4653.97) |  | 0.40(0.35 to 0.46) |  | 42(18 to 88) |  | 35.50(25.09 to 49.96) |  | 132(57 to 279) |  | 40.40(33.77 to 48.01) |  | 0.41(0.36 to 0.45) |
| Egypt |  | 456,388(310,991 to 649,830) |  | 3480.04(3469.81 to 3490.30) |  | 1,146,698(794,113 to 1,621,016) |  | 4406.99(4398.91 to 4415.09) |  | 0.63(0.56 to 0.69) |  | 4,215(1,856 to 8,912) |  | 31.90(30.93 to 32.89) |  | 10,292(4,576 to 21,429) |  | 39.46(38.70 to 40.23) |  | 0.52(0.45 to 0.60) |
| Iran (Islamic Republic of) |  | 403,580(277,962 to 567,348) |  | 3197.28(3187.01 to 3207.57) |  | 990,123(692,440 to 1,403,706) |  | 4240.11(4231.51 to 4248.73) |  | 1.37(1.01 to 1.73) |  | 3,757(1,659 to 7,962) |  | 29.44(28.47 to 30.45) |  | 9,027(4,016 to 18,937) |  | 38.99(38.17 to 39.83) |  | 1.39(1.03 to 1.75) |
| Iraq |  | 137,083(95,071 to 198,011) |  | 3375.77(3357.17 to 3394.47) |  | 409,470(278,284 to 582,149) |  | 3881.34(3869.39 to 3893.32) |  | 0.61(0.49 to 0.73) |  | 1,223(531 to 2,563) |  | 29.83(28.12 to 31.64) |  | 3,616(1,608 to 7,517) |  | 34.14(33.03 to 35.28) |  | 0.60(0.48 to 0.72) |
| Jordan |  | 26,160(18,118 to 37,281) |  | 3135.19(3094.83 to 3176.01) |  | 122,993(86,289 to 173,427) |  | 3972.24(3949.95 to 3994.64) |  | 0.87(0.81 to 0.93) |  | 239(103 to 500) |  | 28.24(24.55 to 32.41) |  | 1,092(467 to 2,328) |  | 35.12(33.06 to 37.29) |  | 0.79(0.70 to 0.88) |
| Kuwait |  | 18,017(12,309 to 25,680) |  | 4289.61(4224.90 to 4355.27) |  | 75,834(52,203 to 107,727) |  | 5169.37(5129.86 to 5209.16) |  | 0.80(0.74 to 0.86) |  | 161(71 to 343) |  | 37.98(32.14 to 44.85) |  | 663(292 to 1,370) |  | 45.70(42.04 to 49.67) |  | 0.79(0.73 to 0.85) |
| Lebanon |  | 25,434(17,494 to 36,660) |  | 3388.97(3347.07 to 3431.29) |  | 66,303(46,191 to 95,529) |  | 4456.87(4422.29 to 4491.68) |  | 0.89(0.87 to 0.91) |  | 223(97 to 454) |  | 29.65(25.86 to 33.87) |  | 576(248 to 1,188) |  | 38.89(35.71 to 42.30) |  | 0.90(0.88 to 0.91) |
| Libya |  | 34,838(23,616 to 49,812) |  | 3826.88(3784.19 to 3870.01) |  | 84,103(58,378 to 119,957) |  | 4233.95(4205.24 to 4262.81) |  | 0.46(0.42 to 0.51) |  | 313(138 to 640) |  | 34.01(30.11 to 38.35) |  | 742(320 to 1,524) |  | 37.52(34.85 to 40.33) |  | 0.47(0.41 to 0.52) |
| Morocco |  | 183,651(125,030 to 260,945) |  | 2914.65(2901.01 to 2928.34) |  | 375,835(258,245 to 539,098) |  | 3880.30(3867.90 to 3892.74) |  | 1.01(0.97 to 1.05) |  | 1,681(750 to 3,502) |  | 26.45(25.17 to 27.79) |  | 3,344(1,488 to 6,919) |  | 34.57(33.41 to 35.76) |  | 0.96(0.92 to 1.00) |
| Palestine |  | 12,937(8,736 to 18,841) |  | 2928.06(2875.02 to 2981.95) |  | 48,483(33,177 to 68,169) |  | 3713.23(3679.67 to 3747.04) |  | 0.75(0.70 to 0.80) |  | 116(48 to 243) |  | 25.90(21.15 to 31.53) |  | 430(194 to 912) |  | 32.75(29.68 to 36.08) |  | 0.75(0.70 to 0.80) |
| Oman |  | 9,450(6,369 to 13,615) |  | 2781.87(2724.48 to 2840.31) |  | 47,064(32,191 to 67,086) |  | 4600.78(4558.46 to 4643.42) |  | 1.74(1.65 to 1.82) |  | 84(37 to 176) |  | 24.56(19.46 to 30.80) |  | 413(179 to 864) |  | 40.53(36.64 to 44.74) |  | 1.74(1.65 to 1.83) |
| Qatar |  | 3,427(2,364 to 4,970) |  | 4290.64(4143.60 to 4442.61) |  | 28,425(19,394 to 41,026) |  | 5075.53(5011.99 to 5139.81) |  | 0.54(0.50 to 0.58) |  | 30(13 to 62) |  | 37.91(25.29 to 56.17) |  | 249(107 to 512) |  | 44.75(38.94 to 51.33) |  | 0.53(0.49 to 0.57) |
| Saudi Arabia |  | 115,261(79,071 to 164,100) |  | 3525.16(3504.00 to 3546.43) |  | 507,689(349,075 to 728,297) |  | 4975.37(4961.40 to 4989.37) |  | 1.22(1.19 to 1.24) |  | 1,026(448 to 2,115) |  | 31.10(29.15 to 33.17) |  | 4,457(2,006 to 9,407) |  | 43.86(42.56 to 45.20) |  | 1.21(1.19 to 1.23) |
| Syrian Arab Republic |  | 80,314(54,095 to 114,487) |  | 2916.71(2895.49 to 2938.08) |  | 148,808(103,269 to 211,471) |  | 3873.00(3852.07 to 3894.03) |  | 0.99(0.91 to 1.07) |  | 714(320 to 1,489) |  | 25.63(23.68 to 27.71) |  | 1,302(574 to 2,695) |  | 33.89(31.95 to 35.93) |  | 0.98(0.90 to 1.07) |
| Tunisia |  | 57,936(39,105 to 85,549) |  | 2824.65(2801.07 to 2848.40) |  | 121,990(84,642 to 172,631) |  | 3978.43(3955.79 to 4001.16) |  | 1.21(1.17 to 1.26) |  | 533(229 to 1,114) |  | 25.78(23.58 to 28.16) |  | 1,078(478 to 2,257) |  | 35.39(33.27 to 37.61) |  | 1.19(1.14 to 1.24) |
| Türkiye |  | 380,697(260,565 to 548,159) |  | 2665.92(2657.31 to 2674.56) |  | 807,612(556,631 to 1,152,639) |  | 3735.04(3726.85 to 3743.25) |  | 1.18(1.12 to 1.24) |  | 3,511(1,559 to 7,326) |  | 24.42(23.61 to 25.26) |  | 7,112(3,160 to 15,020) |  | 33.01(32.24 to 33.79) |  | 1.06(0.99 to 1.14) |
| United Arab Emirates |  | 12,252(8,390 to 17,614) |  | 3527.31(3461.62 to 3594.29) |  | 79,711(55,278 to 115,614) |  | 4644.27(4607.53 to 4681.26) |  | 0.85(0.73 to 0.96) |  | 109(48 to 228) |  | 31.10(25.24 to 38.38) |  | 688(301 to 1,445) |  | 40.86(37.45 to 44.54) |  | 0.84(0.72 to 0.96) |
| Yemen |  | 55,289(38,362 to 81,404) |  | 2021.24(2003.83 to 2038.78) |  | 208,738(140,318 to 303,064) |  | 2500.45(2489.55 to 2511.39) |  | 0.99(0.86 to 1.12) |  | 490(215 to 1,044) |  | 17.76(16.17 to 19.49) |  | 1,843(793 to 3,949) |  | 21.97(20.96 to 23.02) |  | 0.99(0.86 to 1.12) |
| Afghanistan |  | 37,143(25,615 to 53,187) |  | 1725.83(1706.91 to 1744.96) |  | 177,431(121,379 to 253,388) |  | 2480.11(2467.98 to 2492.28) |  | 1.68(1.40 to 1.97) |  | 318(138 to 677) |  | 14.72(13.02 to 16.62) |  | 1,538(658 to 3,276) |  | 21.28(20.17 to 22.43) |  | 1.73(1.44 to 2.02) |
| Bangladesh |  | 173,221(116,993 to 250,593) |  | 713.93(710.41 to 717.47) |  | 543,249(373,346 to 772,778) |  | 1178.89(1175.75 to 1182.03) |  | 1.88(1.74 to 2.01) |  | 1,564(679 to 3,253) |  | 6.36(6.03 to 6.70) |  | 4,814(2,040 to 10,235) |  | 10.43(10.14 to 10.73) |  | 1.84(1.71 to 1.96) |
| Bhutan |  | 1,454(992 to 2,083) |  | 1026.57(971.72 to 1084.19) |  | 3,795(2,595 to 5,461) |  | 1821.77(1764.05 to 1880.99) |  | 2.11(1.99 to 2.24) |  | 13(6 to 28) |  | 9.09(4.65 to 16.79) |  | 34(15 to 71) |  | 16.13(11.13 to 22.76) |  | 2.12(2.00 to 2.25) |
| India |  | 2,610,616(1,872,882 to 3,616,381) |  | 1297.64(1296.04 to 1299.23) |  | 9,198,654(6,496,430 to 12,854,699) |  | 2429.87(2428.30 to 2431.44) |  | 2.35(2.20 to 2.50) |  | 23,266(10,189 to 49,124) |  | 11.49(11.34 to 11.64) |  | 80,390(35,130 to 168,795) |  | 21.22(21.07 to 21.37) |  | 2.29(2.14 to 2.43) |
| Nepal |  | 33,467(22,997 to 48,123) |  | 739.07(731.01 to 747.20) |  | 114,524(78,522 to 162,684) |  | 1255.47(1248.16 to 1262.81) |  | 1.81(1.76 to 1.86) |  | 297(125 to 641) |  | 6.53(5.80 to 7.35) |  | 1,031(436 to 2,129) |  | 11.26(10.58 to 11.98) |  | 1.80(1.76 to 1.84) |
| Pakistan |  | 293,226(206,095 to 424,056) |  | 1259.71(1255.04 to 1264.39) |  | 889,149(606,484 to 1,275,170) |  | 1468.96(1465.88 to 1472.04) |  | 0.54(0.39 to 0.69) |  | 2,640(1,126 to 5,676) |  | 11.23(10.79 to 11.68) |  | 8,059(3,457 to 17,415) |  | 13.26(12.97 to 13.55) |  | 0.55(0.38 to 0.73) |
| Angola |  | 17,769(12,142 to 25,780) |  | 782.54(770.72 to 794.51) |  | 107,216(72,476 to 155,717) |  | 1407.11(1398.50 to 1415.76) |  | 1.80(1.65 to 1.95) |  | 154(66 to 310) |  | 6.74(5.69 to 7.95) |  | 934(395 to 1,913) |  | 12.16(11.38 to 12.99) |  | 1.82(1.67 to 1.96) |
| Central African Republic |  | 6,478(4,426 to 9,487) |  | 1018.78(993.36 to 1044.75) |  | 15,130(10,325 to 21,774) |  | 1104.23(1086.32 to 1122.39) |  | 0.05(-0.08 to 0.18) |  | 57(24 to 123) |  | 8.88(6.66 to 11.68) |  | 133(57 to 280) |  | 9.64(8.04 to 11.50) |  | 0.07(-0.06 to 0.19) |
| Congo |  | 6,074(4,131 to 8,760) |  | 1091.38(1062.84 to 1120.59) |  | 21,729(14,889 to 31,364) |  | 1520.75(1500.50 to 1541.22) |  | 0.90(0.76 to 1.04) |  | 53(22 to 101) |  | 9.34(6.89 to 12.52) |  | 186(82 to 395) |  | 12.99(11.18 to 15.03) |  | 0.88(0.72 to 1.04) |
| Democratic Republic of the Congo |  | 70,429(48,180 to 102,715) |  | 835.96(829.58 to 842.37) |  | 257,726(177,439 to 372,016) |  | 1218.93(1214.11 to 1223.78) |  | 1.19(1.02 to 1.35) |  | 610(256 to 1,246) |  | 7.18(6.60 to 7.80) |  | 2,243(974 to 4,607) |  | 10.53(10.09 to 10.99) |  | 1.18(0.99 to 1.36) |
| Equatorial Guinea |  | 872(597 to 1,280) |  | 893.98(834.16 to 957.41) |  | 7,617(5,162 to 11,043) |  | 2089.87(2042.20 to 2138.52) |  | 2.98(2.53 to 3.43) |  | 8(3 to 16) |  | 7.66(3.12 to 16.48) |  | 66(29 to 136) |  | 17.99(13.83 to 23.19) |  | 3.00(2.54 to 3.45) |
| Gabon |  | 2,705(1,852 to 3,904) |  | 1233.86(1185.29 to 1284.28) |  | 9,094(6,035 to 13,368) |  | 1861.45(1822.87 to 1900.69) |  | 1.18(1.03 to 1.32) |  | 24(10 to 49) |  | 10.63(6.59 to 16.80) |  | 79(33 to 167) |  | 16.00(12.62 to 20.08) |  | 1.18(1.04 to 1.33) |
| Burundi |  | 10,451(7,200 to 14,851) |  | 835.78(819.25 to 852.61) |  | 27,323(18,498 to 39,416) |  | 884.19(873.34 to 895.16) |  | 0.09(0.02 to 0.17) |  | 92(39 to 196) |  | 7.26(5.80 to 9.04) |  | 237(99 to 501) |  | 7.64(6.66 to 8.75) |  | 0.10(0.00 to 0.19) |
| Comoros |  | 1,298(884 to 1,898) |  | 1243.99(1174.69 to 1316.76) |  | 2,882(1,967 to 4,172) |  | 1477.86(1424.15 to 1533.14) |  | 0.31(0.14 to 0.47) |  | 12(5 to 25) |  | 11.03(5.46 to 20.61) |  | 26(11 to 53) |  | 13.17(8.56 to 19.45) |  | 0.34(0.18 to 0.50) |
| Djibouti |  | 956(659 to 1,431) |  | 985.57(921.43 to 1053.65) |  | 5,407(3,636 to 7,886) |  | 1675.39(1630.88 to 1720.86) |  | 1.81(1.67 to 1.96) |  | 8(3 to 18) |  | 8.51(3.59 to 18.24) |  | 47(20 to 101) |  | 14.51(10.64 to 19.40) |  | 1.83(1.69 to 1.97) |
| Eritrea |  | 5,742(3,863 to 8,226) |  | 738.67(719.13 to 758.66) |  | 18,274(12,406 to 26,466) |  | 1107.49(1091.29 to 1123.88) |  | 1.27(1.06 to 1.47) |  | 51(22 to 107) |  | 6.47(4.77 to 8.65) |  | 160(66 to 337) |  | 9.67(8.22 to 11.33) |  | 1.26(1.07 to 1.46) |
| Ethiopia |  | 82,941(57,103 to 119,288) |  | 750.56(745.28 to 755.88) |  | 312,484(218,794 to 453,237) |  | 1142.60(1138.47 to 1146.75) |  | 1.56(1.50 to 1.63) |  | 727(305 to 1,519) |  | 6.52(6.04 to 7.04) |  | 2,745(1,171 to 5,940) |  | 9.95(9.57 to 10.34) |  | 1.55(1.49 to 1.61) |
| Kenya |  | 58,310(40,962 to 83,040) |  | 1151.23(1141.29 to 1161.24) |  | 188,160(131,310 to 271,122) |  | 1430.64(1424.02 to 1437.28) |  | 0.53(0.42 to 0.64) |  | 512(217 to 1,080) |  | 9.99(9.09 to 10.97) |  | 1,632(699 to 3,439) |  | 12.33(11.72 to 12.96) |  | 0.54(0.43 to 0.65) |
| Madagascar |  | 24,567(16,738 to 35,606) |  | 921.30(909.33 to 933.42) |  | 76,585(52,346 to 111,847) |  | 1064.57(1056.85 to 1072.33) |  | 0.53(0.48 to 0.58) |  | 217(93 to 464) |  | 8.07(6.99 to 9.30) |  | 680(296 to 1,392) |  | 9.39(8.68 to 10.14) |  | 0.57(0.52 to 0.62) |
| Malawi |  | 27,859(18,898 to 40,625) |  | 1238.62(1223.45 to 1253.97) |  | 68,529(47,298 to 99,565) |  | 1377.41(1366.67 to 1388.22) |  | 0.44(0.32 to 0.57) |  | 240(101 to 520) |  | 10.57(9.22 to 12.10) |  | 590(260 to 1,208) |  | 11.77(10.80 to 12.82) |  | 0.42(0.30 to 0.54) |
| Mauritius |  | 13,545(9,484 to 19,754) |  | 4492.62(4416.43 to 4569.94) |  | 22,837(15,905 to 32,348) |  | 7204.68(7110.82 to 7299.52) |  | 1.67(1.54 to 1.79) |  | 119(53 to 244) |  | 39.40(32.57 to 47.41) |  | 199(87 to 418) |  | 63.06(54.55 to 72.59) |  | 1.66(1.54 to 1.77) |
| Mozambique |  | 28,139(19,055 to 41,013) |  | 901.62(890.90 to 912.45) |  | 102,859(69,729 to 150,495) |  | 1364.10(1355.43 to 1372.82) |  | 1.33(1.25 to 1.42) |  | 243(102 to 473) |  | 7.74(6.78 to 8.82) |  | 886(378 to 1,813) |  | 11.64(10.85 to 12.47) |  | 1.32(1.23 to 1.42) |
| Rwanda |  | 17,519(12,099 to 25,315) |  | 1088.36(1071.59 to 1105.37) |  | 48,432(33,282 to 69,759) |  | 1377.45(1365.03 to 1389.98) |  | 0.81(0.75 to 0.88) |  | 155(66 to 327) |  | 9.50(8.00 to 11.25) |  | 421(182 to 902) |  | 11.92(10.79 to 13.15) |  | 0.78(0.71 to 0.86) |
| Seychelles |  | 867(606 to 1,238) |  | 4793.17(4467.30 to 5139.60) |  | 1,630(1,142 to 2,303) |  | 6738.58(6412.66 to 7077.41) |  | 1.08(1.02 to 1.14) |  | 8(3 to 16) |  | 42.19(17.13 to 92.11) |  | 14(6 to 30) |  | 59.28(32.45 to 100.31) |  | 1.07(1.01 to 1.13) |
| Somalia |  | 15,364(10,466 to 22,018) |  | 914.77(900.12 to 929.63) |  | 51,131(34,940 to 75,676) |  | 1068.11(1058.57 to 1077.72) |  | 0.59(0.55 to 0.64) |  | 133(57 to 279) |  | 7.88(6.58 to 9.39) |  | 446(187 to 941) |  | 9.25(8.38 to 10.19) |  | 0.62(0.57 to 0.67) |
| United Republic of Tanzania |  | 71,601(48,131 to 104,790) |  | 1205.24(1195.96 to 1214.58) |  | 217,233(148,029 to 310,661) |  | 1467.31(1461.00 to 1473.65) |  | 0.63(0.54 to 0.72) |  | 617(260 to 1,230) |  | 10.26(9.42 to 11.16) |  | 1,861(820 to 3,833) |  | 12.52(11.94 to 13.12) |  | 0.62(0.53 to 0.70) |
| Uganda |  | 39,431(26,728 to 56,976) |  | 1035.67(1024.81 to 1046.65) |  | 133,360(92,200 to 191,728) |  | 1287.88(1280.66 to 1295.14) |  | 0.72(0.69 to 0.76) |  | 346(143 to 743) |  | 8.97(7.99 to 10.06) |  | 1,154(492 to 2,393) |  | 11.06(10.40 to 11.75) |  | 0.67(0.62 to 0.72) |
| Zambia |  | 25,291(17,032 to 37,009) |  | 1388.42(1370.22 to 1406.84) |  | 83,190(55,693 to 121,602) |  | 1691.37(1679.49 to 1703.33) |  | 0.51(0.46 to 0.56) |  | 218(90 to 472) |  | 11.84(10.22 to 13.68) |  | 720(306 to 1,512) |  | 14.54(13.46 to 15.69) |  | 0.47(0.38 to 0.55) |
| Botswana |  | 4,194(2,890 to 6,006) |  | 1316.72(1275.36 to 1359.27) |  | 14,146(9,560 to 20,165) |  | 2066.34(2032.32 to 2100.82) |  | 1.67(1.46 to 1.89) |  | 36(15 to 79) |  | 11.25(7.74 to 16.09) |  | 121(52 to 255) |  | 17.63(14.62 to 21.12) |  | 1.66(1.45 to 1.87) |
| Lesotho |  | 4,502(3,117 to 6,485) |  | 1197.50(1162.35 to 1233.50) |  | 9,093(6,203 to 13,157) |  | 1789.18(1751.90 to 1827.14) |  | 1.25(1.12 to 1.38) |  | 40(17 to 84) |  | 10.47(7.43 to 14.42) |  | 79(34 to 176) |  | 15.48(12.20 to 19.48) |  | 1.24(1.11 to 1.37) |
| Namibia |  | 4,241(2,960 to 5,985) |  | 1263.46(1224.07 to 1303.97) |  | 11,224(7,708 to 16,182) |  | 1693.89(1662.46 to 1725.81) |  | 0.92(0.79 to 1.05) |  | 37(16 to 80) |  | 10.99(7.61 to 15.58) |  | 98(43 to 200) |  | 14.74(11.94 to 18.04) |  | 0.88(0.73 to 1.02) |
| South Africa |  | 169,239(116,857 to 242,007) |  | 1759.86(1751.28 to 1768.48) |  | 351,648(242,006 to 500,909) |  | 2254.18(2246.71 to 2261.67) |  | 0.93(0.85 to 1.00) |  | 1,495(638 to 3,257) |  | 15.42(14.63 to 16.25) |  | 3,047(1,292 to 6,332) |  | 19.57(18.88 to 20.28) |  | 0.89(0.82 to 0.96) |
| Eswatini |  | 3,354(2,302 to 4,829) |  | 1729.44(1668.75 to 1792.08) |  | 6,804(4,644 to 9,653) |  | 2145.53(2094.03 to 2198.14) |  | 0.42(0.17 to 0.68) |  | 30(13 to 61) |  | 15.21(10.04 to 22.57) |  | 60(25 to 121) |  | 18.68(14.18 to 24.38) |  | 0.38(0.13 to 0.63) |
| Zimbabwe |  | 34,719(23,253 to 50,180) |  | 1458.08(1441.94 to 1474.38) |  | 63,881(43,938 to 91,242) |  | 1575.34(1562.96 to 1587.81) |  | -0.12(-0.31 to 0.06) |  | 302(132 to 645) |  | 12.57(11.12 to 14.19) |  | 552(233 to 1,171) |  | 13.55(12.43 to 14.76) |  | -0.15(-0.34 to 0.05) |
| Benin |  | 9,598(6,611 to 13,871) |  | 878.92(860.76 to 897.42) |  | 52,041(35,279 to 74,830) |  | 1608.92(1594.70 to 1623.25) |  | 1.85(1.57 to 2.13) |  | 83(35 to 177) |  | 7.50(5.92 to 9.45) |  | 449(192 to 968) |  | 13.77(12.49 to 15.17) |  | 1.83(1.56 to 2.10) |
| Burkina Faso |  | 17,182(11,659 to 24,926) |  | 828.78(816.12 to 841.61) |  | 72,544(49,724 to 104,581) |  | 1324.59(1314.65 to 1334.60) |  | 1.29(1.08 to 1.50) |  | 153(64 to 329) |  | 7.30(6.16 to 8.61) |  | 632(270 to 1,354) |  | 11.45(10.55 to 12.43) |  | 1.22(1.03 to 1.42) |
| Cameroon |  | 31,217(20,907 to 45,200) |  | 1316.26(1301.16 to 1331.51) |  | 138,280(91,819 to 204,014) |  | 1765.28(1755.74 to 1774.87) |  | 0.74(0.64 to 0.84) |  | 274(115 to 589) |  | 11.45(10.09 to 12.97) |  | 1,223(527 to 2,603) |  | 15.48(14.60 to 16.40) |  | 0.74(0.64 to 0.85) |
| Cabo Verde |  | 752(514 to 1,089) |  | 956.92(885.42 to 1033.62) |  | 2,421(1,663 to 3,544) |  | 1602.44(1538.92 to 1668.09) |  | 1.45(1.23 to 1.67) |  | 7(3 to 14) |  | 8.29(2.98 to 20.18) |  | 21(9 to 44) |  | 13.81(8.51 to 21.43) |  | 1.44(1.23 to 1.66) |
| Chad |  | 8,543(5,765 to 12,322) |  | 652.51(638.34 to 666.94) |  | 37,891(25,898 to 54,736) |  | 992.59(982.17 to 1003.12) |  | 0.90(0.65 to 1.14) |  | 73(31 to 153) |  | 5.55(4.31 to 7.06) |  | 326(139 to 695) |  | 8.47(7.53 to 9.50) |  | 0.87(0.61 to 1.12) |
| Côte d'Ivoire |  | 24,341(16,570 to 35,796) |  | 895.07(883.28 to 907.00) |  | 98,278(67,345 to 145,039) |  | 1474.26(1464.88 to 1483.69) |  | 1.41(1.13 to 1.70) |  | 211(88 to 460) |  | 7.68(6.63 to 8.88) |  | 852(369 to 1,817) |  | 12.71(11.85 to 13.62) |  | 1.42(1.13 to 1.71) |
| Gambia |  | 2,032(1,392 to 2,922) |  | 900.98(859.90 to 943.94) |  | 8,250(5,720 to 12,044) |  | 1344.74(1314.87 to 1375.23) |  | 1.03(0.86 to 1.19) |  | 18(7 to 37) |  | 7.77(4.42 to 13.37) |  | 72(30 to 145) |  | 11.60(8.99 to 14.87) |  | 1.03(0.87 to 1.19) |
| Ghana |  | 32,705(22,544 to 47,234) |  | 932.02(921.62 to 942.51) |  | 127,752(88,182 to 185,211) |  | 1395.60(1387.88 to 1403.35) |  | 0.93(0.68 to 1.18) |  | 283(122 to 610) |  | 8.01(7.07 to 9.05) |  | 1,103(475 to 2,249) |  | 12.01(11.30 to 12.75) |  | 0.88(0.64 to 1.14) |
| Guinea |  | 11,074(7,585 to 15,808) |  | 814.92(799.60 to 830.50) |  | 40,960(28,479 to 58,968) |  | 1235.53(1223.25 to 1247.91) |  | 1.11(0.97 to 1.25) |  | 95(40 to 194) |  | 6.96(5.61 to 8.57) |  | 353(152 to 771) |  | 10.60(9.49 to 11.81) |  | 1.09(0.94 to 1.24) |
| Guinea-Bissau |  | 1,882(1,300 to 2,787) |  | 815.49(777.88 to 854.68) |  | 6,478(4,447 to 9,382) |  | 1233.64(1203.05 to 1264.92) |  | 1.05(0.78 to 1.32) |  | 16(7 to 34) |  | 7.03(3.96 to 11.90) |  | 56(24 to 118) |  | 10.65(7.99 to 14.07) |  | 1.05(0.79 to 1.33) |
| Liberia |  | 5,154(3,513 to 7,536) |  | 932.34(905.92 to 959.46) |  | 18,944(13,004 to 27,792) |  | 1363.87(1344.25 to 1383.73) |  | 1.40(1.27 to 1.52) |  | 45(19 to 96) |  | 8.00(5.74 to 11.04) |  | 161(70 to 335) |  | 11.55(9.82 to 13.53) |  | 1.35(1.24 to 1.46) |
| Mali |  | 13,241(9,025 to 19,043) |  | 696.37(684.29 to 708.63) |  | 60,112(40,623 to 87,123) |  | 1110.53(1101.27 to 1119.85) |  | 1.25(1.04 to 1.47) |  | 116(50 to 255) |  | 6.07(4.99 to 7.34) |  | 523(227 to 1,123) |  | 9.58(8.74 to 10.49) |  | 1.24(1.02 to 1.47) |
| Mauritania |  | 5,345(3,630 to 7,952) |  | 1141.92(1110.55 to 1174.06) |  | 17,217(11,735 to 24,794) |  | 1609.40(1584.86 to 1634.27) |  | 0.76(0.57 to 0.96) |  | 47(20 to 102) |  | 10.00(7.27 to 13.56) |  | 152(65 to 311) |  | 14.07(11.87 to 16.60) |  | 0.76(0.57 to 0.96) |
| Niger |  | 12,550(8,647 to 17,748) |  | 733.07(719.83 to 746.51) |  | 51,986(35,748 to 75,390) |  | 992.13(983.10 to 1001.22) |  | 0.91(0.76 to 1.05) |  | 111(48 to 234) |  | 6.42(5.24 to 7.82) |  | 454(187 to 965) |  | 8.57(7.75 to 9.46) |  | 0.83(0.69 to 0.97) |
| Nigeria |  | 205,802(143,563 to 299,018) |  | 1038.18(1033.45 to 1042.92) |  | 800,540(556,294 to 1,143,886) |  | 1418.26(1415.05 to 1421.47) |  | 0.74(0.56 to 0.91) |  | 1,787(760 to 3,718) |  | 8.92(8.49 to 9.37) |  | 6,997(3,007 to 14,952) |  | 12.28(11.98 to 12.58) |  | 0.76(0.58 to 0.94) |
| Sao Tome and Principe |  | 247(170 to 354) |  | 974.33(850.79 to 1113.07) |  | 809(556 to 1,163) |  | 1447.41(1348.22 to 1552.49) |  | 1.05(0.84 to 1.25) |  | 2(1 to 5) |  | 8.39(0.97 to 36.11) |  | 7(3 to 14) |  | 12.49(4.96 to 26.88) |  | 1.05(0.84 to 1.27) |
| Senegal |  | 17,905(12,347 to 26,370) |  | 1050.25(1034.30 to 1066.41) |  | 51,596(35,437 to 74,502) |  | 1325.73(1314.08 to 1337.47) |  | 0.52(0.39 to 0.64) |  | 158(67 to 347) |  | 9.17(7.74 to 10.82) |  | 453(198 to 968) |  | 11.55(10.49 to 12.70) |  | 0.51(0.39 to 0.64) |
| Sierra Leone |  | 8,061(5,476 to 11,526) |  | 804.79(786.64 to 823.33) |  | 31,032(21,005 to 45,657) |  | 1366.94(1351.24 to 1382.79) |  | 1.53(1.39 to 1.67) |  | 69(30 to 139) |  | 6.86(5.28 to 8.85) |  | 267(114 to 566) |  | 11.66(10.25 to 13.23) |  | 1.51(1.36 to 1.66) |
| Togo |  | 7,097(4,832 to 10,235) |  | 838.45(818.34 to 858.99) |  | 27,663(18,990 to 40,373) |  | 1283.46(1268.28 to 1298.79) |  | 1.09(0.92 to 1.26) |  | 62(26 to 132) |  | 7.27(5.52 to 9.50) |  | 241(102 to 512) |  | 11.14(9.77 to 12.67) |  | 1.10(0.93 to 1.27) |
| American Samoa |  | 465(325 to 654) |  | 3845.00(3493.46 to 4227.25) |  | 615(431 to 896) |  | 5315.55(4899.70 to 5758.69) |  | 0.90(0.72 to 1.08) |  | 4(2 to 9) |  | 33.82(8.98 to 97.88) |  | 5(2 to 11) |  | 46.56(15.69 to 108.97) |  | 0.89(0.71 to 1.07) |
| Bermuda |  | 588(390 to 848) |  | 3415.02(3140.03 to 3710.45) |  | 507(349 to 734) |  | 3800.59(3463.74 to 4164.63) |  | 0.24(0.18 to 0.30) |  | 5(2 to 11) |  | 30.29(9.86 to 75.91) |  | 4(2 to 9) |  | 33.65(9.36 to 91.54) |  | 0.24(0.19 to 0.30) |
| Cook Islands |  | 182(126 to 265) |  | 3966.11(3401.71 to 4605.34) |  | 252(173 to 366) |  | 5892.50(5185.35 to 6672.24) |  | 1.12(0.99 to 1.26) |  | 2(1 to 3) |  | 34.81(2.68 to 164.38) |  | 2(1 to 5) |  | 51.50(7.14 to 183.54) |  | 1.12(0.99 to 1.26) |
| Greenland |  | 358(249 to 503) |  | 2364.63(2119.17 to 2633.77) |  | 404(282 to 574) |  | 3139.38(2839.26 to 3464.13) |  | 0.93(0.84 to 1.02) |  | 3(1 to 7) |  | 21.17(4.44 to 67.91) |  | 4(2 to 8) |  | 28.07(6.92 to 78.52) |  | 0.93(0.85 to 1.02) |
| Guam |  | 1,498(1,027 to 2,129) |  | 4248.25(4032.77 to 4473.90) |  | 2,143(1,470 to 3,058) |  | 5967.62(5716.80 to 6226.84) |  | 1.11(1.02 to 1.20) |  | 13(6 to 27) |  | 37.33(19.78 to 66.78) |  | 19(8 to 39) |  | 52.35(31.36 to 82.39) |  | 1.11(1.01 to 1.20) |
| Monaco |  | 414(285 to 589) |  | 5888.90(5311.83 to 6523.56) |  | 495(342 to 697) |  | 6935.51(6318.16 to 7601.61) |  | 0.48(0.41 to 0.55) |  | 4(2 to 8) |  | 53.09(12.51 to 171.23) |  | 4(2 to 9) |  | 62.20(17.39 to 166.15) |  | 0.46(0.40 to 0.53) |
| Nauru |  | 81(56 to 117) |  | 3332.10(2637.21 to 4176.75) |  | 138(96 to 199) |  | 4827.54(4048.63 to 5723.98) |  | 1.06(0.99 to 1.12) |  | 1(0 to 2) |  | 29.15(0.18 to 267.95) |  | 1(1 to 2) |  | 42.19(1.75 to 236.53) |  | 1.06(0.99 to 1.12) |
| Niue |  | 18(12 to 25) |  | 3632.86(2144.41 to 5789.92) |  | 21(15 to 30) |  | 5514.80(3405.95 to 8478.97) |  | 1.26(1.10 to 1.43) |  | 0(0 to 0) |  | 31.90(0.00 to 890.79) |  | 0(0 to 0) |  | 48.28(0.00 to 1155.36) |  | 1.26(1.10 to 1.42) |
| Northern Mariana Islands |  | 569(392 to 805) |  | 4056.88(3719.69 to 4422.72) |  | 589(408 to 829) |  | 5245.34(4818.79 to 5701.68) |  | 0.66(0.50 to 0.82) |  | 5(2 to 11) |  | 35.62(11.07 to 98.05) |  | 5(2 to 11) |  | 46.00(14.66 to 112.41) |  | 0.66(0.50 to 0.82) |
| Palau |  | 157(109 to 224) |  | 3810.34(3232.42 to 4471.14) |  | 203(140 to 288) |  | 5501.51(4743.58 to 6355.40) |  | 1.04(0.87 to 1.20) |  | 1(1 to 3) |  | 33.37(1.92 to 174.26) |  | 2(1 to 4) |  | 48.13(4.12 to 215.90) |  | 1.03(0.87 to 1.20) |
| Puerto Rico |  | 32,286(21,876 to 46,276) |  | 3371.60(3334.91 to 3408.60) |  | 32,070(21,173 to 47,022) |  | 4303.67(4256.17 to 4351.60) |  | 0.82(0.74 to 0.90) |  | 287(121 to 606) |  | 29.93(26.57 to 33.61) |  | 282(124 to 587) |  | 38.06(33.70 to 42.85) |  | 0.82(0.74 to 0.90) |
| Saint Kitts and Nevis |  | 258(176 to 366) |  | 2559.34(2242.11 to 2919.57) |  | 542(369 to 786) |  | 3476.08(3187.41 to 3785.44) |  | 0.90(0.81 to 0.98) |  | 2(1 to 5) |  | 22.68(2.95 to 104.76) |  | 5(2 to 10) |  | 30.75(9.54 to 76.47) |  | 0.90(0.82 to 0.98) |
| San Marino |  | 352(245 to 502) |  | 5632.10(5057.85 to 6255.58) |  | 475(326 to 668) |  | 6704.95(6093.84 to 7364.75) |  | 0.52(0.43 to 0.60) |  | 3(1 to 7) |  | 50.82(11.11 to 148.48) |  | 4(2 to 9) |  | 60.13(16.13 to 162.72) |  | 0.50(0.42 to 0.59) |
| Tokelau |  | 11(7 to 15) |  | 3044.35(1486.10 to 5700.57) |  | 16(11 to 23) |  | 4988.32(2845.74 to 8156.04) |  | 1.49(1.34 to 1.64) |  | 0(0 to 0) |  | 26.70(0.00 to 1476.44) |  | 0(0 to 0) |  | 43.62(0.00 to 1345.43) |  | 1.48(1.34 to 1.63) |
| Tuvalu |  | 69(48 to 98) |  | 2841.96(2212.16 to 3601.47) |  | 131(90 to 186) |  | 4502.03(3758.97 to 5355.28) |  | 1.26(1.08 to 1.44) |  | 1(0 to 1) |  | 24.90(0.08 to 214.98) |  | 1(1 to 2) |  | 39.38(1.44 to 216.72) |  | 1.28(1.11 to 1.46) |
| United States Virgin Islands |  | 933(628 to 1,372) |  | 3268.25(3060.62 to 3486.77) |  | 703(470 to 1,007) |  | 4128.47(3823.20 to 4453.24) |  | 0.71(0.61 to 0.82) |  | 8(4 to 18) |  | 29.05(12.68 to 57.70) |  | 6(3 to 13) |  | 36.50(13.32 to 82.55) |  | 0.70(0.60 to 0.81) |
| South Sudan |  | 12,988(8,915 to 18,872) |  | 1011.32(993.08 to 1029.87) |  | 25,758(17,596 to 37,296) |  | 1124.47(1110.44 to 1138.65) |  | 0.18(0.07 to 0.29) |  | 114(47 to 251) |  | 8.78(7.16 to 10.72) |  | 225(97 to 480) |  | 9.76(8.50 to 11.18) |  | 0.20(0.09 to 0.30) |
| Sudan |  | 91,335(61,611 to 129,729) |  | 1958.08(1944.99 to 1971.24) |  | 393,149(272,888 to 559,786) |  | 3477.76(3466.73 to 3488.82) |  | 1.95(1.80 to 2.11) |  | 805(357 to 1,764) |  | 17.08(15.88 to 18.35) |  | 3,406(1,529 to 7,111) |  | 29.99(28.97 to 31.03) |  | 1.93(1.76 to 2.09) |

**Table S4:** Prevalence and DALYs cases and age-standardised rate of unexplained infertility for WCBA in 1990 and 2021, and their average annual percentage change from 1990 to 2021 by countries and territories.

| **Location** |  | **Prevalence** | | | | | | | | |  | **DALYs (Disability-Adjusted Life Years)** | | | | | | | | |
| --- | --- | --- | --- | --- | --- | --- | --- | --- | --- | --- | --- | --- | --- | --- | --- | --- | --- | --- | --- | --- |
|  |  | **Number of cases, 1990** |  | **Age-standardised** rate, 1990 |  | **Number of cases, 2021** |  | **Age-standardised** rate, 2021 |  | AAPC, 1990–2021 |  | **Number of cases, 1990** |  | **Age-standardised** rate, 1990 |  | **Number of cases, 2021** |  | **Age-standardised** rate, 2021 |  | AAPC, 1990–2021 |
| China |  | 24,796,281(13,332,614 to 43,051,674) |  | 8030.43(8027.24 to 8033.62) |  | 29,317,000(14,569,167 to 52,098,692) |  | 8374.49(8371.41 to 8377.57) |  | 0.01(-0.04 to 0.05) |  | 129,476(42,981 to 332,624) |  | 41.81(41.58 to 42.04) |  | 153,252(50,580 to 396,547) |  | 43.99(43.77 to 44.22) |  | 0.02(-0.03 to 0.07) |
| Democratic People's Republic of Korea |  | 313,727(164,951 to 577,644) |  | 6065.96(6044.59 to 6087.39) |  | 410,661(201,844 to 776,717) |  | 6133.59(6114.83 to 6152.39) |  | -0.05(-0.08 to -0.02) |  | 1,651(540 to 3,967) |  | 31.78(30.26 to 33.37) |  | 2,149(676 to 5,064) |  | 32.12(30.77 to 33.51) |  | -0.04(-0.08 to -0.01) |
| Cambodia |  | 110,425(54,160 to 203,265) |  | 4758.41(4730.04 to 4786.92) |  | 220,334(102,200 to 415,498) |  | 4753.43(4733.55 to 4773.38) |  | -2.47(-3.46 to -1.48) |  | 589(194 to 1,484) |  | 25.25(23.23 to 27.42) |  | 1,172(367 to 2,926) |  | 25.26(23.83 to 26.76) |  | -2.32(-3.27 to -1.37) |
| Indonesia |  | 2,407,857(1,269,212 to 4,344,202) |  | 5388.88(5381.99 to 5395.79) |  | 6,251,542(3,293,414 to 11,133,561) |  | 8127.14(8120.76 to 8133.52) |  | 1.67(1.07 to 2.28) |  | 13,403(4,585 to 34,320) |  | 29.75(29.25 to 30.27) |  | 34,094(11,773 to 82,100) |  | 44.39(43.91 to 44.86) |  | 1.58(1.01 to 2.16) |
| Lao People's Democratic Republic |  | 37,395(18,559 to 67,849) |  | 4301.29(4257.23 to 4345.73) |  | 78,106(34,643 to 147,647) |  | 3972.06(3944.15 to 4000.13) |  | -0.19(-0.21 to -0.17) |  | 205(67 to 541) |  | 23.47(20.33 to 26.99) |  | 428(124 to 1,100) |  | 21.70(19.68 to 23.87) |  | -0.19(-0.21 to -0.17) |
| Malaysia |  | 220,384(104,273 to 413,416) |  | 5102.42(5080.99 to 5123.92) |  | 407,011(162,381 to 812,003) |  | 4698.62(4684.18 to 4713.10) |  | -0.27(-0.31 to -0.23) |  | 1,171(364 to 2,825) |  | 27.03(25.50 to 28.65) |  | 2,151(600 to 5,596) |  | 24.82(23.78 to 25.89) |  | -0.28(-0.31 to -0.25) |
| Maldives |  | 3,993(2,370 to 6,627) |  | 9852.73(9535.61 to 10178.76) |  | 9,189(7,308 to 11,222) |  | 7024.63(6880.41 to 7171.53) |  | -1.68(-2.25 to -1.11) |  | 22(8 to 52) |  | 54.23(33.28 to 85.08) |  | 52(21 to 107) |  | 39.99(29.77 to 53.14) |  | -1.54(-2.11 to -0.97) |
| Myanmar |  | 435,807(200,894 to 855,418) |  | 4472.84(4459.37 to 4486.36) |  | 296,755(201,457 to 389,740) |  | 2018.01(2010.75 to 2025.28) |  | -3.51(-4.13 to -2.89) |  | 2,403(776 to 6,190) |  | 24.49(23.51 to 25.51) |  | 1,650(620 to 3,742) |  | 11.22(10.68 to 11.77) |  | -3.44(-4.05 to -2.82) |
| Philippines |  | 1,129,720(365,873 to 2,255,613) |  | 7719.34(7704.96 to 7733.75) |  | 2,499,071(851,174 to 4,963,466) |  | 8643.49(8632.75 to 8654.24) |  | 6.57(3.69 to 9.53) |  | 6,320(1,550 to 16,817) |  | 43.00(41.94 to 44.09) |  | 14,334(3,812 to 35,154) |  | 49.48(48.67 to 50.29) |  | 6.52(3.71 to 9.41) |
| Sri Lanka |  | 76,404(48,566 to 104,562) |  | 1662.25(1650.45 to 1674.13) |  | 183,588(67,464 to 366,837) |  | 3175.54(3160.98 to 3190.16) |  | 0.46(-0.09 to 1.01) |  | 443(166 to 988) |  | 9.60(8.73 to 10.55) |  | 997(261 to 2,544) |  | 17.29(16.23 to 18.40) |  | 0.39(-0.11 to 0.90) |
| Thailand |  | 278,904(180,810 to 388,674) |  | 1715.17(1708.77 to 1721.59) |  | 571,872(217,570 to 1,103,625) |  | 3394.21(3385.29 to 3403.14) |  | 0.64(0.11 to 1.17) |  | 1,688(625 to 3,694) |  | 10.35(9.86 to 10.86) |  | 3,169(825 to 8,056) |  | 18.89(18.23 to 19.58) |  | 0.53(0.05 to 1.01) |
| Timor-Leste |  | 5,685(2,877 to 10,146) |  | 3205.77(3121.73 to 3291.71) |  | 8,939(4,102 to 16,409) |  | 3039.85(2975.87 to 3104.91) |  | -0.43(-1.05 to 0.19) |  | 32(10 to 75) |  | 17.69(11.99 to 25.47) |  | 50(15 to 120) |  | 16.81(12.39 to 22.38) |  | -0.42(-1.03 to 0.19) |
| Viet Nam |  | 309,760(120,047 to 642,381) |  | 1964.66(1957.50 to 1971.85) |  | 449,459(113,365 to 985,469) |  | 1596.84(1592.16 to 1601.53) |  | 2.07(1.12 to 3.03) |  | 1,694(461 to 4,509) |  | 10.67(10.15 to 11.21) |  | 2,427(475 to 6,870) |  | 8.65(8.31 to 9.00) |  | 2.01(1.07 to 2.95) |
| Fiji |  | 12,643(6,730 to 21,305) |  | 6731.28(6613.55 to 6850.72) |  | 15,293(8,060 to 26,195) |  | 6609.50(6505.13 to 6715.16) |  | -0.06(-0.08 to -0.04) |  | 71(24 to 166) |  | 37.40(29.13 to 47.47) |  | 85(28 to 205) |  | 36.67(29.28 to 45.38) |  | -0.07(-0.09 to -0.04) |
| Kiribati |  | 802(400 to 1,422) |  | 4528.51(4215.16 to 4861.30) |  | 1,388(632 to 2,564) |  | 4378.23(4150.59 to 4615.90) |  | -0.05(-0.08 to -0.02) |  | 4(1 to 11) |  | 24.66(7.07 to 66.28) |  | 8(2 to 19) |  | 23.86(10.00 to 49.29) |  | -0.05(-0.07 to -0.02) |
| Marshall Islands |  | 400(198 to 695) |  | 4516.15(4075.64 to 5002.19) |  | 634(294 to 1,154) |  | 4346.43(4014.51 to 4699.14) |  | -0.12(-0.14 to -0.10) |  | 2(1 to 6) |  | 24.72(3.27 to 112.68) |  | 3(1 to 9) |  | 23.72(5.66 to 66.17) |  | -0.12(-0.15 to -0.10) |
| Micronesia (Federated States of) |  | 904(438 to 1,638) |  | 4365.08(4080.05 to 4667.63) |  | 1,040(480 to 1,889) |  | 4306.44(4047.73 to 4577.62) |  | -0.01(-0.04 to 0.01) |  | 5(2 to 12) |  | 23.87(7.53 to 62.45) |  | 6(2 to 14) |  | 23.51(8.31 to 52.84) |  | -0.01(-0.03 to 0.01) |
| Papua New Guinea |  | 20,948(7,171 to 42,203) |  | 2333.56(2301.55 to 2365.96) |  | 37,489(20,998 to 55,295) |  | 1385.00(1370.98 to 1399.14) |  | -1.96(-2.51 to -1.40) |  | 114(28 to 310) |  | 12.63(10.38 to 15.27) |  | 208(67 to 519) |  | 7.69(6.68 to 8.82) |  | -1.86(-2.38 to -1.32) |
| Samoa |  | 1,395(676 to 2,526) |  | 4455.34(4219.63 to 4701.66) |  | 1,945(846 to 3,639) |  | 4349.17(4156.91 to 4548.30) |  | -0.07(-0.08 to -0.05) |  | 8(2 to 20) |  | 24.41(10.10 to 50.98) |  | 11(3 to 26) |  | 23.76(11.66 to 43.49) |  | -0.08(-0.09 to -0.06) |
| Solomon Islands |  | 2,686(1,267 to 4,754) |  | 4250.32(4086.89 to 4419.16) |  | 6,866(3,178 to 12,522) |  | 4217.50(4118.09 to 4318.77) |  | 0.01(-0.04 to 0.05) |  | 15(5 to 38) |  | 23.30(12.77 to 39.91) |  | 38(11 to 96) |  | 23.11(16.31 to 31.88) |  | 0.01(-0.03 to 0.06) |
| Tonga |  | 848(401 to 1,515) |  | 4447.09(4148.94 to 4761.66) |  | 1,010(425 to 1,854) |  | 4263.82(4004.40 to 4535.81) |  | -0.10(-0.12 to -0.08) |  | 5(1 to 12) |  | 24.26(7.32 to 60.20) |  | 6(2 to 14) |  | 23.21(8.04 to 52.54) |  | -0.10(-0.12 to -0.08) |
| Vanuatu |  | 1,444(714 to 2,558) |  | 4486.33(4253.39 to 4730.14) |  | 3,164(1,491 to 5,694) |  | 4262.78(4114.77 to 4414.95) |  | -0.15(-0.17 to -0.13) |  | 8(3 to 20) |  | 24.60(10.39 to 51.76) |  | 17(6 to 43) |  | 23.37(13.67 to 37.60) |  | -0.14(-0.17 to -0.12) |
| Armenia |  | 32,530(12,072 to 71,636) |  | 3353.23(3316.46 to 3390.38) |  | 7,690(4,384 to 11,654) |  | 1002.11(979.23 to 1025.47) |  | -2.70(-3.31 to -2.08) |  | 175(49 to 486) |  | 18.06(15.46 to 21.07) |  | 41(14 to 95) |  | 5.41(3.84 to 7.50) |  | -2.76(-3.34 to -2.18) |
| Azerbaijan |  | 88,756(40,088 to 170,343) |  | 4348.83(4319.46 to 4378.40) |  | 135,501(56,369 to 276,168) |  | 4514.67(4490.40 to 4539.06) |  | -0.39(-1.69 to 0.92) |  | 497(162 to 1,257) |  | 24.18(22.04 to 26.51) |  | 747(235 to 1,999) |  | 25.06(23.27 to 26.96) |  | -0.38(-1.62 to 0.87) |
| Georgia |  | 51,074(21,761 to 101,328) |  | 3529.30(3498.65 to 3560.16) |  | 26,627(9,016 to 57,764) |  | 3261.14(3221.50 to 3301.22) |  | -0.34(-0.44 to -0.24) |  | 279(85 to 749) |  | 19.29(17.09 to 21.72) |  | 144(37 to 372) |  | 17.80(14.97 to 21.08) |  | -0.35(-0.45 to -0.26) |
| Kazakhstan |  | 90,988(60,703 to 125,323) |  | 2060.09(2046.65 to 2073.61) |  | 175,036(68,958 to 370,779) |  | 3514.48(3497.86 to 3531.16) |  | 3.27(2.52 to 4.02) |  | 519(193 to 1,119) |  | 11.76(10.76 to 12.83) |  | 955(268 to 2,394) |  | 19.30(18.08 to 20.59) |  | 3.13(2.40 to 3.86) |
| Kyrgyzstan |  | 49,173(24,412 to 93,539) |  | 4610.82(4569.31 to 4652.70) |  | 85,510(40,458 to 163,876) |  | 4800.98(4768.73 to 4833.40) |  | 0.63(-0.01 to 1.28) |  | 268(86 to 658) |  | 24.98(22.02 to 28.32) |  | 462(152 to 1,133) |  | 25.94(23.62 to 28.43) |  | 0.63(0.01 to 1.25) |
| Mongolia |  | 15,841(7,049 to 30,575) |  | 3179.51(3128.30 to 3231.49) |  | 31,156(12,719 to 62,694) |  | 3498.95(3459.96 to 3538.30) |  | 0.34(0.28 to 0.40) |  | 87(26 to 207) |  | 17.30(13.73 to 21.67) |  | 169(50 to 444) |  | 19.07(16.28 to 22.24) |  | 0.36(0.30 to 0.41) |
| Tajikistan |  | 31,059(13,268 to 61,491) |  | 2594.87(2564.85 to 2625.23) |  | 33,510(21,602 to 46,127) |  | 1201.06(1188.21 to 1214.02) |  | -2.80(-3.29 to -2.30) |  | 174(50 to 462) |  | 14.37(12.23 to 16.89) |  | 194(72 to 443) |  | 6.96(6.02 to 8.03) |  | -2.63(-3.09 to -2.16) |
| Turkmenistan |  | 31,417(13,355 to 61,762) |  | 3484.87(3445.14 to 3525.04) |  | 46,457(20,257 to 92,568) |  | 3681.71(3648.29 to 3715.37) |  | 0.28(0.23 to 0.33) |  | 173(54 to 444) |  | 19.01(16.19 to 22.30) |  | 253(76 to 643) |  | 20.00(17.61 to 22.63) |  | 0.26(0.22 to 0.31) |
| Uzbekistan |  | 125,076(52,599 to 253,376) |  | 2546.73(2531.98 to 2561.56) |  | 256,625(98,734 to 509,274) |  | 2728.47(2717.89 to 2739.08) |  | 2.04(1.33 to 2.76) |  | 689(199 to 1,829) |  | 13.90(12.83 to 15.05) |  | 1,389(407 to 3,749) |  | 14.81(14.04 to 15.62) |  | 1.89(1.23 to 2.55) |
| Albania |  | 26,289(11,321 to 52,247) |  | 3098.49(3060.50 to 3136.89) |  | 12,163(8,866 to 15,886) |  | 1970.86(1935.97 to 2006.25) |  | -1.48(-2.60 to -0.35) |  | 147(44 to 377) |  | 17.15(14.44 to 20.29) |  | 68(26 to 139) |  | 11.01(8.55 to 14.02) |  | -1.43(-2.52 to -0.34) |
| Bosnia and Herzegovina |  | 45,685(19,694 to 92,358) |  | 3771.69(3737.16 to 3806.49) |  | 28,239(11,725 to 59,026) |  | 3864.43(3819.00 to 3910.32) |  | 0.16(0.13 to 0.20) |  | 246(71 to 625) |  | 20.31(17.85 to 23.03) |  | 151(43 to 389) |  | 20.70(17.49 to 24.39) |  | 0.15(0.12 to 0.19) |
| Bulgaria |  | 80,026(34,437 to 167,535) |  | 3872.04(3845.07 to 3899.14) |  | 56,192(22,571 to 120,187) |  | 3907.11(3873.96 to 3940.50) |  | 0.03(0.01 to 0.06) |  | 426(126 to 1,107) |  | 20.71(18.78 to 22.79) |  | 297(84 to 747) |  | 20.85(18.48 to 23.48) |  | 0.03(0.01 to 0.06) |
| Croatia |  | 50,252(20,463 to 105,732) |  | 4000.85(3965.84 to 4036.12) |  | 35,645(14,306 to 77,357) |  | 3920.96(3879.93 to 3962.37) |  | -0.15(-0.24 to -0.06) |  | 267(79 to 694) |  | 21.34(18.85 to 24.09) |  | 190(54 to 471) |  | 20.99(18.08 to 24.29) |  | -0.14(-0.23 to -0.06) |
| Czechia |  | 141,914(64,001 to 276,184) |  | 5606.93(5577.43 to 5636.55) |  | 128,037(58,522 to 245,085) |  | 5694.15(5662.13 to 5726.33) |  | 1.44(0.89 to 2.00) |  | 753(237 to 1,967) |  | 29.88(27.76 to 32.13) |  | 677(210 to 1,769) |  | 30.34(28.03 to 32.81) |  | 1.40(0.87 to 1.94) |
| Hungary |  | 96,756(39,450 to 202,488) |  | 3755.22(3731.27 to 3779.30) |  | 80,012(33,659 to 166,650) |  | 3879.23(3851.91 to 3906.72) |  | 0.01(-0.03 to 0.05) |  | 515(146 to 1,372) |  | 20.12(18.39 to 21.97) |  | 426(125 to 1,080) |  | 20.77(18.81 to 22.90) |  | 0.02(-0.02 to 0.06) |
| North Macedonia |  | 19,855(8,584 to 40,158) |  | 3856.51(3803.04 to 3910.55) |  | 21,438(8,983 to 45,206) |  | 3850.37(3798.48 to 3902.89) |  | 0.07(0.05 to 0.09) |  | 106(32 to 269) |  | 20.69(16.95 to 25.03) |  | 114(34 to 291) |  | 20.61(16.97 to 24.94) |  | 0.07(0.05 to 0.09) |
| Montenegro |  | 6,098(2,590 to 12,375) |  | 3813.62(3718.47 to 3910.66) |  | 5,381(2,228 to 11,408) |  | 3741.02(3640.97 to 3843.32) |  | -0.01(-0.02 to 0.01) |  | 33(10 to 82) |  | 20.51(14.10 to 28.95) |  | 29(8 to 75) |  | 20.06(13.36 to 29.21) |  | -0.01(-0.03 to 0.01) |
| Poland |  | 463,163(220,651 to 868,422) |  | 4702.78(4689.05 to 4716.54) |  | 657,230(324,241 to 1,192,629) |  | 7059.43(7041.91 to 7077.00) |  | 1.39(0.96 to 1.81) |  | 2,563(883 to 6,526) |  | 26.28(25.26 to 27.33) |  | 3,522(1,145 to 8,861) |  | 38.22(36.94 to 39.55) |  | 1.29(0.90 to 1.69) |
| Romania |  | 205,725(86,505 to 430,902) |  | 3638.89(3623.05 to 3654.79) |  | 150,446(61,535 to 319,646) |  | 3819.78(3800.11 to 3839.53) |  | 0.25(0.14 to 0.35) |  | 1,101(315 to 2,795) |  | 19.53(18.38 to 20.74) |  | 799(227 to 2,108) |  | 20.44(19.02 to 21.94) |  | 0.24(0.14 to 0.34) |
| Serbia |  | 94,605(39,369 to 191,609) |  | 3970.08(3944.78 to 3995.51) |  | 78,591(31,617 to 161,867) |  | 3840.68(3813.63 to 3867.89) |  | -0.10(-0.16 to -0.04) |  | 507(151 to 1,293) |  | 21.32(19.51 to 23.27) |  | 419(122 to 1,094) |  | 20.57(18.63 to 22.67) |  | -0.10(-0.16 to -0.04) |
| Slovakia |  | 55,096(23,186 to 112,103) |  | 3986.67(3953.31 to 4020.26) |  | 50,680(21,085 to 109,855) |  | 3864.96(3830.64 to 3899.56) |  | -0.24(-0.33 to -0.15) |  | 294(84 to 748) |  | 21.35(18.97 to 23.97) |  | 269(80 to 695) |  | 20.70(18.25 to 23.45) |  | -0.22(-0.30 to -0.13) |
| Slovenia |  | 4,786(2,518 to 7,919) |  | 926.42(900.31 to 953.14) |  | 11,502(3,547 to 26,046) |  | 2675.13(2625.38 to 2725.71) |  | 4.81(3.86 to 5.78) |  | 25(8 to 61) |  | 4.94(3.21 to 7.33) |  | 60(13 to 165) |  | 13.98(10.60 to 18.28) |  | 4.71(3.77 to 5.65) |
| Belarus |  | 134,609(63,029 to 266,142) |  | 4959.13(4932.56 to 4985.81) |  | 115,515(50,429 to 227,190) |  | 5184.65(5153.73 to 5215.73) |  | 0.26(0.21 to 0.30) |  | 750(256 to 1,962) |  | 27.74(25.78 to 29.81) |  | 634(197 to 1,667) |  | 28.87(26.58 to 31.32) |  | 0.23(0.19 to 0.27) |
| Estonia |  | 18,687(8,706 to 35,175) |  | 4755.38(4687.11 to 4824.45) |  | 15,140(6,954 to 28,747) |  | 5283.29(5197.56 to 5370.26) |  | 0.34(0.29 to 0.38) |  | 104(34 to 271) |  | 26.69(21.79 to 32.42) |  | 83(28 to 216) |  | 29.30(23.20 to 36.72) |  | 0.30(0.26 to 0.34) |
| Latvia |  | 19,496(13,697 to 26,872) |  | 2951.77(2910.39 to 2993.63) |  | 21,562(9,796 to 41,262) |  | 5410.90(5337.07 to 5485.63) |  | 2.72(2.21 to 3.23) |  | 109(41 to 237) |  | 16.62(13.64 to 20.09) |  | 117(37 to 301) |  | 29.73(24.46 to 35.97) |  | 2.62(2.13 to 3.12) |
| Lithuania |  | 29,996(21,831 to 39,602) |  | 3217.52(3181.16 to 3254.21) |  | 32,232(15,148 to 62,109) |  | 5553.03(5492.22 to 5614.45) |  | 2.37(1.91 to 2.83) |  | 166(65 to 360) |  | 17.83(15.22 to 20.78) |  | 174(56 to 455) |  | 30.17(25.83 to 35.14) |  | 2.29(1.85 to 2.74) |
| Republic of Moldova |  | 63,163(29,809 to 116,963) |  | 5235.74(5194.67 to 5277.08) |  | 52,438(24,238 to 98,104) |  | 5555.96(5506.94 to 5605.39) |  | 1.43(0.30 to 2.57) |  | 353(118 to 881) |  | 29.47(26.45 to 32.76) |  | 290(95 to 741) |  | 31.15(27.55 to 35.19) |  | 1.33(0.29 to 2.40) |
| Russian Federation |  | 2,933,150(1,474,553 to 5,377,012) |  | 7215.43(7207.11 to 7223.76) |  | 2,715,415(1,370,335 to 5,019,099) |  | 7717.36(7707.79 to 7726.93) |  | 0.26(0.25 to 0.28) |  | 16,265(5,667 to 41,244) |  | 40.30(39.68 to 40.93) |  | 14,835(5,113 to 38,356) |  | 42.80(42.08 to 43.53) |  | 0.24(0.22 to 0.26) |
| Ukraine |  | 809,764(389,637 to 1,478,286) |  | 6169.00(6155.51 to 6182.51) |  | 688,457(337,132 to 1,308,650) |  | 6667.96(6651.60 to 6684.36) |  | 1.88(1.24 to 2.52) |  | 4,654(1,510 to 12,045) |  | 35.68(34.66 to 36.73) |  | 3,872(1,321 to 10,213) |  | 38.12(36.88 to 39.40) |  | 1.80(1.18 to 2.43) |
| Brunei Darussalam |  | 457(50 to 1,460) |  | 803.87(730.44 to 883.62) |  | 952(109 to 3,398) |  | 703.09(659.12 to 749.55) |  | -0.46(-0.74 to -0.18) |  | 2(0 to 10) |  | 4.34(0.68 to 16.37) |  | 5(0 to 22) |  | 3.80(1.26 to 9.42) |  | -0.48(-0.76 to -0.21) |
| Japan |  | 558,713(46,524 to 1,691,595) |  | 1491.38(1487.40 to 1495.36) |  | 424,422(37,954 to 1,273,566) |  | 1455.13(1450.70 to 1459.58) |  | -0.36(-0.66 to -0.05) |  | 2,982(176 to 11,537) |  | 7.99(7.70 to 8.29) |  | 2,258(142 to 8,592) |  | 7.77(7.45 to 8.10) |  | -0.36(-0.66 to -0.06) |
| Republic of Korea |  | 91,666(9,522 to 278,416) |  | 826.45(821.07 to 831.86) |  | 112,151(10,787 to 388,446) |  | 792.58(787.89 to 797.29) |  | -0.14(-0.34 to 0.06) |  | 500(40 to 2,148) |  | 4.49(4.10 to 4.90) |  | 602(41 to 2,520) |  | 4.27(3.94 to 4.64) |  | -0.16(-0.36 to 0.03) |
| Singapore |  | 7,222(757 to 24,084) |  | 781.35(763.39 to 799.65) |  | 18,299(1,566 to 64,247) |  | 864.78(852.04 to 877.76) |  | 0.07(-0.22 to 0.36) |  | 39(3 to 178) |  | 4.24(3.01 to 5.83) |  | 98(6 to 396) |  | 4.66(3.76 to 5.85) |  | 0.05(-0.24 to 0.34) |
| Australia |  | 12,712(3,245 to 55,439) |  | 270.88(266.18 to 275.64) |  | 16,214(4,511 to 69,965) |  | 249.15(245.31 to 253.05) |  | -0.19(-0.37 to -0.02) |  | 72(11 to 307) |  | 1.54(1.20 to 1.94) |  | 93(16 to 412) |  | 1.43(1.15 to 1.76) |  | -0.19(-0.38 to -0.01) |
| New Zealand |  | 2,257(669 to 9,882) |  | 244.04(234.07 to 254.33) |  | 7,732(1,326 to 29,990) |  | 622.79(608.97 to 636.88) |  | 4.31(3.45 to 5.18) |  | 13(2 to 58) |  | 1.39(0.74 to 2.40) |  | 43(5 to 180) |  | 3.47(2.51 to 4.71) |  | 4.22(3.39 to 5.07) |
| Andorra |  | 367(119 to 789) |  | 2288.86(2059.92 to 2539.19) |  | 495(139 to 1,018) |  | 2142.72(1952.54 to 2350.63) |  | -0.23(-0.30 to -0.16) |  | 2(0 to 6) |  | 13.08(1.69 to 52.82) |  | 3(1 to 8) |  | 12.18(2.13 to 47.71) |  | -0.24(-0.31 to -0.17) |
| Austria |  | 110,559(48,360 to 206,627) |  | 5567.76(5534.93 to 5600.75) |  | 126,855(56,158 to 232,867) |  | 5877.34(5844.89 to 5909.97) |  | 1.06(0.73 to 1.39) |  | 617(178 to 1,644) |  | 31.02(28.61 to 33.58) |  | 697(218 to 1,691) |  | 32.47(30.09 to 35.02) |  | 1.14(0.76 to 1.51) |
| Belgium |  | 67,208(42,407 to 98,399) |  | 2527.63(2508.54 to 2546.85) |  | 135,219(58,389 to 247,336) |  | 5047.97(5020.95 to 5075.11) |  | 2.42(1.87 to 2.97) |  | 382(134 to 859) |  | 14.41(13.00 to 15.94) |  | 750(218 to 1,844) |  | 28.16(26.17 to 30.28) |  | 2.35(1.81 to 2.89) |
| Cyprus |  | 4,918(1,756 to 9,774) |  | 2419.26(2352.08 to 2487.94) |  | 9,763(2,994 to 20,762) |  | 2230.99(2186.31 to 2276.65) |  | -0.49(-0.67 to -0.30) |  | 28(7 to 74) |  | 13.90(9.26 to 20.18) |  | 55(13 to 159) |  | 12.73(9.54 to 17.05) |  | -0.51(-0.68 to -0.33) |
| Denmark |  | 3,214(575 to 8,518) |  | 227.69(219.83 to 235.77) |  | 8,206(718 to 22,725) |  | 641.28(627.43 to 655.38) |  | 3.66(2.48 to 4.86) |  | 20(2 to 64) |  | 1.39(0.84 to 2.20) |  | 49(4 to 161) |  | 3.87(2.86 to 5.14) |  | 3.63(2.45 to 4.82) |
| Finland |  | 7,561(2,427 to 13,645) |  | 494.25(483.05 to 505.69) |  | 21,050(5,178 to 45,662) |  | 1679.45(1656.73 to 1702.43) |  | 1.30(0.49 to 2.12) |  | 44(9 to 109) |  | 2.89(2.09 to 3.96) |  | 120(22 to 348) |  | 9.61(7.96 to 11.55) |  | 1.29(0.48 to 2.10) |
| France |  | 361,687(235,601 to 511,815) |  | 2348.36(2340.70 to 2356.05) |  | 625,853(259,355 to 1,114,550) |  | 4236.42(4225.85 to 4247.01) |  | 2.05(1.44 to 2.67) |  | 2,197(820 to 4,915) |  | 14.33(13.74 to 14.94) |  | 3,662(1,064 to 9,306) |  | 25.02(24.21 to 25.85) |  | 1.94(1.36 to 2.54) |
| Germany |  | 60,647(11,329 to 143,417) |  | 299.95(297.57 to 302.35) |  | 220,847(38,085 to 558,906) |  | 1138.65(1133.88 to 1143.44) |  | 4.70(3.27 to 6.15) |  | 366(57 to 1,057) |  | 1.81(1.63 to 2.01) |  | 1,244(166 to 4,044) |  | 6.46(6.10 to 6.83) |  | 4.48(3.10 to 5.88) |
| Greece |  | 59,092(18,759 to 123,216) |  | 2297.55(2279.05 to 2316.17) |  | 51,813(11,610 to 113,114) |  | 2069.82(2051.53 to 2088.26) |  | -0.39(-0.51 to -0.26) |  | 336(77 to 908) |  | 13.09(11.73 to 14.58) |  | 288(52 to 799) |  | 11.67(10.32 to 13.17) |  | -0.42(-0.55 to -0.30) |
| Iceland |  | 1,525(507 to 3,150) |  | 2322.59(2207.34 to 2442.58) |  | 1,956(557 to 4,219) |  | 2319.10(2217.27 to 2424.80) |  | -0.12(-0.25 to 0.01) |  | 9(2 to 23) |  | 13.31(6.00 to 25.97) |  | 11(3 to 31) |  | 13.14(6.56 to 24.17) |  | -0.15(-0.28 to -0.02) |
| Ireland |  | 20,508(6,927 to 42,799) |  | 2336.09(2304.21 to 2368.30) |  | 30,327(9,074 to 64,436) |  | 2286.62(2260.40 to 2313.11) |  | -0.05(-0.15 to 0.04) |  | 117(28 to 319) |  | 13.34(11.03 to 16.00) |  | 169(38 to 470) |  | 12.87(10.96 to 15.05) |  | -0.09(-0.18 to -0.00) |
| Israel |  | 19,073(4,846 to 40,456) |  | 1515.01(1493.56 to 1536.72) |  | 39,816(9,916 to 90,331) |  | 1758.11(1740.85 to 1775.50) |  | 0.28(0.06 to 0.51) |  | 108(19 to 288) |  | 8.57(7.03 to 10.39) |  | 223(42 to 653) |  | 9.88(8.62 to 11.27) |  | 0.26(0.03 to 0.49) |
| Italy |  | 163,799(18,145 to 570,937) |  | 1133.31(1127.81 to 1138.82) |  | 281,020(34,316 to 762,230) |  | 2194.22(2185.98 to 2202.50) |  | 2.78(2.32 to 3.25) |  | 904(81 to 3,322) |  | 6.26(5.86 to 6.68) |  | 1,545(168 to 5,227) |  | 12.16(11.55 to 12.80) |  | 2.81(2.34 to 3.28) |
| Luxembourg |  | 2,238(606 to 4,771) |  | 2105.87(2019.46 to 2195.67) |  | 3,592(905 to 8,029) |  | 2017.94(1952.05 to 2085.97) |  | -0.04(-0.23 to 0.16) |  | 13(3 to 34) |  | 12.09(6.40 to 21.85) |  | 20(4 to 57) |  | 11.44(6.98 to 18.44) |  | -0.07(-0.26 to 0.12) |
| Malta |  | 2,581(895 to 5,104) |  | 2412.76(2319.60 to 2509.11) |  | 2,516(762 to 5,322) |  | 2315.91(2225.56 to 2409.82) |  | -0.23(-0.46 to -0.00) |  | 15(4 to 38) |  | 13.82(7.61 to 23.67) |  | 14(3 to 38) |  | 13.08(7.12 to 23.40) |  | -0.27(-0.50 to -0.04) |
| Netherlands |  | 99,447(32,635 to 195,790) |  | 2347.44(2332.85 to 2362.11) |  | 88,185(25,914 to 189,032) |  | 2328.13(2312.75 to 2343.58) |  | 0.03(-0.02 to 0.08) |  | 569(141 to 1,480) |  | 13.46(12.37 to 14.62) |  | 499(121 to 1,365) |  | 13.23(12.09 to 14.45) |  | 0.00(-0.04 to 0.05) |
| Norway |  | 39,291(13,514 to 79,732) |  | 3568.29(3533.01 to 3603.85) |  | 46,117(14,503 to 93,940) |  | 3643.23(3609.97 to 3676.75) |  | -0.13(-0.28 to 0.01) |  | 223(57 to 612) |  | 20.35(17.76 to 23.23) |  | 261(63 to 700) |  | 20.69(18.25 to 23.41) |  | -0.13(-0.27 to 0.00) |
| Portugal |  | 59,052(21,257 to 114,422) |  | 2319.23(2300.54 to 2338.03) |  | 55,230(15,934 to 118,061) |  | 2112.04(2093.99 to 2130.23) |  | -0.24(-0.32 to -0.17) |  | 338(86 to 873) |  | 13.28(11.90 to 14.78) |  | 309(73 to 821) |  | 11.98(10.65 to 13.46) |  | -0.26(-0.33 to -0.20) |
| Spain |  | 38,792(12,010 to 75,358) |  | 413.61(409.50 to 417.75) |  | 136,108(18,766 to 358,903) |  | 1156.27(1149.94 to 1162.64) |  | 4.91(3.84 to 6.00) |  | 226(50 to 592) |  | 2.41(2.10 to 2.74) |  | 753(76 to 2,350) |  | 6.46(5.99 to 6.96) |  | 4.73(3.69 to 5.78) |
| Sweden |  | 91,065(39,463 to 172,604) |  | 4253.62(4225.82 to 4281.57) |  | 100,556(38,852 to 194,729) |  | 4308.65(4281.93 to 4335.51) |  | -0.03(-0.06 to 0.01) |  | 517(160 to 1,366) |  | 24.32(22.25 to 26.54) |  | 565(158 to 1,543) |  | 24.40(22.42 to 26.53) |  | -0.04(-0.07 to -0.02) |
| Switzerland |  | 39,788(12,582 to 84,583) |  | 2102.77(2082.12 to 2123.60) |  | 49,304(13,913 to 107,443) |  | 2208.92(2189.30 to 2228.70) |  | 0.19(0.04 to 0.33) |  | 227(51 to 597) |  | 12.05(10.53 to 13.76) |  | 277(62 to 797) |  | 12.52(11.08 to 14.14) |  | 0.16(0.02 to 0.30) |
| United Kingdom |  | 471,759(146,487 to 970,946) |  | 3196.36(3187.21 to 3205.52) |  | 455,080(93,421 to 1,045,815) |  | 2770.86(2762.79 to 2778.95) |  | -0.08(-0.27 to 0.11) |  | 2,695(686 to 7,380) |  | 18.26(17.58 to 18.97) |  | 2,572(445 to 7,815) |  | 15.76(15.16 to 16.39) |  | -0.09(-0.28 to 0.10) |
| Argentina |  | 185,370(48,725 to 402,069) |  | 2347.51(2336.83 to 2358.22) |  | 261,639(63,094 to 579,074) |  | 2128.18(2120.02 to 2136.36) |  | -0.29(-0.38 to -0.20) |  | 1,037(209 to 3,117) |  | 13.12(12.34 to 13.95) |  | 1,465(248 to 4,055) |  | 11.94(11.34 to 12.57) |  | -0.29(-0.38 to -0.20) |
| Chile |  | 79,750(21,536 to 162,842) |  | 2282.23(2266.33 to 2298.22) |  | 106,501(24,098 to 238,349) |  | 2167.32(2154.31 to 2180.39) |  | -0.17(-0.29 to -0.04) |  | 450(89 to 1,279) |  | 12.77(11.61 to 14.02) |  | 590(102 to 1,740) |  | 12.02(11.07 to 13.04) |  | -0.21(-0.33 to -0.08) |
| Uruguay |  | 16,911(4,391 to 36,285) |  | 2267.35(2233.30 to 2301.79) |  | 18,948(4,254 to 41,382) |  | 2204.48(2173.11 to 2236.20) |  | -0.15(-0.25 to -0.05) |  | 95(19 to 272) |  | 12.77(10.33 to 15.61) |  | 105(18 to 310) |  | 12.28(10.04 to 14.90) |  | -0.19(-0.28 to -0.09) |
| Canada |  | 82,459(8,233 to 209,987) |  | 1029.62(1022.58 to 1036.70) |  | 81,972(8,231 to 216,141) |  | 954.59(948.03 to 961.20) |  | -0.19(-0.24 to -0.14) |  | 488(41 to 1,448) |  | 6.12(5.58 to 6.69) |  | 482(38 to 1,589) |  | 5.64(5.15 to 6.18) |  | -0.21(-0.26 to -0.16) |
| United States of America |  | 756,311(72,613 to 2,042,293) |  | 1041.07(1038.72 to 1043.42) |  | 1,396,795(208,173 to 3,438,035) |  | 1801.03(1798.04 to 1804.03) |  | 3.30(1.67 to 4.96) |  | 4,488(360 to 14,329) |  | 6.19(6.01 to 6.38) |  | 8,025(976 to 25,097) |  | 10.38(10.16 to 10.61) |  | 3.19(1.56 to 4.84) |
| Antigua and Barbuda |  | 953(498 to 1,697) |  | 5811.68(5446.10 to 6198.03) |  | 1,447(740 to 2,618) |  | 5833.59(5536.04 to 6144.30) |  | 0.05(0.03 to 0.07) |  | 5(2 to 14) |  | 32.18(10.79 to 79.04) |  | 8(3 to 21) |  | 32.28(13.88 to 66.07) |  | 0.04(0.02 to 0.06) |
| Bahamas |  | 3,980(2,088 to 7,232) |  | 5579.14(5404.87 to 5758.11) |  | 6,013(3,031 to 11,082) |  | 5611.07(5469.95 to 5754.99) |  | 0.06(0.05 to 0.08) |  | 22(7 to 55) |  | 30.94(19.31 to 47.78) |  | 33(11 to 84) |  | 31.05(21.39 to 43.68) |  | 0.06(0.04 to 0.07) |
| Barbados |  | 4,129(2,103 to 7,447) |  | 5815.00(5638.20 to 5996.38) |  | 4,146(2,113 to 7,506) |  | 5761.16(5586.28 to 5940.52) |  | 0.02(-0.00 to 0.04) |  | 23(8 to 59) |  | 32.16(20.31 to 49.05) |  | 23(8 to 59) |  | 31.85(20.10 to 48.51) |  | 0.02(-0.00 to 0.04) |
| Belize |  | 749(449 to 1,045) |  | 1855.19(1720.90 to 1999.18) |  | 3,321(1,311 to 6,349) |  | 2835.83(2739.91 to 2934.35) |  | 1.65(1.19 to 2.11) |  | 4(2 to 10) |  | 10.82(3.06 to 31.49) |  | 19(5 to 47) |  | 16.04(9.62 to 25.27) |  | 1.54(1.11 to 1.97) |
| Cuba |  | 179,439(97,159 to 312,534) |  | 6132.31(6103.68 to 6161.04) |  | 144,318(74,438 to 263,135) |  | 5849.15(5818.92 to 5879.51) |  | -0.07(-0.14 to 0.01) |  | 993(338 to 2,484) |  | 33.68(31.60 to 35.87) |  | 792(265 to 2,049) |  | 32.21(29.99 to 34.55) |  | -0.06(-0.13 to 0.01) |
| Dominica |  | 882(460 to 1,578) |  | 5679.10(5305.24 to 6073.68) |  | 929(474 to 1,730) |  | 5666.31(5307.50 to 6043.37) |  | 0.02(0.00 to 0.03) |  | 5(2 to 13) |  | 31.53(9.94 to 77.46) |  | 5(2 to 13) |  | 31.33(10.35 to 73.10) |  | 0.01(-0.00 to 0.03) |
| Dominican Republic |  | 33,420(23,074 to 45,312) |  | 1862.62(1842.27 to 1883.17) |  | 139,681(63,324 to 270,113) |  | 4817.41(4792.15 to 4842.77) |  | 1.98(0.70 to 3.28) |  | 195(75 to 428) |  | 10.80(9.31 to 12.50) |  | 763(242 to 2,010) |  | 26.26(24.43 to 28.20) |  | 1.89(0.66 to 3.13) |
| Grenada |  | 1,059(567 to 1,934) |  | 5745.99(5398.92 to 6112.18) |  | 1,439(743 to 2,680) |  | 5798.34(5501.51 to 6107.47) |  | 0.03(0.01 to 0.05) |  | 6(2 to 15) |  | 31.86(11.32 to 75.83) |  | 8(3 to 20) |  | 32.03(13.76 to 64.09) |  | 0.03(0.01 to 0.05) |
| Guyana |  | 15,384(8,839 to 27,011) |  | 7972.18(7844.29 to 8101.91) |  | 15,888(8,842 to 27,705) |  | 8025.75(7900.55 to 8152.50) |  | -0.43(-1.51 to 0.66) |  | 85(30 to 212) |  | 43.69(34.74 to 54.58) |  | 87(30 to 222) |  | 43.79(35.03 to 54.17) |  | -0.41(-1.42 to 0.62) |
| Haiti |  | 55,675(42,493 to 70,111) |  | 3857.97(3825.52 to 3890.66) |  | 113,714(84,161 to 142,353) |  | 3150.18(3131.88 to 3168.56) |  | -0.19(-0.40 to 0.03) |  | 313(125 to 655) |  | 21.45(19.10 to 24.03) |  | 619(239 to 1,340) |  | 17.15(15.83 to 18.57) |  | -0.24(-0.45 to -0.03) |
| Jamaica |  | 38,282(21,079 to 65,928) |  | 6864.63(6794.38 to 6935.51) |  | 54,434(29,719 to 95,285) |  | 6887.15(6829.33 to 6945.37) |  | 0.03(0.01 to 0.06) |  | 214(72 to 517) |  | 37.94(32.91 to 43.62) |  | 301(101 to 748) |  | 37.99(33.81 to 42.56) |  | 0.02(0.00 to 0.04) |
| Saint Lucia |  | 1,778(937 to 3,250) |  | 5636.42(5371.67 to 5912.30) |  | 2,726(1,358 to 5,025) |  | 5834.79(5617.07 to 6059.50) |  | 0.11(0.09 to 0.13) |  | 10(3 to 25) |  | 31.21(14.64 to 60.84) |  | 15(5 to 38) |  | 32.17(17.96 to 54.25) |  | 0.10(0.08 to 0.12) |
| Saint Vincent and the Grenadines |  | 1,390(741 to 2,463) |  | 5759.74(5452.34 to 6082.36) |  | 1,608(800 to 2,958) |  | 5783.71(5503.83 to 6074.47) |  | -0.03(-0.05 to -0.01) |  | 8(3 to 19) |  | 31.92(13.19 to 69.23) |  | 9(3 to 23) |  | 31.91(14.46 to 61.52) |  | -0.03(-0.06 to -0.01) |
| Suriname |  | 5,254(2,812 to 9,227) |  | 5807.48(5649.20 to 5969.26) |  | 8,403(4,131 to 15,027) |  | 5775.85(5652.95 to 5900.81) |  | -0.03(-0.05 to -0.01) |  | 29(10 to 75) |  | 32.07(21.38 to 46.49) |  | 46(15 to 121) |  | 31.79(23.28 to 42.45) |  | -0.03(-0.05 to -0.02) |
| Trinidad and Tobago |  | 13,496(10,166 to 17,283) |  | 4387.68(4313.53 to 4462.90) |  | 26,027(13,452 to 46,485) |  | 7110.50(7023.12 to 7198.78) |  | 0.60(0.26 to 0.94) |  | 78(31 to 168) |  | 25.33(20.00 to 31.77) |  | 143(48 to 368) |  | 39.29(33.01 to 46.51) |  | 0.55(0.24 to 0.87) |
| Bolivia (Plurinational State of) |  | 4,043(1,328 to 9,024) |  | 309.56(300.04 to 319.32) |  | 24,344(2,796 to 79,116) |  | 782.76(772.94 to 792.67) |  | 6.70(3.78 to 9.70) |  | 22(5 to 58) |  | 1.66(1.03 to 2.55) |  | 132(10 to 510) |  | 4.23(3.54 to 5.02) |  | 6.65(3.77 to 9.62) |
| Ecuador |  | 3,744(2,047 to 10,205) |  | 158.48(153.36 to 163.74) |  | 34,628(4,370 to 118,287) |  | 737.09(729.34 to 744.90) |  | 9.33(7.19 to 11.51) |  | 21(6 to 66) |  | 0.87(0.53 to 1.37) |  | 187(15 to 732) |  | 3.98(3.43 to 4.60) |  | 9.15(7.05 to 11.29) |
| Peru |  | 3,859(3,017 to 6,779) |  | 80.49(77.94 to 83.12) |  | 121,873(10,651 to 372,425) |  | 1227.72(1220.84 to 1234.64) |  | 7.12(5.46 to 8.81) |  | 21(8 to 51) |  | 0.44(0.27 to 0.68) |  | 651(48 to 2,457) |  | 6.56(6.06 to 7.08) |  | 7.08(5.47 to 8.72) |
| Colombia |  | 27,186(7,279 to 58,827) |  | 338.04(333.98 to 342.13) |  | 58,268(13,249 to 111,088) |  | 430.77(427.28 to 434.29) |  | 1.23(0.57 to 1.90) |  | 158(28 to 452) |  | 1.95(1.65 to 2.28) |  | 345(62 to 920) |  | 2.55(2.29 to 2.84) |  | 1.35(0.69 to 2.00) |
| Costa Rica |  | 28,171(12,182 to 56,549) |  | 3686.15(3642.70 to 3730.06) |  | 49,838(20,850 to 100,255) |  | 3619.29(3587.56 to 3651.26) |  | -0.06(-0.08 to -0.04) |  | 154(46 to 437) |  | 20.06(16.98 to 23.62) |  | 269(74 to 740) |  | 19.54(17.27 to 22.05) |  | -0.08(-0.10 to -0.05) |
| El Salvador |  | 11,016(4,797 to 18,959) |  | 944.15(926.32 to 962.26) |  | 26,372(4,332 to 64,834) |  | 1514.85(1496.56 to 1533.31) |  | 5.16(2.85 to 7.52) |  | 62(17 to 153) |  | 5.26(4.01 to 6.80) |  | 143(19 to 427) |  | 8.17(6.88 to 9.64) |  | 5.07(2.78 to 7.40) |
| Guatemala |  | 31,469(21,459 to 42,611) |  | 2056.67(2033.80 to 2079.75) |  | 133,608(51,054 to 283,637) |  | 3212.24(3194.95 to 3229.61) |  | -1.27(-2.49 to -0.03) |  | 166(61 to 377) |  | 10.83(9.23 to 12.65) |  | 706(193 to 1,920) |  | 16.91(15.67 to 18.21) |  | -1.13(-2.32 to 0.08) |
| Honduras |  | 32,559(13,656 to 67,266) |  | 3440.64(3402.79 to 3478.84) |  | 95,108(38,407 to 188,330) |  | 3492.79(3470.50 to 3515.20) |  | 0.56(-1.63 to 2.80) |  | 174(51 to 467) |  | 18.30(15.64 to 21.33) |  | 505(143 to 1,393) |  | 18.48(16.90 to 20.19) |  | 0.57(-1.57 to 2.75) |
| Mexico |  | 312,139(77,172 to 706,727) |  | 1710.32(1704.25 to 1716.41) |  | 1,614,547(684,946 to 3,192,541) |  | 4584.91(4577.84 to 4591.99) |  | 1.49(0.81 to 2.17) |  | 1,685(307 to 5,153) |  | 9.17(8.73 to 9.62) |  | 8,537(2,505 to 22,628) |  | 24.25(23.74 to 24.77) |  | 1.47(0.79 to 2.15) |
| Nicaragua |  | 14,620(3,851 to 33,155) |  | 1816.66(1786.72 to 1847.05) |  | 32,873(8,145 to 74,925) |  | 1797.90(1778.50 to 1817.47) |  | 3.92(1.89 to 5.99) |  | 80(16 to 228) |  | 9.84(7.76 to 12.39) |  | 178(33 to 530) |  | 9.73(8.35 to 11.28) |  | 3.83(1.86 to 5.84) |
| Panama |  | 25,402(12,085 to 49,682) |  | 4434.31(4379.40 to 4489.80) |  | 45,747(19,728 to 90,415) |  | 4325.89(4286.31 to 4365.73) |  | -0.06(-0.09 to -0.04) |  | 136(42 to 351) |  | 23.59(19.75 to 28.02) |  | 242(73 to 633) |  | 22.89(20.10 to 25.97) |  | -0.07(-0.10 to -0.05) |
| Venezuela (Bolivarian Republic of) |  | 137,420(52,863 to 276,578) |  | 2940.63(2924.96 to 2956.38) |  | 213,844(77,431 to 440,384) |  | 2984.76(2972.00 to 2997.57) |  | 0.13(0.08 to 0.19) |  | 753(202 to 2,139) |  | 16.02(14.88 to 17.23) |  | 1,152(286 to 3,090) |  | 16.17(15.24 to 17.14) |  | 0.12(0.07 to 0.17) |
| Brazil |  | 1,019,804(523,587 to 1,840,146) |  | 2710.57(2705.27 to 2715.87) |  | 2,125,447(887,052 to 4,170,154) |  | 3389.50(3384.93 to 3394.08) |  | 1.70(1.06 to 2.35) |  | 5,862(2,035 to 15,345) |  | 15.49(15.09 to 15.89) |  | 11,672(3,616 to 31,644) |  | 18.69(18.35 to 19.03) |  | 1.62(0.98 to 2.26) |
| Paraguay |  | 22,650(17,257 to 29,549) |  | 2763.79(2727.40 to 2800.56) |  | 90,276(48,834 to 157,990) |  | 4792.58(4761.32 to 4824.00) |  | 1.95(1.41 to 2.49) |  | 126(48 to 262) |  | 15.28(12.69 to 18.27) |  | 495(169 to 1,243) |  | 26.20(23.94 to 28.63) |  | 1.91(1.39 to 2.44) |
| Algeria |  | 97,705(49,202 to 169,050) |  | 1754.98(1743.51 to 1766.51) |  | 411,463(180,229 to 736,498) |  | 3456.43(3445.82 to 3467.08) |  | 2.87(2.52 to 3.23) |  | 575(181 to 1,416) |  | 10.18(9.33 to 11.10) |  | 2,282(642 to 5,900) |  | 19.28(18.50 to 20.10) |  | 2.75(2.39 to 3.10) |
| Bahrain |  | 4,263(1,899 to 7,401) |  | 3732.60(3616.36 to 3852.44) |  | 12,863(5,953 to 22,979) |  | 3789.84(3724.49 to 3856.13) |  | 0.06(0.03 to 0.09) |  | 24(7 to 62) |  | 20.91(13.13 to 32.91) |  | 72(21 to 183) |  | 21.19(16.56 to 26.79) |  | 0.06(0.03 to 0.09) |
| Egypt |  | 258,429(201,960 to 313,104) |  | 2057.60(2049.59 to 2065.63) |  | 989,412(442,270 to 1,672,172) |  | 3803.81(3796.31 to 3811.32) |  | 1.36(0.71 to 2.03) |  | 1,590(628 to 3,464) |  | 12.52(11.90 to 13.16) |  | 5,698(1,652 to 13,987) |  | 21.84(21.28 to 22.42) |  | 1.13(0.51 to 1.76) |
| Iran (Islamic Republic of) |  | 419,255(195,272 to 751,833) |  | 3407.78(3397.20 to 3418.39) |  | 815,742(367,319 to 1,472,829) |  | 3319.80(3312.38 to 3327.24) |  | -0.77(-1.67 to 0.15) |  | 2,650(823 to 6,798) |  | 21.20(20.38 to 22.05) |  | 4,978(1,502 to 14,117) |  | 20.62(20.03 to 21.22) |  | -0.68(-1.58 to 0.23) |
| Iraq |  | 137,112(65,470 to 241,760) |  | 3704.01(3683.89 to 3724.22) |  | 385,454(183,787 to 661,420) |  | 3810.76(3798.68 to 3822.87) |  | 0.12(0.10 to 0.14) |  | 781(221 to 1,784) |  | 20.78(19.31 to 22.35) |  | 2,166(681 to 5,349) |  | 21.28(20.39 to 22.20) |  | 0.11(0.09 to 0.14) |
| Jordan |  | 10,482(4,838 to 18,435) |  | 1312.01(1285.47 to 1339.02) |  | 99,625(74,090 to 127,976) |  | 3348.80(3327.99 to 3369.72) |  | 2.33(1.92 to 2.74) |  | 63(20 to 161) |  | 7.70(5.81 to 10.09) |  | 558(214 to 1,229) |  | 18.69(17.17 to 20.31) |  | 2.10(1.70 to 2.51) |
| Kuwait |  | 15,769(7,297 to 27,281) |  | 3750.71(3691.10 to 3811.26) |  | 62,231(27,749 to 113,601) |  | 3656.27(3626.19 to 3686.61) |  | -0.04(-0.09 to -0.00) |  | 89(26 to 219) |  | 21.05(16.83 to 26.31) |  | 342(97 to 861) |  | 20.42(18.20 to 22.91) |  | -0.07(-0.11 to -0.03) |
| Lebanon |  | 38,269(20,678 to 65,575) |  | 5299.65(5246.24 to 5353.48) |  | 86,054(42,323 to 153,793) |  | 5436.71(5399.89 to 5473.75) |  | 1.94(1.29 to 2.59) |  | 212(67 to 517) |  | 29.17(25.34 to 33.43) |  | 468(144 to 1,127) |  | 29.73(27.05 to 32.62) |  | 1.92(1.27 to 2.57) |
| Libya |  | 15,921(7,537 to 26,999) |  | 1904.80(1873.83 to 1936.21) |  | 69,077(30,788 to 125,656) |  | 3353.37(3328.31 to 3378.59) |  | 2.41(1.93 to 2.89) |  | 93(29 to 225) |  | 10.94(8.73 to 13.62) |  | 389(109 to 1,006) |  | 18.97(17.12 to 20.97) |  | 2.36(1.88 to 2.83) |
| Morocco |  | 123,558(87,531 to 163,202) |  | 2176.52(2164.22 to 2188.88) |  | 430,242(216,983 to 739,243) |  | 4383.16(4370.07 to 4396.29) |  | 3.88(2.63 to 5.14) |  | 729(288 to 1,579) |  | 12.69(11.77 to 13.67) |  | 2,461(749 to 6,122) |  | 25.13(24.14 to 26.14) |  | 3.85(2.59 to 5.11) |
| Palestine |  | 15,166(7,312 to 25,932) |  | 3871.98(3807.84 to 3937.03) |  | 48,573(23,815 to 84,700) |  | 3953.37(3917.79 to 3989.22) |  | 0.07(0.05 to 0.09) |  | 87(27 to 202) |  | 21.67(17.16 to 27.13) |  | 274(80 to 642) |  | 22.05(19.48 to 24.89) |  | 0.07(0.04 to 0.09) |
| Oman |  | 13,062(6,525 to 21,951) |  | 4092.45(4021.11 to 4164.89) |  | 43,442(19,846 to 76,751) |  | 3910.79(3873.62 to 3948.25) |  | -0.19(-0.22 to -0.17) |  | 74(23 to 172) |  | 22.87(17.85 to 29.05) |  | 240(69 to 588) |  | 21.77(19.07 to 24.79) |  | -0.20(-0.22 to -0.18) |
| Qatar |  | 3,177(1,427 to 5,715) |  | 3776.50(3644.12 to 3913.63) |  | 24,658(10,605 to 45,057) |  | 3818.90(3768.63 to 3869.84) |  | 0.09(0.06 to 0.12) |  | 18(5 to 44) |  | 21.13(12.34 to 35.67) |  | 136(38 to 333) |  | 21.27(17.64 to 25.63) |  | 0.08(0.05 to 0.11) |
| Saudi Arabia |  | 117,024(54,834 to 198,912) |  | 3900.14(3877.26 to 3923.14) |  | 432,939(194,918 to 767,797) |  | 3865.43(3853.81 to 3877.09) |  | -0.02(-0.04 to 0.01) |  | 663(203 to 1,615) |  | 21.79(20.12 to 23.59) |  | 2,393(684 to 5,944) |  | 21.52(20.66 to 22.41) |  | -0.03(-0.05 to -0.01) |
| Syrian Arab Republic |  | 50,362(24,342 to 80,517) |  | 2084.05(2065.21 to 2103.05) |  | 131,615(59,933 to 235,991) |  | 3518.08(3498.16 to 3538.10) |  | 2.55(1.96 to 3.15) |  | 284(92 to 683) |  | 11.54(10.19 to 13.05) |  | 725(209 to 1,806) |  | 19.38(17.93 to 20.94) |  | 2.55(1.94 to 3.17) |
| Tunisia |  | 39,275(25,578 to 56,834) |  | 1980.16(1960.13 to 2000.37) |  | 119,052(56,105 to 220,149) |  | 3636.91(3616.01 to 3657.91) |  | 2.30(2.02 to 2.59) |  | 240(91 to 539) |  | 11.92(10.42 to 13.60) |  | 670(197 to 1,660) |  | 20.68(19.12 to 22.34) |  | 2.25(1.98 to 2.52) |
| Türkiye |  | 183,539(97,070 to 279,690) |  | 1244.70(1238.94 to 1250.49) |  | 811,791(352,581 to 1,511,078) |  | 3641.63(3633.68 to 3649.60) |  | 0.69(-0.40 to 1.79) |  | 1,147(416 to 2,517) |  | 7.72(7.28 to 8.19) |  | 4,511(1,290 to 11,630) |  | 20.31(19.72 to 20.92) |  | 0.47(-0.56 to 1.51) |
| United Arab Emirates |  | 14,249(6,579 to 24,275) |  | 4056.80(3988.38 to 4126.48) |  | 87,699(38,397 to 164,375) |  | 3935.86(3905.39 to 3966.58) |  | -0.08(-0.10 to -0.07) |  | 80(25 to 195) |  | 22.60(17.78 to 28.83) |  | 473(126 to 1,165) |  | 21.80(19.55 to 24.30) |  | -0.09(-0.11 to -0.08) |
| Yemen |  | 110,259(58,928 to 187,374) |  | 4188.76(4163.44 to 4214.23) |  | 428,501(231,588 to 717,519) |  | 5273.09(5257.22 to 5289.01) |  | 0.39(-0.43 to 1.21) |  | 636(200 to 1,571) |  | 23.90(22.03 to 25.90) |  | 2,409(784 to 5,939) |  | 29.48(28.31 to 30.70) |  | 0.34(-0.50 to 1.18) |
| Afghanistan |  | 52,221(25,099 to 93,820) |  | 2783.40(2758.41 to 2808.59) |  | 103,774(65,690 to 142,851) |  | 1592.02(1581.90 to 1602.18) |  | -2.50(-2.95 to -2.05) |  | 278(81 to 686) |  | 14.70(12.94 to 16.67) |  | 567(205 to 1,284) |  | 8.58(7.86 to 9.36) |  | -2.38(-2.81 to -1.96) |
| Bangladesh |  | 435,322(313,130 to 582,556) |  | 1586.17(1581.33 to 1591.01) |  | 1,506,087(1,190,413 to 1,810,954) |  | 3133.65(3128.64 to 3138.66) |  | 2.96(2.55 to 3.38) |  | 2,617(1,031 to 5,443) |  | 9.42(9.05 to 9.80) |  | 8,613(3,349 to 17,060) |  | 17.90(17.52 to 18.28) |  | 2.78(2.40 to 3.17) |
| Bhutan |  | 3,992(1,824 to 7,236) |  | 2791.22(2702.65 to 2882.46) |  | 6,049(2,539 to 11,297) |  | 2794.34(2724.27 to 2865.88) |  | -0.02(-0.04 to 0.00) |  | 23(7 to 56) |  | 15.87(9.89 to 24.92) |  | 34(10 to 83) |  | 15.92(11.05 to 22.39) |  | -0.01(-0.04 to 0.01) |
| India |  | 8,702,433(4,330,615 to 15,264,114) |  | 4417.94(4414.99 to 4420.90) |  | 29,075,289(16,070,794 to 49,483,699) |  | 7614.03(7611.26 to 7616.80) |  | 2.53(1.82 to 3.23) |  | 49,709(15,752 to 118,620) |  | 24.97(24.75 to 25.19) |  | 161,474(57,797 to 392,596) |  | 42.24(42.04 to 42.45) |  | 2.39(1.73 to 3.06) |
| Nepal |  | 159,018(72,212 to 295,491) |  | 3409.96(3393.01 to 3426.98) |  | 150,430(109,902 to 194,827) |  | 1536.90(1529.12 to 1544.71) |  | -0.47(-1.87 to 0.94) |  | 908(275 to 2,250) |  | 19.38(18.12 to 20.71) |  | 891(344 to 1,880) |  | 9.08(8.49 to 9.70) |  | -0.50(-1.81 to 0.83) |
| Pakistan |  | 1,813,531(583,633 to 3,850,438) |  | 8185.30(8173.22 to 8197.40) |  | 4,817,403(1,352,930 to 10,740,041) |  | 7834.12(7827.10 to 7841.15) |  | -5.60(-10.23 to -0.72) |  | 10,403(2,354 to 27,797) |  | 46.35(45.45 to 47.26) |  | 28,463(5,747 to 79,955) |  | 46.08(45.55 to 46.63) |  | -5.46(-10.10 to -0.58) |
| Angola |  | 150,739(79,881 to 275,189) |  | 7270.84(7233.42 to 7308.41) |  | 533,943(287,662 to 941,956) |  | 7712.55(7691.65 to 7733.50) |  | 0.24(0.21 to 0.27) |  | 809(270 to 2,034) |  | 38.62(35.95 to 41.46) |  | 2,854(982 to 6,963) |  | 40.90(39.39 to 42.45) |  | 0.26(0.23 to 0.29) |
| Central African Republic |  | 54,151(43,986 to 65,804) |  | 9505.56(9423.78 to 9587.93) |  | 148,169(90,817 to 237,833) |  | 11621.61(11561.84 to 11681.64) |  | 0.70(0.46 to 0.94) |  | 298(120 to 625) |  | 51.58(45.75 to 58.00) |  | 817(303 to 1,882) |  | 63.45(59.12 to 68.04) |  | 0.74(0.50 to 0.97) |
| Congo |  | 28,217(13,550 to 52,094) |  | 5883.04(5812.76 to 5954.02) |  | 87,057(41,048 to 161,490) |  | 6274.82(6233.17 to 6316.69) |  | -0.52(-1.31 to 0.28) |  | 148(47 to 366) |  | 30.60(25.75 to 36.19) |  | 453(135 to 1,181) |  | 32.60(29.67 to 35.76) |  | -0.54(-1.32 to 0.26) |
| Democratic Republic of the Congo |  | 333,015(151,242 to 632,710) |  | 4414.35(4399.04 to 4429.70) |  | 862,739(409,645 to 1,644,018) |  | 4525.62(4515.94 to 4535.31) |  | -0.80(-2.42 to 0.84) |  | 1,769(517 to 4,355) |  | 23.20(22.11 to 24.34) |  | 4,624(1,375 to 11,278) |  | 24.04(23.34 to 24.76) |  | -0.73(-2.32 to 0.88) |
| Equatorial Guinea |  | 6,560(3,538 to 11,504) |  | 7443.46(7261.24 to 7629.43) |  | 26,891(14,679 to 47,461) |  | 7902.09(7806.90 to 7998.28) |  | 0.27(0.22 to 0.33) |  | 35(12 to 86) |  | 39.39(27.25 to 55.56) |  | 143(48 to 344) |  | 41.75(35.12 to 49.42) |  | 0.28(0.22 to 0.33) |
| Gabon |  | 19,309(10,801 to 33,007) |  | 10109.18(9960.31 to 10260.01) |  | 46,817(26,562 to 77,282) |  | 10249.42(10156.03 to 10343.51) |  | 0.28(-0.22 to 0.78) |  | 104(35 to 257) |  | 53.79(43.50 to 66.15) |  | 251(85 to 620) |  | 54.53(47.93 to 61.83) |  | 0.32(-0.18 to 0.83) |
| Burundi |  | 26,435(18,326 to 36,329) |  | 2407.09(2377.46 to 2437.04) |  | 20,729(11,395 to 31,123) |  | 695.09(685.53 to 704.78) |  | -4.49(-5.68 to -3.28) |  | 143(56 to 315) |  | 12.84(10.77 to 15.24) |  | 114(39 to 279) |  | 3.79(3.11 to 4.59) |  | -4.42(-5.56 to -3.28) |
| Comoros |  | 8,907(5,092 to 14,341) |  | 9198.50(9003.92 to 9396.60) |  | 17,948(10,106 to 28,707) |  | 9423.35(9285.50 to 9562.79) |  | 1.02(0.44 to 1.60) |  | 51(18 to 124) |  | 52.02(38.45 to 69.36) |  | 102(36 to 242) |  | 53.33(43.45 to 64.84) |  | 1.14(0.54 to 1.75) |
| Djibouti |  | 9,440(5,551 to 14,989) |  | 10580.44(10362.88 to 10801.99) |  | 36,059(20,121 to 58,772) |  | 10718.40(10607.89 to 10829.84) |  | 0.39(-0.20 to 0.98) |  | 51(18 to 120) |  | 56.61(41.81 to 75.71) |  | 193(68 to 471) |  | 57.38(49.55 to 66.17) |  | 0.39(-0.20 to 0.99) |
| Eritrea |  | 39,352(31,488 to 47,385) |  | 5171.83(5119.74 to 5224.36) |  | 142,798(79,663 to 237,797) |  | 8931.56(8884.96 to 8978.36) |  | 2.27(2.01 to 2.54) |  | 222(91 to 460) |  | 28.81(25.07 to 33.01) |  | 783(276 to 1,807) |  | 48.68(45.31 to 52.26) |  | 2.18(1.93 to 2.43) |
| Ethiopia |  | 625,159(318,796 to 1,118,052) |  | 5849.31(5834.47 to 5864.17) |  | 992,154(476,708 to 1,804,689) |  | 3840.96(3833.28 to 3848.65) |  | -0.56(-0.90 to -0.23) |  | 3,433(1,157 to 8,500) |  | 31.73(30.65 to 32.84) |  | 5,419(1,812 to 12,945) |  | 20.75(20.19 to 21.32) |  | -0.58(-0.92 to -0.24) |
| Kenya |  | 139,189(64,567 to 264,098) |  | 3147.82(3130.68 to 3165.04) |  | 617,522(314,620 to 1,122,348) |  | 4965.15(4952.62 to 4977.70) |  | -3.88(-5.66 to -2.06) |  | 755(243 to 1,808) |  | 16.88(15.65 to 18.19) |  | 3,309(1,112 to 8,149) |  | 26.41(25.50 to 27.34) |  | -3.89(-5.67 to -2.07) |
| Madagascar |  | 86,375(67,438 to 108,494) |  | 3499.30(3475.41 to 3523.33) |  | 355,631(185,469 to 597,454) |  | 5227.13(5209.71 to 5244.60) |  | 1.27(0.38 to 2.18) |  | 487(202 to 1,034) |  | 19.55(17.81 to 21.44) |  | 2,003(688 to 4,801) |  | 29.12(27.84 to 30.45) |  | 1.31(0.44 to 2.17) |
| Malawi |  | 128,751(103,379 to 155,926) |  | 6380.50(6344.70 to 6416.48) |  | 41,567(25,927 to 57,043) |  | 790.97(783.29 to 798.72) |  | -6.20(-6.67 to -5.74) |  | 688(281 to 1,486) |  | 33.59(31.06 to 36.31) |  | 224(82 to 524) |  | 4.25(3.70 to 4.87) |  | -6.18(-6.64 to -5.71) |
| Mauritius |  | 12,657(5,703 to 24,145) |  | 4168.85(4096.07 to 4242.73) |  | 12,586(4,867 to 24,531) |  | 3862.18(3794.60 to 3930.73) |  | -0.25(-0.27 to -0.22) |  | 69(21 to 175) |  | 22.54(17.49 to 28.77) |  | 68(19 to 172) |  | 20.77(16.09 to 26.48) |  | -0.26(-0.29 to -0.24) |
| Mozambique |  | 233,412(126,012 to 401,852) |  | 7874.79(7842.59 to 7907.11) |  | 574,163(323,719 to 930,604) |  | 8404.38(8382.10 to 8426.72) |  | -1.13(-1.94 to -0.32) |  | 1,258(438 to 3,053) |  | 42.15(39.83 to 44.58) |  | 3,094(1,102 to 7,419) |  | 44.70(43.09 to 46.35) |  | -1.09(-1.86 to -0.30) |
| Rwanda |  | 7,754(2,637 to 15,263) |  | 481.15(470.25 to 492.29) |  | 46,497(11,842 to 108,671) |  | 1350.40(1338.08 to 1362.81) |  | -0.61(-1.98 to 0.77) |  | 44(11 to 116) |  | 2.69(1.94 to 3.70) |  | 253(48 to 703) |  | 7.32(6.44 to 8.30) |  | -0.71(-2.09 to 0.69) |
| Seychelles |  | 683(301 to 1,296) |  | 4197.45(3878.46 to 4538.13) |  | 941(376 to 1,867) |  | 3788.43(3549.34 to 4040.20) |  | -0.26(-0.28 to -0.24) |  | 4(1 to 10) |  | 22.79(5.50 to 67.17) |  | 5(1 to 13) |  | 20.57(6.73 to 49.27) |  | -0.26(-0.28 to -0.23) |
| Somalia |  | 75,362(36,191 to 132,753) |  | 4623.18(4590.06 to 4656.51) |  | 214,543(108,124 to 381,213) |  | 4850.66(4829.86 to 4871.54) |  | 0.16(0.12 to 0.19) |  | 406(127 to 979) |  | 24.80(22.44 to 27.38) |  | 1,169(385 to 2,849) |  | 26.17(24.67 to 27.75) |  | 0.18(0.14 to 0.21) |
| United Republic of Tanzania |  | 315,713(265,741 to 369,311) |  | 6305.32(6282.64 to 6328.07) |  | 501,885(417,743 to 597,445) |  | 3638.99(3628.80 to 3649.21) |  | -2.16(-2.32 to -2.00) |  | 1,653(678 to 3,379) |  | 32.61(31.00 to 34.28) |  | 2,671(1,080 to 5,532) |  | 19.28(18.55 to 20.04) |  | -2.12(-2.28 to -1.95) |
| Uganda |  | 132,885(107,287 to 159,807) |  | 4008.47(3986.01 to 4031.03) |  | 108,004(70,117 to 150,324) |  | 1018.94(1012.78 to 1025.14) |  | -4.93(-5.30 to -4.55) |  | 734(297 to 1,497) |  | 21.82(20.21 to 23.55) |  | 584(208 to 1,282) |  | 5.47(5.03 to 5.95) |  | -5.02(-5.40 to -4.63) |
| Zambia |  | 74,548(58,819 to 90,752) |  | 4806.66(4770.84 to 4842.70) |  | 187,200(84,708 to 350,078) |  | 4104.39(4085.46 to 4123.39) |  | -3.28(-4.62 to -1.92) |  | 398(155 to 833) |  | 25.27(22.75 to 28.01) |  | 1,004(315 to 2,483) |  | 21.82(20.47 to 23.26) |  | -3.30(-4.63 to -1.96) |
| Botswana |  | 6,605(4,802 to 9,144) |  | 2230.71(2175.89 to 2286.74) |  | 32,629(12,794 to 67,450) |  | 4544.26(4495.01 to 4593.95) |  | 0.77(0.24 to 1.31) |  | 35(14 to 78) |  | 11.75(8.10 to 16.74) |  | 170(47 to 457) |  | 23.66(20.23 to 27.55) |  | 0.75(0.23 to 1.28) |
| Lesotho |  | 21,191(10,042 to 39,739) |  | 5979.97(5899.33 to 6061.49) |  | 28,597(13,669 to 52,989) |  | 5867.38(5798.95 to 5936.52) |  | -3.85(-5.58 to -2.08) |  | 115(38 to 286) |  | 32.29(26.64 to 38.86) |  | 154(50 to 404) |  | 31.36(26.56 to 36.88) |  | -3.79(-5.48 to -2.07) |
| Namibia |  | 8,022(5,912 to 10,464) |  | 2880.65(2816.68 to 2945.82) |  | 19,808(6,571 to 42,758) |  | 3034.73(2992.42 to 3077.52) |  | -1.95(-4.46 to 0.62) |  | 43(17 to 89) |  | 15.40(11.08 to 21.01) |  | 107(27 to 290) |  | 16.37(13.41 to 19.84) |  | -1.94(-4.44 to 0.62) |
| South Africa |  | 603,965(274,935 to 1,124,775) |  | 6510.79(6494.16 to 6527.45) |  | 564,552(178,120 to 1,273,505) |  | 3399.51(3390.63 to 3408.41) |  | -0.71(-1.64 to 0.23) |  | 3,338(1,130 to 8,469) |  | 35.65(34.43 to 36.90) |  | 3,038(759 to 8,289) |  | 18.35(17.70 to 19.02) |  | -0.78(-1.70 to 0.16) |
| Eswatini |  | 4,405(1,412 to 9,073) |  | 2496.22(2421.25 to 2573.20) |  | 8,002(2,446 to 17,221) |  | 2521.62(2466.27 to 2578.07) |  | -0.64(-2.46 to 1.21) |  | 24(6 to 64) |  | 13.69(8.70 to 20.92) |  | 44(10 to 126) |  | 13.68(9.90 to 18.66) |  | -0.68(-2.47 to 1.15) |
| Zimbabwe |  | 47,604(35,654 to 61,906) |  | 2312.81(2291.57 to 2334.22) |  | 128,921(48,224 to 269,824) |  | 3325.84(3307.61 to 3344.16) |  | -1.51(-2.53 to -0.47) |  | 254(101 to 568) |  | 12.25(10.75 to 13.92) |  | 684(189 to 1,747) |  | 17.56(16.26 to 18.94) |  | -1.48(-2.48 to -0.46) |
| Benin |  | 31,766(10,081 to 73,350) |  | 3128.47(3093.09 to 3164.21) |  | 43,774(17,721 to 70,639) |  | 1397.10(1383.77 to 1410.53) |  | -1.88(-2.16 to -1.59) |  | 168(34 to 441) |  | 16.34(13.88 to 19.15) |  | 236(72 to 617) |  | 7.45(6.51 to 8.50) |  | -1.84(-2.11 to -1.57) |
| Burkina Faso |  | 24,829(10,379 to 48,941) |  | 1223.90(1208.36 to 1239.61) |  | 151,357(46,038 to 322,972) |  | 2988.31(2972.98 to 3003.72) |  | 2.15(1.26 to 3.05) |  | 143(45 to 360) |  | 6.88(5.77 to 8.16) |  | 813(173 to 2,240) |  | 15.88(14.79 to 17.05) |  | 2.06(1.18 to 2.94) |
| Cameroon |  | 103,169(65,556 to 152,047) |  | 5018.49(4987.13 to 5050.01) |  | 549,482(278,289 to 975,463) |  | 7408.29(7388.42 to 7428.21) |  | 0.32(-0.92 to 1.58) |  | 563(215 to 1,261) |  | 27.00(24.75 to 29.40) |  | 3,034(983 to 8,142) |  | 40.44(38.99 to 41.94) |  | 0.37(-0.88 to 1.63) |
| Cabo Verde |  | 3,538(1,589 to 6,720) |  | 5089.91(4914.45 to 5270.79) |  | 8,535(3,439 to 16,585) |  | 5591.34(5472.84 to 5711.94) |  | 0.46(0.41 to 0.51) |  | 19(6 to 47) |  | 26.87(15.65 to 44.24) |  | 45(13 to 108) |  | 29.33(21.35 to 39.54) |  | 0.43(0.38 to 0.48) |
| Chad |  | 42,009(12,905 to 93,149) |  | 3442.72(3409.23 to 3476.48) |  | 143,985(52,871 to 287,190) |  | 4267.99(4245.42 to 4290.66) |  | -4.22(-5.54 to -2.87) |  | 220(50 to 602) |  | 17.87(15.54 to 20.48) |  | 756(191 to 1,785) |  | 22.16(20.57 to 23.86) |  | -4.14(-5.44 to -2.82) |
| Côte d'Ivoire |  | 62,095(32,462 to 97,479) |  | 2546.59(2525.73 to 2567.60) |  | 405,916(192,551 to 762,142) |  | 6326.08(6306.44 to 6345.77) |  | 2.59(1.83 to 3.36) |  | 333(113 to 788) |  | 13.43(11.97 to 15.05) |  | 2,148(646 to 5,223) |  | 33.28(31.88 to 34.74) |  | 2.60(1.85 to 3.35) |
| Gambia |  | 9,723(4,321 to 19,271) |  | 4707.80(4610.85 to 4806.66) |  | 28,494(11,775 to 55,242) |  | 5025.31(4965.97 to 5085.28) |  | -0.57(-1.02 to -0.11) |  | 52(15 to 127) |  | 24.90(18.37 to 33.54) |  | 152(45 to 379) |  | 26.50(22.37 to 31.28) |  | -0.56(-1.01 to -0.11) |
| Ghana |  | 54,579(34,383 to 78,853) |  | 1647.66(1633.54 to 1661.90) |  | 377,924(137,289 to 822,057) |  | 4250.66(4237.03 to 4264.33) |  | 0.40(-0.51 to 1.32) |  | 291(107 to 667) |  | 8.71(7.72 to 9.81) |  | 1,996(473 to 5,006) |  | 22.34(21.36 to 23.35) |  | 0.38(-0.53 to 1.30) |
| Guinea |  | 71,490(28,335 to 140,171) |  | 5441.92(5401.75 to 5482.35) |  | 136,365(87,441 to 189,499) |  | 4182.30(4159.80 to 4204.91) |  | -0.20(-0.52 to 0.12) |  | 378(102 to 959) |  | 28.57(25.73 to 31.66) |  | 732(265 to 1,673) |  | 22.32(20.71 to 24.04) |  | -0.16(-0.47 to 0.14) |
| Guinea-Bissau |  | 7,716(2,327 to 16,731) |  | 3554.65(3474.08 to 3636.83) |  | 21,954(8,174 to 42,574) |  | 4375.67(4317.22 to 4434.83) |  | 1.01(0.90 to 1.12) |  | 41(10 to 106) |  | 18.83(13.43 to 25.97) |  | 118(31 to 288) |  | 23.28(19.21 to 28.09) |  | 1.00(0.90 to 1.11) |
| Liberia |  | 23,748(15,134 to 33,919) |  | 4811.37(4748.10 to 4875.38) |  | 87,235(40,516 to 163,086) |  | 6627.31(6583.13 to 6671.72) |  | 0.07(-0.54 to 0.69) |  | 127(46 to 274) |  | 25.25(20.89 to 30.39) |  | 456(132 to 1,138) |  | 34.48(31.37 to 37.84) |  | 0.02(-0.62 to 0.66) |
| Mali |  | 38,353(17,301 to 68,337) |  | 2066.91(2045.85 to 2088.16) |  | 109,179(60,461 to 158,688) |  | 1883.59(1872.16 to 1895.08) |  | 0.69(-0.37 to 1.77) |  | 215(65 to 517) |  | 11.38(9.88 to 13.07) |  | 613(203 to 1,366) |  | 10.45(9.62 to 11.35) |  | 0.74(-0.30 to 1.80) |
| Mauritania |  | 7,683(2,791 to 14,256) |  | 1709.79(1670.79 to 1749.58) |  | 58,064(26,277 to 107,048) |  | 5838.81(5790.73 to 5887.22) |  | 4.36(3.84 to 4.88) |  | 44(11 to 123) |  | 9.53(6.85 to 13.07) |  | 320(95 to 894) |  | 31.83(28.39 to 35.61) |  | 4.27(3.75 to 4.78) |
| Niger |  | 80,500(54,425 to 113,657) |  | 5511.48(5472.71 to 5550.48) |  | 231,243(98,432 to 427,363) |  | 5084.52(5063.09 to 5106.03) |  | -0.42(-1.16 to 0.33) |  | 436(166 to 953) |  | 29.37(26.62 to 32.35) |  | 1,247(349 to 2,931) |  | 26.97(25.44 to 28.58) |  | -0.46(-1.21 to 0.29) |
| Nigeria |  | 744,775(289,713 to 1,502,000) |  | 3863.23(3854.07 to 3872.40) |  | 1,892,833(719,710 to 3,940,193) |  | 3663.66(3658.35 to 3668.98) |  | -1.14(-1.56 to -0.71) |  | 4,056(1,147 to 10,044) |  | 20.69(20.03 to 21.36) |  | 10,217(2,610 to 26,220) |  | 19.57(19.19 to 19.96) |  | -1.12(-1.53 to -0.70) |
| Sao Tome and Principe |  | 464(117 to 1,116) |  | 2049.15(1860.85 to 2253.12) |  | 1,155(275 to 2,799) |  | 2176.47(2052.12 to 2306.82) |  | -0.06(-1.32 to 1.22) |  | 2(0 to 7) |  | 10.83(1.63 to 40.20) |  | 6(1 to 17) |  | 11.56(4.29 to 25.83) |  | -0.06(-1.30 to 1.19) |
| Senegal |  | 39,818(20,416 to 62,556) |  | 2636.54(2610.02 to 2663.30) |  | 117,792(73,765 to 167,570) |  | 3221.63(3203.01 to 3240.34) |  | 1.69(0.86 to 2.53) |  | 219(72 to 538) |  | 14.21(12.34 to 16.32) |  | 644(234 to 1,431) |  | 17.45(16.11 to 18.88) |  | 1.73(0.88 to 2.59) |
| Sierra Leone |  | 54,992(24,036 to 108,934) |  | 5805.79(5756.06 to 5855.92) |  | 137,490(61,834 to 251,113) |  | 6585.11(6549.64 to 6620.76) |  | -0.60(-1.24 to 0.05) |  | 290(86 to 745) |  | 30.28(26.80 to 34.16) |  | 722(220 to 1,839) |  | 34.30(31.79 to 36.98) |  | -0.61(-1.25 to 0.04) |
| Togo |  | 8,217(3,014 to 15,948) |  | 962.86(941.55 to 984.61) |  | 80,306(28,385 to 163,791) |  | 3814.70(3788.27 to 3841.29) |  | 2.66(1.71 to 3.62) |  | 46(12 to 119) |  | 5.32(3.86 to 7.26) |  | 430(110 to 1,145) |  | 20.36(18.47 to 22.39) |  | 2.66(1.72 to 3.60) |
| American Samoa |  | 476(219 to 888) |  | 4273.80(3890.60 to 4688.75) |  | 450(190 to 851) |  | 4069.28(3700.16 to 4466.35) |  | -0.12(-0.15 to -0.09) |  | 3(1 to 7) |  | 23.38(3.92 to 84.18) |  | 2(1 to 6) |  | 22.13(3.52 to 74.42) |  | -0.13(-0.16 to -0.10) |
| Bermuda |  | 1,079(552 to 1,934) |  | 5735.32(5396.64 to 6093.13) |  | 824(404 to 1,502) |  | 5787.12(5388.92 to 6210.97) |  | 0.07(0.05 to 0.08) |  | 6(2 to 16) |  | 31.73(11.50 to 76.18) |  | 5(1 to 12) |  | 31.94(9.20 to 87.52) |  | 0.06(0.05 to 0.08) |
| Cook Islands |  | 185(87 to 333) |  | 4458.73(3832.71 to 5163.28) |  | 178(79 to 327) |  | 4249.83(3646.73 to 4926.74) |  | -0.14(-0.16 to -0.12) |  | 1(0 to 3) |  | 24.33(0.60 to 150.04) |  | 1(0 to 3) |  | 23.13(0.52 to 138.57) |  | -0.15(-0.17 to -0.13) |
| Greenland |  | 161(18 to 405) |  | 977.60(829.60 to 1148.45) |  | 132(13 to 356) |  | 962.12(804.40 to 1143.97) |  | -0.15(-0.20 to -0.10) |  | 1(0 to 3) |  | 5.82(0.12 to 44.55) |  | 1(0 to 3) |  | 5.70(0.06 to 43.72) |  | -0.15(-0.20 to -0.10) |
| Guam |  | 1,516(682 to 2,788) |  | 4271.19(4057.57 to 4494.87) |  | 1,400(539 to 2,632) |  | 3987.53(3781.21 to 4202.35) |  | -0.28(-0.32 to -0.25) |  | 8(3 to 20) |  | 23.44(10.28 to 48.89) |  | 8(2 to 19) |  | 21.75(9.16 to 43.82) |  | -0.28(-0.31 to -0.25) |
| Monaco |  | 178(56 to 359) |  | 2244.20(1921.66 to 2621.42) |  | 172(50 to 351) |  | 2163.80(1846.23 to 2527.64) |  | -0.12(-0.17 to -0.06) |  | 1(0 to 3) |  | 12.82(0.30 to 113.03) |  | 1(0 to 3) |  | 12.25(0.24 to 90.17) |  | -0.13(-0.18 to -0.08) |
| Nauru |  | 105(49 to 193) |  | 4517.63(3687.37 to 5499.13) |  | 119(54 to 220) |  | 4380.76(3626.70 to 5255.15) |  | -0.12(-0.14 to -0.09) |  | 1(0 to 1) |  | 24.70(0.05 to 261.87) |  | 1(0 to 2) |  | 23.81(0.10 to 207.87) |  | -0.12(-0.14 to -0.09) |
| Niue |  | 21(10 to 38) |  | 4512.84(2790.85 to 6916.95) |  | 16(7 to 30) |  | 4319.07(2481.17 to 7021.96) |  | -0.15(-0.17 to -0.13) |  | 0(0 to 0) |  | 24.65(0.00 to 878.82) |  | 0(0 to 0) |  | 23.47(0.00 to 1107.86) |  | -0.17(-0.18 to -0.15) |
| Northern Mariana Islands |  | 607(268 to 1,137) |  | 4342.26(3996.09 to 4716.81) |  | 433(192 to 835) |  | 4241.00(3848.75 to 4664.00) |  | -0.05(-0.08 to -0.02) |  | 3(1 to 9) |  | 23.79(5.26 to 81.85) |  | 2(1 to 6) |  | 23.19(3.53 to 80.43) |  | -0.05(-0.08 to -0.02) |
| Palau |  | 180(85 to 323) |  | 4502.43(3865.49 to 5222.95) |  | 161(66 to 305) |  | 4296.79(3640.87 to 5047.37) |  | -0.10(-0.12 to -0.07) |  | 1(0 to 2) |  | 24.61(0.58 to 161.06) |  | 1(0 to 2) |  | 23.34(0.29 to 175.99) |  | -0.12(-0.14 to -0.09) |
| Puerto Rico |  | 54,882(28,164 to 97,695) |  | 5764.05(5715.91 to 5812.49) |  | 42,735(21,025 to 78,105) |  | 5664.04(5610.18 to 5718.32) |  | -0.05(-0.07 to -0.03) |  | 304(102 to 772) |  | 31.88(28.39 to 35.68) |  | 235(79 to 603) |  | 31.26(27.37 to 35.58) |  | -0.05(-0.07 to -0.03) |
| Saint Kitts and Nevis |  | 566(296 to 1,026) |  | 5762.20(5283.62 to 6282.75) |  | 945(482 to 1,695) |  | 5870.59(5501.44 to 6259.83) |  | 0.04(0.01 to 0.06) |  | 3(1 to 8) |  | 31.82(6.49 to 117.02) |  | 5(2 to 13) |  | 32.38(10.77 to 78.04) |  | 0.04(0.01 to 0.06) |
| San Marino |  | 141(40 to 292) |  | 2267.19(1907.62 to 2676.74) |  | 166(48 to 355) |  | 2202.49(1873.51 to 2578.51) |  | -0.12(-0.18 to -0.05) |  | 1(0 to 2) |  | 12.95(0.15 to 89.08) |  | 1(0 to 3) |  | 12.48(0.22 to 89.74) |  | -0.14(-0.20 to -0.08) |
| Tokelau |  | 15(7 to 26) |  | 4441.70(2439.65 to 7549.40) |  | 13(6 to 25) |  | 4252.75(2283.31 to 7264.48) |  | -0.11(-0.13 to -0.10) |  | 0(0 to 0) |  | 24.34(0.00 to 1474.43) |  | 0(0 to 0) |  | 23.14(0.00 to 1306.63) |  | -0.13(-0.14 to -0.11) |
| Tuvalu |  | 110(53 to 202) |  | 4494.01(3693.13 to 5423.11) |  | 119(54 to 219) |  | 4349.43(3600.29 to 5212.44) |  | -0.08(-0.10 to -0.07) |  | 1(0 to 2) |  | 24.58(0.07 to 214.31) |  | 1(0 to 2) |  | 23.66(0.10 to 191.84) |  | -0.08(-0.10 to -0.05) |
| United States Virgin Islands |  | 1,526(755 to 2,797) |  | 5348.10(5081.49 to 5625.56) |  | 948(463 to 1,759) |  | 5479.32(5132.63 to 5845.08) |  | 0.14(0.12 to 0.15) |  | 8(3 to 22) |  | 29.75(13.12 to 58.65) |  | 5(2 to 13) |  | 30.33(10.00 to 73.58) |  | 0.12(0.10 to 0.14) |
| South Sudan |  | 51,477(24,386 to 90,657) |  | 4344.72(4306.08 to 4383.67) |  | 100,303(50,253 to 179,055) |  | 4720.18(4690.72 to 4749.78) |  | 0.28(0.24 to 0.31) |  | 282(91 to 679) |  | 23.47(20.73 to 26.54) |  | 546(168 to 1,339) |  | 25.56(23.44 to 27.83) |  | 0.29(0.25 to 0.32) |
| Sudan |  | 207,093(155,780 to 267,600) |  | 4954.90(4933.10 to 4976.78) |  | 757,022(396,287 to 1,294,129) |  | 6972.99(6957.16 to 6988.85) |  | 1.76(1.21 to 2.31) |  | 1,136(422 to 2,400) |  | 26.77(25.20 to 28.42) |  | 4,086(1,371 to 9,725) |  | 37.42(36.27 to 38.60) |  | 1.77(1.23 to 2.32) |

**Table S5:** Prevalence and YLDs cases and age-standardised rate of infertility attributable to endometriosis, PCOS and unexplained infertility for WCBA in 2021 by location.

| **location** | **Disease** |  | **Prevalence in 2021** | | | | |  | **YLDs (Years Lived with Disability) in 2021** | | | |
| --- | --- | --- | --- | --- | --- | --- | --- | --- | --- | --- | --- | --- |
|  |  |  | **Number of cases** | |  | **ASR per 100,000 population** | |  | **Number of cases** | |  | **ASR per 100,000 population** |
| **Global** |  |  |  | |  |  | |  |  | |  |  |
|  | Endometriosis |  | 1,185,906(744,392 to 1,820,853) | |  | 60.61(60.50 to 60.72) | |  | 6,820(2,483 to 15,981) | |  | 0.35(0.34 to 0.36) |
|  | Unexplained infertility |  | 110,089,486(58,608,815 to 195,025,585) | |  | 5586.19(5585.15 to 5587.24) | |  | 614,385(216,073 to 1,514,360) | |  | 31.22(31.14 to 31.30) |
|  | Polycystic ovarian syndrome |  | 12,465,534(8,218,101 to 18,556,065) | |  | 638.15(637.79 to 638.50) | |  | 71,555(27,969 to 161,718) | |  | 3.67(3.64 to 3.70) |
| **GBD Region** |  |  |  | |  |  | |  |  | |  |  |
| East Asia | Endometriosis |  | 156,466(94,769 to 244,482) | |  | 44.59(44.36 to 44.82) | |  | 844(293 to 2,071) | |  | 0.24(0.23 to 0.26) |
|  | Unexplained infertility |  | 30,097,127(14,985,652 to 53,555,149) | |  | 8289.89(8286.88 to 8292.89) | |  | 159,881(52,448 to 415,209) | |  | 44.24(44.02 to 44.46) |
|  | Polycystic ovarian syndrome |  | 1,993,543(1,283,858 to 3,063,863) | |  | 602.50(601.64 to 603.37) | |  | 10,765(3,980 to 25,376) | |  | 3.29(3.23 to 3.36) |
| Southeast Asia | Endometriosis |  | 128,269(78,882 to 198,591) | |  | 69.09(68.71 to 69.47) | |  | 740(267 to 1,779) | |  | 0.40(0.37 to 0.43) |
|  | Unexplained infertility |  | 11,004,751(5,574,141 to 19,376,109) | |  | 5898.53(5895.05 to 5902.02) | |  | 61,817(21,273 to 154,949) | |  | 33.17(32.90 to 33.43) |
|  | Polycystic ovarian syndrome |  | 1,909,729(1,240,715 to 2,884,365) | |  | 1034.75(1033.28 to 1036.22) | |  | 11,001(4,214 to 24,631) | |  | 5.98(5.87 to 6.09) |
| Oceania | Endometriosis |  | 3,588(2,139 to 5,501) | |  | 103.14(99.78 to 106.59) | |  | 20(7 to 50) | |  | 0.58(0.36 to 0.91) |
|  | Unexplained infertility |  | 75,092(42,007 to 115,416) | |  | 2159.76(2144.29 to 2175.31) | |  | 425(144 to 1,041) | |  | 12.19(11.05 to 13.41) |
|  | Polycystic ovarian syndrome |  | 22,379(14,387 to 34,265) | |  | 637.62(629.27 to 646.07) | |  | 126(48 to 283) | |  | 3.59(2.99 to 4.29) |
| Central Asia | Endometriosis |  | 14,931(9,173 to 23,072) | |  | 59.83(58.87 to 60.81) | |  | 85(31 to 199) | |  | 0.34(0.27 to 0.43) |
|  | Unexplained infertility |  | 798,112(353,395 to 1,591,761) | |  | 3113.71(3106.86 to 3120.58) | |  | 4,440(1,417 to 11,115) | |  | 17.39(16.88 to 17.91) |
|  | Polycystic ovarian syndrome |  | 45,180(28,235 to 70,452) | |  | 184.63(182.92 to 186.35) | |  | 256(95 to 612) | |  | 1.05(0.93 to 1.19) |
| Central Europe | Endometriosis |  | 13,342(8,230 to 20,893) | |  | 53.04(52.12 to 53.97) | |  | 74(27 to 179) | |  | 0.30(0.23 to 0.38) |
|  | Unexplained infertility |  | 1,334,989(617,168 to 2,521,006) | |  | 5108.75(5099.92 to 5117.59) | |  | 7,261(2,306 to 18,714) | |  | 27.99(27.33 to 28.65) |
|  | Polycystic ovarian syndrome |  | 21,791(13,820 to 33,706) | |  | 87.39(86.20 to 88.60) | |  | 121(45 to 287) | |  | 0.49(0.40 to 0.59) |
| Eastern Europe | Endometriosis |  | 46,639(28,541 to 72,209) | |  | 96.35(95.44 to 97.28) | |  | 268(96 to 637) | |  | 0.56(0.49 to 0.64) |
|  | Unexplained infertility |  | 3,640,758(1,828,022 to 6,714,993) | |  | 7282.72(7274.94 to 7290.51) | |  | 20,461(6,906 to 54,292) | |  | 41.49(40.90 to 42.09) |
|  | Polycystic ovarian syndrome |  | 50,457(31,471 to 79,958) | |  | 104.79(103.84 to 105.76) | |  | 289(105 to 659) | |  | 0.61(0.54 to 0.69) |
| High-income Asia Pacific | Endometriosis |  | 19,825(10,835 to 32,305) | |  | 48.80(48.11 to 49.51) | |  | 111(36 to 284) | |  | 0.28(0.23 to 0.34) |
|  | Unexplained infertility |  | 555,824(51,072 to 1,751,423) | |  | 1222.61(1219.36 to 1225.88) | |  | 3,028(193 to 11,523) | |  | 6.68(6.44 to 6.93) |
|  | Polycystic ovarian syndrome |  | 555,398(361,577 to 807,387) | |  | 1396.20(1392.43 to 1399.98) | |  | 3,087(1,198 to 6,455) | |  | 7.88(7.59 to 8.17) |
| Australasia | Endometriosis |  | 2,623(1,304 to 4,630) | |  | 35.48(34.12 to 36.88) | |  | 15(5 to 37) | |  | 0.21(0.12 to 0.36) |
|  | Unexplained infertility |  | 23,946(5,824 to 97,727) | |  | 308.63(304.72 to 312.59) | |  | 140(21 to 609) | |  | 1.81(1.52 to 2.14) |
|  | Polycystic ovarian syndrome |  | 81,323(49,825 to 126,790) | |  | 1091.33(1083.79 to 1098.92) | |  | 475(175 to 1,021) | |  | 6.42(5.86 to 7.04) |
| Western Europe | Endometriosis |  | 43,119(26,697 to 67,651) | |  | 45.66(45.22 to 46.10) | |  | 260(95 to 613) | |  | 0.28(0.25 to 0.32) |
|  | Unexplained infertility |  | 2,492,439(748,783 to 5,485,462) | |  | 2474.19(2471.09 to 2477.29) | |  | 14,495(3,324 to 41,088) | |  | 14.51(14.27 to 14.75) |
|  | Polycystic ovarian syndrome |  | 1,218,181(811,975 to 1,780,862) | |  | 1275.81(1273.51 to 1278.11) | |  | 7,277(2,911 to 16,359) | |  | 7.73(7.55 to 7.91) |
| Southern Latin America | Endometriosis |  | 7,828(4,958 to 12,047) | |  | 43.65(42.69 to 44.63) | |  | 46(16 to 113) | |  | 0.26(0.19 to 0.34) |
|  | Unexplained infertility |  | 387,109(94,046 to 864,584) | |  | 2142.36(2135.61 to 2149.13) | |  | 2,217(390 to 6,296) | |  | 12.30(11.79 to 12.82) |
|  | Polycystic ovarian syndrome |  | 117,138(74,044 to 179,854) | |  | 661.08(657.30 to 664.89) | |  | 690(261 to 1,562) | |  | 3.91(3.62 to 4.21) |
| High-income North America | Endometriosis |  | 27,843(17,529 to 41,840) | |  | 32.31(31.93 to 32.69) | |  | 167(61 to 379) | |  | 0.19(0.17 to 0.23) |
|  | Unexplained infertility |  | 1,478,922(218,891 to 3,682,781) | |  | 1714.94(1712.17 to 1717.71) | |  | 8,771(1,051 to 28,191) | |  | 10.21(9.99 to 10.42) |
|  | Polycystic ovarian syndrome |  | 1,053,154(712,054 to 1,519,627) | |  | 1240.88(1238.50 to 1243.26) | |  | 6,315(2,468 to 13,943) | |  | 7.48(7.29 to 7.67) |
| Caribbean | Endometriosis |  | 5,823(3,528 to 9,211) | |  | 48.20(46.97 to 49.45) | |  | 34(12 to 80) | |  | 0.28(0.19 to 0.39) |
|  | Unexplained infertility |  | 589,496(342,122 to 997,994) | |  | 4863.01(4850.60 to 4875.44) | |  | 3,311(1,220 to 8,461) | |  | 27.32(26.40 to 28.27) |
|  | Polycystic ovarian syndrome |  | 66,560(42,790 to 101,625) | |  | 554.29(550.08 to 558.52) | |  | 384(148 to 876) | |  | 3.20(2.89 to 3.54) |
| Andean Latin America | Endometriosis |  | 6,877(3,783 to 11,325) | |  | 38.44(37.53 to 39.36) | |  | 39(12 to 91) | |  | 0.22(0.15 to 0.30) |
|  | Unexplained infertility |  | 180,844(18,126 to 574,607) | |  | 1019.66(1014.96 to 1024.37) | |  | 991(84 to 3,500) | |  | 5.59(5.24 to 5.94) |
|  | Polycystic ovarian syndrome |  | 176,064(115,620 to 261,913) | |  | 988.69(984.07 to 993.32) | |  | 977(379 to 2,231) | |  | 5.48(5.14 to 5.84) |
| Central Latin America | Endometriosis |  | 28,901(17,137 to 45,075) | |  | 42.17(41.68 to 42.66) | |  | 164(59 to 398) | |  | 0.24(0.20 to 0.28) |
|  | Unexplained infertility |  | 2,270,205(933,287 to 4,395,051) | |  | 3307.78(3303.48 to 3312.09) | |  | 12,396(3,538 to 33,056) | |  | 18.06(17.74 to 18.38) |
|  | Polycystic ovarian syndrome |  | 689,094(444,481 to 1,016,171) | |  | 1008.30(1005.92 to 1010.69) | |  | 3,873(1,510 to 8,835) | |  | 5.67(5.49 to 5.85) |
| Tropical Latin America | Endometriosis |  | 32,697(19,730 to 51,249) | |  | 53.15(52.58 to 53.74) | |  | 193(70 to 469) | |  | 0.32(0.27 to 0.36) |
|  | Unexplained infertility |  | 2,215,723(943,705 to 4,309,763) | |  | 3428.96(3424.43 to 3433.49) | |  | 12,540(3,928 to 34,219) | |  | 19.47(19.13 to 19.82) |
|  | Polycystic ovarian syndrome |  | 135,259(84,556 to 211,245) | |  | 221.17(219.99 to 222.36) | |  | 792(295 to 1,818) | |  | 1.30(1.21 to 1.40) |
| North Africa and Middle East | Endometriosis |  | 124,240(77,733 to 194,558) | |  | 77.37(76.94 to 77.80) | |  | 735(269 to 1,762) | |  | 0.46(0.43 to 0.49) |
|  | Unexplained infertility |  | 6,357,159(3,075,148 to 10,825,896) | |  | 3897.40(3894.37 to 3900.44) | |  | 36,969(11,255 to 92,996) | |  | 22.71(22.48 to 22.94) |
|  | Polycystic ovarian syndrome |  | 1,326,036(846,139 to 2,061,910) | |  | 828.11(826.70 to 829.52) | |  | 7,844(3,024 to 17,832) | |  | 4.91(4.80 to 5.02) |
| South Asia | Endometriosis |  | 327,167(203,560 to 507,520) | |  | 65.70(65.58 to 65.81) | |  | 1,914(704 to 4,584) | |  | 0.38(0.38 to 0.39) |
|  | Unexplained infertility |  | 35,555,278(19,863,521 to 60,335,282) | |  | 7119.36(7118.18 to 7120.53) | |  | 204,124(73,277 to 482,246) | |  | 40.82(40.73 to 40.91) |
|  | Polycystic ovarian syndrome |  | 2,198,417(1,394,157 to 3,337,554) | |  | 441.86(441.57 to 442.15) | |  | 12,709(4,770 to 29,126) | |  | 2.55(2.53 to 2.57) |
| Central Sub-Saharan Africa | Endometriosis |  | 23,482(14,124 to 35,759) | |  | 74.35(73.38 to 75.32) | |  | 134(49 to 321) | |  | 0.42(0.35 to 0.50) |
|  | Unexplained infertility |  | 1,705,616(872,692 to 3,067,605) | |  | 5790.10(5781.31 to 5798.90) | |  | 9,424(2,975 to 22,622) | |  | 31.76(31.12 to 32.42) |
|  | Polycystic ovarian syndrome |  | 88,814(55,549 to 140,433) | |  | 270.88(269.06 to 272.71) | |  | 505(190 to 1,156) | |  | 1.52(1.39 to 1.67) |
| Eastern Sub-Saharan Africa | Endometriosis |  | 65,613(39,784 to 102,293) | |  | 62.85(62.36 to 63.35) | |  | 372(137 to 874) | |  | 0.35(0.32 to 0.39) |
|  | Unexplained infertility |  | 3,960,451(2,299,441 to 6,526,195) | |  | 3974.09(3970.12 to 3978.07) | |  | 22,034(7,888 to 51,730) | |  | 21.93(21.64 to 22.23) |
|  | Polycystic ovarian syndrome |  | 284,221(179,654 to 439,995) | |  | 261.48(260.50 to 262.47) | |  | 1,606(603 to 3,639) | |  | 1.46(1.39 to 1.54) |
| Southern Sub-Saharan Africa | Endometriosis |  | 14,121(8,765 to 22,267) | |  | 63.59(62.54 to 64.65) | |  | 81(29 to 190) | |  | 0.37(0.29 to 0.46) |
|  | Unexplained infertility |  | 782,508(260,859 to 1,743,635) | |  | 3466.06(3458.38 to 3473.76) | |  | 4,346(1,128 to 11,746) | |  | 19.27(18.70 to 19.85) |
|  | Polycystic ovarian syndrome |  | 88,848(56,567 to 136,584) | |  | 401.86(399.21 to 404.51) | |  | 509(197 to 1,171) | |  | 2.30(2.11 to 2.51) |
| Western Sub-Saharan Africa | Endometriosis |  | 92,514(56,347 to 143,095) | |  | 79.19(78.67 to 79.71) | |  | 524(188 to 1,232) | |  | 0.44(0.41 to 0.48) |
|  | Unexplained infertility |  | 4,583,137(2,061,353 to 8,923,482) | |  | 4156.79(4152.92 to 4160.66) | |  | 25,315(7,432 to 63,557) | |  | 22.76(22.47 to 23.04) |
|  | Polycystic ovarian syndrome |  | 343,948(216,042 to 538,311) | |  | 283.84(282.87 to 284.81) | |  | 1,954(753 to 4,460) | |  | 1.59(1.52 to 1.67) |
| **Countries and territories** |  |  |  | |  |  | |  |  | |  |  |
| China | Endometriosis |  | 145,510(87,361 to 227,669) | |  | 43.04(42.81 to 43.27) | |  | 784(271 to 1,922) | |  | 0.23(0.22 to 0.25) |
|  | Unexplained infertility |  | 29,317,000(14,569,167 to 52,098,692) | |  | 8374.49(8371.41 to 8377.57) | |  | 155,737(51,019 to 405,081) | |  | 44.69(44.47 to 44.92) |
|  | Polycystic ovarian syndrome |  | 1,916,743(1,234,703 to 2,946,495) | |  | 601.73(600.84 to 602.61) | |  | 10,349(3,825 to 24,418) | |  | 3.29(3.22 to 3.35) |
| Democratic People's Republic of Korea | Endometriosis |  | 4,873(2,922 to 7,719) | |  | 72.20(70.18 to 74.26) | |  | 27(9 to 67) | |  | 0.39(0.26 to 0.58) |
|  | Unexplained infertility |  | 410,661(201,844 to 776,717) | |  | 6133.59(6114.83 to 6152.39) | |  | 2,185(674 to 5,110) | |  | 32.65(31.29 to 34.05) |
|  | Polycystic ovarian syndrome |  | 25,000(16,085 to 38,556) | |  | 380.75(376.02 to 385.52) | |  | 136(50 to 312) | |  | 2.08(1.75 to 2.47) |
| Cambodia | Endometriosis |  | 3,325(2,001 to 5,303) | |  | 72.17(69.73 to 74.68) | |  | 19(6 to 46) | |  | 0.40(0.24 to 0.64) |
|  | Unexplained infertility |  | 220,334(102,200 to 415,498) | |  | 4753.43(4733.55 to 4773.38) | |  | 1,193(368 to 3,020) | |  | 25.73(24.29 to 27.24) |
|  | Polycystic ovarian syndrome |  | 34,119(21,753 to 52,155) | |  | 741.65(733.77 to 749.59) | |  | 191(69 to 442) | |  | 4.15(3.58 to 4.79) |
| Indonesia | Endometriosis |  | 56,631(35,545 to 87,645) | |  | 73.99(73.38 to 74.60) | |  | 324(116 to 789) | |  | 0.42(0.38 to 0.47) |
|  | Unexplained infertility |  | 6,251,542(3,293,414 to 11,133,561) | |  | 8127.14(8120.76 to 8133.52) | |  | 34,723(11,959 to 84,489) | |  | 45.20(44.72 to 45.68) |
|  | Polycystic ovarian syndrome |  | 832,688(547,306 to 1,252,967) | |  | 1096.94(1094.58 to 1099.31) | |  | 4,781(1,859 to 10,720) | |  | 6.32(6.14 to 6.50) |
| Lao People's Democratic Republic | Endometriosis |  | 1,483(883 to 2,340) | |  | 74.06(70.32 to 77.95) | |  | 9(3 to 21) | |  | 0.43(0.19 to 0.85) |
|  | Unexplained infertility |  | 78,106(34,643 to 147,647) | |  | 3972.06(3944.15 to 4000.13) | |  | 435(125 to 1,121) | |  | 22.06(20.03 to 24.26) |
|  | Polycystic ovarian syndrome |  | 17,841(11,504 to 26,759) | |  | 888.17(875.13 to 901.37) | |  | 103(39 to 235) | |  | 5.12(4.18 to 6.23) |
| Malaysia | Endometriosis |  | 5,822(3,449 to 9,158) | |  | 67.09(65.38 to 68.85) | |  | 32(11 to 79) | |  | 0.37(0.26 to 0.53) |
|  | Unexplained infertility |  | 407,011(162,381 to 812,003) | |  | 4698.62(4684.18 to 4713.10) | |  | 2,193(620 to 5,698) | |  | 25.31(24.26 to 26.39) |
|  | Polycystic ovarian syndrome |  | 124,874(78,847 to 187,685) | |  | 1442.49(1434.49 to 1450.53) | |  | 693(265 to 1,606) | |  | 8.00(7.41 to 8.62) |
| Maldives | Endometriosis |  | 87(53 to 134) | |  | 70.15(55.98 to 87.29) | |  | 1(0 to 1) | |  | 0.41(0.00 to 5.06) |
|  | Unexplained infertility |  | 9,189(7,308 to 11,222) | |  | 7024.63(6880.41 to 7171.53) | |  | 53(21 to 112) | |  | 40.73(30.41 to 53.98) |
|  | Polycystic ovarian syndrome |  | 1,426(912 to 2,233) | |  | 1170.32(1109.42 to 1234.06) | |  | 8(3 to 19) | |  | 6.87(2.97 to 14.17) |
| Myanmar | Endometriosis |  | 9,677(5,875 to 15,200) | |  | 64.60(63.32 to 65.90) | |  | 57(19 to 143) | |  | 0.38(0.29 to 0.49) |
|  | Unexplained infertility |  | 296,755(201,457 to 389,740) | |  | 2018.01(2010.75 to 2025.28) | |  | 1,682(637 to 3,787) | |  | 11.43(10.89 to 11.99) |
|  | Polycystic ovarian syndrome |  | 131,154(85,359 to 193,639) | |  | 873.94(869.22 to 878.69) | |  | 764(293 to 1,690) | |  | 5.08(4.73 to 5.46) |
| Philippines | Endometriosis |  | 22,683(13,838 to 35,236) | |  | 77.63(76.62 to 78.65) | |  | 135(48 to 320) | |  | 0.46(0.39 to 0.55) |
|  | Unexplained infertility |  | 2,499,077(851,174 to 4,963,466) | |  | 8643.51(8632.77 to 8654.26) | |  | 14,613(3,870 to 35,879) | |  | 50.45(49.63 to 51.28) |
|  | Polycystic ovarian syndrome |  | 291,387(185,726 to 443,166) | |  | 991.72(988.11 to 995.34) | |  | 1,723(643 to 3,908) | |  | 5.85(5.57 to 6.13) |
| Sri Lanka | Endometriosis |  | 3,268(1,994 to 5,004) | |  | 57.95(55.97 to 59.98) | |  | 19(6 to 43) | |  | 0.33(0.20 to 0.52) |
|  | Unexplained infertility |  | 183,588(67,464 to 366,837) | |  | 3175.54(3160.98 to 3190.16) | |  | 1,018(266 to 2,617) | |  | 17.65(16.57 to 18.77) |
|  | Polycystic ovarian syndrome |  | 62,870(40,457 to 95,051) | |  | 1118.99(1110.22 to 1127.80) | |  | 360(137 to 819) | |  | 6.43(5.78 to 7.13) |
| Thailand | Endometriosis |  | 9,100(5,601 to 13,961) | |  | 55.52(54.37 to 56.70) | |  | 53(19 to 129) | |  | 0.33(0.24 to 0.43) |
|  | Unexplained infertility |  | 571,872(217,570 to 1,103,625) | |  | 3394.21(3385.29 to 3403.14) | |  | 3,229(815 to 8,352) | |  | 19.24(18.58 to 19.93) |
|  | Polycystic ovarian syndrome |  | 199,298(128,754 to 304,414) | |  | 1234.34(1228.82 to 1239.88) | |  | 1,165(437 to 2,672) | |  | 7.29(6.87 to 7.73) |
| Timor-Leste | Endometriosis |  | 312(183 to 487) | |  | 96.12(85.46 to 107.84) | |  | 2(1 to 4) | |  | 0.56(0.05 to 2.42) |
|  | Unexplained infertility |  | 8,939(4,102 to 16,409) | |  | 3039.85(2975.87 to 3104.91) | |  | 51(15 to 123) | |  | 17.14(12.67 to 22.75) |
|  | Polycystic ovarian syndrome |  | 2,523(1,606 to 3,934) | |  | 752.70(722.69 to 783.73) | |  | 15(6 to 34) | |  | 4.39(2.40 to 7.52) |
| Viet Nam | Endometriosis |  | 15,506(9,218 to 23,983) | |  | 58.26(57.34 to 59.20) | |  | 88(31 to 214) | |  | 0.33(0.27 to 0.41) |
|  | Unexplained infertility |  | 449,459(113,365 to 985,469) | |  | 1596.84(1592.16 to 1601.53) | |  | 2,467(477 to 6,804) | |  | 8.79(8.45 to 9.15) |
|  | Polycystic ovarian syndrome |  | 204,342(130,950 to 304,918) | |  | 776.30(772.90 to 779.71) | |  | 1,157(435 to 2,607) | |  | 4.43(4.18 to 4.70) |
| Fiji | Endometriosis |  | 192(118 to 298) | |  | 83.91(72.47 to 96.67) | |  | 1(0 to 3) | |  | 0.48(0.02 to 2.56) |
|  | Unexplained infertility |  | 15,293(8,060 to 26,195) | |  | 6609.50(6505.13 to 6715.16) | |  | 87(29 to 212) | |  | 37.46(29.99 to 46.26) |
|  | Polycystic ovarian syndrome |  | 2,125(1,361 to 3,263) | |  | 928.18(889.12 to 968.52) | |  | 12(5 to 28) | |  | 5.37(2.80 to 9.34) |
| Kiribati | Endometriosis |  | 32(19 to 49) | |  | 100.99(69.15 to 143.53) | |  | 0(0 to 0) | |  | 0.58(0.00 to 15.42) |
|  | Unexplained infertility |  | 1,388(632 to 2,564) | |  | 4378.23(4150.59 to 4615.90) | |  | 8(2 to 19) | |  | 24.40(10.35 to 50.01) |
|  | Polycystic ovarian syndrome |  | 241(155 to 365) | |  | 747.12(655.61 to 848.75) | |  | 1(1 to 3) | |  | 4.26(0.25 to 21.45) |
| Marshall Islands | Endometriosis |  | 12(7 to 19) | |  | 83.28(43.20 to 146.09) | |  | 0(0 to 0) | |  | 0.47(0.00 to 28.52) |
|  | Unexplained infertility |  | 634(294 to 1,154) | |  | 4346.43(4014.51 to 4699.14) | |  | 4(1 to 9) | |  | 24.28(5.92 to 66.98) |
|  | Polycystic ovarian syndrome |  | 108(69 to 162) | |  | 732.33(600.58 to 885.22) | |  | 1(0 to 1) | |  | 4.17(0.01 to 35.30) |
| Micronesia (Federated States of) | Endometriosis |  | 21(13 to 33) | |  | 84.47(52.27 to 129.74) | |  | 0(0 to 0) | |  | 0.48(0.00 to 17.28) |
|  | Unexplained infertility |  | 1,040(480 to 1,889) | |  | 4306.44(4047.73 to 4577.62) | |  | 6(2 to 15) | |  | 24.04(8.62 to 53.58) |
|  | Polycystic ovarian syndrome |  | 205(130 to 311) | |  | 800.31(693.95 to 918.93) | |  | 1(0 to 3) | |  | 4.57(0.18 to 24.39) |
| Papua New Guinea | Endometriosis |  | 2,785(1,652 to 4,320) | |  | 105.53(101.63 to 109.55) | |  | 16(6 to 39) | |  | 0.60(0.34 to 0.99) |
|  | Unexplained infertility |  | 37,489(20,998 to 55,295) | |  | 1385.00(1370.98 to 1399.14) | |  | 213(67 to 529) | |  | 7.86(6.83 to 9.00) |
|  | Polycystic ovarian syndrome |  | 15,520(9,974 to 23,751) | |  | 582.63(573.47 to 591.91) | |  | 87(33 to 196) | |  | 3.26(2.61 to 4.04) |
| Samoa | Endometriosis |  | 39(24 to 61) | |  | 84.30(59.88 to 115.83) | |  | 0(0 to 1) | |  | 0.48(0.00 to 10.21) |
|  | Unexplained infertility |  | 1,945(846 to 3,639) | |  | 4349.17(4156.91 to 4548.30) | |  | 11(3 to 27) | |  | 24.22(11.98 to 44.09) |
|  | Polycystic ovarian syndrome |  | 426(265 to 642) | |  | 890.06(806.84 to 979.98) | |  | 2(1 to 6) | |  | 5.06(0.79 to 17.57) |
| Solomon Islands | Endometriosis |  | 192(113 to 308) | |  | 115.37(99.57 to 133.06) | |  | 1(0 to 3) | |  | 0.66(0.02 to 3.71) |
|  | Unexplained infertility |  | 6,866(3,178 to 12,522) | |  | 4217.50(4118.09 to 4318.77) | |  | 39(12 to 99) | |  | 23.62(16.74 to 32.47) |
|  | Polycystic ovarian syndrome |  | 1,157(741 to 1,771) | |  | 679.10(640.34 to 719.70) | |  | 7(3 to 15) | |  | 3.88(1.51 to 8.37) |
| Tonga | Endometriosis |  | 23(14 to 36) | |  | 95.65(60.68 to 143.79) | |  | 0(0 to 0) | |  | 0.55(0.00 to 17.29) |
|  | Unexplained infertility |  | 1,010(425 to 1,854) | |  | 4263.82(4004.40 to 4535.81) | |  | 6(2 to 14) | |  | 23.67(8.31 to 53.19) |
|  | Polycystic ovarian syndrome |  | 251(158 to 384) | |  | 1012.06(890.14 to 1146.38) | |  | 1(1 to 3) | |  | 5.75(0.37 to 26.36) |
| Vanuatu | Endometriosis |  | 69(42 to 106) | |  | 89.71(69.68 to 114.01) | |  | 0(0 to 1) | |  | 0.51(0.00 to 6.66) |
|  | Unexplained infertility |  | 3,164(1,491 to 5,694) | |  | 4262.78(4114.77 to 4414.95) | |  | 18(6 to 45) | |  | 23.86(14.05 to 38.21) |
|  | Polycystic ovarian syndrome |  | 582(375 to 894) | |  | 742.42(682.92 to 806.00) | |  | 3(1 to 8) | |  | 4.24(0.96 to 12.45) |
| Armenia | Endometriosis |  | 325(210 to 496) | |  | 43.28(38.58 to 48.49) | |  | 2(1 to 4) | |  | 0.24(0.02 to 1.14) |
|  | Unexplained infertility |  | 7,690(4,384 to 11,654) | |  | 1002.11(979.23 to 1025.47) | |  | 42(15 to 97) | |  | 5.49(3.91 to 7.58) |
|  | Polycystic ovarian syndrome |  | 1,219(760 to 1,900) | |  | 164.24(154.83 to 174.14) | |  | 7(2 to 16) | |  | 0.91(0.34 to 2.09) |
| Azerbaijan | Endometriosis |  | 1,608(988 to 2,569) | |  | 56.08(53.34 to 58.95) | |  | 9(3 to 21) | |  | 0.32(0.15 to 0.64) |
|  | Unexplained infertility |  | 135,501(56,369 to 276,168) | |  | 4514.67(4490.40 to 4539.06) | |  | 761(237 to 2,053) | |  | 25.52(23.72 to 27.45) |
|  | Polycystic ovarian syndrome |  | 5,437(3,376 to 8,553) | |  | 196.53(191.24 to 201.95) | |  | 31(11 to 73) | |  | 1.13(0.76 to 1.64) |
| Georgia | Endometriosis |  | 457(283 to 700) | |  | 58.93(53.55 to 64.76) | |  | 3(1 to 6) | |  | 0.34(0.06 to 1.21) |
|  | Unexplained infertility |  | 26,627(9,016 to 57,764) | |  | 3261.14(3221.50 to 3301.22) | |  | 146(37 to 383) | |  | 18.09(15.23 to 21.39) |
|  | Polycystic ovarian syndrome |  | 2,078(1,312 to 3,254) | |  | 270.45(258.66 to 282.68) | |  | 12(4 to 28) | |  | 1.54(0.77 to 2.83) |
| Kazakhstan | Endometriosis |  | 3,028(1,836 to 4,747) | |  | 63.12(60.86 to 65.44) | |  | 17(6 to 41) | |  | 0.36(0.21 to 0.59) |
|  | Unexplained infertility |  | 175,036(68,958 to 370,779) | |  | 3514.48(3497.86 to 3531.16) | |  | 975(284 to 2,471) | |  | 19.70(18.47 to 21.00) |
|  | Polycystic ovarian syndrome |  | 9,410(5,865 to 15,006) | |  | 199.47(195.41 to 203.61) | |  | 53(20 to 124) | |  | 1.14(0.85 to 1.50) |
| Kyrgyzstan | Endometriosis |  | 1,192(719 to 1,881) | |  | 67.16(63.39 to 71.11) | |  | 7(2 to 16) | |  | 0.38(0.15 to 0.81) |
|  | Unexplained infertility |  | 85,510(40,458 to 163,876) | |  | 4800.98(4768.73 to 4833.40) | |  | 471(152 to 1,146) | |  | 26.46(24.12 to 28.98) |
|  | Polycystic ovarian syndrome |  | 2,786(1,746 to 4,466) | |  | 159.75(153.85 to 165.83) | |  | 16(6 to 35) | |  | 0.90(0.51 to 1.48) |
| Mongolia | Endometriosis |  | 586(354 to 940) | |  | 68.10(62.66 to 73.93) | |  | 3(1 to 8) | |  | 0.39(0.09 to 1.16) |
|  | Unexplained infertility |  | 31,156(12,719 to 62,694) | |  | 3498.95(3459.96 to 3538.30) | |  | 172(51 to 454) | |  | 19.44(16.63 to 22.64) |
|  | Polycystic ovarian syndrome |  | 1,495(930 to 2,365) | |  | 177.20(168.24 to 186.54) | |  | 9(3 to 20) | |  | 1.01(0.45 to 2.03) |
| Tajikistan | Endometriosis |  | 1,643(995 to 2,610) | |  | 61.63(58.67 to 64.71) | |  | 10(4 to 24) | |  | 0.36(0.17 to 0.70) |
|  | Unexplained infertility |  | 33,510(21,602 to 46,127) | |  | 1201.06(1188.21 to 1214.02) | |  | 197(74 to 444) | |  | 7.08(6.13 to 8.16) |
|  | Polycystic ovarian syndrome |  | 3,416(2,116 to 5,396) | |  | 130.05(125.70 to 134.52) | |  | 20(7 to 49) | |  | 0.76(0.46 to 1.20) |
| Turkmenistan | Endometriosis |  | 844(514 to 1,330) | |  | 66.63(62.21 to 71.29) | |  | 5(2 to 12) | |  | 0.38(0.12 to 0.91) |
|  | Unexplained infertility |  | 46,457(20,257 to 92,568) | |  | 3681.71(3648.29 to 3715.37) | |  | 258(79 to 659) | |  | 20.39(17.98 to 23.05) |
|  | Polycystic ovarian syndrome |  | 2,426(1,508 to 3,902) | |  | 192.60(185.00 to 200.42) | |  | 14(5 to 33) | |  | 1.09(0.59 to 1.84) |
| Uzbekistan | Endometriosis |  | 5,247(3,148 to 8,233) | |  | 57.02(55.48 to 58.59) | |  | 30(10 to 69) | |  | 0.33(0.22 to 0.47) |
|  | Unexplained infertility |  | 256,625(98,734 to 509,274) | |  | 2728.47(2717.89 to 2739.08) | |  | 1,418(407 to 3,776) | |  | 15.11(14.34 to 15.93) |
|  | Polycystic ovarian syndrome |  | 16,913(10,690 to 26,358) | |  | 187.82(184.98 to 190.69) | |  | 95(33 to 223) | |  | 1.06(0.86 to 1.31) |
| Albania | Endometriosis |  | 331(204 to 525) | |  | 53.60(47.97 to 59.75) | |  | 2(1 to 4) | |  | 0.31(0.04 to 1.26) |
|  | Unexplained infertility |  | 12,163(8,866 to 15,886) | |  | 1970.86(1935.97 to 2006.25) | |  | 69(27 to 142) | |  | 11.20(8.72 to 14.23) |
|  | Polycystic ovarian syndrome |  | 409(246 to 653) | |  | 67.52(61.11 to 74.45) | |  | 2(1 to 6) | |  | 0.40(0.06 to 1.39) |
| Bosnia and Herzegovina | Endometriosis |  | 370(230 to 580) | |  | 51.57(46.36 to 57.26) | |  | 2(1 to 5) | |  | 0.29(0.03 to 1.18) |
|  | Unexplained infertility |  | 28,239(11,725 to 59,026) | |  | 3864.43(3819.00 to 3910.32) | |  | 153(44 to 399) | |  | 21.07(17.84 to 24.79) |
|  | Polycystic ovarian syndrome |  | 517(313 to 835) | |  | 72.54(66.30 to 79.28) | |  | 3(1 to 7) | |  | 0.41(0.08 to 1.36) |
| Bulgaria | Endometriosis |  | 751(453 to 1,193) | |  | 55.29(51.26 to 59.58) | |  | 4(1 to 10) | |  | 0.31(0.08 to 0.87) |
|  | Unexplained infertility |  | 56,192(22,571 to 120,187) | |  | 3907.11(3873.96 to 3940.50) | |  | 303(89 to 770) | |  | 21.24(18.85 to 23.89) |
|  | Polycystic ovarian syndrome |  | 1,124(694 to 1,792) | |  | 82.68(77.71 to 87.92) | |  | 6(2 to 15) | |  | 0.46(0.16 to 1.08) |
| Croatia | Endometriosis |  | 397(258 to 592) | |  | 45.42(40.99 to 50.24) | |  | 2(1 to 5) | |  | 0.26(0.03 to 1.00) |
|  | Unexplained infertility |  | 35,645(14,306 to 77,357) | |  | 3920.96(3879.93 to 3962.37) | |  | 193(56 to 479) | |  | 21.34(18.40 to 24.67) |
|  | Polycystic ovarian syndrome |  | 697(421 to 1,125) | |  | 79.71(73.78 to 86.04) | |  | 4(1 to 10) | |  | 0.45(0.11 to 1.29) |
| Czechia | Endometriosis |  | 1,276(780 to 1,975) | |  | 58.83(55.52 to 62.32) | |  | 7(3 to 17) | |  | 0.33(0.12 to 0.73) |
|  | Unexplained infertility |  | 128,037(58,522 to 245,085) | |  | 5694.15(5662.13 to 5726.33) | |  | 690(219 to 1,795) | |  | 30.91(28.58 to 33.40) |
|  | Polycystic ovarian syndrome |  | 1,729(1,050 to 2,794) | |  | 80.03(76.12 to 84.10) | |  | 9(3 to 23) | |  | 0.44(0.20 to 0.89) |
| Hungary | Endometriosis |  | 1,068(670 to 1,710) | |  | 52.67(49.47 to 56.04) | |  | 6(2 to 15) | |  | 0.30(0.10 to 0.70) |
|  | Unexplained infertility |  | 80,012(33,659 to 166,650) | |  | 3879.23(3851.91 to 3906.72) | |  | 433(127 to 1,098) | |  | 21.11(19.14 to 23.26) |
|  | Polycystic ovarian syndrome |  | 1,643(1,014 to 2,654) | |  | 81.03(77.03 to 85.21) | |  | 9(3 to 21) | |  | 0.45(0.20 to 0.90) |
| North Macedonia | Endometriosis |  | 285(174 to 444) | |  | 53.27(47.16 to 60.06) | |  | 2(1 to 4) | |  | 0.30(0.02 to 1.53) |
|  | Unexplained infertility |  | 21,438(8,983 to 45,206) | |  | 3850.37(3798.48 to 3902.89) | |  | 116(34 to 295) | |  | 21.02(17.34 to 25.39) |
|  | Polycystic ovarian syndrome |  | 390(236 to 644) | |  | 74.19(66.84 to 82.23) | |  | 2(1 to 5) | |  | 0.42(0.05 to 1.71) |
| Montenegro | Endometriosis |  | 79(48 to 125) | |  | 55.86(44.10 to 70.00) | |  | 0(0 to 1) | |  | 0.31(0.00 to 3.85) |
|  | Unexplained infertility |  | 5,381(2,228 to 11,408) | |  | 3741.02(3640.97 to 3843.32) | |  | 29(8 to 74) | |  | 20.46(13.69 to 29.68) |
|  | Polycystic ovarian syndrome |  | 115(70 to 185) | |  | 81.45(67.06 to 98.22) | |  | 1(0 to 2) | |  | 0.46(0.00 to 4.10) |
| Poland | Endometriosis |  | 4,608(2,870 to 7,262) | |  | 51.85(50.31 to 53.43) | |  | 26(9 to 61) | |  | 0.29(0.19 to 0.45) |
|  | Unexplained infertility |  | 657,230(324,241 to 1,192,629) | |  | 7059.43(7041.91 to 7077.00) | |  | 3,590(1,161 to 9,124) | |  | 38.93(37.63 to 40.27) |
|  | Polycystic ovarian syndrome |  | 9,009(5,795 to 13,617) | |  | 104.53(102.28 to 106.82) | |  | 50(19 to 119) | |  | 0.59(0.43 to 0.80) |
| Romania | Endometriosis |  | 2,093(1,261 to 3,262) | |  | 54.67(52.28 to 57.14) | |  | 12(4 to 28) | |  | 0.30(0.15 to 0.56) |
|  | Unexplained infertility |  | 150,446(61,535 to 319,646) | |  | 3819.78(3800.11 to 3839.53) | |  | 816(240 to 2,141) | |  | 20.84(19.40 to 22.36) |
|  | Polycystic ovarian syndrome |  | 3,037(1,836 to 4,903) | |  | 78.51(75.66 to 81.45) | |  | 17(6 to 41) | |  | 0.44(0.25 to 0.72) |
| Serbia | Endometriosis |  | 974(587 to 1,517) | |  | 48.88(45.82 to 52.11) | |  | 5(2 to 14) | |  | 0.27(0.09 to 0.65) |
|  | Unexplained infertility |  | 78,591(31,617 to 161,867) | |  | 3840.68(3813.63 to 3867.89) | |  | 427(125 to 1,114) | |  | 20.95(18.99 to 23.07) |
|  | Polycystic ovarian syndrome |  | 1,492(911 to 2,405) | |  | 75.03(71.21 to 79.01) | |  | 8(3 to 20) | |  | 0.42(0.18 to 0.85) |
| Slovakia | Endometriosis |  | 648(402 to 993) | |  | 52.07(48.01 to 56.43) | |  | 4(1 to 8) | |  | 0.29(0.07 to 0.91) |
|  | Unexplained infertility |  | 50,680(21,085 to 109,855) | |  | 3864.96(3830.64 to 3899.56) | |  | 275(82 to 710) | |  | 21.11(18.63 to 23.88) |
|  | Polycystic ovarian syndrome |  | 979(603 to 1,574) | |  | 79.30(74.21 to 84.70) | |  | 5(2 to 13) | |  | 0.44(0.14 to 1.12) |
| Slovenia | Endometriosis |  | 269(161 to 428) | |  | 65.92(58.03 to 74.70) | |  | 1(0 to 4) | |  | 0.36(0.02 to 1.94) |
|  | Unexplained infertility |  | 11,502(3,547 to 26,046) | |  | 2675.13(2625.38 to 2725.71) | |  | 61(13 to 171) | |  | 14.24(10.83 to 18.58) |
|  | Polycystic ovarian syndrome |  | 332(205 to 535) | |  | 81.06(72.26 to 90.76) | |  | 2(1 to 4) | |  | 0.43(0.04 to 2.05) |
| Belarus | Endometriosis |  | 1,946(1,173 to 3,076) | |  | 91.33(87.15 to 95.69) | |  | 11(4 to 27) | |  | 0.53(0.26 to 1.02) |
|  | Unexplained infertility |  | 115,515(50,429 to 227,190) | |  | 5184.65(5153.73 to 5215.73) | |  | 649(203 to 1,707) | |  | 29.48(27.18 to 31.96) |
|  | Polycystic ovarian syndrome |  | 2,184(1,366 to 3,603) | |  | 104.30(99.75 to 109.03) | |  | 13(5 to 31) | |  | 0.61(0.31 to 1.12) |
| Estonia | Endometriosis |  | 241(145 to 368) | |  | 86.97(76.03 to 99.21) | |  | 1(0 to 3) | |  | 0.50(0.03 to 2.78) |
|  | Unexplained infertility |  | 15,140(6,954 to 28,747) | |  | 5283.29(5197.56 to 5370.26) | |  | 85(28 to 222) | |  | 29.91(23.75 to 37.39) |
|  | Polycystic ovarian syndrome |  | 323(201 to 524) | |  | 118.27(105.30 to 132.57) | |  | 2(1 to 4) | |  | 0.69(0.06 to 3.07) |
| Latvia | Endometriosis |  | 319(192 to 508) | |  | 83.28(74.09 to 93.43) | |  | 2(1 to 5) | |  | 0.48(0.04 to 2.22) |
|  | Unexplained infertility |  | 21,562(9,796 to 41,262) | |  | 5410.90(5337.07 to 5485.63) | |  | 120(38 to 310) | |  | 30.34(25.02 to 36.63) |
|  | Polycystic ovarian syndrome |  | 412(254 to 649) | |  | 108.66(98.03 to 120.26) | |  | 2(1 to 5) | |  | 0.63(0.09 to 2.45) |
| Lithuania | Endometriosis |  | 564(342 to 885) | |  | 98.39(90.34 to 107.07) | |  | 3(1 to 7) | |  | 0.56(0.12 to 1.82) |
|  | Unexplained infertility |  | 32,232(15,148 to 62,109) | |  | 5553.03(5492.22 to 5614.45) | |  | 178(57 to 465) | |  | 30.81(26.42 to 35.82) |
|  | Polycystic ovarian syndrome |  | 587(359 to 941) | |  | 105.01(96.51 to 114.14) | |  | 3(1 to 8) | |  | 0.60(0.13 to 1.90) |
| Republic of Moldova | Endometriosis |  | 746(459 to 1,150) | |  | 83.02(76.93 to 89.56) | |  | 4(2 to 10) | |  | 0.48(0.13 to 1.42) |
|  | Unexplained infertility |  | 52,438(24,238 to 98,104) | |  | 5555.96(5506.94 to 5605.39) | |  | 296(98 to 761) | |  | 31.79(28.15 to 35.86) |
|  | Polycystic ovarian syndrome |  | 855(526 to 1,362) | |  | 97.44(90.68 to 104.65) | |  | 5(2 to 12) | |  | 0.58(0.17 to 1.56) |
| Russian Federation | Endometriosis |  | 33,486(20,558 to 51,915) | |  | 98.58(97.48 to 99.70) | |  | 191(69 to 466) | |  | 0.57(0.49 to 0.66) |
|  | Unexplained infertility |  | 2,715,415(1,370,335 to 5,019,099) | |  | 7717.36(7707.79 to 7726.93) | |  | 15,176(5,194 to 39,508) | |  | 43.73(43.00 to 44.46) |
|  | Polycystic ovarian syndrome |  | 36,666(22,904 to 58,294) | |  | 108.23(107.07 to 109.41) | |  | 209(75 to 479) | |  | 0.63(0.54 to 0.73) |
| Ukraine | Endometriosis |  | 9,336(5,666 to 14,469) | |  | 91.81(89.86 to 93.79) | |  | 55(19 to 128) | |  | 0.55(0.40 to 0.73) |
|  | Unexplained infertility |  | 688,457(337,132 to 1,308,650) | |  | 6667.96(6651.60 to 6684.36) | |  | 3,958(1,355 to 10,580) | |  | 38.90(37.65 to 40.19) |
|  | Polycystic ovarian syndrome |  | 9,431(5,805 to 14,891) | |  | 93.39(91.41 to 95.41) | |  | 55(20 to 127) | |  | 0.56(0.41 to 0.74) |
| Brunei Darussalam | Endometriosis |  | 59(29 to 104) | |  | 44.96(34.23 to 58.36) | |  | 0(0 to 1) | |  | 0.26(0.00 to 4.27) |
|  | Unexplained infertility |  | 952(109 to 3,398) | |  | 703.09(659.12 to 749.55) | |  | 5(0 to 23) | |  | 3.89(1.31 to 9.53) |
|  | Polycystic ovarian syndrome |  | 1,251(760 to 1,914) | |  | 951.31(899.20 to 1005.95) | |  | 7(3 to 15) | |  | 5.41(2.18 to 11.65) |
| Japan | Endometriosis |  | 13,293(7,570 to 21,468) | |  | 50.71(49.83 to 51.61) | |  | 74(24 to 183) | |  | 0.29(0.22 to 0.37) |
|  | Unexplained infertility |  | 424,422(37,954 to 1,273,566) | |  | 1455.13(1450.70 to 1459.58) | |  | 2,308(141 to 8,862) | |  | 7.94(7.61 to 8.27) |
|  | Polycystic ovarian syndrome |  | 435,775(284,373 to 624,168) | |  | 1700.92(1695.73 to 1706.13) | |  | 2,419(938 to 5,096) | |  | 9.58(9.20 to 9.99) |
| Republic of Korea | Endometriosis |  | 5,769(2,951 to 9,590) | |  | 45.96(44.75 to 47.19) | |  | 33(10 to 80) | |  | 0.26(0.18 to 0.39) |
|  | Unexplained infertility |  | 112,151(10,787 to 388,446) | |  | 792.58(787.89 to 797.29) | |  | 615(40 to 2,563) | |  | 4.36(4.02 to 4.74) |
|  | Polycystic ovarian syndrome |  | 102,152(61,895 to 156,164) | |  | 831.48(826.28 to 836.72) | |  | 572(218 to 1,200) | |  | 4.72(4.33 to 5.14) |
| Singapore | Endometriosis |  | 704(360 to 1,174) | |  | 40.96(37.81 to 44.40) | |  | 4(1 to 10) | |  | 0.23(0.06 to 0.87) |
|  | Unexplained infertility |  | 18,299(1,566 to 64,247) | |  | 864.78(852.04 to 877.76) | |  | 100(6 to 403) | |  | 4.75(3.84 to 5.95) |
|  | Polycystic ovarian syndrome |  | 16,220(9,923 to 24,460) | |  | 963.55(947.84 to 979.55) | |  | 90(35 to 195) | |  | 5.44(4.31 to 6.90) |
| Australia | Endometriosis |  | 2,008(926 to 3,679) | |  | 32.44(31.02 to 33.90) | |  | 12(4 to 29) | |  | 0.19(0.10 to 0.35) |
|  | Unexplained infertility |  | 16,214(4,511 to 69,965) | |  | 249.15(245.31 to 253.05) | |  | 95(15 to 440) | |  | 1.47(1.19 to 1.81) |
|  | Polycystic ovarian syndrome |  | 63,368(38,134 to 99,889) | |  | 1016.77(1008.80 to 1024.79) | |  | 370(135 to 802) | |  | 5.99(5.39 to 6.65) |
| New Zealand | Endometriosis |  | 614(327 to 1,025) | |  | 50.57(46.64 to 54.77) | |  | 4(1 to 9) | |  | 0.30(0.08 to 0.86) |
|  | Unexplained infertility |  | 7,732(1,326 to 29,990) | |  | 622.79(608.97 to 636.88) | |  | 44(5 to 190) | |  | 3.58(2.60 to 4.84) |
|  | Polycystic ovarian syndrome |  | 17,955(11,377 to 26,601) | |  | 1469.73(1448.24 to 1491.48) | |  | 105(40 to 227) | |  | 8.60(7.02 to 10.45) |
| Andorra | Endometriosis |  | 8(5 to 13) | |  | 39.78(16.51 to 86.23) | |  | 0(0 to 0) | |  | 0.24(0.00 to 31.13) |
|  | Unexplained infertility |  | 495(139 to 1,018) | |  | 2142.72(1952.54 to 2350.63) | |  | 3(1 to 8) | |  | 12.52(2.27 to 48.16) |
|  | Polycystic ovarian syndrome |  | 250(167 to 365) | |  | 1208.33(1056.29 to 1379.65) | |  | 1(1 to 3) | |  | 7.36(0.42 to 41.85) |
| Austria | Endometriosis |  | 1,402(889 to 2,072) | |  | 68.46(64.88 to 72.22) | |  | 8(3 to 19) | |  | 0.41(0.18 to 0.85) |
|  | Unexplained infertility |  | 126,855(56,158 to 232,867) | |  | 5877.34(5844.89 to 5909.97) | |  | 716(218 to 1,735) | |  | 33.34(30.93 to 35.92) |
|  | Polycystic ovarian syndrome |  | 29,858(19,132 to 44,917) | |  | 1447.20(1430.56 to 1464.01) | |  | 174(68 to 398) | |  | 8.53(7.29 to 9.95) |
| Belgium | Endometriosis |  | 1,567(941 to 2,529) | |  | 61.36(58.32 to 64.52) | |  | 9(3 to 24) | |  | 0.37(0.17 to 0.72) |
|  | Unexplained infertility |  | 135,219(58,389 to 247,336) | |  | 5047.97(5020.95 to 5075.11) | |  | 771(230 to 1,932) | |  | 28.97(26.95 to 31.12) |
|  | Polycystic ovarian syndrome |  | 33,341(21,916 to 49,521) | |  | 1305.92(1291.77 to 1320.20) | |  | 197(76 to 443) | |  | 7.83(6.76 to 9.03) |
| Cyprus | Endometriosis |  | 164(97 to 269) | |  | 42.48(36.04 to 50.05) | |  | 1(0 to 2) | |  | 0.26(0.01 to 2.29) |
|  | Unexplained infertility |  | 9,763(2,994 to 20,762) | |  | 2230.99(2186.31 to 2276.65) | |  | 57(13 to 158) | |  | 13.07(9.84 to 17.43) |
|  | Polycystic ovarian syndrome |  | 4,408(2,801 to 6,518) | |  | 1138.00(1103.51 to 1173.54) | |  | 26(10 to 59) | |  | 6.95(4.48 to 10.67) |
| Denmark | Endometriosis |  | 388(206 to 654) | |  | 30.50(27.53 to 33.73) | |  | 2(1 to 6) | |  | 0.19(0.03 to 0.68) |
|  | Unexplained infertility |  | 8,206(718 to 22,725) | |  | 641.28(627.43 to 655.38) | |  | 51(4 to 163) | |  | 3.97(2.95 to 5.26) |
|  | Polycystic ovarian syndrome |  | 12,146(8,060 to 18,067) | |  | 953.11(936.14 to 970.32) | |  | 77(31 to 168) | |  | 6.04(4.76 to 7.59) |
| Finland | Endometriosis |  | 578(331 to 915) | |  | 49.22(45.26 to 53.48) | |  | 4(1 to 8) | |  | 0.30(0.07 to 0.89) |
|  | Unexplained infertility |  | 21,050(5,178 to 45,662) | |  | 1679.45(1656.73 to 1702.43) | |  | 123(23 to 360) | |  | 9.89(8.21 to 11.85) |
|  | Polycystic ovarian syndrome |  | 12,917(8,487 to 19,518) | |  | 1098.98(1079.93 to 1118.30) | |  | 78(30 to 172) | |  | 6.75(5.32 to 8.47) |
| France | Endometriosis |  | 6,777(4,186 to 10,962) | |  | 47.58(46.44 to 48.74) | |  | 42(15 to 103) | |  | 0.30(0.21 to 0.41) |
|  | Unexplained infertility |  | 625,853(259,355 to 1,114,550) | |  | 4236.42(4225.85 to 4247.01) | |  | 3,762(1,065 to 9,699) | |  | 25.68(24.86 to 26.53) |
|  | Polycystic ovarian syndrome |  | 168,051(107,234 to 251,316) | |  | 1174.38(1168.70 to 1180.08) | |  | 1,040(417 to 2,415) | |  | 7.36(6.91 to 7.83) |
| Germany | Endometriosis |  | 8,843(5,189 to 14,190) | |  | 49.23(48.19 to 50.28) | |  | 53(18 to 127) | |  | 0.30(0.22 to 0.40) |
|  | Unexplained infertility |  | 220,847(38,085 to 558,906) | |  | 1138.65(1133.88 to 1143.44) | |  | 1,282(163 to 4,215) | |  | 6.65(6.29 to 7.03) |
|  | Polycystic ovarian syndrome |  | 174,556(118,132 to 263,700) | |  | 966.98(962.39 to 971.59) | |  | 1,050(423 to 2,335) | |  | 5.89(5.53 to 6.27) |
| Greece | Endometriosis |  | 930(540 to 1,515) | |  | 42.34(39.56 to 45.27) | |  | 6(2 to 14) | |  | 0.26(0.09 to 0.63) |
|  | Unexplained infertility |  | 51,813(11,610 to 113,114) | |  | 2069.82(2051.53 to 2088.26) | |  | 297(53 to 832) | |  | 12.01(10.64 to 13.53) |
|  | Polycystic ovarian syndrome |  | 26,679(17,609 to 38,987) | |  | 1201.55(1186.62 to 1216.64) | |  | 159(63 to 364) | |  | 7.30(6.17 to 8.60) |
| Iceland | Endometriosis |  | 23(14 to 38) | |  | 28.41(18.04 to 43.11) | |  | 0(0 to 0) | |  | 0.17(0.00 to 6.12) |
|  | Unexplained infertility |  | 1,956(557 to 4,219) | |  | 2319.10(2217.27 to 2424.80) | |  | 11(3 to 31) | |  | 13.48(6.80 to 24.61) |
|  | Polycystic ovarian syndrome |  | 1,042(692 to 1,516) | |  | 1268.70(1192.52 to 1348.85) | |  | 6(3 to 14) | |  | 7.70(2.89 to 17.19) |
| Ireland | Endometriosis |  | 517(302 to 846) | |  | 43.97(40.17 to 48.06) | |  | 3(1 to 7) | |  | 0.27(0.05 to 0.84) |
|  | Unexplained infertility |  | 30,327(9,074 to 64,436) | |  | 2286.62(2260.40 to 2313.11) | |  | 174(38 to 487) | |  | 13.23(11.30 to 15.44) |
|  | Polycystic ovarian syndrome |  | 13,985(9,343 to 20,676) | |  | 1177.77(1157.81 to 1198.01) | |  | 83(32 to 189) | |  | 7.15(5.66 to 8.94) |
| Israel | Endometriosis |  | 1,270(735 to 2,042) | |  | 57.53(54.40 to 60.79) | |  | 8(3 to 19) | |  | 0.35(0.15 to 0.70) |
|  | Unexplained infertility |  | 39,816(9,916 to 90,331) | |  | 1758.11(1740.85 to 1775.50) | |  | 229(44 to 669) | |  | 10.13(8.85 to 11.54) |
|  | Polycystic ovarian syndrome |  | 23,064(15,235 to 34,455) | |  | 1041.87(1028.43 to 1055.44) | |  | 138(54 to 313) | |  | 6.27(5.27 to 7.42) |
| Italy | Endometriosis |  | 4,688(2,901 to 6,923) | |  | 39.90(38.73 to 41.09) | |  | 28(10 to 66) | |  | 0.24(0.16 to 0.36) |
|  | Unexplained infertility |  | 281,020(34,316 to 762,230) | |  | 2194.22(2185.98 to 2202.50) | |  | 1,588(175 to 5,375) | |  | 12.49(11.87 to 13.13) |
|  | Polycystic ovarian syndrome |  | 283,581(191,245 to 406,208) | |  | 2345.40(2336.54 to 2354.28) | |  | 1,656(679 to 3,820) | |  | 13.93(13.25 to 14.64) |
| Luxembourg | Endometriosis |  | 74(43 to 121) | |  | 45.01(35.21 to 57.23) | |  | 0(0 to 1) | |  | 0.28(0.00 to 4.21) |
|  | Unexplained infertility |  | 3,592(905 to 8,029) | |  | 2017.94(1952.05 to 2085.97) | |  | 21(4 to 60) | |  | 11.75(7.23 to 18.81) |
|  | Polycystic ovarian syndrome |  | 1,986(1,295 to 2,965) | |  | 1205.75(1152.42 to 1261.33) | |  | 12(5 to 27) | |  | 7.37(3.75 to 13.71) |
| Malta | Endometriosis |  | 34(21 to 54) | |  | 34.11(23.44 to 48.99) | |  | 0(0 to 0) | |  | 0.21(0.00 to 6.67) |
|  | Unexplained infertility |  | 2,516(762 to 5,322) | |  | 2315.91(2225.56 to 2409.82) | |  | 14(3 to 39) | |  | 13.42(7.37 to 23.82) |
|  | Polycystic ovarian syndrome |  | 1,229(811 to 1,793) | |  | 1229.01(1159.89 to 1301.90) | |  | 7(3 to 17) | |  | 7.47(3.01 to 16.70) |
| Netherlands | Endometriosis |  | 1,641(965 to 2,621) | |  | 44.07(41.95 to 46.27) | |  | 10(3 to 24) | |  | 0.27(0.13 to 0.51) |
|  | Unexplained infertility |  | 88,185(25,914 to 189,032) | |  | 2328.13(2312.75 to 2343.58) | |  | 513(121 to 1,407) | |  | 13.58(12.42 to 14.82) |
|  | Polycystic ovarian syndrome |  | 40,612(26,783 to 60,082) | |  | 1087.40(1076.78 to 1098.11) | |  | 245(94 to 553) | |  | 6.62(5.81 to 7.51) |
| Norway | Endometriosis |  | 484(285 to 771) | |  | 39.36(35.91 to 43.08) | |  | 3(1 to 7) | |  | 0.24(0.05 to 0.78) |
|  | Unexplained infertility |  | 46,117(14,503 to 93,940) | |  | 3643.23(3609.97 to 3676.75) | |  | 268(64 to 728) | |  | 21.24(18.77 to 23.99) |
|  | Polycystic ovarian syndrome |  | 13,863(8,864 to 20,867) | |  | 1128.40(1109.56 to 1147.51) | |  | 83(33 to 191) | |  | 6.85(5.45 to 8.53) |
| Portugal | Endometriosis |  | 707(431 to 1,129) | |  | 30.15(27.90 to 32.56) | |  | 4(1 to 10) | |  | 0.19(0.05 to 0.51) |
|  | Unexplained infertility |  | 55,230(15,934 to 118,061) | |  | 2112.04(2093.99 to 2130.23) | |  | 319(76 to 845) | |  | 12.34(10.98 to 13.83) |
|  | Polycystic ovarian syndrome |  | 26,646(17,486 to 38,931) | |  | 1118.67(1104.79 to 1132.69) | |  | 158(63 to 356) | |  | 6.79(5.74 to 8.00) |
| Spain | Endometriosis |  | 3,116(1,905 to 4,733) | |  | 30.67(29.55 to 31.82) | |  | 18(6 to 45) | |  | 0.18(0.11 to 0.30) |
|  | Unexplained infertility |  | 136,108(18,766 to 358,903) | |  | 1156.27(1149.94 to 1162.64) | |  | 772(79 to 2,442) | |  | 6.62(6.15 to 7.13) |
|  | Polycystic ovarian syndrome |  | 103,168(68,116 to 150,208) | |  | 1012.29(1005.84 to 1018.78) | |  | 603(237 to 1,337) | |  | 6.06(5.56 to 6.59) |
| Sweden | Endometriosis |  | 1,187(726 to 1,906) | |  | 52.94(49.94 to 56.10) | |  | 7(3 to 17) | |  | 0.32(0.13 to 0.69) |
|  | Unexplained infertility |  | 100,556(38,852 to 194,729) | |  | 4308.65(4281.93 to 4335.51) | |  | 580(162 to 1,605) | |  | 25.01(23.01 to 27.16) |
|  | Polycystic ovarian syndrome |  | 21,190(13,520 to 32,007) | |  | 946.80(933.93 to 959.83) | |  | 126(48 to 294) | |  | 5.71(4.74 to 6.83) |
| Switzerland | Endometriosis |  | 1,106(647 to 1,763) | |  | 53.74(50.56 to 57.10) | |  | 7(2 to 16) | |  | 0.33(0.12 to 0.76) |
|  | Unexplained infertility |  | 49,304(13,913 to 107,443) | |  | 2208.92(2189.30 to 2228.70) | |  | 285(63 to 824) | |  | 12.87(11.40 to 14.51) |
|  | Polycystic ovarian syndrome |  | 21,966(14,436 to 32,268) | |  | 1067.50(1053.15 to 1082.02) | |  | 131(51 to 298) | |  | 6.49(5.41 to 7.76) |
| United Kingdom | Endometriosis |  | 7,569(4,669 to 11,797) | |  | 48.70(47.60 to 49.82) | |  | 46(17 to 107) | |  | 0.30(0.22 to 0.40) |
|  | Unexplained infertility |  | 455,080(93,421 to 1,045,815) | |  | 2770.86(2762.79 to 2778.95) | |  | 2,647(454 to 8,093) | |  | 16.22(15.60 to 16.85) |
|  | Polycystic ovarian syndrome |  | 202,395(130,836 to 301,074) | |  | 1286.11(1280.47 to 1291.77) | |  | 1,218(476 to 2,778) | |  | 7.82(7.38 to 8.28) |
| Argentina | Endometriosis |  | 4,534(2,708 to 7,216) | |  | 37.33(36.25 to 38.43) | |  | 27(9 to 67) | |  | 0.22(0.15 to 0.32) |
|  | Unexplained infertility |  | 261,639(63,094 to 579,074) | |  | 2128.18(2120.02 to 2136.36) | |  | 1,502(262 to 4,187) | |  | 12.24(11.63 to 12.88) |
|  | Polycystic ovarian syndrome |  | 75,729(48,181 to 116,278) | |  | 628.68(624.20 to 633.18) | |  | 446(169 to 1,006) | |  | 3.72(3.38 to 4.08) |
| Chile | Endometriosis |  | 2,902(1,880 to 4,333) | |  | 59.11(56.97 to 61.31) | |  | 17(6 to 40) | |  | 0.34(0.20 to 0.56) |
|  | Unexplained infertility |  | 106,501(24,098 to 238,349) | |  | 2167.32(2154.31 to 2180.39) | |  | 607(107 to 1,783) | |  | 12.38(11.41 to 13.41) |
|  | Polycystic ovarian syndrome |  | 35,638(22,206 to 54,009) | |  | 736.85(729.19 to 744.57) | |  | 210(76 to 476) | |  | 4.36(3.79 to 5.00) |
| Uruguay | Endometriosis |  | 391(229 to 618) | |  | 46.03(41.56 to 50.86) | |  | 2(1 to 5) | |  | 0.27(0.04 to 0.96) |
|  | Unexplained infertility |  | 18,948(4,254 to 41,382) | |  | 2204.48(2173.11 to 2236.20) | |  | 108(19 to 324) | |  | 12.62(10.35 to 15.27) |
|  | Polycystic ovarian syndrome |  | 5,764(3,602 to 8,922) | |  | 688.14(670.43 to 706.21) | |  | 34(12 to 75) | |  | 4.06(2.80 to 5.71) |
| Canada | Endometriosis |  | 2,994(1,710 to 4,755) | |  | 34.71(33.47 to 35.99) | |  | 18(6 to 43) | |  | 0.21(0.12 to 0.34) |
|  | Unexplained infertility |  | 81,972(8,231 to 216,141) | |  | 954.59(948.03 to 961.20) | |  | 494(40 to 1,648) | |  | 5.79(5.28 to 6.33) |
|  | Polycystic ovarian syndrome |  | 51,131(32,283 to 77,475) | |  | 606.59(601.29 to 611.93) | |  | 309(119 to 702) | |  | 3.69(3.29 to 4.14) |
| United States of America | Endometriosis |  | 24,843(15,819 to 37,121) | |  | 32.05(31.65 to 32.45) | |  | 149(55 to 344) | |  | 0.19(0.16 to 0.23) |
|  | Unexplained infertility |  | 1,396,795(208,173 to 3,438,035) | |  | 1801.03(1798.04 to 1804.03) | |  | 8,276(1,005 to 26,434) | |  | 10.71(10.48 to 10.94) |
|  | Polycystic ovarian syndrome |  | 1,001,935(677,296 to 1,442,418) | |  | 1311.21(1308.64 to 1313.79) | |  | 6,006(2,341 to 13,245) | |  | 7.90(7.70 to 8.10) |
| Antigua and Barbuda | Endometriosis |  | 10(6 to 15) | |  | 41.03(19.56 to 77.78) | |  | 0(0 to 0) | |  | 0.24(0.00 to 19.97) |
|  | Unexplained infertility |  | 1,447(740 to 2,618) | |  | 5833.59(5536.04 to 6144.30) | |  | 8(3 to 22) | |  | 33.03(14.37 to 67.05) |
|  | Polycystic ovarian syndrome |  | 142(90 to 220) | |  | 597.27(502.60 to 705.84) | |  | 1(0 to 2) | |  | 3.49(0.04 to 25.52) |
| Bahamas | Endometriosis |  | 42(25 to 65) | |  | 39.46(28.42 to 53.46) | |  | 0(0 to 1) | |  | 0.23(0.00 to 4.16) |
|  | Unexplained infertility |  | 6,013(3,031 to 11,082) | |  | 5611.07(5469.95 to 5754.99) | |  | 34(11 to 88) | |  | 31.79(22.00 to 44.53) |
|  | Polycystic ovarian syndrome |  | 714(451 to 1,099) | |  | 672.33(623.82 to 723.68) | |  | 4(2 to 10) | |  | 3.92(1.10 to 10.02) |
| Barbados | Endometriosis |  | 28(17 to 44) | |  | 40.28(26.74 to 58.74) | |  | 0(0 to 0) | |  | 0.24(0.00 to 6.97) |
|  | Unexplained infertility |  | 4,146(2,113 to 7,506) | |  | 5761.16(5586.28 to 5940.52) | |  | 23(8 to 61) | |  | 32.59(20.69 to 49.39) |
|  | Polycystic ovarian syndrome |  | 456(287 to 683) | |  | 657.29(597.80 to 721.43) | |  | 3(1 to 7) | |  | 3.85(0.67 to 12.77) |
| Belize | Endometriosis |  | 54(33 to 85) | |  | 45.10(33.86 to 59.05) | |  | 0(0 to 1) | |  | 0.27(0.00 to 4.04) |
|  | Unexplained infertility |  | 3,321(1,311 to 6,349) | |  | 2835.83(2739.91 to 2934.35) | |  | 19(5 to 49) | |  | 16.42(9.91 to 25.74) |
|  | Polycystic ovarian syndrome |  | 745(461 to 1,127) | |  | 615.74(572.13 to 661.92) | |  | 4(2 to 11) | |  | 3.67(1.09 to 9.27) |
| Cuba | Endometriosis |  | 1,123(665 to 1,731) | |  | 46.11(43.43 to 48.93) | |  | 7(2 to 16) | |  | 0.27(0.10 to 0.60) |
|  | Unexplained infertility |  | 144,318(74,438 to 263,135) | |  | 5849.15(5818.92 to 5879.51) | |  | 811(269 to 2,112) | |  | 32.97(30.73 to 35.34) |
|  | Polycystic ovarian syndrome |  | 15,451(9,794 to 23,930) | |  | 647.27(636.99 to 657.68) | |  | 89(34 to 205) | |  | 3.77(3.02 to 4.66) |
| Dominica | Endometriosis |  | 7(4 to 11) | |  | 42.65(17.07 to 88.72) | |  | 0(0 to 0) | |  | 0.25(0.00 to 24.37) |
|  | Unexplained infertility |  | 929(474 to 1,730) | |  | 5666.31(5307.50 to 6043.37) | |  | 5(2 to 13) | |  | 32.12(10.80 to 74.21) |
|  | Polycystic ovarian syndrome |  | 100(61 to 153) | |  | 609.08(495.30 to 741.60) | |  | 1(0 to 1) | |  | 3.57(0.01 to 30.64) |
| Dominican Republic | Endometriosis |  | 1,502(895 to 2,371) | |  | 51.08(48.53 to 53.74) | |  | 9(3 to 22) | |  | 0.30(0.13 to 0.57) |
|  | Unexplained infertility |  | 139,681(63,324 to 270,113) | |  | 4817.41(4792.15 to 4842.77) | |  | 781(253 to 2,052) | |  | 26.89(25.03 to 28.85) |
|  | Polycystic ovarian syndrome |  | 17,549(10,882 to 27,136) | |  | 599.82(590.96 to 608.77) | |  | 102(39 to 235) | |  | 3.46(2.82 to 4.21) |
| Grenada | Endometriosis |  | 11(7 to 18) | |  | 44.15(22.25 to 79.40) | |  | 0(0 to 0) | |  | 0.26(0.00 to 17.00) |
|  | Unexplained infertility |  | 1,439(743 to 2,680) | |  | 5798.34(5501.51 to 6107.47) | |  | 8(3 to 21) | |  | 32.83(14.28 to 65.15) |
|  | Polycystic ovarian syndrome |  | 140(88 to 213) | |  | 552.53(464.51 to 652.98) | |  | 1(0 to 2) | |  | 3.24(0.04 to 22.33) |
| Guyana | Endometriosis |  | 98(59 to 157) | |  | 47.45(38.46 to 58.02) | |  | 1(0 to 1) | |  | 0.28(0.00 to 2.62) |
|  | Unexplained infertility |  | 15,888(8,842 to 27,705) | |  | 8025.75(7900.55 to 8152.50) | |  | 90(31 to 228) | |  | 45.11(36.20 to 55.62) |
|  | Polycystic ovarian syndrome |  | 1,201(767 to 1,855) | |  | 587.56(554.59 to 622.07) | |  | 7(3 to 16) | |  | 3.43(1.37 to 7.23) |
| Haiti | Endometriosis |  | 1,841(1,096 to 2,910) | |  | 51.28(48.96 to 53.69) | |  | 11(4 to 26) | |  | 0.29(0.14 to 0.54) |
|  | Unexplained infertility |  | 113,714(84,161 to 142,353) | |  | 3150.18(3131.88 to 3168.56) | |  | 638(251 to 1,400) | |  | 17.66(16.31 to 19.09) |
|  | Polycystic ovarian syndrome |  | 13,216(8,516 to 20,145) | |  | 366.67(360.44 to 373.00) | |  | 75(28 to 168) | |  | 2.07(1.63 to 2.61) |
| Jamaica | Endometriosis |  | 348(204 to 552) | |  | 43.88(39.38 to 48.77) | |  | 2(1 to 5) | |  | 0.26(0.03 to 0.97) |
|  | Unexplained infertility |  | 54,434(29,719 to 95,285) | |  | 6887.15(6829.33 to 6945.37) | |  | 307(104 to 761) | |  | 38.83(34.60 to 43.45) |
|  | Polycystic ovarian syndrome |  | 4,621(2,923 to 7,249) | |  | 592.34(575.33 to 609.73) | |  | 27(10 to 62) | |  | 3.46(2.28 to 5.07) |
| Saint Lucia | Endometriosis |  | 19(11 to 29) | |  | 40.80(24.37 to 65.05) | |  | 0(0 to 0) | |  | 0.24(0.00 to 10.84) |
|  | Unexplained infertility |  | 2,726(1,358 to 5,025) | |  | 5834.79(5617.07 to 6059.50) | |  | 15(5 to 39) | |  | 33.00(18.58 to 55.27) |
|  | Polycystic ovarian syndrome |  | 256(163 to 389) | |  | 569.84(501.65 to 645.36) | |  | 1(1 to 3) | |  | 3.34(0.23 to 15.97) |
| Saint Vincent and the Grenadines | Endometriosis |  | 12(7 to 19) | |  | 44.31(23.04 to 77.57) | |  | 0(0 to 0) | |  | 0.26(0.00 to 15.04) |
|  | Unexplained infertility |  | 1,608(800 to 2,958) | |  | 5783.71(5503.83 to 6074.47) | |  | 9(3 to 24) | |  | 32.71(14.99 to 62.56) |
|  | Polycystic ovarian syndrome |  | 160(100 to 248) | |  | 582.82(495.83 to 681.02) | |  | 1(0 to 2) | |  | 3.41(0.07 to 20.77) |
| Suriname | Endometriosis |  | 74(44 to 117) | |  | 51.06(40.07 to 64.19) | |  | 0(0 to 1) | |  | 0.30(0.00 to 3.31) |
|  | Unexplained infertility |  | 8,403(4,131 to 15,027) | |  | 5775.85(5652.95 to 5900.81) | |  | 47(15 to 126) | |  | 32.66(24.03 to 43.45) |
|  | Polycystic ovarian syndrome |  | 867(550 to 1,340) | |  | 602.46(562.98 to 644.02) | |  | 5(2 to 12) | |  | 3.51(1.15 to 8.26) |
| Trinidad and Tobago | Endometriosis |  | 152(90 to 240) | |  | 44.37(37.49 to 52.24) | |  | 1(0 to 2) | |  | 0.26(0.00 to 1.83) |
|  | Unexplained infertility |  | 26,027(13,452 to 46,485) | |  | 7110.50(7023.12 to 7198.78) | |  | 146(48 to 383) | |  | 40.30(33.95 to 47.61) |
|  | Polycystic ovarian syndrome |  | 2,210(1,422 to 3,377) | |  | 653.27(625.84 to 681.66) | |  | 13(5 to 30) | |  | 3.85(2.01 to 6.77) |
| Bolivia (Plurinational State of) | Endometriosis |  | 1,457(777 to 2,329) | |  | 45.91(43.58 to 48.34) | |  | 8(2 to 20) | |  | 0.26(0.11 to 0.52) |
|  | Unexplained infertility |  | 24,344(2,796 to 79,116) | |  | 782.76(772.94 to 792.67) | |  | 135(10 to 513) | |  | 4.34(3.64 to 5.14) |
|  | Polycystic ovarian syndrome |  | 28,322(17,756 to 43,145) | |  | 895.45(885.04 to 905.96) | |  | 158(63 to 367) | |  | 4.99(4.25 to 5.84) |
| Ecuador | Endometriosis |  | 1,734(959 to 2,932) | |  | 36.21(34.52 to 37.95) | |  | 10(3 to 24) | |  | 0.20(0.10 to 0.38) |
|  | Unexplained infertility |  | 34,628(4,370 to 118,287) | |  | 737.09(729.34 to 744.90) | |  | 191(16 to 751) | |  | 4.07(3.51 to 4.69) |
|  | Polycystic ovarian syndrome |  | 50,105(32,078 to 74,259) | |  | 1049.89(1040.70 to 1059.13) | |  | 281(105 to 648) | |  | 5.88(5.22 to 6.62) |
| Peru | Endometriosis |  | 3,686(1,995 to 6,012) | |  | 37.14(35.95 to 38.36) | |  | 20(6 to 49) | |  | 0.21(0.13 to 0.32) |
|  | Unexplained infertility |  | 121,873(10,651 to 372,425) | |  | 1227.72(1220.84 to 1234.64) | |  | 665(49 to 2,449) | |  | 6.69(6.20 to 7.23) |
|  | Polycystic ovarian syndrome |  | 97,638(63,167 to 148,494) | |  | 989.23(983.03 to 995.46) | |  | 537(207 to 1,236) | |  | 5.45(5.00 to 5.93) |
| Colombia | Endometriosis |  | 4,853(2,883 to 7,687) | |  | 36.01(35.01 to 37.04) | |  | 29(10 to 73) | |  | 0.22(0.14 to 0.31) |
|  | Unexplained infertility |  | 58,268(13,249 to 111,088) | |  | 430.77(427.28 to 434.29) | |  | 352(64 to 971) | |  | 2.60(2.34 to 2.89) |
|  | Polycystic ovarian syndrome |  | 92,922(61,416 to 133,349) | |  | 693.62(689.17 to 698.10) | |  | 551(226 to 1,250) | |  | 4.12(3.78 to 4.48) |
| Costa Rica | Endometriosis |  | 505(297 to 804) | |  | 37.73(34.50 to 41.20) | |  | 3(1 to 7) | |  | 0.22(0.04 to 0.69) |
|  | Unexplained infertility |  | 49,838(20,850 to 100,255) | |  | 3619.29(3587.56 to 3651.26) | |  | 275(77 to 768) | |  | 20.04(17.74 to 22.58) |
|  | Polycystic ovarian syndrome |  | 12,726(7,940 to 19,463) | |  | 958.44(941.80 to 975.31) | |  | 72(27 to 169) | |  | 5.45(4.26 to 6.89) |
| El Salvador | Endometriosis |  | 651(364 to 1,032) | |  | 35.82(33.12 to 38.70) | |  | 4(1 to 9) | |  | 0.20(0.05 to 0.55) |
|  | Unexplained infertility |  | 26,372(4,332 to 64,834) | |  | 1514.85(1496.56 to 1533.31) | |  | 146(18 to 426) | |  | 8.34(7.03 to 9.82) |
|  | Polycystic ovarian syndrome |  | 14,149(9,008 to 22,118) | |  | 787.05(774.09 to 800.18) | |  | 80(31 to 183) | |  | 4.43(3.51 to 5.53) |
| Guatemala | Endometriosis |  | 1,980(1,157 to 3,184) | |  | 44.66(42.70 to 46.70) | |  | 11(4 to 28) | |  | 0.25(0.12 to 0.46) |
|  | Unexplained infertility |  | 133,608(51,054 to 283,637) | |  | 3212.24(3194.95 to 3229.61) | |  | 726(201 to 1,956) | |  | 17.39(16.14 to 18.72) |
|  | Polycystic ovarian syndrome |  | 32,272(19,542 to 50,391) | |  | 727.09(719.11 to 735.14) | |  | 178(69 to 405) | |  | 4.00(3.43 to 4.64) |
| Honduras | Endometriosis |  | 1,248(719 to 1,979) | |  | 43.33(40.94 to 45.84) | |  | 7(2 to 16) | |  | 0.24(0.10 to 0.52) |
|  | Unexplained infertility |  | 95,108(38,407 to 188,330) | |  | 3492.79(3470.50 to 3515.20) | |  | 518(145 to 1,463) | |  | 18.98(17.37 to 20.71) |
|  | Polycystic ovarian syndrome |  | 21,109(13,216 to 32,224) | |  | 733.63(723.69 to 743.68) | |  | 118(44 to 276) | |  | 4.07(3.37 to 4.90) |
| Mexico | Endometriosis |  | 15,715(9,267 to 24,385) | |  | 44.88(44.18 to 45.59) | |  | 88(32 to 213) | |  | 0.25(0.20 to 0.31) |
|  | Unexplained infertility |  | 1,614,547(684,946 to 3,192,541) | |  | 4584.91(4577.84 to 4591.99) | |  | 8,765(2,542 to 23,460) | |  | 24.90(24.38 to 25.43) |
|  | Polycystic ovarian syndrome |  | 433,619(278,289 to 638,066) | |  | 1241.70(1238.00 to 1245.40) | |  | 2,410(933 to 5,541) | |  | 6.91(6.63 to 7.19) |
| Nicaragua | Endometriosis |  | 728(406 to 1,170) | |  | 39.31(36.50 to 42.28) | |  | 4(1 to 10) | |  | 0.22(0.06 to 0.58) |
|  | Unexplained infertility |  | 32,873(8,145 to 74,925) | |  | 1797.90(1778.50 to 1817.47) | |  | 182(33 to 553) | |  | 9.95(8.56 to 11.52) |
|  | Polycystic ovarian syndrome |  | 13,924(8,701 to 20,713) | |  | 754.45(741.96 to 767.11) | |  | 79(31 to 187) | |  | 4.28(3.38 to 5.34) |
| Panama | Endometriosis |  | 440(255 to 701) | |  | 41.00(37.25 to 45.02) | |  | 2(1 to 6) | |  | 0.23(0.04 to 0.75) |
|  | Unexplained infertility |  | 45,747(19,728 to 90,415) | |  | 4325.89(4286.31 to 4365.73) | |  | 248(72 to 661) | |  | 23.41(20.59 to 26.52) |
|  | Polycystic ovarian syndrome |  | 8,875(5,654 to 13,529) | |  | 830.53(813.32 to 848.01) | |  | 49(19 to 115) | |  | 4.61(3.41 to 6.09) |
| Venezuela (Bolivarian Republic of) | Endometriosis |  | 2,781(1,648 to 4,447) | |  | 41.44(39.89 to 43.03) | |  | 16(6 to 38) | |  | 0.24(0.13 to 0.39) |
|  | Unexplained infertility |  | 213,844(77,431 to 440,384) | |  | 2984.76(2972.00 to 2997.57) | |  | 1,182(294 to 3,296) | |  | 16.59(15.64 to 17.57) |
|  | Polycystic ovarian syndrome |  | 59,496(38,035 to 91,247) | |  | 880.53(873.37 to 887.73) | |  | 335(128 to 759) | |  | 4.98(4.46 to 5.56) |
| Brazil | Endometriosis |  | 31,740(19,138 to 49,768) | |  | 53.27(52.69 to 53.87) | |  | 188(68 to 456) | |  | 0.32(0.27 to 0.37) |
|  | Unexplained infertility |  | 2,125,447(887,052 to 4,170,154) | |  | 3389.50(3384.93 to 3394.08) | |  | 12,032(3,755 to 32,845) | |  | 19.26(18.91 to 19.61) |
|  | Polycystic ovarian syndrome |  | 130,648(81,750 to 204,118) | |  | 220.52(219.31 to 221.72) | |  | 765(285 to 1,757) | |  | 1.30(1.21 to 1.40) |
| Paraguay | Endometriosis |  | 957(577 to 1,505) | |  | 50.13(46.99 to 53.43) | |  | 6(2 to 14) | |  | 0.29(0.10 to 0.68) |
|  | Unexplained infertility |  | 90,276(48,834 to 157,990) | |  | 4792.58(4761.32 to 4824.00) | |  | 508(173 to 1,290) | |  | 26.93(24.63 to 29.39) |
|  | Polycystic ovarian syndrome |  | 4,610(2,910 to 7,340) | |  | 241.38(234.45 to 248.48) | |  | 27(10 to 63) | |  | 1.40(0.92 to 2.06) |
| Algeria | Endometriosis |  | 8,834(5,292 to 13,735) | |  | 76.57(74.97 to 78.20) | |  | 52(18 to 125) | |  | 0.45(0.33 to 0.59) |
|  | Unexplained infertility |  | 411,463(180,229 to 736,498) | |  | 3456.43(3445.82 to 3467.08) | |  | 2,345(671 to 6,014) | |  | 19.80(19.00 to 20.62) |
|  | Polycystic ovarian syndrome |  | 98,991(61,606 to 153,136) | |  | 877.25(871.74 to 882.78) | |  | 574(216 to 1,364) | |  | 5.11(4.70 to 5.55) |
| Bahrain | Endometriosis |  | 208(126 to 325) | |  | 62.42(54.21 to 71.60) | |  | 1(0 to 3) | |  | 0.37(0.02 to 1.96) |
|  | Unexplained infertility |  | 12,863(5,953 to 22,979) | |  | 3789.84(3724.49 to 3856.13) | |  | 74(21 to 189) | |  | 21.81(17.11 to 27.49) |
|  | Polycystic ovarian syndrome |  | 3,124(1,990 to 4,788) | |  | 954.90(921.59 to 989.16) | |  | 18(7 to 40) | |  | 5.57(3.30 to 8.88) |
| Egypt | Endometriosis |  | 21,656(13,234 to 33,842) | |  | 83.20(82.09 to 84.32) | |  | 131(46 to 310) | |  | 0.50(0.42 to 0.60) |
|  | Unexplained infertility |  | 989,412(442,270 to 1,672,172) | |  | 3803.81(3796.31 to 3811.32) | |  | 5,851(1,686 to 14,635) | |  | 22.44(21.87 to 23.03) |
|  | Polycystic ovarian syndrome |  | 241,586(151,115 to 369,130) | |  | 921.84(918.16 to 925.53) | |  | 1,454(569 to 3,236) | |  | 5.53(5.25 to 5.83) |
| Iran (Islamic Republic of) | Endometriosis |  | 15,576(9,779 to 24,467) | |  | 64.40(63.36 to 65.45) | |  | 98(37 to 232) | |  | 0.41(0.33 to 0.51) |
|  | Unexplained infertility |  | 815,742(367,319 to 1,472,829) | |  | 3319.80(3312.38 to 3327.24) | |  | 5,125(1,526 to 14,546) | |  | 21.20(20.60 to 21.81) |
|  | Polycystic ovarian syndrome |  | 204,001(129,069 to 312,290) | |  | 869.51(865.62 to 873.42) | |  | 1,279(496 to 2,854) | |  | 5.53(5.22 to 5.85) |
| Iraq | Endometriosis |  | 8,555(5,160 to 13,575) | |  | 82.09(80.35 to 83.85) | |  | 51(18 to 121) | |  | 0.48(0.36 to 0.64) |
|  | Unexplained infertility |  | 385,454(183,787 to 661,420) | |  | 3810.76(3798.68 to 3822.87) | |  | 2,230(689 to 5,519) | |  | 21.93(21.03 to 22.87) |
|  | Polycystic ovarian syndrome |  | 86,175(53,899 to 135,044) | |  | 814.41(808.95 to 819.90) | |  | 506(195 to 1,130) | |  | 4.76(4.35 to 5.20) |
| Jordan | Endometriosis |  | 2,758(1,644 to 4,375) | |  | 90.83(87.46 to 94.31) | |  | 16(6 to 39) | |  | 0.53(0.30 to 0.87) |
|  | Unexplained infertility |  | 99,625(74,090 to 127,976) | |  | 3348.80(3327.99 to 3369.72) | |  | 574(221 to 1,255) | |  | 19.23(17.68 to 20.88) |
|  | Polycystic ovarian syndrome |  | 25,341(16,329 to 37,820) | |  | 816.96(806.87 to 827.14) | |  | 150(56 to 348) | |  | 4.80(4.05 to 5.64) |
| Kuwait | Endometriosis |  | 990(604 to 1,525) | |  | 63.51(59.41 to 67.90) | |  | 6(2 to 13) | |  | 0.37(0.12 to 1.01) |
|  | Unexplained infertility |  | 62,231(27,749 to 113,601) | |  | 3656.27(3626.19 to 3686.61) | |  | 352(100 to 908) | |  | 20.99(18.75 to 23.51) |
|  | Polycystic ovarian syndrome |  | 15,825(10,218 to 24,420) | |  | 1069.42(1051.58 to 1087.55) | |  | 91(35 to 212) | |  | 6.24(4.94 to 7.86) |
| Lebanon | Endometriosis |  | 1,322(791 to 2,029) | |  | 85.25(80.63 to 90.09) | |  | 8(3 to 18) | |  | 0.49(0.20 to 1.04) |
|  | Unexplained infertility |  | 86,054(42,323 to 153,793) | |  | 5436.71(5399.89 to 5473.75) | |  | 482(148 to 1,184) | |  | 30.61(27.89 to 33.54) |
|  | Polycystic ovarian syndrome |  | 14,138(8,993 to 22,175) | |  | 948.36(932.46 to 964.50) | |  | 81(30 to 184) | |  | 5.44(4.30 to 6.83) |
| Libya | Endometriosis |  | 1,285(796 to 2,014) | |  | 64.23(60.75 to 67.87) | |  | 8(3 to 19) | |  | 0.38(0.16 to 0.78) |
|  | Unexplained infertility |  | 69,077(30,788 to 125,656) | |  | 3353.37(3328.31 to 3378.59) | |  | 400(114 to 1,031) | |  | 19.52(17.65 to 21.55) |
|  | Polycystic ovarian syndrome |  | 17,419(11,005 to 26,800) | |  | 880.03(866.95 to 893.27) | |  | 103(39 to 237) | |  | 5.22(4.25 to 6.34) |
| Morocco | Endometriosis |  | 6,521(3,965 to 10,218) | |  | 66.96(65.34 to 68.60) | |  | 39(14 to 97) | |  | 0.40(0.29 to 0.55) |
|  | Unexplained infertility |  | 430,242(216,983 to 739,243) | |  | 4383.16(4370.07 to 4396.29) | |  | 2,533(769 to 6,271) | |  | 25.85(24.85 to 26.88) |
|  | Polycystic ovarian syndrome |  | 79,303(49,427 to 125,424) | |  | 819.44(813.75 to 825.17) | |  | 472(180 to 1,100) | |  | 4.89(4.46 to 5.35) |
| Palestine | Endometriosis |  | 1,131(692 to 1,756) | |  | 87.74(82.62 to 93.11) | |  | 7(2 to 16) | |  | 0.51(0.20 to 1.14) |
|  | Unexplained infertility |  | 48,573(23,815 to 84,700) | |  | 3953.37(3917.79 to 3989.22) | |  | 281(81 to 662) | |  | 22.69(20.08 to 25.57) |
|  | Polycystic ovarian syndrome |  | 10,299(6,526 to 15,991) | |  | 782.09(766.81 to 797.62) | |  | 61(23 to 136) | |  | 4.58(3.48 to 5.94) |
| Oman | Endometriosis |  | 751(456 to 1,180) | |  | 70.13(65.12 to 75.46) | |  | 4(2 to 11) | |  | 0.41(0.12 to 1.09) |
|  | Unexplained infertility |  | 43,442(19,846 to 76,751) | |  | 3910.79(3873.62 to 3948.25) | |  | 247(71 to 610) | |  | 22.39(19.65 to 25.44) |
|  | Polycystic ovarian syndrome |  | 9,964(6,194 to 15,316) | |  | 959.71(940.59 to 979.15) | |  | 58(22 to 129) | |  | 5.59(4.22 to 7.30) |
| Qatar | Endometriosis |  | 449(271 to 691) | |  | 74.27(67.12 to 82.14) | |  | 3(1 to 6) | |  | 0.44(0.06 to 1.84) |
|  | Unexplained infertility |  | 24,658(10,605 to 45,057) | |  | 3818.90(3768.63 to 3869.84) | |  | 140(39 to 343) | |  | 21.87(18.19 to 26.27) |
|  | Polycystic ovarian syndrome |  | 6,015(3,727 to 9,270) | |  | 1052.45(1023.87 to 1081.75) | |  | 35(13 to 77) | |  | 6.14(4.13 to 8.98) |
| Saudi Arabia | Endometriosis |  | 6,638(4,000 to 10,242) | |  | 61.97(60.47 to 63.51) | |  | 38(14 to 92) | |  | 0.36(0.26 to 0.50) |
|  | Unexplained infertility |  | 432,939(194,918 to 767,797) | |  | 3865.43(3853.81 to 3877.09) | |  | 2,460(692 to 6,189) | |  | 22.10(21.23 to 23.01) |
|  | Polycystic ovarian syndrome |  | 106,442(67,079 to 164,797) | |  | 1032.67(1026.34 to 1039.02) | |  | 614(234 to 1,382) | |  | 6.00(5.52 to 6.51) |
| Syrian Arab Republic | Endometriosis |  | 2,632(1,636 to 4,131) | |  | 74.67(71.66 to 77.79) | |  | 15(5 to 38) | |  | 0.43(0.23 to 0.76) |
|  | Unexplained infertility |  | 131,615(59,933 to 235,991) | |  | 3518.08(3498.16 to 3538.10) | |  | 747(211 to 1,874) | |  | 19.98(18.51 to 21.57) |
|  | Polycystic ovarian syndrome |  | 30,701(19,931 to 47,623) | |  | 810.14(800.51 to 819.88) | |  | 177(68 to 396) | |  | 4.68(3.97 to 5.50) |
| Tunisia | Endometriosis |  | 2,200(1,382 to 3,428) | |  | 70.30(67.35 to 73.34) | |  | 13(5 to 32) | |  | 0.42(0.22 to 0.74) |
|  | Unexplained infertility |  | 119,052(56,105 to 220,149) | |  | 3636.91(3616.01 to 3657.91) | |  | 691(201 to 1,703) | |  | 21.30(19.72 to 22.98) |
|  | Polycystic ovarian syndrome |  | 25,536(16,283 to 38,825) | |  | 835.41(825.04 to 845.89) | |  | 150(58 to 345) | |  | 4.95(4.18 to 5.83) |
| Türkiye | Endometriosis |  | 14,985(9,305 to 23,521) | |  | 68.61(67.51 to 69.72) | |  | 87(32 to 204) | |  | 0.40(0.32 to 0.50) |
|  | Unexplained infertility |  | 811,791(352,581 to 1,511,078) | |  | 3641.63(3633.68 to 3649.60) | |  | 4,638(1,301 to 11,914) | |  | 20.87(20.27 to 21.48) |
|  | Polycystic ovarian syndrome |  | 165,967(106,960 to 254,559) | |  | 772.95(769.22 to 776.70) | |  | 965(364 to 2,221) | |  | 4.52(4.23 to 4.81) |
| United Arab Emirates | Endometriosis |  | 1,047(642 to 1,648) | |  | 59.66(55.56 to 64.04) | |  | 6(2 to 14) | |  | 0.35(0.11 to 0.95) |
|  | Unexplained infertility |  | 87,699(38,397 to 164,375) | |  | 3935.86(3905.39 to 3966.58) | |  | 490(132 to 1,216) | |  | 22.47(20.19 to 25.00) |
|  | Polycystic ovarian syndrome |  | 16,676(10,582 to 26,045) | |  | 968.74(951.93 to 985.81) | |  | 94(35 to 214) | |  | 5.63(4.41 to 7.15) |
| Yemen | Endometriosis |  | 8,209(4,937 to 12,724) | |  | 100.95(98.75 to 103.18) | |  | 48(18 to 114) | |  | 0.59(0.43 to 0.79) |
|  | Unexplained infertility |  | 428,501(231,588 to 717,519) | |  | 5273.09(5257.22 to 5289.01) | |  | 2,488(781 to 6,133) | |  | 30.48(29.28 to 31.71) |
|  | Polycystic ovarian syndrome |  | 45,319(28,419 to 71,909) | |  | 537.12(532.11 to 542.17) | |  | 267(100 to 618) | |  | 3.15(2.78 to 3.56) |
| Afghanistan | Endometriosis |  | 7,645(4,563 to 12,344) | |  | 113.03(110.39 to 115.72) | |  | 43(16 to 104) | |  | 0.63(0.45 to 0.87) |
|  | Unexplained infertility |  | 103,774(65,690 to 142,851) | |  | 1592.02(1581.90 to 1602.18) | |  | 586(211 to 1,340) | |  | 8.88(8.14 to 9.67) |
|  | Polycystic ovarian syndrome |  | 36,127(23,385 to 53,826) | |  | 503.87(498.40 to 509.39) | |  | 205(80 to 468) | |  | 2.82(2.43 to 3.27) |
| Bangladesh | Endometriosis |  | 26,264(15,609 to 41,704) | |  | 56.39(55.71 to 57.08) | |  | 152(50 to 360) | |  | 0.33(0.28 to 0.38) |
|  | Unexplained infertility |  | 1,506,087(1,190,413 to 1,810,954) | |  | 3133.65(3128.64 to 3138.66) | |  | 8,782(3,426 to 17,322) | |  | 18.25(17.87 to 18.64) |
|  | Polycystic ovarian syndrome |  | 108,080(67,774 to 168,131) | |  | 232.39(231.00 to 233.78) | |  | 635(239 to 1,428) | |  | 1.36(1.26 to 1.47) |
| Bhutan | Endometriosis |  | 119(73 to 188) | |  | 55.70(46.11 to 66.82) | |  | 1(0 to 2) | |  | 0.33(0.00 to 2.74) |
|  | Unexplained infertility |  | 6,049(2,539 to 11,297) | |  | 2794.34(2724.27 to 2865.88) | |  | 35(10 to 85) | |  | 16.20(11.28 to 22.71) |
|  | Polycystic ovarian syndrome |  | 779(494 to 1,171) | |  | 368.32(342.85 to 395.30) | |  | 5(2 to 10) | |  | 2.15(0.65 to 5.47) |
| India | Endometriosis |  | 241,978(150,639 to 374,643) | |  | 63.54(63.28 to 63.79) | |  | 1,397(520 to 3,359) | |  | 0.37(0.35 to 0.39) |
|  | Unexplained infertility |  | 29,075,289(16,070,794 to 49,483,699) | |  | 7614.03(7611.26 to 7616.80) | |  | 165,263(58,813 to 403,773) | |  | 43.24(43.04 to 43.45) |
|  | Polycystic ovarian syndrome |  | 1,887,785(1,201,309 to 2,870,098) | |  | 496.11(495.41 to 496.82) | |  | 10,828(4,060 to 24,878) | |  | 2.84(2.79 to 2.90) |
| Nepal | Endometriosis |  | 4,811(2,974 to 7,602) | |  | 51.26(49.81 to 52.74) | |  | 29(10 to 71) | |  | 0.31(0.21 to 0.45) |
|  | Unexplained infertility |  | 150,430(109,902 to 194,827) | |  | 1536.90(1529.12 to 1544.71) | |  | 907(350 to 1,934) | |  | 9.24(8.65 to 9.86) |
|  | Polycystic ovarian syndrome |  | 21,608(13,656 to 32,355) | |  | 229.63(226.57 to 232.73) | |  | 132(50 to 302) | |  | 1.40(1.17 to 1.66) |
| Pakistan | Endometriosis |  | 53,994(33,386 to 84,488) | |  | 88.22(87.47 to 88.97) | |  | 334(118 to 774) | |  | 0.54(0.49 to 0.61) |
|  | Unexplained infertility |  | 4,817,423(1,352,930 to 10,740,041) | |  | 7834.15(7827.13 to 7841.18) | |  | 29,137(5,823 to 82,480) | |  | 47.20(46.66 to 47.75) |
|  | Polycystic ovarian syndrome |  | 180,165(114,617 to 273,654) | |  | 292.21(290.86 to 293.57) | |  | 1,110(418 to 2,603) | |  | 1.79(1.69 to 1.90) |
| Angola | Endometriosis |  | 6,112(3,714 to 9,372) | |  | 82.81(80.72 to 84.96) | |  | 35(13 to 89) | |  | 0.47(0.32 to 0.66) |
|  | Unexplained infertility |  | 533,943(287,662 to 941,956) | |  | 7712.55(7691.65 to 7733.50) | |  | 2,939(994 to 7,228) | |  | 42.16(40.63 to 43.73) |
|  | Polycystic ovarian syndrome |  | 23,085(14,183 to 36,230) | |  | 299.94(296.00 to 303.92) | |  | 131(48 to 296) | |  | 1.69(1.40 to 2.01) |
| Central African Republic | Endometriosis |  | 977(589 to 1,528) | |  | 73.51(68.90 to 78.38) | |  | 6(2 to 14) | |  | 0.42(0.15 to 1.00) |
|  | Unexplained infertility |  | 148,169(90,817 to 237,833) | |  | 11621.61(11561.84 to 11681.64) | |  | 842(308 to 1,967) | |  | 65.45(61.06 to 70.11) |
|  | Polycystic ovarian syndrome |  | 3,308(2,070 to 5,262) | |  | 239.77(231.50 to 248.29) | |  | 19(7 to 45) | |  | 1.39(0.83 to 2.23) |
| Congo | Endometriosis |  | 869(553 to 1,349) | |  | 61.60(57.56 to 65.86) | |  | 5(2 to 13) | |  | 0.34(0.11 to 0.84) |
|  | Unexplained infertility |  | 87,057(41,048 to 161,490) | |  | 6274.82(6233.17 to 6316.69) | |  | 468(138 to 1,222) | |  | 33.66(30.67 to 36.87) |
|  | Polycystic ovarian syndrome |  | 4,574(2,847 to 7,234) | |  | 318.35(309.16 to 327.76) | |  | 25(10 to 56) | |  | 1.76(1.14 to 2.62) |
| Democratic Republic of the Congo | Endometriosis |  | 15,035(9,235 to 23,535) | |  | 72.89(71.71 to 74.09) | |  | 86(30 to 202) | |  | 0.41(0.33 to 0.51) |
|  | Unexplained infertility |  | 862,739(409,645 to 1,644,018) | |  | 4525.62(4515.94 to 4535.31) | |  | 4,771(1,407 to 11,831) | |  | 24.83(24.12 to 25.56) |
|  | Polycystic ovarian syndrome |  | 54,250(34,098 to 84,918) | |  | 253.08(250.91 to 255.27) | |  | 309(116 to 715) | |  | 1.42(1.27 to 1.60) |
| Equatorial Guinea | Endometriosis |  | 223(136 to 350) | |  | 62.05(54.05 to 71.06) | |  | 1(0 to 3) | |  | 0.35(0.02 to 2.10) |
|  | Unexplained infertility |  | 26,891(14,679 to 47,461) | |  | 7902.09(7806.90 to 7998.28) | |  | 148(49 to 358) | |  | 43.13(36.39 to 50.92) |
|  | Polycystic ovarian syndrome |  | 1,630(1,009 to 2,552) | |  | 442.66(421.03 to 465.26) | |  | 9(3 to 21) | |  | 2.48(1.13 to 5.00) |
| Gabon | Endometriosis |  | 265(159 to 412) | |  | 55.23(48.72 to 62.43) | |  | 2(1 to 4) | |  | 0.31(0.02 to 1.49) |
|  | Unexplained infertility |  | 46,817(26,562 to 77,282) | |  | 10249.42(10156.03 to 10343.51) | |  | 258(87 to 651) | |  | 56.23(49.52 to 63.64) |
|  | Polycystic ovarian syndrome |  | 1,967(1,227 to 3,147) | |  | 400.46(382.73 to 418.85) | |  | 11(4 to 26) | |  | 2.25(1.11 to 4.13) |
| Burundi | Endometriosis |  | 2,307(1,381 to 3,612) | |  | 75.17(72.08 to 78.39) | |  | 13(5 to 32) | |  | 0.42(0.22 to 0.78) |
|  | Unexplained infertility |  | 20,729(11,395 to 31,123) | |  | 695.09(685.53 to 704.78) | |  | 116(39 to 271) | |  | 3.87(3.18 to 4.68) |
|  | Polycystic ovarian syndrome |  | 5,348(3,476 to 8,286) | |  | 169.69(165.06 to 174.43) | |  | 30(11 to 70) | |  | 0.95(0.63 to 1.40) |
| Comoros | Endometriosis |  | 97(59 to 155) | |  | 50.19(40.68 to 61.34) | |  | 1(0 to 1) | |  | 0.30(0.00 to 2.69) |
|  | Unexplained infertility |  | 17,948(10,106 to 28,707) | |  | 9423.35(9285.50 to 9562.79) | |  | 104(37 to 252) | |  | 54.51(44.52 to 66.14) |
|  | Polycystic ovarian syndrome |  | 610(383 to 977) | |  | 310.75(286.48 to 336.61) | |  | 4(1 to 8) | |  | 1.83(0.45 to 5.08) |
| Djibouti | Endometriosis |  | 196(120 to 307) | |  | 59.71(51.62 to 68.77) | |  | 1(0 to 3) | |  | 0.33(0.01 to 1.93) |
|  | Unexplained infertility |  | 36,059(20,121 to 58,772) | |  | 10718.40(10607.89 to 10829.84) | |  | 197(69 to 495) | |  | 58.73(50.81 to 67.62) |
|  | Polycystic ovarian syndrome |  | 1,156(729 to 1,872) | |  | 355.46(335.21 to 376.68) | |  | 6(2 to 15) | |  | 1.98(0.76 to 4.33) |
| Eritrea | Endometriosis |  | 909(542 to 1,434) | |  | 55.45(51.87 to 59.23) | |  | 5(2 to 13) | |  | 0.32(0.10 to 0.77) |
|  | Unexplained infertility |  | 142,798(79,663 to 237,797) | |  | 8931.56(8884.96 to 8978.36) | |  | 800(281 to 1,869) | |  | 49.76(46.34 to 53.37) |
|  | Polycystic ovarian syndrome |  | 3,905(2,421 to 6,328) | |  | 233.45(226.10 to 240.99) | |  | 22(8 to 52) | |  | 1.33(0.83 to 2.04) |
| Ethiopia | Endometriosis |  | 16,232(9,999 to 25,266) | |  | 59.91(58.97 to 60.86) | |  | 94(35 to 223) | |  | 0.34(0.27 to 0.42) |
|  | Unexplained infertility |  | 992,154(476,708 to 1,804,689) | |  | 3840.96(3833.28 to 3848.65) | |  | 5,550(1,862 to 13,298) | |  | 21.27(20.71 to 21.85) |
|  | Polycystic ovarian syndrome |  | 65,877(41,714 to 103,375) | |  | 235.08(233.24 to 236.93) | |  | 379(146 to 867) | |  | 1.34(1.20 to 1.48) |
| Kenya | Endometriosis |  | 6,967(4,317 to 10,897) | |  | 53.44(52.17 to 54.74) | |  | 39(14 to 94) | |  | 0.30(0.21 to 0.42) |
|  | Unexplained infertility |  | 617,522(314,620 to 1,122,348) | |  | 4965.15(4952.62 to 4977.70) | |  | 3,391(1,133 to 8,407) | |  | 27.09(26.18 to 28.03) |
|  | Polycystic ovarian syndrome |  | 39,724(24,898 to 62,754) | |  | 295.39(292.43 to 298.37) | |  | 223(83 to 503) | |  | 1.64(1.43 to 1.88) |
| Madagascar | Endometriosis |  | 3,974(2,321 to 6,328) | |  | 56.22(54.46 to 58.03) | |  | 23(8 to 56) | |  | 0.33(0.21 to 0.50) |
|  | Unexplained infertility |  | 355,631(185,469 to 597,454) | |  | 5227.13(5209.71 to 5244.60) | |  | 2,049(689 to 4,931) | |  | 29.82(28.53 to 31.17) |
|  | Polycystic ovarian syndrome |  | 16,202(10,075 to 26,384) | |  | 220.41(216.96 to 223.92) | |  | 95(37 to 213) | |  | 1.28(1.03 to 1.58) |
| Malawi | Endometriosis |  | 2,396(1,447 to 3,820) | |  | 48.90(46.92 to 50.95) | |  | 13(5 to 32) | |  | 0.27(0.14 to 0.48) |
|  | Unexplained infertility |  | 41,567(25,927 to 57,043) | |  | 790.97(783.29 to 798.72) | |  | 229(83 to 525) | |  | 4.34(3.79 to 4.96) |
|  | Polycystic ovarian syndrome |  | 13,079(8,531 to 19,364) | |  | 255.20(250.72 to 259.75) | |  | 72(27 to 165) | |  | 1.39(1.08 to 1.78) |
| Mauritius | Endometriosis |  | 180(110 to 280) | |  | 55.99(48.07 to 64.92) | |  | 1(0 to 3) | |  | 0.32(0.01 to 1.95) |
|  | Unexplained infertility |  | 12,586(4,867 to 24,531) | |  | 3862.18(3794.60 to 3930.73) | |  | 69(20 to 177) | |  | 21.25(16.52 to 27.01) |
|  | Polycystic ovarian syndrome |  | 4,241(2,737 to 6,429) | |  | 1329.34(1289.39 to 1370.27) | |  | 24(9 to 56) | |  | 7.59(4.85 to 11.39) |
| Mozambique | Endometriosis |  | 4,866(2,952 to 7,827) | |  | 66.86(64.94 to 68.84) | |  | 28(10 to 68) | |  | 0.38(0.25 to 0.56) |
|  | Unexplained infertility |  | 574,163(323,719 to 930,604) | |  | 8404.38(8382.10 to 8426.72) | |  | 3,195(1,126 to 7,628) | |  | 46.23(44.60 to 47.92) |
|  | Polycystic ovarian syndrome |  | 22,085(13,704 to 34,940) | |  | 287.20(283.28 to 291.17) | |  | 125(46 to 279) | |  | 1.61(1.33 to 1.94) |
| Rwanda | Endometriosis |  | 1,828(1,066 to 2,866) | |  | 52.14(49.76 to 54.61) | |  | 10(4 to 26) | |  | 0.30(0.14 to 0.56) |
|  | Unexplained infertility |  | 46,497(11,842 to 108,671) | |  | 1350.40(1338.08 to 1362.81) | |  | 259(47 to 723) | |  | 7.49(6.60 to 8.48) |
|  | Polycystic ovarian syndrome |  | 9,519(5,934 to 14,345) | |  | 265.17(259.82 to 270.62) | |  | 54(20 to 123) | |  | 1.49(1.12 to 1.97) |
| Seychelles | Endometriosis |  | 16(10 to 26) | |  | 66.73(38.16 to 109.33) | |  | 0(0 to 0) | |  | 0.38(0.00 to 18.45) |
|  | Unexplained infertility |  | 941(376 to 1,867) | |  | 3788.43(3549.34 to 4040.20) | |  | 5(1 to 13) | |  | 20.95(6.95 to 49.80) |
|  | Polycystic ovarian syndrome |  | 300(195 to 455) | |  | 1245.99(1108.22 to 1396.83) | |  | 2(1 to 4) | |  | 7.15(0.65 to 29.92) |
| Somalia | Endometriosis |  | 4,282(2,539 to 6,615) | |  | 92.95(90.13 to 95.84) | |  | 24(9 to 60) | |  | 0.53(0.33 to 0.80) |
|  | Unexplained infertility |  | 214,543(108,124 to 381,213) | |  | 4850.66(4829.86 to 4871.54) | |  | 1,200(393 to 2,921) | |  | 26.89(25.37 to 28.49) |
|  | Polycystic ovarian syndrome |  | 10,843(6,763 to 17,583) | |  | 221.04(216.78 to 225.37) | |  | 62(22 to 142) | |  | 1.25(0.96 to 1.63) |
| United Republic of Tanzania | Endometriosis |  | 10,417(6,290 to 16,274) | |  | 71.94(70.54 to 73.36) | |  | 57(21 to 144) | |  | 0.39(0.30 to 0.52) |
|  | Unexplained infertility |  | 501,885(417,743 to 597,445) | |  | 3638.99(3628.80 to 3649.21) | |  | 2,736(1,116 to 5,743) | |  | 19.76(19.02 to 20.53) |
|  | Polycystic ovarian syndrome |  | 45,458(28,547 to 69,435) | |  | 301.43(298.61 to 304.28) | |  | 250(90 to 595) | |  | 1.65(1.45 to 1.87) |
| Uganda | Endometriosis |  | 6,542(3,924 to 10,394) | |  | 64.42(62.82 to 66.05) | |  | 36(13 to 90) | |  | 0.35(0.25 to 0.50) |
|  | Unexplained infertility |  | 108,004(70,117 to 150,324) | |  | 1018.94(1012.78 to 1025.14) | |  | 595(211 to 1,301) | |  | 5.59(5.14 to 6.07) |
|  | Polycystic ovarian syndrome |  | 27,285(17,345 to 41,061) | |  | 255.66(252.52 to 258.83) | |  | 152(57 to 361) | |  | 1.41(1.19 to 1.67) |
| Zambia | Endometriosis |  | 2,842(1,747 to 4,442) | |  | 58.66(56.47 to 60.93) | |  | 16(6 to 38) | |  | 0.33(0.18 to 0.56) |
|  | Unexplained infertility |  | 187,200(84,708 to 350,078) | |  | 4104.39(4085.46 to 4123.39) | |  | 1,032(328 to 2,586) | |  | 22.47(21.09 to 23.93) |
|  | Polycystic ovarian syndrome |  | 17,474(10,689 to 27,397) | |  | 346.18(340.92 to 351.52) | |  | 98(35 to 224) | |  | 1.92(1.55 to 2.37) |
| Botswana | Endometriosis |  | 405(250 to 646) | |  | 57.69(52.20 to 63.64) | |  | 2(1 to 6) | |  | 0.32(0.05 to 1.17) |
|  | Unexplained infertility |  | 32,629(12,794 to 67,450) | |  | 4544.26(4495.01 to 4593.95) | |  | 175(48 to 477) | |  | 24.42(20.94 to 28.37) |
|  | Polycystic ovarian syndrome |  | 2,817(1,761 to 4,265) | |  | 404.75(389.91 to 420.04) | |  | 16(6 to 36) | |  | 2.23(1.26 to 3.70) |
| Lesotho | Endometriosis |  | 318(193 to 505) | |  | 62.37(55.62 to 69.82) | |  | 2(1 to 4) | |  | 0.36(0.04 to 1.64) |
|  | Unexplained infertility |  | 28,597(13,669 to 52,989) | |  | 5867.38(5798.95 to 5936.52) | |  | 160(51 to 420) | |  | 32.55(27.65 to 38.17) |
|  | Polycystic ovarian syndrome |  | 1,884(1,194 to 2,933) | |  | 362.81(346.39 to 379.92) | |  | 11(4 to 25) | |  | 2.08(1.02 to 3.95) |
| Namibia | Endometriosis |  | 424(247 to 670) | |  | 63.45(57.53 to 69.87) | |  | 2(1 to 6) | |  | 0.36(0.06 to 1.29) |
|  | Unexplained infertility |  | 19,808(6,571 to 42,758) | |  | 3034.73(2992.42 to 3077.52) | |  | 110(29 to 297) | |  | 16.86(13.85 to 20.38) |
|  | Polycystic ovarian syndrome |  | 2,202(1,439 to 3,386) | |  | 327.61(313.99 to 341.71) | |  | 13(5 to 30) | |  | 1.87(0.98 to 3.31) |
| South Africa | Endometriosis |  | 9,816(5,997 to 15,593) | |  | 61.33(60.12 to 62.56) | |  | 57(20 to 133) | |  | 0.36(0.27 to 0.46) |
|  | Unexplained infertility |  | 564,552(178,120 to 1,273,505) | |  | 3399.51(3390.63 to 3408.41) | |  | 3,152(772 to 8,670) | |  | 19.04(18.37 to 19.71) |
|  | Polycystic ovarian syndrome |  | 67,771(42,616 to 103,955) | |  | 427.98(424.75 to 431.23) | |  | 390(149 to 917) | |  | 2.47(2.23 to 2.73) |
| Eswatini | Endometriosis |  | 179(108 to 283) | |  | 55.26(47.39 to 64.26) | |  | 1(0 to 3) | |  | 0.32(0.01 to 2.27) |
|  | Unexplained infertility |  | 8,002(2,446 to 17,221) | |  | 2521.62(2466.27 to 2578.07) | |  | 45(11 to 130) | |  | 14.19(10.33 to 19.24) |
|  | Polycystic ovarian syndrome |  | 1,331(826 to 1,984) | |  | 409.32(387.42 to 432.33) | |  | 8(3 to 18) | |  | 2.36(0.99 to 5.07) |
| Zimbabwe | Endometriosis |  | 2,978(1,777 to 4,646) | |  | 74.01(71.36 to 76.75) | |  | 17(6 to 41) | |  | 0.42(0.24 to 0.69) |
|  | Unexplained infertility |  | 128,921(48,224 to 269,824) | |  | 3325.84(3307.61 to 3344.16) | |  | 703(191 to 1,787) | |  | 18.06(16.75 to 19.47) |
|  | Polycystic ovarian syndrome |  | 12,844(8,232 to 19,814) | |  | 311.43(306.01 to 316.94) | |  | 72(28 to 166) | |  | 1.73(1.35 to 2.20) |
| Benin | Endometriosis |  | 2,464(1,510 to 3,762) | |  | 77.41(74.31 to 80.61) | |  | 14(5 to 32) | |  | 0.42(0.23 to 0.75) |
|  | Unexplained infertility |  | 43,774(17,721 to 70,639) | |  | 1397.10(1383.77 to 1410.53) | |  | 241(73 to 639) | |  | 7.61(6.66 to 8.68) |
|  | Polycystic ovarian syndrome |  | 10,566(6,765 to 15,965) | |  | 321.32(315.06 to 327.68) | |  | 59(22 to 135) | |  | 1.77(1.34 to 2.32) |
| Burkina Faso | Endometriosis |  | 4,421(2,591 to 6,892) | |  | 81.89(79.44 to 84.41) | |  | 25(9 to 60) | |  | 0.46(0.29 to 0.70) |
|  | Unexplained infertility |  | 151,357(46,038 to 322,972) | |  | 2988.31(2972.98 to 3003.72) | |  | 833(182 to 2,292) | |  | 16.28(15.17 to 17.46) |
|  | Polycystic ovarian syndrome |  | 15,140(9,551 to 23,174) | |  | 269.22(264.83 to 273.67) | |  | 85(32 to 196) | |  | 1.50(1.19 to 1.88) |
| Cameroon | Endometriosis |  | 4,973(3,093 to 7,821) | |  | 64.36(62.55 to 66.22) | |  | 29(10 to 70) | |  | 0.37(0.25 to 0.55) |
|  | Unexplained infertility |  | 549,482(278,289 to 975,463) | |  | 7408.29(7388.42 to 7428.21) | |  | 3,112(999 to 8,522) | |  | 41.53(40.06 to 43.05) |
|  | Polycystic ovarian syndrome |  | 29,676(18,159 to 47,018) | |  | 371.56(367.25 to 375.92) | |  | 173(64 to 384) | |  | 2.14(1.82 to 2.50) |
| Cabo Verde | Endometriosis |  | 74(45 to 117) | |  | 47.86(37.54 to 60.37) | |  | 0(0 to 1) | |  | 0.26(0.00 to 3.51) |
|  | Unexplained infertility |  | 8,535(3,439 to 16,585) | |  | 5591.34(5472.84 to 5711.94) | |  | 46(13 to 111) | |  | 30.07(21.98 to 40.40) |
|  | Polycystic ovarian syndrome |  | 510(320 to 789) | |  | 333.55(305.12 to 364.11) | |  | 3(1 to 6) | |  | 1.84(0.35 to 5.97) |
| Chad | Endometriosis |  | 4,404(2,634 to 6,786) | |  | 120.17(116.54 to 123.90) | |  | 24(8 to 57) | |  | 0.65(0.41 to 1.01) |
|  | Unexplained infertility |  | 143,985(52,871 to 287,190) | |  | 4267.99(4245.42 to 4290.66) | |  | 777(194 to 1,844) | |  | 22.81(21.19 to 24.53) |
|  | Polycystic ovarian syndrome |  | 8,036(4,969 to 12,940) | |  | 205.32(200.67 to 210.06) | |  | 44(16 to 101) | |  | 1.11(0.80 to 1.54) |
| Côte d'Ivoire | Endometriosis |  | 4,869(2,921 to 7,556) | |  | 73.07(71.00 to 75.18) | |  | 27(10 to 65) | |  | 0.40(0.27 to 0.60) |
|  | Unexplained infertility |  | 405,916(192,551 to 762,142) | |  | 6326.08(6306.44 to 6345.77) | |  | 2,206(667 to 5,467) | |  | 34.20(32.78 to 35.68) |
|  | Polycystic ovarian syndrome |  | 21,004(13,077 to 32,632) | |  | 308.91(304.69 to 313.19) | |  | 117(42 to 274) | |  | 1.71(1.41 to 2.06) |
| Gambia | Endometriosis |  | 406(242 to 634) | |  | 66.93(60.43 to 74.06) | |  | 2(1 to 5) | |  | 0.38(0.05 to 1.57) |
|  | Unexplained infertility |  | 28,494(11,775 to 55,242) | |  | 5025.31(4965.97 to 5085.28) | |  | 156(45 to 394) | |  | 27.29(23.10 to 32.14) |
|  | Polycystic ovarian syndrome |  | 1,758(1,073 to 2,778) | |  | 279.44(266.16 to 293.33) | |  | 10(4 to 22) | |  | 1.56(0.73 to 3.13) |
| Ghana | Endometriosis |  | 4,741(2,883 to 7,415) | |  | 51.18(49.72 to 52.67) | |  | 27(9 to 63) | |  | 0.28(0.19 to 0.42) |
|  | Unexplained infertility |  | 377,924(137,289 to 822,057) | |  | 4250.66(4237.03 to 4264.33) | |  | 2,055(472 to 5,258) | |  | 23.02(22.02 to 24.04) |
|  | Polycystic ovarian syndrome |  | 26,742(17,123 to 41,794) | |  | 287.22(283.76 to 290.71) | |  | 149(53 to 335) | |  | 1.59(1.35 to 1.88) |
| Guinea | Endometriosis |  | 2,562(1,522 to 4,030) | |  | 77.60(74.57 to 80.74) | |  | 14(5 to 33) | |  | 0.43(0.23 to 0.75) |
|  | Unexplained infertility |  | 136,365(87,441 to 189,499) | |  | 4182.30(4159.80 to 4204.91) | |  | 750(271 to 1,702) | |  | 22.89(21.26 to 24.64) |
|  | Polycystic ovarian syndrome |  | 8,693(5,440 to 13,919) | |  | 254.90(249.46 to 260.45) | |  | 48(17 to 114) | |  | 1.40(1.02 to 1.89) |
| Guinea-Bissau | Endometriosis |  | 316(194 to 507) | |  | 59.98(53.44 to 67.22) | |  | 2(1 to 4) | |  | 0.34(0.03 to 1.65) |
|  | Unexplained infertility |  | 21,954(8,174 to 42,574) | |  | 4375.67(4317.22 to 4434.83) | |  | 121(31 to 293) | |  | 23.85(19.73 to 28.71) |
|  | Polycystic ovarian syndrome |  | 1,374(847 to 2,227) | |  | 255.10(241.55 to 269.34) | |  | 8(3 to 18) | |  | 1.43(0.60 to 3.12) |
| Liberia | Endometriosis |  | 786(493 to 1,224) | |  | 57.65(53.65 to 61.88) | |  | 4(2 to 11) | |  | 0.32(0.09 to 0.84) |
|  | Unexplained infertility |  | 87,235(40,516 to 163,086) | |  | 6627.31(6583.13 to 6671.72) | |  | 471(134 to 1,190) | |  | 35.62(32.45 to 39.03) |
|  | Polycystic ovarian syndrome |  | 4,030(2,514 to 6,425) | |  | 286.29(277.41 to 295.41) | |  | 22(8 to 51) | |  | 1.57(0.98 to 2.42) |
| Mali | Endometriosis |  | 5,034(3,104 to 7,944) | |  | 94.83(92.16 to 97.58) | |  | 28(10 to 68) | |  | 0.53(0.35 to 0.79) |
|  | Unexplained infertility |  | 109,179(60,461 to 158,688) | |  | 1883.59(1872.16 to 1895.08) | |  | 626(209 to 1,417) | |  | 10.69(9.85 to 11.60) |
|  | Polycystic ovarian syndrome |  | 12,658(7,942 to 20,353) | |  | 225.04(220.99 to 229.15) | |  | 72(27 to 172) | |  | 1.26(0.98 to 1.62) |
| Mauritania | Endometriosis |  | 673(407 to 1,058) | |  | 64.38(59.52 to 69.56) | |  | 4(1 to 9) | |  | 0.37(0.09 to 1.05) |
|  | Unexplained infertility |  | 58,064(26,277 to 107,048) | |  | 5838.81(5790.73 to 5887.22) | |  | 328(95 to 914) | |  | 32.61(29.12 to 36.43) |
|  | Polycystic ovarian syndrome |  | 3,648(2,223 to 5,715) | |  | 334.88(323.86 to 346.22) | |  | 21(8 to 49) | |  | 1.91(1.17 to 3.01) |
| Niger | Endometriosis |  | 7,114(4,267 to 11,378) | |  | 144.83(141.35 to 148.39) | |  | 40(14 to 97) | |  | 0.80(0.57 to 1.12) |
|  | Unexplained infertility |  | 231,243(98,432 to 427,363) | |  | 5084.52(5063.09 to 5106.03) | |  | 1,275(349 to 3,032) | |  | 27.63(26.08 to 29.26) |
|  | Polycystic ovarian syndrome |  | 11,112(6,877 to 18,381) | |  | 206.94(202.89 to 211.06) | |  | 63(24 to 149) | |  | 1.16(0.88 to 1.52) |
| Nigeria | Endometriosis |  | 44,150(27,009 to 68,268) | |  | 80.02(79.26 to 80.79) | |  | 252(89 to 592) | |  | 0.45(0.40 to 0.51) |
|  | Unexplained infertility |  | 1,892,833(719,710 to 3,940,193) | |  | 3663.66(3658.35 to 3668.98) | |  | 10,471(2,649 to 26,902) | |  | 20.08(19.69 to 20.48) |
|  | Polycystic ovarian syndrome |  | 165,579(104,722 to 258,417) | |  | 288.04(286.61 to 289.47) | |  | 947(365 to 2,166) | |  | 1.62(1.52 to 1.73) |
| Sao Tome and Principe | Endometriosis |  | 27(16 to 42) | |  | 48.25(31.57 to 71.30) | |  | 0(0 to 0) | |  | 0.27(0.00 to 9.02) |
|  | Unexplained infertility |  | 1,155(275 to 2,799) | |  | 2176.47(2052.12 to 2306.82) | |  | 6(1 to 18) | |  | 11.82(4.44 to 26.19) |
|  | Polycystic ovarian syndrome |  | 162(102 to 242) | |  | 285.37(242.69 to 334.02) | |  | 1(0 to 2) | |  | 1.58(0.03 to 11.17) |
| Senegal | Endometriosis |  | 2,584(1,577 to 4,060) | |  | 66.93(64.33 to 69.61) | |  | 15(5 to 36) | |  | 0.38(0.21 to 0.65) |
|  | Unexplained infertility |  | 117,792(73,765 to 167,570) | |  | 3221.63(3203.01 to 3240.34) | |  | 659(234 to 1,476) | |  | 17.87(16.51 to 19.32) |
|  | Polycystic ovarian syndrome |  | 10,835(6,895 to 16,724) | |  | 272.97(267.77 to 278.26) | |  | 62(23 to 146) | |  | 1.55(1.19 to 2.01) |
| Sierra Leone | Endometriosis |  | 1,557(934 to 2,455) | |  | 69.09(65.61 to 72.73) | |  | 9(3 to 21) | |  | 0.38(0.17 to 0.80) |
|  | Unexplained infertility |  | 137,490(61,834 to 251,113) | |  | 6585.11(6549.64 to 6620.76) | |  | 741(225 to 1,913) | |  | 35.25(32.71 to 37.97) |
|  | Polycystic ovarian syndrome |  | 6,653(4,072 to 10,567) | |  | 286.74(279.69 to 293.95) | |  | 37(13 to 86) | |  | 1.57(1.09 to 2.23) |
| Togo | Endometriosis |  | 1,360(831 to 2,146) | |  | 63.14(59.81 to 66.61) | |  | 8(3 to 19) | |  | 0.36(0.15 to 0.74) |
|  | Unexplained infertility |  | 80,306(28,385 to 163,791) | |  | 3814.70(3788.27 to 3841.29) | |  | 441(109 to 1,161) | |  | 20.89(18.98 to 22.96) |
|  | Polycystic ovarian syndrome |  | 5,769(3,622 to 9,271) | |  | 263.75(256.96 to 270.70) | |  | 33(12 to 73) | |  | 1.49(1.02 to 2.12) |
| American Samoa | Endometriosis |  | 10(6 to 15) | |  | 87.32(40.94 to 164.64) | |  | 0(0 to 0) | |  | 0.49(0.00 to 38.31) |
|  | Unexplained infertility |  | 450(190 to 851) | |  | 4069.28(3700.16 to 4466.35) | |  | 3(1 to 6) | |  | 22.69(3.73 to 75.27) |
|  | Polycystic ovarian syndrome |  | 112(71 to 170) | |  | 1007.58(828.55 to 1215.03) | |  | 1(0 to 1) | |  | 5.75(0.02 to 47.89) |
| Bermuda | Endometriosis |  | 5(3 to 9) | |  | 41.16(13.61 to 101.49) | |  | 0(0 to 0) | |  | 0.24(0.00 to 41.27) |
|  | Unexplained infertility |  | 824(404 to 1,502) | |  | 5787.12(5388.92 to 6210.97) | |  | 5(2 to 12) | |  | 32.64(9.57 to 88.46) |
|  | Polycystic ovarian syndrome |  | 97(61 to 152) | |  | 746.43(600.93 to 920.11) | |  | 1(0 to 1) | |  | 4.36(0.01 to 48.12) |
| Cook Islands | Endometriosis |  | 3(2 to 5) | |  | 77.29(17.29 to 222.93) | |  | 0(0 to 0) | |  | 0.44(0.00 to 97.60) |
|  | Unexplained infertility |  | 178(79 to 327) | |  | 4249.83(3646.73 to 4926.74) | |  | 1(0 to 3) | |  | 23.64(0.57 to 139.42) |
|  | Polycystic ovarian syndrome |  | 47(30 to 71) | |  | 1112.15(816.11 to 1483.68) | |  | 0(0 to 1) | |  | 6.30(0.00 to 108.73) |
| Greenland | Endometriosis |  | 5(3 to 8) | |  | 39.40(13.20 to 93.76) | |  | 0(0 to 0) | |  | 0.24(0.00 to 34.45) |
|  | Unexplained infertility |  | 132(13 to 356) | |  | 962.12(804.40 to 1143.97) | |  | 1(0 to 3) | |  | 5.86(0.07 to 43.98) |
|  | Polycystic ovarian syndrome |  | 72(45 to 108) | |  | 540.86(422.55 to 684.10) | |  | 0(0 to 1) | |  | 3.30(0.00 to 39.85) |
| Guam | Endometriosis |  | 31(19 to 49) | |  | 88.23(60.05 to 125.32) | |  | 0(0 to 0) | |  | 0.50(0.00 to 12.09) |
|  | Unexplained infertility |  | 1,400(539 to 2,632) | |  | 3987.53(3781.21 to 4202.35) | |  | 8(2 to 20) | |  | 22.18(9.44 to 44.40) |
|  | Polycystic ovarian syndrome |  | 398(253 to 620) | |  | 1122.39(1014.58 to 1238.74) | |  | 2(1 to 5) | |  | 6.40(0.93 to 22.05) |
| Monaco | Endometriosis |  | 3(2 to 5) | |  | 42.16(8.33 to 136.51) | |  | 0(0 to 0) | |  | 0.26(0.00 to 71.42) |
|  | Unexplained infertility |  | 172(50 to 351) | |  | 2163.80(1846.23 to 2527.64) | |  | 1(0 to 3) | |  | 12.60(0.27 to 90.70) |
|  | Polycystic ovarian syndrome |  | 89(59 to 132) | |  | 1226.17(978.86 to 1522.94) | |  | 1(0 to 1) | |  | 7.46(0.01 to 83.55) |
| Nauru | Endometriosis |  | 3(2 to 4) | |  | 101.17(19.24 to 327.23) | |  | 0(0 to 0) | |  | 0.58(0.00 to 167.54) |
|  | Unexplained infertility |  | 119(54 to 220) | |  | 4380.76(3626.70 to 5255.15) | |  | 1(0 to 2) | |  | 24.33(0.12 to 208.76) |
|  | Polycystic ovarian syndrome |  | 26(17 to 40) | |  | 923.50(603.08 to 1367.52) | |  | 0(0 to 0) | |  | 5.26(0.00 to 175.69) |
| Niue | Endometriosis |  | 0(0 to 0) | |  | 78.68(0.00 to 1213.31) | |  | 0(0 to 0) | |  | 0.45(0.00 to 1062.69) |
|  | Unexplained infertility |  | 16(7 to 30) | |  | 4319.07(2481.17 to 7021.96) | |  | 0(0 to 0) | |  | 23.94(0.00 to 1108.78) |
|  | Polycystic ovarian syndrome |  | 4(2 to 6) | |  | 1046.85(278.86 to 2751.29) | |  | 0(0 to 0) | |  | 5.95(0.00 to 1073.67) |
| Northern Mariana Islands | Endometriosis |  | 8(5 to 12) | |  | 74.18(31.16 to 151.65) | |  | 0(0 to 0) | |  | 0.42(0.00 to 42.76) |
|  | Unexplained infertility |  | 433(192 to 835) | |  | 4241.00(3848.75 to 4664.00) | |  | 2(1 to 6) | |  | 23.62(3.69 to 81.08) |
|  | Polycystic ovarian syndrome |  | 106(67 to 159) | |  | 997.87(814.01 to 1212.84) | |  | 1(0 to 1) | |  | 5.68(0.01 to 52.14) |
| Palau | Endometriosis |  | 3(2 to 4) | |  | 72.40(11.63 to 253.17) | |  | 0(0 to 0) | |  | 0.41(0.00 to 137.93) |
|  | Unexplained infertility |  | 161(66 to 305) | |  | 4296.79(3640.87 to 5047.37) | |  | 1(0 to 2) | |  | 23.83(0.33 to 176.74) |
|  | Polycystic ovarian syndrome |  | 37(23 to 56) | |  | 1045.20(727.06 to 1465.17) | |  | 0(0 to 0) | |  | 5.94(0.00 to 147.78) |
| Puerto Rico | Endometriosis |  | 285(175 to 449) | |  | 38.36(34.00 to 43.15) | |  | 2(1 to 4) | |  | 0.22(0.02 to 0.98) |
|  | Unexplained infertility |  | 42,735(21,025 to 78,105) | |  | 5664.04(5610.18 to 5718.32) | |  | 240(81 to 617) | |  | 31.95(28.02 to 36.32) |
|  | Polycystic ovarian syndrome |  | 6,143(3,956 to 9,300) | |  | 836.90(815.94 to 858.29) | |  | 36(13 to 85) | |  | 4.88(3.40 to 6.82) |
| Saint Kitts and Nevis | Endometriosis |  | 6(4 to 10) | |  | 40.27(15.19 to 89.20) | |  | 0(0 to 0) | |  | 0.24(0.00 to 29.76) |
|  | Unexplained infertility |  | 945(482 to 1,695) | |  | 5870.59(5501.44 to 6259.83) | |  | 5(2 to 14) | |  | 33.09(11.17 to 79.00) |
|  | Polycystic ovarian syndrome |  | 106(66 to 165) | |  | 686.49(561.56 to 832.67) | |  | 1(0 to 1) | |  | 4.00(0.01 to 36.35) |
| San Marino | Endometriosis |  | 3(2 to 5) | |  | 41.32(7.72 to 135.00) | |  | 0(0 to 0) | |  | 0.25(0.00 to 69.84) |
|  | Unexplained infertility |  | 166(48 to 355) | |  | 2202.49(1873.51 to 2578.51) | |  | 1(0 to 3) | |  | 12.86(0.25 to 90.32) |
|  | Polycystic ovarian syndrome |  | 85(57 to 126) | |  | 1190.62(943.89 to 1487.02) | |  | 1(0 to 1) | |  | 7.25(0.01 to 81.97) |
| Tokelau | Endometriosis |  | 0(0 to 1) | |  | 111.77(0.01 to 1475.17) | |  | 0(0 to 0) | |  | 0.64(0.00 to 1261.99) |
|  | Unexplained infertility |  | 13(6 to 25) | |  | 4252.75(2283.31 to 7264.48) | |  | 0(0 to 0) | |  | 23.67(0.00 to 1307.65) |
|  | Polycystic ovarian syndrome |  | 3(2 to 5) | |  | 951.88(196.02 to 2837.25) | |  | 0(0 to 0) | |  | 5.41(0.00 to 1271.48) |
| Tuvalu | Endometriosis |  | 2(1 to 4) | |  | 82.70(12.48 to 286.13) | |  | 0(0 to 0) | |  | 0.47(0.00 to 149.02) |
|  | Unexplained infertility |  | 119(54 to 219) | |  | 4349.43(3600.29 to 5212.44) | |  | 1(0 to 2) | |  | 24.20(0.12 to 192.79) |
|  | Polycystic ovarian syndrome |  | 25(16 to 38) | |  | 866.37(559.25 to 1288.96) | |  | 0(0 to 0) | |  | 4.93(0.00 to 157.32) |
| United States Virgin Islands | Endometriosis |  | 7(4 to 12) | |  | 44.11(17.97 to 92.84) | |  | 0(0 to 0) | |  | 0.26(0.00 to 28.45) |
|  | Unexplained infertility |  | 948(463 to 1,759) | |  | 5479.32(5132.63 to 5845.08) | |  | 5(2 to 14) | |  | 31.04(10.40 to 74.54) |
|  | Polycystic ovarian syndrome |  | 133(86 to 206) | |  | 803.41(670.99 to 955.97) | |  | 1(0 to 2) | |  | 4.69(0.04 to 36.12) |
| South Sudan | Endometriosis |  | 1,700(1,023 to 2,655) | |  | 78.50(74.76 to 82.40) | |  | 10(4 to 23) | |  | 0.44(0.21 to 0.85) |
|  | Unexplained infertility |  | 100,303(50,253 to 179,055) | |  | 4720.18(4690.72 to 4749.78) | |  | 561(169 to 1,378) | |  | 26.27(24.12 to 28.57) |
|  | Polycystic ovarian syndrome |  | 5,407(3,377 to 8,553) | |  | 232.44(226.13 to 238.89) | |  | 31(11 to 76) | |  | 1.32(0.89 to 1.91) |
| Sudan | Endometriosis |  | 10,733(6,359 to 16,191) | |  | 96.98(95.13 to 98.86) | |  | 60(21 to 148) | |  | 0.54(0.41 to 0.71) |
|  | Unexplained infertility |  | 757,022(396,287 to 1,294,129) | |  | 6972.99(6957.16 to 6988.85) | |  | 4,199(1,396 to 10,152) | |  | 38.50(37.33 to 39.70) |
|  | Polycystic ovarian syndrome | |  | 85,851(52,982 to 132,292) | |  | 754.69(749.58 to 759.83) | |  | 482(182 to 1,097) |  | 4.22(3.84 to 4.62) |

**Table S6:** Prevalence and YLDs cases and age-standardised rate of primary infertility attributable to endometriosis, PCOS and unexplained infertility for WCBA in 2021 by location.

| **location** | **Disease** |  | **Prevalence in 2021** | | |  | **YLDs (Years Lived with Disability) in 2021** | | |
| --- | --- | --- | --- | --- | --- | --- | --- | --- | --- |
|  |  |  | **Number of cases** |  | **ASR per 100,000 population** |  | **Number of cases** |  | **ASR per 100,000 population** |
| **Global** |  |  |  |  |  |  |  |  |  |
|  | Endometriosis |  | 312,363(155,324 to 585,826) |  | 16.21(16.15 to 16.26) |  | 2,341(818 to 5,541) |  | 0.12(0.12 to 0.13) |
|  | Unexplained infertility |  | 21,074,754(9,211,232 to 42,316,093) |  | 1087.58(1087.12 to 1088.05) |  | 157,158(48,072 to 389,165) |  | 8.11(8.07 to 8.15) |
|  | Polycystic ovarian syndrome |  | 3,452,839(1,753,946 to 6,317,755) |  | 179.84(179.65 to 180.03) |  | 25,668(9,502 to 56,325) |  | 1.34(1.32 to 1.35) |
| **GBD region** |  |  |  |  |  |  |  |  |  |
| East Asia | Endometriosis |  | 17,651(7,562 to 35,912) |  | 5.86(5.77 to 5.95) |  | 133(42 to 343) |  | 0.04(0.04 to 0.05) |
|  | Unexplained infertility |  | 2,230,090(1,038,544 to 4,134,396) |  | 692.17(691.24 to 693.10) |  | 16,681(5,117 to 37,816) |  | 5.18(5.10 to 5.26) |
|  | Polycystic ovarian syndrome |  | 267,149(116,525 to 527,521) |  | 96.40(96.03 to 96.78) |  | 1,984(627 to 4,880) |  | 0.72(0.68 to 0.75) |
| Southeast Asia | Endometriosis |  | 34,107(16,209 to 64,528) |  | 18.68(18.48 to 18.88) |  | 256(90 to 617) |  | 0.14(0.12 to 0.16) |
|  | Unexplained infertility |  | 2,241,327(884,659 to 4,742,340) |  | 1216.40(1214.81 to 1218.00) |  | 16,700(4,754 to 40,960) |  | 9.06(8.93 to 9.20) |
|  | Polycystic ovarian syndrome |  | 545,322(273,227 to 1,003,212) |  | 302.30(301.50 to 303.10) |  | 4,051(1,475 to 9,267) |  | 2.25(2.18 to 2.32) |
| Oceania | Endometriosis |  | 810(464 to 1,393) |  | 22.93(21.38 to 24.58) |  | 6(2 to 15) |  | 0.17(0.06 to 0.39) |
|  | Unexplained infertility |  | 16,464(8,978 to 26,802) |  | 464.41(457.33 to 471.58) |  | 123(42 to 275) |  | 3.48(2.89 to 4.16) |
|  | Polycystic ovarian syndrome |  | 5,333(2,855 to 9,074) |  | 149.62(145.62 to 153.71) |  | 40(14 to 91) |  | 1.11(0.79 to 1.53) |
| Central Asia | Endometriosis |  | 3,671(1,699 to 7,121) |  | 15.28(14.79 to 15.78) |  | 27(9 to 68) |  | 0.11(0.08 to 0.17) |
|  | Unexplained infertility |  | 145,639(58,311 to 300,447) |  | 594.20(591.13 to 597.27) |  | 1,085(312 to 2,493) |  | 4.43(4.17 to 4.70) |
|  | Polycystic ovarian syndrome |  | 11,282(4,900 to 21,222) |  | 48.35(47.45 to 49.25) |  | 84(26 to 204) |  | 0.36(0.29 to 0.45) |
| Central Europe | Endometriosis |  | 2,553(1,123 to 5,162) |  | 11.35(10.90 to 11.81) |  | 19(6 to 50) |  | 0.09(0.05 to 0.14) |
|  | Unexplained infertility |  | 174,546(73,307 to 358,044) |  | 755.53(751.92 to 759.16) |  | 1,307(410 to 3,375) |  | 5.66(5.35 to 5.98) |
|  | Polycystic ovarian syndrome |  | 4,374(1,757 to 8,474) |  | 20.00(19.39 to 20.62) |  | 33(10 to 80) |  | 0.15(0.10 to 0.21) |
| Eastern Europe | Endometriosis |  | 11,967(5,609 to 22,765) |  | 28.31(27.78 to 28.85) |  | 90(29 to 222) |  | 0.21(0.17 to 0.26) |
|  | Unexplained infertility |  | 743,057(328,374 to 1,452,450) |  | 1725.74(1721.65 to 1729.84) |  | 5,557(1,653 to 13,459) |  | 12.90(12.55 to 13.26) |
|  | Polycystic ovarian syndrome |  | 13,856(6,637 to 26,462) |  | 33.76(33.17 to 34.35) |  | 103(35 to 240) |  | 0.25(0.20 to 0.31) |
| High-income Asia Pacific | Endometriosis |  | 3,855(1,470 to 8,166) |  | 11.25(10.90 to 11.62) |  | 29(9 to 73) |  | 0.08(0.06 to 0.12) |
|  | Unexplained infertility |  | 41,558(8,184 to 121,969) |  | 104.40(103.38 to 105.44) |  | 315(47 to 1,089) |  | 0.79(0.70 to 0.89) |
|  | Polycystic ovarian syndrome |  | 110,671(45,785 to 216,124) |  | 326.44(324.49 to 328.40) |  | 823(254 to 1,875) |  | 2.43(2.26 to 2.61) |
| Australasia | Endometriosis |  | 861(312 to 1,816) |  | 12.31(11.50 to 13.17) |  | 6(2 to 17) |  | 0.09(0.04 to 0.21) |
|  | Unexplained infertility |  | 4,928(1,264 to 21,033) |  | 67.92(66.02 to 69.86) |  | 38(7 to 180) |  | 0.52(0.37 to 0.72) |
|  | Polycystic ovarian syndrome |  | 25,157(11,112 to 47,595) |  | 358.53(354.09 to 363.03) |  | 188(59 to 440) |  | 2.67(2.30 to 3.09) |
| Western Europe | Endometriosis |  | 16,740(8,198 to 29,617) |  | 19.34(19.04 to 19.64) |  | 126(45 to 310) |  | 0.15(0.12 to 0.17) |
|  | Unexplained infertility |  | 658,550(186,946 to 1,554,711) |  | 710.18(708.45 to 711.92) |  | 4,950(1,052 to 13,975) |  | 5.34(5.19 to 5.49) |
|  | Polycystic ovarian syndrome |  | 446,756(218,547 to 759,741) |  | 513.94(512.41 to 515.46) |  | 3,336(1,244 to 7,576) |  | 3.84(3.71 to 3.97) |
| Southern Latin America | Endometriosis |  | 2,363(1,093 to 4,330) |  | 13.54(13.00 to 14.10) |  | 18(6 to 46) |  | 0.10(0.06 to 0.16) |
|  | Unexplained infertility |  | 88,319(17,607 to 229,159) |  | 501.10(497.79 to 504.42) |  | 661(97 to 2,047) |  | 3.75(3.47 to 4.05) |
|  | Polycystic ovarian syndrome |  | 39,968(18,287 to 75,452) |  | 231.44(229.18 to 233.73) |  | 297(103 to 703) |  | 1.72(1.53 to 1.93) |
| High-income North America | Endometriosis |  | 10,072(4,806 to 17,851) |  | 12.04(11.80 to 12.28) |  | 76(24 to 185) |  | 0.09(0.07 to 0.11) |
|  | Unexplained infertility |  | 474,391(71,586 to 1,295,452) |  | 566.86(565.24 to 568.48) |  | 3,555(397 to 10,802) |  | 4.25(4.11 to 4.39) |
|  | Polycystic ovarian syndrome |  | 404,222(196,053 to 717,870) |  | 491.59(490.07 to 493.12) |  | 3,009(1,134 to 6,703) |  | 3.66(3.53 to 3.79) |
| Caribbean | Endometriosis |  | 1,569(824 to 2,881) |  | 13.04(12.41 to 13.71) |  | 12(4 to 29) |  | 0.10(0.05 to 0.17) |
|  | Unexplained infertility |  | 121,413(58,051 to 229,460) |  | 1003.96(998.32 to 1009.63) |  | 909(311 to 2,200) |  | 7.52(7.04 to 8.02) |
|  | Polycystic ovarian syndrome |  | 19,530(9,918 to 36,899) |  | 164.18(161.88 to 166.50) |  | 145(51 to 342) |  | 1.22(1.03 to 1.44) |
| Andean Latin America | Endometriosis |  | 1,349(504 to 3,007) |  | 7.49(7.10 to 7.90) |  | 10(3 to 27) |  | 0.06(0.03 to 0.10) |
|  | Unexplained infertility |  | 20,366(3,357 to 60,181) |  | 113.76(112.20 to 115.33) |  | 153(19 to 551) |  | 0.86(0.73 to 1.00) |
|  | Polycystic ovarian syndrome |  | 33,934(13,961 to 64,758) |  | 189.41(187.40 to 191.44) |  | 253(82 to 597) |  | 1.41(1.24 to 1.60) |
| Central Latin America | Endometriosis |  | 6,757(3,279 to 13,248) |  | 9.87(9.64 to 10.11) |  | 51(17 to 129) |  | 0.07(0.06 to 0.10) |
|  | Unexplained infertility |  | 309,257(115,168 to 677,335) |  | 451.58(449.99 to 453.18) |  | 2,319(562 to 6,529) |  | 3.39(3.25 to 3.53) |
|  | Polycystic ovarian syndrome |  | 155,551(72,668 to 280,346) |  | 228.17(227.04 to 229.31) |  | 1,157(397 to 2,665) |  | 1.70(1.60 to 1.80) |
| Tropical Latin America | Endometriosis |  | 10,784(5,130 to 19,861) |  | 18.14(17.79 to 18.48) |  | 81(26 to 201) |  | 0.14(0.11 to 0.17) |
|  | Unexplained infertility |  | 492,926(184,556 to 1,011,260) |  | 796.11(793.88 to 798.34) |  | 3,682(963 to 9,730) |  | 5.95(5.75 to 6.14) |
|  | Polycystic ovarian syndrome |  | 43,751(20,374 to 83,252) |  | 74.47(73.77 to 75.18) |  | 325(114 to 769) |  | 0.55(0.50 to 0.62) |
| North Africa and Middle East | Endometriosis |  | 41,569(21,700 to 75,549) |  | 26.18(25.93 to 26.43) |  | 311(110 to 731) |  | 0.20(0.17 to 0.22) |
|  | Unexplained infertility |  | 1,801,572(737,782 to 3,716,380) |  | 1125.07(1123.42 to 1126.71) |  | 13,463(3,913 to 35,698) |  | 8.41(8.27 to 8.55) |
|  | Polycystic ovarian syndrome |  | 462,718(224,736 to 815,941) |  | 292.22(291.38 to 293.07) |  | 3,442(1,214 to 7,728) |  | 2.17(2.10 to 2.25) |
| South Asia | Endometriosis |  | 100,617(50,646 to 189,791) |  | 20.04(19.98 to 20.10) |  | 753(264 to 1,886) |  | 0.15(0.14 to 0.16) |
|  | Unexplained infertility |  | 9,546,478(4,011,619 to 19,250,128) |  | 1889.80(1889.20 to 1890.40) |  | 71,026(22,323 to 172,086) |  | 14.06(14.01 to 14.11) |
|  | Polycystic ovarian syndrome |  | 658,771(328,936 to 1,218,271) |  | 131.39(131.23 to 131.55) |  | 4,881(1,786 to 11,310) |  | 0.97(0.96 to 0.99) |
| Central Sub-Saharan Africa | Endometriosis |  | 5,665(2,353 to 11,224) |  | 16.32(15.89 to 16.75) |  | 42(13 to 112) |  | 0.12(0.09 to 0.17) |
|  | Unexplained infertility |  | 291,701(118,258 to 591,228) |  | 889.40(886.13 to 892.68) |  | 2,174(596 to 5,478) |  | 6.63(6.35 to 6.92) |
|  | Polycystic ovarian syndrome |  | 23,087(9,308 to 44,605) |  | 63.35(62.53 to 64.19) |  | 171(51 to 443) |  | 0.47(0.40 to 0.55) |
| Eastern Sub-Saharan Africa | Endometriosis |  | 14,770(7,333 to 27,294) |  | 12.91(12.70 to 13.13) |  | 111(39 to 274) |  | 0.10(0.08 to 0.12) |
|  | Unexplained infertility |  | 748,844(346,392 to 1,453,262) |  | 675.85(674.30 to 677.41) |  | 5,580(1,833 to 13,577) |  | 5.04(4.90 to 5.17) |
|  | Polycystic ovarian syndrome |  | 69,039(32,832 to 129,873) |  | 57.36(56.92 to 57.79) |  | 511(172 to 1,230) |  | 0.42(0.39 to 0.46) |
| Southern Sub-Saharan Africa | Endometriosis |  | 3,650(1,563 to 7,141) |  | 16.54(16.01 to 17.09) |  | 27(9 to 69) |  | 0.12(0.08 to 0.18) |
|  | Unexplained infertility |  | 139,416(48,610 to 316,832) |  | 621.62(618.36 to 624.90) |  | 1,039(275 to 2,835) |  | 4.63(4.36 to 4.93) |
|  | Polycystic ovarian syndrome |  | 24,321(10,784 to 47,577) |  | 110.84(109.45 to 112.25) |  | 180(59 to 447) |  | 0.82(0.71 to 0.95) |
| Western Sub-Saharan Africa | Endometriosis |  | 20,983(8,989 to 41,275) |  | 16.14(15.92 to 16.37) |  | 157(52 to 399) |  | 0.12(0.10 to 0.14) |
|  | Unexplained infertility |  | 783,912(304,952 to 1,646,947) |  | 622.97(621.57 to 624.38) |  | 5,841(1,619 to 15,136) |  | 4.64(4.52 to 4.77) |
|  | Polycystic ovarian syndrome |  | 88,045(37,703 to 170,042) |  | 64.33(63.90 to 64.77) |  | 653(201 to 1,631) |  | 0.48(0.44 to 0.52) |
| **Countries and territories** |  |  |  |  |  |  |  |  |  |
| China | Endometriosis |  | 16,401(7,010 to 33,398) |  | 5.67(5.58 to 5.76) |  | 123(39 to 318) |  | 0.04(0.04 to 0.05) |
|  | Unexplained infertility |  | 2,171,079(1,011,727 to 4,025,808) |  | 700.81(699.85 to 701.77) |  | 16,239(5,001 to 36,701) |  | 5.24(5.16 to 5.33) |
|  | Polycystic ovarian syndrome |  | 256,404(111,417 to 507,761) |  | 96.41(96.02 to 96.79) |  | 1,904(598 to 4,681) |  | 0.72(0.68 to 0.75) |
| Democratic People's Republic of Korea | Endometriosis |  | 634(256 to 1,355) |  | 9.75(9.00 to 10.54) |  | 5(1 to 13) |  | 0.07(0.02 to 0.18) |
|  | Unexplained infertility |  | 33,763(14,912 to 64,387) |  | 514.25(508.77 to 519.78) |  | 253(75 to 625) |  | 3.85(3.39 to 4.36) |
|  | Polycystic ovarian syndrome |  | 4,016(1,563 to 7,927) |  | 64.68(62.68 to 66.72) |  | 30(9 to 74) |  | 0.48(0.32 to 0.69) |
| Cambodia | Endometriosis |  | 642(251 to 1,365) |  | 13.79(12.74 to 14.90) |  | 5(1 to 12) |  | 0.10(0.03 to 0.26) |
|  | Unexplained infertility |  | 26,576(11,061 to 52,606) |  | 565.20(558.42 to 572.06) |  | 198(51 to 496) |  | 4.22(3.65 to 4.86) |
|  | Polycystic ovarian syndrome |  | 7,408(3,148 to 13,776) |  | 160.36(156.72 to 164.07) |  | 55(17 to 139) |  | 1.19(0.90 to 1.56) |
| Indonesia | Endometriosis |  | 14,246(6,724 to 26,817) |  | 19.06(18.75 to 19.37) |  | 107(36 to 253) |  | 0.14(0.12 to 0.17) |
|  | Unexplained infertility |  | 1,104,211(478,957 to 2,213,168) |  | 1462.35(1459.62 to 1465.08) |  | 8,254(2,542 to 20,858) |  | 10.93(10.70 to 11.17) |
|  | Polycystic ovarian syndrome |  | 232,480(115,660 to 426,218) |  | 316.39(315.10 to 317.68) |  | 1,725(614 to 3,870) |  | 2.35(2.24 to 2.46) |
| Lao People's Democratic Republic | Endometriosis |  | 400(162 to 802) |  | 19.31(17.46 to 21.32) |  | 3(1 to 8) |  | 0.15(0.03 to 0.46) |
|  | Unexplained infertility |  | 14,419(5,412 to 29,500) |  | 708.64(697.08 to 720.36) |  | 108(28 to 272) |  | 5.29(4.33 to 6.40) |
|  | Polycystic ovarian syndrome |  | 5,303(2,261 to 9,991) |  | 255.89(249.03 to 262.91) |  | 39(14 to 95) |  | 1.90(1.35 to 2.61) |
| Malaysia | Endometriosis |  | 1,066(420 to 2,350) |  | 12.16(11.44 to 12.92) |  | 8(2 to 21) |  | 0.09(0.04 to 0.18) |
|  | Unexplained infertility |  | 43,679(16,249 to 88,583) |  | 497.73(493.07 to 502.43) |  | 326(82 to 840) |  | 3.72(3.32 to 4.14) |
|  | Polycystic ovarian syndrome |  | 24,820(10,494 to 48,266) |  | 286.10(282.54 to 289.68) |  | 184(60 to 437) |  | 2.12(1.83 to 2.45) |
| Maldives | Endometriosis |  | 26(15 to 43) |  | 22.87(14.87 to 34.07) |  | 0(0 to 0) |  | 0.17(0.00 to 4.69) |
|  | Unexplained infertility |  | 2,479(1,722 to 3,441) |  | 2009.04(1929.82 to 2091.05) |  | 19(8 to 40) |  | 15.08(8.96 to 24.36) |
|  | Polycystic ovarian syndrome |  | 447(269 to 741) |  | 399.24(362.51 to 438.98) |  | 3(1 to 8) |  | 2.98(0.67 to 9.03) |
| Myanmar | Endometriosis |  | 2,805(1,589 to 4,702) |  | 18.48(17.81 to 19.18) |  | 21(7 to 55) |  | 0.14(0.09 to 0.22) |
|  | Unexplained infertility |  | 67,051(40,764 to 103,096) |  | 453.96(450.53 to 457.41) |  | 505(179 to 1,148) |  | 3.42(3.13 to 3.73) |
|  | Polycystic ovarian syndrome |  | 41,558(24,646 to 65,898) |  | 273.04(270.42 to 275.68) |  | 310(118 to 697) |  | 2.03(1.81 to 2.28) |
| Philippines | Endometriosis |  | 7,701(2,889 to 16,231) |  | 25.95(25.37 to 26.54) |  | 57(16 to 151) |  | 0.19(0.15 to 0.25) |
|  | Unexplained infertility |  | 759,324(172,147 to 2,006,427) |  | 2581.30(2575.48 to 2587.13) |  | 5,617(1,019 to 17,689) |  | 19.10(18.60 to 19.61) |
|  | Polycystic ovarian syndrome |  | 101,695(37,323 to 202,846) |  | 339.86(337.77 to 341.97) |  | 754(228 to 1,816) |  | 2.52(2.34 to 2.71) |
| Sri Lanka | Endometriosis |  | 822(361 to 1,624) |  | 15.09(14.08 to 16.16) |  | 6(2 to 15) |  | 0.11(0.04 to 0.25) |
|  | Unexplained infertility |  | 30,480(10,709 to 65,913) |  | 545.66(539.53 to 551.84) |  | 228(54 to 576) |  | 4.08(3.56 to 4.65) |
|  | Polycystic ovarian syndrome |  | 17,036(8,124 to 31,328) |  | 314.15(309.43 to 318.91) |  | 126(44 to 288) |  | 2.32(1.93 to 2.77) |
| Thailand | Endometriosis |  | 2,707(1,323 to 5,160) |  | 17.67(17.00 to 18.37) |  | 20(6 to 51) |  | 0.13(0.08 to 0.21) |
|  | Unexplained infertility |  | 120,772(44,632 to 251,367) |  | 760.54(756.21 to 764.90) |  | 905(216 to 2,333) |  | 5.70(5.33 to 6.09) |
|  | Polycystic ovarian syndrome |  | 63,117(30,191 to 115,760) |  | 423.73(420.38 to 427.11) |  | 471(171 to 1,029) |  | 3.16(2.88 to 3.47) |
| Timor-Leste | Endometriosis |  | 100(42 to 192) |  | 27.52(22.26 to 33.80) |  | 1(0 to 2) |  | 0.21(0.00 to 1.85) |
|  | Unexplained infertility |  | 2,104(710 to 4,551) |  | 636.55(608.92 to 665.21) |  | 16(4 to 42) |  | 4.75(2.65 to 8.00) |
|  | Polycystic ovarian syndrome |  | 904(410 to 1,580) |  | 237.19(221.54 to 253.79) |  | 7(2 to 15) |  | 1.76(0.67 to 4.03) |
| Viet Nam | Endometriosis |  | 3,500(1,478 to 7,136) |  | 14.09(13.62 to 14.57) |  | 26(8 to 67) |  | 0.11(0.07 to 0.16) |
|  | Unexplained infertility |  | 65,009(17,577 to 145,969) |  | 241.48(239.61 to 243.36) |  | 486(97 to 1,302) |  | 1.81(1.65 to 1.98) |
|  | Polycystic ovarian syndrome |  | 48,643(21,408 to 93,562) |  | 200.36(198.55 to 202.17) |  | 363(125 to 856) |  | 1.50(1.34 to 1.66) |
| Fiji | Endometriosis |  | 53(25 to 104) |  | 23.39(17.54 to 30.59) |  | 0(0 to 1) |  | 0.17(0.00 to 2.02) |
|  | Unexplained infertility |  | 3,439(1,525 to 6,568) |  | 1500.23(1450.49 to 1551.26) |  | 26(8 to 62) |  | 11.21(7.30 to 16.49) |
|  | Polycystic ovarian syndrome |  | 630(299 to 1,153) |  | 276.66(255.48 to 299.14) |  | 5(2 to 11) |  | 2.05(0.63 to 4.95) |
| Kiribati | Endometriosis |  | 8(4 to 17) |  | 24.72(10.74 to 50.09) |  | 0(0 to 0) |  | 0.18(0.00 to 14.73) |
|  | Unexplained infertility |  | 268(106 to 544) |  | 821.81(726.28 to 927.37) |  | 2(1 to 5) |  | 6.14(0.74 to 24.31) |
|  | Polycystic ovarian syndrome |  | 65(27 to 122) |  | 196.30(151.44 to 251.42) |  | 0(0 to 1) |  | 1.45(0.00 to 16.87) |
| Marshall Islands | Endometriosis |  | 3(1 to 6) |  | 20.74(4.33 to 61.72) |  | 0(0 to 0) |  | 0.16(0.00 to 27.90) |
|  | Unexplained infertility |  | 121(49 to 242) |  | 830.95(689.51 to 993.60) |  | 1(0 to 2) |  | 6.21(0.11 to 38.88) |
|  | Polycystic ovarian syndrome |  | 29(13 to 54) |  | 194.56(130.19 to 280.65) |  | 0(0 to 1) |  | 1.44(0.00 to 30.34) |
| Micronesia (Federated States of) | Endometriosis |  | 5(2 to 11) |  | 20.95(7.19 to 48.51) |  | 0(0 to 0) |  | 0.15(0.00 to 16.65) |
|  | Unexplained infertility |  | 205(81 to 422) |  | 810.86(703.11 to 930.96) |  | 2(0 to 4) |  | 6.06(0.44 to 26.89) |
|  | Polycystic ovarian syndrome |  | 57(25 to 109) |  | 211.33(159.61 to 275.34) |  | 0(0 to 1) |  | 1.56(0.00 to 19.18) |
| Papua New Guinea | Endometriosis |  | 605(341 to 1,033) |  | 22.66(20.88 to 24.55) |  | 5(2 to 11) |  | 0.17(0.05 to 0.44) |
|  | Unexplained infertility |  | 8,579(4,754 to 13,273) |  | 315.65(308.99 to 322.43) |  | 64(22 to 148) |  | 2.37(1.83 to 3.04) |
|  | Polycystic ovarian syndrome |  | 3,450(1,912 to 5,788) |  | 128.20(123.95 to 132.57) |  | 26(9 to 58) |  | 0.95(0.62 to 1.42) |
| Samoa | Endometriosis |  | 10(4 to 20) |  | 20.24(9.62 to 38.26) |  | 0(0 to 0) |  | 0.15(0.00 to 9.60) |
|  | Unexplained infertility |  | 372(141 to 771) |  | 786.87(708.54 to 871.93) |  | 3(1 to 7) |  | 5.87(1.09 to 18.83) |
|  | Polycystic ovarian syndrome |  | 115(49 to 215) |  | 227.85(187.81 to 274.54) |  | 1(0 to 2) |  | 1.69(0.02 to 12.19) |
| Solomon Islands | Endometriosis |  | 50(22 to 100) |  | 28.78(21.31 to 38.16) |  | 0(0 to 1) |  | 0.22(0.00 to 2.94) |
|  | Unexplained infertility |  | 1,390(534 to 2,819) |  | 823.63(780.77 to 868.34) |  | 10(3 to 27) |  | 6.14(2.98 to 11.36) |
|  | Polycystic ovarian syndrome |  | 323(136 to 595) |  | 182.85(163.38 to 204.14) |  | 2(1 to 6) |  | 1.35(0.21 to 4.75) |
| Tonga | Endometriosis |  | 6(3 to 12) |  | 22.91(8.12 to 51.58) |  | 0(0 to 0) |  | 0.17(0.00 to 16.54) |
|  | Unexplained infertility |  | 186(68 to 393) |  | 762.83(656.76 to 881.44) |  | 1(0 to 4) |  | 5.68(0.34 to 26.35) |
|  | Polycystic ovarian syndrome |  | 66(28 to 128) |  | 258.34(199.60 to 329.54) |  | 0(0 to 1) |  | 1.91(0.00 to 19.74) |
| Vanuatu | Endometriosis |  | 18(8 to 36) |  | 22.54(13.36 to 36.09) |  | 0(0 to 0) |  | 0.17(0.00 to 6.04) |
|  | Unexplained infertility |  | 644(255 to 1,302) |  | 819.98(757.50 to 886.53) |  | 5(1 to 12) |  | 6.13(1.92 to 15.14) |
|  | Polycystic ovarian syndrome |  | 163(72 to 307) |  | 198.49(169.06 to 231.96) |  | 1(0 to 3) |  | 1.47(0.06 to 8.18) |
| Armenia | Endometriosis |  | 55(29 to 100) |  | 8.03(6.00 to 10.59) |  | 0(0 to 1) |  | 0.06(0.00 to 0.87) |
|  | Unexplained infertility |  | 1,097(512 to 1,973) |  | 158.81(149.29 to 168.84) |  | 8(3 to 19) |  | 1.19(0.51 to 2.48) |
|  | Polycystic ovarian syndrome |  | 218(105 to 420) |  | 32.72(28.38 to 37.59) |  | 2(1 to 4) |  | 0.25(0.02 to 1.17) |
| Azerbaijan | Endometriosis |  | 426(185 to 847) |  | 15.85(14.36 to 17.47) |  | 3(1 to 8) |  | 0.12(0.03 to 0.37) |
|  | Unexplained infertility |  | 27,601(10,721 to 57,305) |  | 990.31(978.48 to 1002.27) |  | 206(61 to 487) |  | 7.38(6.39 to 8.50) |
|  | Polycystic ovarian syndrome |  | 1,488(658 to 2,907) |  | 58.48(55.49 to 61.61) |  | 11(4 to 28) |  | 0.43(0.21 to 0.80) |
| Georgia | Endometriosis |  | 101(43 to 206) |  | 14.41(11.70 to 17.61) |  | 1(0 to 2) |  | 0.11(0.00 to 0.87) |
|  | Unexplained infertility |  | 4,141(1,413 to 8,974) |  | 572.20(554.64 to 590.23) |  | 31(7 to 79) |  | 4.28(2.89 to 6.17) |
|  | Polycystic ovarian syndrome |  | 463(181 to 893) |  | 67.68(61.53 to 74.32) |  | 3(1 to 9) |  | 0.50(0.11 to 1.48) |
| Kazakhstan | Endometriosis |  | 738(330 to 1,458) |  | 16.50(15.32 to 17.76) |  | 6(2 to 14) |  | 0.12(0.04 to 0.29) |
|  | Unexplained infertility |  | 31,714(11,749 to 67,352) |  | 692.81(685.10 to 700.59) |  | 236(64 to 554) |  | 5.16(4.51 to 5.87) |
|  | Polycystic ovarian syndrome |  | 2,388(991 to 4,581) |  | 54.72(52.52 to 57.00) |  | 18(5 to 43) |  | 0.41(0.24 to 0.65) |
| Kyrgyzstan | Endometriosis |  | 266(111 to 568) |  | 14.98(13.23 to 16.91) |  | 2(1 to 5) |  | 0.11(0.01 to 0.43) |
|  | Unexplained infertility |  | 13,750(5,447 to 28,861) |  | 767.10(754.29 to 780.08) |  | 102(30 to 249) |  | 5.72(4.66 to 6.95) |
|  | Polycystic ovarian syndrome |  | 644(261 to 1,303) |  | 37.12(34.30 to 40.12) |  | 5(1 to 11) |  | 0.27(0.09 to 0.67) |
| Mongolia | Endometriosis |  | 138(57 to 280) |  | 16.94(14.21 to 20.08) |  | 1(0 to 3) |  | 0.13(0.00 to 0.77) |
|  | Unexplained infertility |  | 5,352(1,949 to 11,058) |  | 643.36(626.11 to 660.99) |  | 40(11 to 98) |  | 4.79(3.41 to 6.58) |
|  | Polycystic ovarian syndrome |  | 364(149 to 719) |  | 46.09(41.44 to 51.16) |  | 3(1 to 7) |  | 0.34(0.06 to 1.11) |
| Tajikistan | Endometriosis |  | 530(295 to 895) |  | 19.72(18.07 to 21.50) |  | 4(1 to 10) |  | 0.15(0.04 to 0.41) |
|  | Unexplained infertility |  | 10,869(6,157 to 17,514) |  | 392.54(385.18 to 400.01) |  | 82(28 to 188) |  | 2.95(2.35 to 3.68) |
|  | Polycystic ovarian syndrome |  | 1,131(572 to 2,072) |  | 42.83(40.36 to 45.43) |  | 8(3 to 20) |  | 0.32(0.14 to 0.64) |
| Turkmenistan | Endometriosis |  | 203(83 to 419) |  | 15.92(13.80 to 18.27) |  | 2(0 to 4) |  | 0.12(0.01 to 0.52) |
|  | Unexplained infertility |  | 8,201(3,072 to 17,984) |  | 641.05(627.24 to 655.08) |  | 61(17 to 147) |  | 4.78(3.66 to 6.15) |
|  | Polycystic ovarian syndrome |  | 613(253 to 1,210) |  | 48.31(44.56 to 52.30) |  | 4(1 to 12) |  | 0.35(0.11 to 0.87) |
| Uzbekistan | Endometriosis |  | 1,213(508 to 2,567) |  | 13.58(12.82 to 14.37) |  | 9(3 to 23) |  | 0.10(0.05 to 0.20) |
|  | Unexplained infertility |  | 42,914(15,458 to 96,434) |  | 472.24(467.77 to 476.75) |  | 319(80 to 831) |  | 3.51(3.14 to 3.92) |
|  | Polycystic ovarian syndrome |  | 3,974(1,613 to 7,803) |  | 45.95(44.53 to 47.42) |  | 30(9 to 74) |  | 0.34(0.23 to 0.49) |
| Albania | Endometriosis |  | 108(63 to 179) |  | 17.35(14.22 to 21.01) |  | 1(0 to 2) |  | 0.13(0.00 to 0.97) |
|  | Unexplained infertility |  | 2,947(1,720 to 4,703) |  | 473.98(457.01 to 491.46) |  | 22(8 to 46) |  | 3.56(2.23 to 5.44) |
|  | Polycystic ovarian syndrome |  | 135(69 to 240) |  | 22.26(18.64 to 26.42) |  | 1(0 to 2) |  | 0.17(0.00 to 1.04) |
| Bosnia and Herzegovina | Endometriosis |  | 72(30 to 151) |  | 10.75(8.38 to 13.65) |  | 1(0 to 1) |  | 0.08(0.00 to 0.86) |
|  | Unexplained infertility |  | 3,592(1,358 to 7,473) |  | 530.68(513.30 to 548.55) |  | 27(7 to 69) |  | 3.99(2.61 to 5.89) |
|  | Polycystic ovarian syndrome |  | 103(37 to 212) |  | 15.87(12.90 to 19.36) |  | 1(0 to 2) |  | 0.12(0.00 to 0.93) |
| Bulgaria | Endometriosis |  | 134(57 to 272) |  | 11.35(9.45 to 13.54) |  | 1(0 to 3) |  | 0.09(0.00 to 0.55) |
|  | Unexplained infertility |  | 6,421(2,525 to 12,487) |  | 524.04(510.92 to 537.45) |  | 48(14 to 121) |  | 3.93(2.87 to 5.28) |
|  | Polycystic ovarian syndrome |  | 208(78 to 429) |  | 17.87(15.44 to 20.60) |  | 2(0 to 4) |  | 0.13(0.01 to 0.63) |
| Croatia | Endometriosis |  | 76(30 to 158) |  | 9.41(7.39 to 11.86) |  | 1(0 to 2) |  | 0.07(0.00 to 0.73) |
|  | Unexplained infertility |  | 4,273(1,566 to 8,828) |  | 513.38(497.95 to 529.21) |  | 32(9 to 82) |  | 3.85(2.62 to 5.51) |
|  | Polycystic ovarian syndrome |  | 132(48 to 276) |  | 16.79(14.00 to 20.01) |  | 1(0 to 3) |  | 0.13(0.00 to 0.82) |
| Czechia | Endometriosis |  | 212(89 to 444) |  | 11.09(9.59 to 12.78) |  | 2(0 to 4) |  | 0.08(0.01 to 0.39) |
|  | Unexplained infertility |  | 13,928(5,738 to 27,539) |  | 712.62(700.45 to 724.96) |  | 104(31 to 262) |  | 5.34(4.34 to 6.53) |
|  | Polycystic ovarian syndrome |  | 300(109 to 633) |  | 16.01(14.18 to 18.03) |  | 2(1 to 6) |  | 0.12(0.02 to 0.46) |
| Hungary | Endometriosis |  | 198(84 to 408) |  | 10.77(9.28 to 12.43) |  | 2(0 to 4) |  | 0.08(0.01 to 0.40) |
|  | Unexplained infertility |  | 9,699(3,669 to 19,620) |  | 520.03(509.54 to 530.70) |  | 73(21 to 187) |  | 3.90(3.04 to 4.94) |
|  | Polycystic ovarian syndrome |  | 310(109 to 630) |  | 17.17(15.26 to 19.27) |  | 2(1 to 6) |  | 0.13(0.02 to 0.47) |
| North Macedonia | Endometriosis |  | 54(21 to 114) |  | 11.08(8.28 to 14.62) |  | 0(0 to 1) |  | 0.08(0.00 to 1.21) |
|  | Unexplained infertility |  | 2,665(1,042 to 5,536) |  | 525.90(505.87 to 546.61) |  | 20(6 to 51) |  | 3.95(2.40 to 6.28) |
|  | Polycystic ovarian syndrome |  | 75(27 to 156) |  | 16.08(12.58 to 20.34) |  | 1(0 to 1) |  | 0.12(0.00 to 1.28) |
| Montenegro | Endometriosis |  | 16(6 to 33) |  | 11.77(6.65 to 19.50) |  | 0(0 to 0) |  | 0.09(0.00 to 3.47) |
|  | Unexplained infertility |  | 700(273 to 1,453) |  | 520.07(482.02 to 560.48) |  | 5(1 to 13) |  | 3.91(1.30 to 9.27) |
|  | Polycystic ovarian syndrome |  | 23(8 to 48) |  | 17.92(11.36 to 27.06) |  | 0(0 to 0) |  | 0.13(0.00 to 3.55) |
| Poland | Endometriosis |  | 913(407 to 1,860) |  | 11.74(10.96 to 12.56) |  | 7(2 to 18) |  | 0.09(0.03 to 0.20) |
|  | Unexplained infertility |  | 92,630(39,154 to 195,045) |  | 1154.80(1147.16 to 1162.48) |  | 693(213 to 1,777) |  | 8.64(7.99 to 9.33) |
|  | Polycystic ovarian syndrome |  | 1,908(809 to 3,619) |  | 26.06(24.85 to 27.31) |  | 14(5 to 34) |  | 0.19(0.10 to 0.34) |
| Romania | Endometriosis |  | 395(165 to 822) |  | 11.44(10.32 to 12.66) |  | 3(1 to 8) |  | 0.09(0.02 to 0.26) |
|  | Unexplained infertility |  | 18,313(7,058 to 36,114) |  | 523.81(516.10 to 531.60) |  | 137(37 to 359) |  | 3.91(3.27 to 4.64) |
|  | Polycystic ovarian syndrome |  | 595(215 to 1,225) |  | 17.22(15.83 to 18.70) |  | 4(1 to 12) |  | 0.13(0.04 to 0.33) |
| Serbia | Endometriosis |  | 189(76 to 400) |  | 10.22(8.80 to 11.82) |  | 1(0 to 4) |  | 0.08(0.00 to 0.37) |
|  | Unexplained infertility |  | 10,020(3,744 to 20,672) |  | 530.85(520.41 to 541.46) |  | 75(20 to 190) |  | 3.97(3.12 to 5.01) |
|  | Polycystic ovarian syndrome |  | 298(107 to 610) |  | 16.35(14.52 to 18.35) |  | 2(1 to 6) |  | 0.12(0.02 to 0.44) |
| Slovakia | Endometriosis |  | 120(51 to 254) |  | 10.98(9.05 to 13.24) |  | 1(0 to 2) |  | 0.08(0.00 to 0.61) |
|  | Unexplained infertility |  | 6,096(2,418 to 13,018) |  | 533.64(519.99 to 547.60) |  | 46(13 to 120) |  | 4.00(2.90 to 5.44) |
|  | Polycystic ovarian syndrome |  | 184(65 to 381) |  | 17.35(14.85 to 20.19) |  | 1(0 to 3) |  | 0.13(0.01 to 0.69) |
| Slovenia | Endometriosis |  | 31(11 to 71) |  | 8.76(5.88 to 12.65) |  | 0(0 to 1) |  | 0.07(0.00 to 1.49) |
|  | Unexplained infertility |  | 722(258 to 1,519) |  | 195.19(180.88 to 210.42) |  | 5(1 to 15) |  | 1.46(0.48 to 3.55) |
|  | Polycystic ovarian syndrome |  | 38(12 to 86) |  | 10.90(7.63 to 15.19) |  | 0(0 to 1) |  | 0.08(0.00 to 1.52) |
| Belarus | Endometriosis |  | 493(228 to 929) |  | 26.44(24.06 to 29.01) |  | 4(1 to 9) |  | 0.20(0.05 to 0.59) |
|  | Unexplained infertility |  | 22,734(9,564 to 45,169) |  | 1173.11(1157.30 to 1189.10) |  | 171(49 to 406) |  | 8.80(7.48 to 10.31) |
|  | Polycystic ovarian syndrome |  | 601(272 to 1,140) |  | 33.95(31.17 to 36.93) |  | 4(1 to 11) |  | 0.25(0.07 to 0.67) |
| Estonia | Endometriosis |  | 61(29 to 119) |  | 24.69(18.74 to 32.07) |  | 0(0 to 1) |  | 0.19(0.00 to 2.30) |
|  | Unexplained infertility |  | 2,985(1,224 to 5,867) |  | 1166.62(1124.03 to 1210.54) |  | 22(7 to 55) |  | 8.74(5.42 to 13.52) |
|  | Polycystic ovarian syndrome |  | 90(43 to 169) |  | 37.95(30.30 to 47.05) |  | 1(0 to 2) |  | 0.28(0.00 to 2.47) |
| Latvia | Endometriosis |  | 74(34 to 145) |  | 21.83(16.99 to 27.73) |  | 1(0 to 1) |  | 0.17(0.00 to 1.76) |
|  | Unexplained infertility |  | 3,781(1,562 to 7,601) |  | 1070.22(1035.29 to 1106.13) |  | 28(8 to 71) |  | 8.01(5.26 to 11.84) |
|  | Polycystic ovarian syndrome |  | 105(47 to 202) |  | 31.88(25.86 to 38.96) |  | 1(0 to 2) |  | 0.24(0.00 to 1.89) |
| Lithuania | Endometriosis |  | 123(54 to 252) |  | 23.11(19.15 to 27.74) |  | 1(0 to 2) |  | 0.17(0.00 to 1.27) |
|  | Unexplained infertility |  | 5,235(2,232 to 10,217) |  | 958.77(932.73 to 985.44) |  | 39(11 to 96) |  | 7.17(5.08 to 9.94) |
|  | Polycystic ovarian syndrome |  | 140(61 to 282) |  | 28.20(23.63 to 33.48) |  | 1(0 to 3) |  | 0.21(0.01 to 1.34) |
| Republic of Moldova | Endometriosis |  | 202(98 to 385) |  | 25.62(22.06 to 29.66) |  | 2(0 to 4) |  | 0.19(0.01 to 1.03) |
|  | Unexplained infertility |  | 11,271(4,690 to 21,488) |  | 1367.78(1341.62 to 1394.38) |  | 84(25 to 195) |  | 10.20(8.06 to 12.83) |
|  | Polycystic ovarian syndrome |  | 248(114 to 462) |  | 33.57(29.31 to 38.32) |  | 2(1 to 5) |  | 0.25(0.02 to 1.13) |
| Russian Federation | Endometriosis |  | 8,162(3,760 to 15,569) |  | 27.68(27.06 to 28.32) |  | 61(20 to 154) |  | 0.21(0.16 to 0.27) |
|  | Unexplained infertility |  | 521,503(230,649 to 1,010,499) |  | 1731.39(1726.49 to 1736.30) |  | 3,901(1,167 to 9,508) |  | 12.95(12.53 to 13.38) |
|  | Polycystic ovarian syndrome |  | 9,647(4,567 to 18,629) |  | 33.53(32.83 to 34.24) |  | 72(24 to 172) |  | 0.25(0.19 to 0.32) |
| Ukraine | Endometriosis |  | 2,850(1,393 to 5,352) |  | 31.74(30.52 to 32.99) |  | 21(7 to 53) |  | 0.24(0.14 to 0.38) |
|  | Unexplained infertility |  | 175,548(75,839 to 352,217) |  | 1949.80(1940.29 to 1959.35) |  | 1,311(387 to 3,227) |  | 14.57(13.75 to 15.42) |
|  | Polycystic ovarian syndrome |  | 3,025(1,476 to 5,737) |  | 34.77(33.47 to 36.11) |  | 23(7 to 54) |  | 0.26(0.16 to 0.41) |
| Brunei Darussalam | Endometriosis |  | 14(5 to 31) |  | 10.75(5.82 to 18.59) |  | 0(0 to 0) |  | 0.08(0.00 to 3.99) |
|  | Unexplained infertility |  | 79(17 to 258) |  | 60.10(47.59 to 75.24) |  | 1(0 to 2) |  | 0.46(0.00 to 4.59) |
|  | Polycystic ovarian syndrome |  | 299(117 to 641) |  | 235.77(209.74 to 264.42) |  | 2(1 to 6) |  | 1.76(0.25 to 6.62) |
| Japan | Endometriosis |  | 2,485(962 to 5,225) |  | 11.38(10.93 to 11.85) |  | 19(5 to 48) |  | 0.09(0.05 to 0.14) |
|  | Unexplained infertility |  | 31,457(6,172 to 93,354) |  | 123.78(122.38 to 125.19) |  | 238(32 to 817) |  | 0.93(0.82 to 1.07) |
|  | Polycystic ovarian syndrome |  | 84,546(35,436 to 166,810) |  | 390.96(388.29 to 393.64) |  | 630(199 to 1,436) |  | 2.91(2.69 to 3.16) |
| Republic of Korea | Endometriosis |  | 1,229(443 to 2,713) |  | 11.25(10.62 to 11.91) |  | 9(3 to 26) |  | 0.09(0.04 to 0.17) |
|  | Unexplained infertility |  | 8,827(1,660 to 29,549) |  | 70.54(69.05 to 72.06) |  | 67(9 to 251) |  | 0.54(0.41 to 0.69) |
|  | Polycystic ovarian syndrome |  | 22,777(8,821 to 46,569) |  | 211.05(208.29 to 213.85) |  | 169(51 to 410) |  | 1.57(1.34 to 1.83) |
| Singapore | Endometriosis |  | 128(49 to 283) |  | 9.39(7.74 to 11.38) |  | 1(0 to 3) |  | 0.07(0.00 to 0.67) |
|  | Unexplained infertility |  | 1,194(213 to 3,705) |  | 66.88(62.92 to 71.13) |  | 9(1 to 35) |  | 0.51(0.22 to 1.21) |
|  | Polycystic ovarian syndrome |  | 3,048(1,203 to 6,282) |  | 227.17(218.69 to 235.97) |  | 23(7 to 55) |  | 1.68(1.03 to 2.72) |
| Australia | Endometriosis |  | 654(226 to 1,413) |  | 11.21(10.36 to 12.12) |  | 5(1 to 13) |  | 0.08(0.03 to 0.21) |
|  | Unexplained infertility |  | 3,430(976 to 14,284) |  | 56.73(54.84 to 58.68) |  | 26(5 to 128) |  | 0.44(0.29 to 0.65) |
|  | Polycystic ovarian syndrome |  | 19,676(8,642 to 37,841) |  | 336.94(332.20 to 341.72) |  | 147(46 to 335) |  | 2.51(2.12 to 2.97) |
| New Zealand | Endometriosis |  | 207(81 to 428) |  | 17.57(15.25 to 20.17) |  | 2(0 to 4) |  | 0.13(0.01 to 0.61) |
|  | Unexplained infertility |  | 1,498(270 to 6,738) |  | 123.60(117.41 to 130.06) |  | 11(1 to 51) |  | 0.93(0.47 to 1.70) |
|  | Polycystic ovarian syndrome |  | 5,481(2,323 to 10,440) |  | 463.11(450.88 to 475.60) |  | 41(13 to 91) |  | 3.45(2.47 to 4.72) |
| Andorra | Endometriosis |  | 3(1 to 5) |  | 16.80(3.14 to 56.60) |  | 0(0 to 0) |  | 0.13(0.00 to 30.96) |
|  | Unexplained infertility |  | 118(31 to 280) |  | 586.09(481.58 to 710.28) |  | 1(0 to 3) |  | 4.40(0.06 to 37.37) |
|  | Polycystic ovarian syndrome |  | 89(42 to 157) |  | 504.95(402.02 to 629.03) |  | 1(0 to 1) |  | 3.77(0.01 to 36.90) |
| Austria | Endometriosis |  | 439(206 to 783) |  | 23.37(21.21 to 25.72) |  | 3(1 to 8) |  | 0.18(0.04 to 0.55) |
|  | Unexplained infertility |  | 25,639(9,545 to 56,567) |  | 1261.50(1245.99 to 1277.19) |  | 192(49 to 518) |  | 9.45(8.15 to 10.93) |
|  | Polycystic ovarian syndrome |  | 9,052(4,190 to 16,484) |  | 484.10(474.03 to 494.35) |  | 68(25 to 157) |  | 3.62(2.80 to 4.64) |
| Belgium | Endometriosis |  | 544(265 to 1,004) |  | 23.08(21.16 to 25.13) |  | 4(1 to 9) |  | 0.17(0.05 to 0.47) |
|  | Unexplained infertility |  | 31,230(11,062 to 66,619) |  | 1243.74(1229.88 to 1257.72) |  | 234(61 to 612) |  | 9.32(8.16 to 10.62) |
|  | Polycystic ovarian syndrome |  | 11,564(5,822 to 19,703) |  | 493.58(484.54 to 502.77) |  | 86(32 to 194) |  | 3.68(2.94 to 4.57) |
| Cyprus | Endometriosis |  | 62(28 to 111) |  | 18.07(13.72 to 23.66) |  | 0(0 to 1) |  | 0.13(0.00 to 2.13) |
|  | Unexplained infertility |  | 2,474(647 to 5,929) |  | 625.62(600.58 to 651.70) |  | 19(4 to 53) |  | 4.70(2.77 to 7.90) |
|  | Polycystic ovarian syndrome |  | 1,631(761 to 2,831) |  | 478.94(455.14 to 503.88) |  | 12(4 to 28) |  | 3.59(1.82 to 6.74) |
| Denmark | Endometriosis |  | 202(85 to 390) |  | 16.19(14.03 to 18.62) |  | 2(0 to 4) |  | 0.12(0.01 to 0.57) |
|  | Unexplained infertility |  | 3,255(338 to 11,220) |  | 257.11(248.33 to 266.15) |  | 25(2 to 92) |  | 1.94(1.25 to 2.91) |
|  | Polycystic ovarian syndrome |  | 6,230(2,986 to 10,964) |  | 499.35(486.98 to 511.98) |  | 46(17 to 101) |  | 3.72(2.73 to 4.99) |
| Finland | Endometriosis |  | 239(109 to 432) |  | 21.78(19.08 to 24.77) |  | 2(1 to 4) |  | 0.16(0.02 to 0.69) |
|  | Unexplained infertility |  | 5,749(1,336 to 15,008) |  | 485.75(473.19 to 498.59) |  | 43(7 to 136) |  | 3.66(2.65 to 4.97) |
|  | Polycystic ovarian syndrome |  | 5,294(2,621 to 9,160) |  | 482.38(469.36 to 495.69) |  | 39(15 to 88) |  | 3.59(2.55 to 4.94) |
| France | Endometriosis |  | 3,093(1,619 to 5,429) |  | 23.25(22.43 to 24.09) |  | 23(8 to 60) |  | 0.17(0.11 to 0.26) |
|  | Unexplained infertility |  | 221,333(76,297 to 466,864) |  | 1600.64(1593.93 to 1607.38) |  | 1,660(414 to 4,548) |  | 11.99(11.42 to 12.59) |
|  | Polycystic ovarian syndrome |  | 76,371(40,729 to 126,422) |  | 573.22(569.12 to 577.33) |  | 570(220 to 1,298) |  | 4.28(3.93 to 4.65) |
| Germany | Endometriosis |  | 3,472(1,532 to 6,589) |  | 20.86(20.17 to 21.58) |  | 26(8 to 64) |  | 0.16(0.10 to 0.23) |
|  | Unexplained infertility |  | 53,820(9,157 to 154,684) |  | 296.49(293.97 to 299.03) |  | 406(61 to 1,323) |  | 2.23(2.02 to 2.47) |
|  | Polycystic ovarian syndrome |  | 66,977(30,663 to 115,935) |  | 402.08(399.00 to 405.17) |  | 500(178 to 1,138) |  | 3.00(2.74 to 3.29) |
| Greece | Endometriosis |  | 334(151 to 629) |  | 17.53(15.66 to 19.58) |  | 2(1 to 7) |  | 0.13(0.02 to 0.46) |
|  | Unexplained infertility |  | 11,838(2,531 to 29,190) |  | 542.54(532.50 to 552.74) |  | 89(14 to 255) |  | 4.06(3.23 to 5.05) |
|  | Polycystic ovarian syndrome |  | 9,448(4,625 to 16,924) |  | 492.57(482.44 to 502.88) |  | 70(26 to 166) |  | 3.68(2.85 to 4.68) |
| Iceland | Endometriosis |  | 9(4 to 17) |  | 11.69(5.39 to 22.61) |  | 0(0 to 0) |  | 0.09(0.00 to 5.98) |
|  | Unexplained infertility |  | 496(117 to 1,257) |  | 604.29(552.18 to 660.38) |  | 4(1 to 11) |  | 4.55(1.16 to 12.81) |
|  | Polycystic ovarian syndrome |  | 406(193 to 709) |  | 514.63(465.58 to 567.78) |  | 3(1 to 7) |  | 3.85(0.80 to 11.92) |
| Ireland | Endometriosis |  | 190(87 to 356) |  | 18.11(15.58 to 20.94) |  | 1(0 to 3) |  | 0.14(0.01 to 0.65) |
|  | Unexplained infertility |  | 7,035(1,804 to 17,294) |  | 598.04(583.79 to 612.58) |  | 53(10 to 149) |  | 4.47(3.32 to 5.93) |
|  | Polycystic ovarian syndrome |  | 5,059(2,455 to 9,064) |  | 480.08(466.71 to 493.76) |  | 38(14 to 86) |  | 3.58(2.51 to 4.96) |
| Israel | Endometriosis |  | 478(200 to 909) |  | 22.25(20.30 to 24.34) |  | 4(1 to 9) |  | 0.17(0.04 to 0.45) |
|  | Unexplained infertility |  | 9,127(1,926 to 25,008) |  | 416.05(407.54 to 424.69) |  | 69(12 to 203) |  | 3.13(2.43 to 3.97) |
|  | Polycystic ovarian syndrome |  | 8,642(4,011 to 15,172) |  | 401.57(393.13 to 410.14) |  | 65(24 to 154) |  | 3.00(2.32 to 3.83) |
| Italy | Endometriosis |  | 1,550(666 to 2,960) |  | 14.83(14.09 to 15.60) |  | 12(3 to 29) |  | 0.11(0.06 to 0.20) |
|  | Unexplained infertility |  | 53,145(7,527 to 159,508) |  | 461.23(457.24 to 465.24) |  | 402(45 to 1,302) |  | 3.48(3.15 to 3.85) |
|  | Polycystic ovarian syndrome |  | 86,851(39,451 to 152,357) |  | 815.68(810.16 to 821.23) |  | 650(238 to 1,466) |  | 6.11(5.64 to 6.61) |
| Luxembourg | Endometriosis |  | 29(12 to 53) |  | 19.09(12.68 to 28.12) |  | 0(0 to 1) |  | 0.14(0.00 to 4.03) |
|  | Unexplained infertility |  | 913(207 to 2,404) |  | 550.37(514.85 to 588.15) |  | 7(1 to 20) |  | 4.12(1.61 to 9.48) |
|  | Polycystic ovarian syndrome |  | 762(364 to 1,327) |  | 507.31(471.32 to 545.69) |  | 6(2 to 13) |  | 3.79(1.31 to 9.24) |
| Malta | Endometriosis |  | 13(6 to 23) |  | 14.14(7.37 to 25.55) |  | 0(0 to 0) |  | 0.11(0.00 to 6.52) |
|  | Unexplained infertility |  | 608(154 to 1,493) |  | 606.07(558.19 to 657.73) |  | 5(1 to 13) |  | 4.54(1.35 to 12.72) |
|  | Polycystic ovarian syndrome |  | 450(217 to 774) |  | 500.61(454.53 to 550.73) |  | 3(1 to 8) |  | 3.74(0.83 to 11.89) |
| Netherlands | Endometriosis |  | 655(309 to 1,212) |  | 18.43(17.04 to 19.91) |  | 5(2 to 13) |  | 0.14(0.04 to 0.34) |
|  | Unexplained infertility |  | 23,219(5,989 to 57,412) |  | 633.69(625.55 to 641.92) |  | 174(31 to 515) |  | 4.76(4.08 to 5.53) |
|  | Polycystic ovarian syndrome |  | 16,005(7,768 to 27,983) |  | 450.55(443.57 to 457.62) |  | 119(45 to 275) |  | 3.36(2.79 to 4.03) |
| Norway | Endometriosis |  | 187(91 to 345) |  | 16.13(13.89 to 18.65) |  | 1(0 to 3) |  | 0.12(0.01 to 0.60) |
|  | Unexplained infertility |  | 12,129(3,248 to 29,662) |  | 1004.59(986.72 to 1022.72) |  | 91(18 to 266) |  | 7.56(6.08 to 9.31) |
|  | Polycystic ovarian syndrome |  | 5,333(2,639 to 9,233) |  | 462.31(449.91 to 474.98) |  | 40(15 to 96) |  | 3.45(2.46 to 4.74) |
| Portugal | Endometriosis |  | 266(123 to 488) |  | 12.89(11.36 to 14.58) |  | 2(1 to 5) |  | 0.10(0.01 to 0.39) |
|  | Unexplained infertility |  | 13,422(3,577 to 32,409) |  | 581.48(571.42 to 591.69) |  | 101(20 to 282) |  | 4.35(3.52 to 5.33) |
|  | Polycystic ovarian syndrome |  | 9,763(4,725 to 17,180) |  | 469.39(459.92 to 479.01) |  | 72(27 to 163) |  | 3.48(2.71 to 4.42) |
| Spain | Endometriosis |  | 973(424 to 1,815) |  | 11.34(10.62 to 12.10) |  | 7(2 to 19) |  | 0.08(0.03 to 0.18) |
|  | Unexplained infertility |  | 23,998(3,931 to 64,319) |  | 236.26(233.16 to 239.39) |  | 181(22 to 606) |  | 1.78(1.52 to 2.08) |
|  | Polycystic ovarian syndrome |  | 32,195(14,758 to 59,339) |  | 375.04(370.83 to 379.29) |  | 240(84 to 529) |  | 2.80(2.45 to 3.19) |
| Sweden | Endometriosis |  | 433(217 to 810) |  | 21.00(19.04 to 23.11) |  | 3(1 to 8) |  | 0.16(0.03 to 0.47) |
|  | Unexplained infertility |  | 25,327(8,162 to 58,146) |  | 1153.78(1139.48 to 1168.23) |  | 191(46 to 516) |  | 8.70(7.49 to 10.05) |
|  | Polycystic ovarian syndrome |  | 7,681(3,866 to 13,656) |  | 375.24(366.78 to 383.85) |  | 57(21 to 140) |  | 2.79(2.11 to 3.64) |
| Switzerland | Endometriosis |  | 418(185 to 776) |  | 22.40(20.27 to 24.72) |  | 3(1 to 8) |  | 0.17(0.03 to 0.55) |
|  | Unexplained infertility |  | 12,346(2,847 to 30,043) |  | 600.29(589.60 to 611.15) |  | 92(15 to 260) |  | 4.49(3.61 to 5.55) |
|  | Polycystic ovarian syndrome |  | 8,234(4,023 to 14,777) |  | 443.58(433.87 to 453.47) |  | 61(22 to 142) |  | 3.31(2.52 to 4.30) |
| United Kingdom | Endometriosis |  | 3,134(1,439 to 5,438) |  | 21.48(20.73 to 22.25) |  | 24(8 to 57) |  | 0.16(0.10 to 0.24) |
|  | Unexplained infertility |  | 120,670(22,045 to 317,924) |  | 778.32(773.91 to 782.75) |  | 908(131 to 2,830) |  | 5.86(5.48 to 6.26) |
|  | Polycystic ovarian syndrome |  | 78,263(37,376 to 134,386) |  | 531.83(528.08 to 535.60) |  | 585(213 to 1,364) |  | 3.97(3.65 to 4.31) |
| Argentina | Endometriosis |  | 1,455(647 to 2,777) |  | 12.29(11.67 to 12.94) |  | 11(3 to 29) |  | 0.09(0.05 to 0.17) |
|  | Unexplained infertility |  | 60,821(12,119 to 156,714) |  | 508.75(504.71 to 512.81) |  | 455(66 to 1,414) |  | 3.80(3.46 to 4.17) |
|  | Polycystic ovarian syndrome |  | 26,279(12,018 to 48,918) |  | 223.52(220.82 to 226.24) |  | 195(67 to 462) |  | 1.66(1.43 to 1.91) |
| Chile | Endometriosis |  | 789(387 to 1,385) |  | 16.45(15.32 to 17.65) |  | 6(2 to 14) |  | 0.12(0.04 to 0.28) |
|  | Unexplained infertility |  | 23,369(4,003 to 61,300) |  | 481.89(475.72 to 488.13) |  | 175(24 to 544) |  | 3.61(3.09 to 4.19) |
|  | Polycystic ovarian syndrome |  | 11,769(5,238 to 22,862) |  | 249.98(245.47 to 254.56) |  | 88(31 to 213) |  | 1.87(1.50 to 2.31) |
| Uruguay | Endometriosis |  | 119(52 to 217) |  | 14.56(12.06 to 17.46) |  | 1(0 to 2) |  | 0.11(0.00 to 0.71) |
|  | Unexplained infertility |  | 4,125(821 to 10,862) |  | 500.95(485.75 to 516.53) |  | 31(5 to 89) |  | 3.76(2.56 to 5.37) |
|  | Polycystic ovarian syndrome |  | 1,918(863 to 3,632) |  | 237.75(227.21 to 248.67) |  | 14(5 to 32) |  | 1.76(0.97 to 2.97) |
| Canada | Endometriosis |  | 1,148(541 to 2,209) |  | 13.82(13.02 to 14.65) |  | 9(3 to 22) |  | 0.10(0.05 to 0.21) |
|  | Unexplained infertility |  | 29,169(3,143 to 83,051) |  | 353.26(349.20 to 357.37) |  | 218(20 to 669) |  | 2.64(2.30 to 3.03) |
|  | Polycystic ovarian syndrome |  | 20,521(10,063 to 38,946) |  | 253.55(250.06 to 257.09) |  | 153(55 to 354) |  | 1.89(1.60 to 2.22) |
| United States of America | Endometriosis |  | 8,921(4,197 to 15,795) |  | 11.84(11.60 to 12.09) |  | 67(22 to 163) |  | 0.09(0.07 to 0.11) |
|  | Unexplained infertility |  | 445,164(67,358 to 1,222,787) |  | 590.56(588.82 to 592.30) |  | 3,336(379 to 10,193) |  | 4.43(4.28 to 4.58) |
|  | Polycystic ovarian syndrome |  | 383,664(186,975 to 682,837) |  | 517.37(515.73 to 519.01) |  | 2,856(1,073 to 6,341) |  | 3.85(3.71 to 4.00) |
| Antigua and Barbuda | Endometriosis |  | 3(1 to 6) |  | 12.42(2.45 to 39.32) |  | 0(0 to 0) |  | 0.09(0.00 to 19.72) |
|  | Unexplained infertility |  | 309(124 to 616) |  | 1292.58(1152.07 to 1446.73) |  | 2(1 to 6) |  | 9.66(1.43 to 35.05) |
|  | Polycystic ovarian syndrome |  | 44(20 to 83) |  | 193.74(140.42 to 261.75) |  | 0(0 to 1) |  | 1.44(0.00 to 22.15) |
| Bahamas | Endometriosis |  | 13(6 to 25) |  | 12.11(6.37 to 20.95) |  | 0(0 to 0) |  | 0.09(0.00 to 3.90) |
|  | Unexplained infertility |  | 1,324(541 to 2,710) |  | 1261.50(1194.36 to 1331.48) |  | 10(3 to 26) |  | 9.43(4.49 to 17.49) |
|  | Polycystic ovarian syndrome |  | 229(102 to 426) |  | 219.52(191.95 to 249.98) |  | 2(1 to 4) |  | 1.63(0.15 to 6.59) |
| Barbados | Endometriosis |  | 8(4 to 16) |  | 12.12(5.25 to 24.32) |  | 0(0 to 0) |  | 0.09(0.00 to 6.71) |
|  | Unexplained infertility |  | 869(354 to 1,745) |  | 1267.11(1183.85 to 1355.00) |  | 7(2 to 16) |  | 9.49(3.64 to 20.70) |
|  | Polycystic ovarian syndrome |  | 139(67 to 262) |  | 211.83(177.83 to 250.70) |  | 1(0 to 2) |  | 1.58(0.04 to 9.33) |
| Belize | Endometriosis |  | 20(9 to 38) |  | 16.03(9.76 to 25.04) |  | 0(0 to 0) |  | 0.12(0.00 to 3.76) |
|  | Unexplained infertility |  | 964(325 to 2,224) |  | 790.07(740.81 to 841.89) |  | 7(2 to 19) |  | 5.91(2.41 to 12.33) |
|  | Polycystic ovarian syndrome |  | 289(138 to 519) |  | 229.42(203.62 to 257.76) |  | 2(1 to 5) |  | 1.70(0.23 to 6.34) |
| Cuba | Endometriosis |  | 318(150 to 620) |  | 13.53(12.07 to 15.13) |  | 2(1 to 6) |  | 0.10(0.02 to 0.37) |
|  | Unexplained infertility |  | 29,735(12,334 to 58,888) |  | 1248.34(1234.13 to 1262.68) |  | 222(65 to 552) |  | 9.34(8.15 to 10.67) |
|  | Polycystic ovarian syndrome |  | 4,626(2,093 to 8,851) |  | 204.14(198.22 to 210.19) |  | 34(12 to 82) |  | 1.52(1.05 to 2.14) |
| Dominica | Endometriosis |  | 2(1 to 4) |  | 13.16(1.76 to 46.58) |  | 0(0 to 0) |  | 0.10(0.00 to 24.07) |
|  | Unexplained infertility |  | 209(85 to 426) |  | 1284.49(1116.20 to 1471.35) |  | 2(0 to 4) |  | 9.61(0.75 to 40.92) |
|  | Polycystic ovarian syndrome |  | 33(15 to 62) |  | 200.69(137.89 to 282.73) |  | 0(0 to 1) |  | 1.49(0.00 to 26.80) |
| Dominican Republic | Endometriosis |  | 420(187 to 836) |  | 14.07(12.75 to 15.48) |  | 3(1 to 8) |  | 0.11(0.02 to 0.31) |
|  | Unexplained infertility |  | 27,088(10,755 to 56,663) |  | 912.79(901.94 to 923.74) |  | 203(52 to 548) |  | 6.84(5.93 to 7.85) |
|  | Polycystic ovarian syndrome |  | 5,215(2,201 to 10,408) |  | 176.10(171.35 to 180.95) |  | 39(13 to 93) |  | 1.31(0.93 to 1.80) |
| Grenada | Endometriosis |  | 4(2 to 7) |  | 13.54(3.32 to 37.99) |  | 0(0 to 0) |  | 0.10(0.00 to 16.71) |
|  | Unexplained infertility |  | 338(133 to 701) |  | 1302.61(1166.83 to 1450.51) |  | 3(1 to 7) |  | 9.75(1.63 to 32.57) |
|  | Polycystic ovarian syndrome |  | 47(22 to 90) |  | 182.27(133.80 to 243.34) |  | 0(0 to 1) |  | 1.35(0.00 to 19.00) |
| Guyana | Endometriosis |  | 29(14 to 58) |  | 13.54(9.05 to 19.62) |  | 0(0 to 1) |  | 0.10(0.00 to 2.31) |
|  | Unexplained infertility |  | 3,515(1,479 to 7,193) |  | 1673.07(1617.79 to 1729.85) |  | 26(8 to 68) |  | 12.54(8.19 to 18.52) |
|  | Polycystic ovarian syndrome |  | 386(176 to 722) |  | 182.32(164.49 to 201.67) |  | 3(1 to 7) |  | 1.36(0.26 to 4.31) |
| Haiti | Endometriosis |  | 433(234 to 739) |  | 12.04(10.93 to 13.24) |  | 3(1 to 8) |  | 0.09(0.02 to 0.27) |
|  | Unexplained infertility |  | 23,159(16,165 to 33,103) |  | 639.81(631.59 to 648.12) |  | 174(68 to 375) |  | 4.81(4.12 to 5.59) |
|  | Polycystic ovarian syndrome |  | 3,345(1,816 to 5,660) |  | 92.44(89.33 to 95.64) |  | 25(9 to 59) |  | 0.68(0.44 to 1.02) |
| Jamaica | Endometriosis |  | 104(48 to 201) |  | 13.05(10.66 to 15.84) |  | 1(0 to 2) |  | 0.10(0.00 to 0.72) |
|  | Unexplained infertility |  | 12,191(5,118 to 24,651) |  | 1517.47(1490.61 to 1544.72) |  | 91(28 to 228) |  | 11.33(9.12 to 13.94) |
|  | Polycystic ovarian syndrome |  | 1,474(713 to 2,815) |  | 190.02(180.41 to 200.03) |  | 11(4 to 26) |  | 1.42(0.70 to 2.57) |
| Saint Lucia | Endometriosis |  | 5(3 to 11) |  | 12.43(4.27 to 29.16) |  | 0(0 to 0) |  | 0.09(0.00 to 10.59) |
|  | Unexplained infertility |  | 584(243 to 1,204) |  | 1296.10(1192.71 to 1406.68) |  | 4(1 to 11) |  | 9.71(2.83 to 25.25) |
|  | Polycystic ovarian syndrome |  | 80(38 to 149) |  | 186.54(147.53 to 233.26) |  | 1(0 to 1) |  | 1.38(0.00 to 12.87) |
| Saint Vincent and the Grenadines | Endometriosis |  | 4(2 to 7) |  | 13.57(3.44 to 36.57) |  | 0(0 to 0) |  | 0.10(0.00 to 14.73) |
|  | Unexplained infertility |  | 354(146 to 722) |  | 1293.13(1161.61 to 1435.76) |  | 3(1 to 7) |  | 9.67(1.71 to 30.75) |
|  | Polycystic ovarian syndrome |  | 52(24 to 98) |  | 191.54(142.80 to 251.82) |  | 0(0 to 1) |  | 1.42(0.00 to 17.27) |
| Suriname | Endometriosis |  | 22(10 to 42) |  | 15.47(9.71 to 23.46) |  | 0(0 to 0) |  | 0.12(0.00 to 2.97) |
|  | Unexplained infertility |  | 1,839(757 to 3,712) |  | 1279.97(1222.09 to 1339.92) |  | 14(4 to 35) |  | 9.58(5.20 to 16.22) |
|  | Polycystic ovarian syndrome |  | 279(129 to 513) |  | 196.79(174.36 to 221.34) |  | 2(1 to 5) |  | 1.46(0.19 to 5.26) |
| Trinidad and Tobago | Endometriosis |  | 44(21 to 88) |  | 13.82(9.98 to 18.72) |  | 0(0 to 1) |  | 0.10(0.00 to 1.57) |
|  | Unexplained infertility |  | 5,472(2,418 to 10,470) |  | 1638.57(1594.73 to 1683.37) |  | 41(13 to 100) |  | 12.24(8.73 to 16.78) |
|  | Polycystic ovarian syndrome |  | 680(327 to 1,282) |  | 219.71(203.27 to 237.16) |  | 5(2 to 12) |  | 1.64(0.53 to 3.93) |
| Bolivia (Plurinational State of) | Endometriosis |  | 311(112 to 659) |  | 9.63(8.59 to 10.77) |  | 2(1 to 6) |  | 0.07(0.01 to 0.25) |
|  | Unexplained infertility |  | 3,247(525 to 10,021) |  | 102.22(98.73 to 105.81) |  | 25(3 to 96) |  | 0.77(0.50 to 1.15) |
|  | Polycystic ovarian syndrome |  | 5,979(2,461 to 11,749) |  | 186.05(181.36 to 190.84) |  | 44(14 to 103) |  | 1.38(1.00 to 1.86) |
| Ecuador | Endometriosis |  | 376(137 to 833) |  | 7.74(6.98 to 8.57) |  | 3(1 to 7) |  | 0.06(0.01 to 0.18) |
|  | Unexplained infertility |  | 4,521(819 to 15,103) |  | 94.73(91.99 to 97.54) |  | 34(4 to 127) |  | 0.71(0.49 to 1.00) |
|  | Polycystic ovarian syndrome |  | 10,697(4,336 to 21,282) |  | 220.90(216.73 to 225.13) |  | 80(26 to 188) |  | 1.65(1.31 to 2.06) |
| Peru | Endometriosis |  | 663(251 to 1,473) |  | 6.68(6.18 to 7.21) |  | 5(2 to 14) |  | 0.05(0.02 to 0.12) |
|  | Unexplained infertility |  | 12,598(1,845 to 35,386) |  | 126.52(124.32 to 128.75) |  | 95(11 to 312) |  | 0.95(0.77 to 1.16) |
|  | Polycystic ovarian syndrome |  | 17,258(6,917 to 34,064) |  | 175.12(172.52 to 177.76) |  | 129(40 to 304) |  | 1.31(1.09 to 1.56) |
| Colombia | Endometriosis |  | 1,741(1,013 to 3,012) |  | 12.88(12.28 to 13.50) |  | 13(5 to 33) |  | 0.10(0.05 to 0.17) |
|  | Unexplained infertility |  | 20,685(4,594 to 41,890) |  | 152.24(150.17 to 154.33) |  | 157(29 to 425) |  | 1.16(0.98 to 1.36) |
|  | Polycystic ovarian syndrome |  | 33,085(20,545 to 52,437) |  | 246.72(244.07 to 249.40) |  | 247(94 to 583) |  | 1.85(1.62 to 2.09) |
| Costa Rica | Endometriosis |  | 124(53 to 254) |  | 9.46(7.86 to 11.31) |  | 1(0 to 3) |  | 0.07(0.00 to 0.47) |
|  | Unexplained infertility |  | 8,096(2,684 to 17,497) |  | 601.47(588.42 to 614.76) |  | 61(14 to 174) |  | 4.52(3.46 to 5.84) |
|  | Polycystic ovarian syndrome |  | 3,105(1,295 to 6,077) |  | 240.80(232.37 to 249.48) |  | 23(7 to 61) |  | 1.80(1.14 to 2.72) |
| El Salvador | Endometriosis |  | 157(56 to 346) |  | 8.37(7.11 to 9.80) |  | 1(0 to 3) |  | 0.06(0.00 to 0.34) |
|  | Unexplained infertility |  | 3,933(629 to 10,483) |  | 216.68(209.94 to 223.60) |  | 30(4 to 93) |  | 1.63(1.09 to 2.35) |
|  | Polycystic ovarian syndrome |  | 3,343(1,284 to 6,265) |  | 180.50(174.41 to 186.75) |  | 25(8 to 59) |  | 1.35(0.87 to 2.00) |
| Guatemala | Endometriosis |  | 389(143 to 832) |  | 8.30(7.49 to 9.19) |  | 3(1 to 7) |  | 0.06(0.01 to 0.20) |
|  | Unexplained infertility |  | 16,202(5,159 to 36,489) |  | 364.33(358.70 to 370.04) |  | 122(27 to 328) |  | 2.73(2.27 to 3.28) |
|  | Polycystic ovarian syndrome |  | 6,302(2,194 to 12,575) |  | 134.27(130.95 to 137.67) |  | 47(13 to 115) |  | 0.99(0.73 to 1.34) |
| Honduras | Endometriosis |  | 258(93 to 570) |  | 8.51(7.49 to 9.63) |  | 2(1 to 5) |  | 0.06(0.01 to 0.27) |
|  | Unexplained infertility |  | 12,323(3,838 to 27,575) |  | 424.92(417.40 to 432.56) |  | 92(21 to 250) |  | 3.18(2.56 to 3.92) |
|  | Polycystic ovarian syndrome |  | 4,357(1,565 to 9,000) |  | 143.77(139.50 to 148.14) |  | 32(9 to 84) |  | 1.07(0.73 to 1.53) |
| Mexico | Endometriosis |  | 3,177(1,258 to 6,706) |  | 9.14(8.82 to 9.46) |  | 24(7 to 61) |  | 0.07(0.04 to 0.10) |
|  | Unexplained infertility |  | 204,579(74,547 to 462,775) |  | 586.18(583.64 to 588.73) |  | 1,531(382 to 4,329) |  | 4.39(4.17 to 4.61) |
|  | Polycystic ovarian syndrome |  | 86,402(35,953 to 163,384) |  | 249.30(247.64 to 250.97) |  | 642(197 to 1,517) |  | 1.85(1.71 to 2.00) |
| Nicaragua | Endometriosis |  | 180(70 to 385) |  | 9.58(8.23 to 11.10) |  | 1(0 to 3) |  | 0.07(0.00 to 0.35) |
|  | Unexplained infertility |  | 5,202(1,174 to 12,931) |  | 279.14(271.60 to 286.85) |  | 39(6 to 115) |  | 2.09(1.49 to 2.88) |
|  | Polycystic ovarian syndrome |  | 3,391(1,386 to 6,642) |  | 181.33(175.27 to 187.56) |  | 25(8 to 62) |  | 1.35(0.88 to 2.01) |
| Panama | Endometriosis |  | 85(34 to 186) |  | 7.82(6.25 to 9.68) |  | 1(0 to 2) |  | 0.06(0.00 to 0.47) |
|  | Unexplained infertility |  | 5,475(2,074 to 11,479) |  | 510.53(497.08 to 524.25) |  | 41(10 to 111) |  | 3.82(2.74 to 5.19) |
|  | Polycystic ovarian syndrome |  | 1,718(668 to 3,407) |  | 158.77(151.34 to 166.47) |  | 13(4 to 32) |  | 1.19(0.63 to 2.05) |
| Venezuela (Bolivarian Republic of) | Endometriosis |  | 647(273 to 1,296) |  | 10.29(9.50 to 11.13) |  | 5(1 to 12) |  | 0.08(0.02 to 0.19) |
|  | Unexplained infertility |  | 32,763(10,649 to 70,017) |  | 492.46(487.08 to 497.90) |  | 247(56 to 655) |  | 3.71(3.25 to 4.21) |
|  | Polycystic ovarian syndrome |  | 13,849(5,895 to 27,623) |  | 218.36(214.70 to 222.08) |  | 103(35 to 267) |  | 1.62(1.32 to 1.97) |
| Brazil | Endometriosis |  | 10,491(4,977 to 19,322) |  | 18.25(17.90 to 18.60) |  | 79(26 to 195) |  | 0.14(0.11 to 0.17) |
|  | Unexplained infertility |  | 474,098(176,669 to 978,352) |  | 790.70(788.45 to 792.97) |  | 3,541(925 to 9,349) |  | 5.90(5.71 to 6.10) |
|  | Polycystic ovarian syndrome |  | 42,339(19,607 to 80,551) |  | 74.53(73.82 to 75.25) |  | 315(110 to 745) |  | 0.55(0.49 to 0.62) |
| Paraguay | Endometriosis |  | 293(140 to 565) |  | 15.09(13.41 to 16.93) |  | 2(1 to 5) |  | 0.11(0.02 to 0.42) |
|  | Unexplained infertility |  | 18,828(8,085 to 36,411) |  | 977.08(963.16 to 991.17) |  | 141(44 to 346) |  | 7.32(6.16 to 8.65) |
|  | Polycystic ovarian syndrome |  | 1,412(617 to 2,724) |  | 72.71(68.96 to 76.62) |  | 10(3 to 26) |  | 0.54(0.26 to 1.00) |
| Algeria | Endometriosis |  | 2,611(1,220 to 4,960) |  | 23.49(22.59 to 24.42) |  | 20(6 to 49) |  | 0.18(0.11 to 0.28) |
|  | Unexplained infertility |  | 94,223(37,054 to 200,774) |  | 836.25(830.87 to 841.65) |  | 705(181 to 1,879) |  | 6.26(5.80 to 6.74) |
|  | Polycystic ovarian syndrome |  | 29,430(13,327 to 55,406) |  | 271.41(268.29 to 274.55) |  | 219(70 to 510) |  | 2.02(1.76 to 2.31) |
| Bahrain | Endometriosis |  | 65(30 to 124) |  | 19.89(15.34 to 25.43) |  | 0(0 to 1) |  | 0.15(0.00 to 1.60) |
|  | Unexplained infertility |  | 3,159(1,266 to 6,553) |  | 957.05(923.90 to 991.15) |  | 24(6 to 63) |  | 7.16(4.56 to 10.78) |
|  | Polycystic ovarian syndrome |  | 978(465 to 1,722) |  | 305.53(286.62 to 325.42) |  | 7(3 to 17) |  | 2.26(0.92 to 4.71) |
| Egypt | Endometriosis |  | 8,188(4,181 to 15,194) |  | 30.86(30.20 to 31.54) |  | 62(19 to 151) |  | 0.23(0.18 to 0.30) |
|  | Unexplained infertility |  | 321,840(129,186 to 684,618) |  | 1211.73(1207.55 to 1215.93) |  | 2,400(656 to 6,239) |  | 9.04(8.68 to 9.41) |
|  | Polycystic ovarian syndrome |  | 93,015(44,657 to 161,545) |  | 348.43(346.19 to 350.68) |  | 692(239 to 1,582) |  | 2.59(2.40 to 2.80) |
| Iran (Islamic Republic of) | Endometriosis |  | 7,733(4,324 to 13,341) |  | 34.18(33.40 to 34.97) |  | 58(21 to 137) |  | 0.26(0.19 to 0.34) |
|  | Unexplained infertility |  | 394,189(150,901 to 862,521) |  | 1752.64(1747.01 to 1758.29) |  | 2,949(710 to 7,968) |  | 13.11(12.63 to 13.61) |
|  | Polycystic ovarian syndrome |  | 101,672(56,990 to 174,921) |  | 463.94(461.01 to 466.89) |  | 758(287 to 1,667) |  | 3.46(3.21 to 3.72) |
| Iraq | Endometriosis |  | 2,839(1,371 to 5,436) |  | 26.20(25.24 to 27.19) |  | 21(6 to 53) |  | 0.20(0.12 to 0.30) |
|  | Unexplained infertility |  | 104,704(40,338 to 227,499) |  | 980.77(974.81 to 986.76) |  | 782(206 to 2,028) |  | 7.33(6.82 to 7.86) |
|  | Polycystic ovarian syndrome |  | 28,874(13,416 to 52,592) |  | 262.67(259.63 to 265.73) |  | 214(72 to 489) |  | 1.94(1.69 to 2.23) |
| Jordan | Endometriosis |  | 848(481 to 1,425) |  | 26.91(25.12 to 28.80) |  | 6(2 to 16) |  | 0.20(0.08 to 0.44) |
|  | Unexplained infertility |  | 26,405(16,893 to 40,569) |  | 857.55(847.20 to 868.00) |  | 199(73 to 436) |  | 6.46(5.59 to 7.43) |
|  | Polycystic ovarian syndrome |  | 8,658(5,042 to 14,412) |  | 266.27(260.66 to 271.98) |  | 65(25 to 147) |  | 2.00(1.54 to 2.56) |
| Kuwait | Endometriosis |  | 281(134 to 535) |  | 20.09(17.69 to 22.79) |  | 2(1 to 6) |  | 0.15(0.02 to 0.72) |
|  | Unexplained infertility |  | 13,310(5,108 to 26,974) |  | 904.64(888.43 to 921.14) |  | 99(27 to 260) |  | 6.76(5.42 to 8.40) |
|  | Polycystic ovarian syndrome |  | 4,453(2,054 to 7,961) |  | 338.86(328.30 to 349.73) |  | 33(12 to 73) |  | 2.52(1.69 to 3.70) |
| Lebanon | Endometriosis |  | 342(154 to 676) |  | 22.95(20.53 to 25.60) |  | 3(1 to 6) |  | 0.17(0.03 to 0.61) |
|  | Unexplained infertility |  | 16,804(7,325 to 33,285) |  | 1125.99(1108.64 to 1143.56) |  | 126(36 to 313) |  | 8.41(6.98 to 10.09) |
|  | Polycystic ovarian syndrome |  | 3,674(1,587 to 6,848) |  | 258.55(250.05 to 267.27) |  | 27(9 to 65) |  | 1.93(1.26 to 2.85) |
| Libya | Endometriosis |  | 434(220 to 804) |  | 22.50(20.43 to 24.73) |  | 3(1 to 8) |  | 0.17(0.04 to 0.49) |
|  | Unexplained infertility |  | 18,865(7,260 to 40,487) |  | 962.48(948.74 to 976.37) |  | 141(35 to 369) |  | 7.18(6.04 to 8.48) |
|  | Polycystic ovarian syndrome |  | 5,912(2,742 to 10,623) |  | 309.76(301.89 to 317.79) |  | 44(15 to 103) |  | 2.31(1.68 to 3.11) |
| Morocco | Endometriosis |  | 2,371(1,179 to 4,356) |  | 24.63(23.65 to 25.64) |  | 18(6 to 43) |  | 0.18(0.11 to 0.29) |
|  | Unexplained infertility |  | 134,386(53,591 to 273,327) |  | 1390.93(1383.50 to 1398.40) |  | 1,005(290 to 2,626) |  | 10.40(9.77 to 11.06) |
|  | Polycystic ovarian syndrome |  | 29,014(14,595 to 51,462) |  | 303.39(299.90 to 306.90) |  | 216(75 to 501) |  | 2.26(1.97 to 2.58) |
| Palestine | Endometriosis |  | 380(178 to 712) |  | 27.47(24.75 to 30.45) |  | 3(1 to 7) |  | 0.21(0.04 to 0.70) |
|  | Unexplained infertility |  | 13,458(4,920 to 29,464) |  | 996.91(979.91 to 1014.15) |  | 101(28 to 255) |  | 7.45(6.05 to 9.11) |
|  | Polycystic ovarian syndrome |  | 3,504(1,574 to 6,479) |  | 248.94(240.67 to 257.45) |  | 26(8 to 59) |  | 1.85(1.20 to 2.77) |
| Oman | Endometriosis |  | 226(108 to 439) |  | 21.73(18.94 to 24.85) |  | 2(1 to 4) |  | 0.16(0.01 to 0.73) |
|  | Unexplained infertility |  | 10,012(3,932 to 20,390) |  | 950.88(932.03 to 970.04) |  | 75(20 to 198) |  | 7.11(5.56 to 8.98) |
|  | Polycystic ovarian syndrome |  | 2,993(1,351 to 5,521) |  | 299.06(288.24 to 310.21) |  | 22(8 to 51) |  | 2.22(1.38 to 3.42) |
| Qatar | Endometriosis |  | 128(59 to 242) |  | 22.98(18.91 to 27.83) |  | 1(0 to 2) |  | 0.17(0.00 to 1.48) |
|  | Unexplained infertility |  | 5,308(2,004 to 10,798) |  | 923.64(897.00 to 951.01) |  | 40(10 to 99) |  | 6.89(4.77 to 9.82) |
|  | Polycystic ovarian syndrome |  | 1,710(745 to 3,167) |  | 327.77(311.14 to 345.17) |  | 13(4 to 29) |  | 2.44(1.21 to 4.58) |
| Saudi Arabia | Endometriosis |  | 1,966(923 to 3,780) |  | 19.16(18.31 to 20.04) |  | 15(5 to 36) |  | 0.14(0.08 to 0.25) |
|  | Unexplained infertility |  | 97,993(40,164 to 199,014) |  | 931.91(926.01 to 937.84) |  | 732(199 to 1,912) |  | 6.96(6.46 to 7.50) |
|  | Polycystic ovarian syndrome |  | 31,377(14,436 to 57,039) |  | 319.83(316.23 to 323.47) |  | 232(81 to 505) |  | 2.37(2.07 to 2.70) |
| Syrian Arab Republic | Endometriosis |  | 739(356 to 1,386) |  | 21.79(20.13 to 23.57) |  | 5(2 to 13) |  | 0.16(0.05 to 0.42) |
|  | Unexplained infertility |  | 28,692(10,945 to 62,357) |  | 792.40(782.65 to 802.27) |  | 215(54 to 560) |  | 5.92(5.11 to 6.85) |
|  | Polycystic ovarian syndrome |  | 8,919(3,948 to 17,129) |  | 238.66(233.34 to 244.09) |  | 66(21 to 157) |  | 1.77(1.34 to 2.31) |
| Tunisia | Endometriosis |  | 742(379 to 1,392) |  | 25.21(23.41 to 27.12) |  | 6(2 to 14) |  | 0.19(0.07 to 0.44) |
|  | Unexplained infertility |  | 32,694(13,111 to 66,579) |  | 1086.67(1074.77 to 1098.68) |  | 245(67 to 642) |  | 8.14(7.14 to 9.25) |
|  | Polycystic ovarian syndrome |  | 8,651(4,080 to 15,330) |  | 301.36(294.96 to 307.86) |  | 64(22 to 146) |  | 2.24(1.72 to 2.88) |
| Türkiye | Endometriosis |  | 4,519(2,199 to 8,590) |  | 21.36(20.74 to 22.00) |  | 34(11 to 90) |  | 0.16(0.11 to 0.22) |
|  | Unexplained infertility |  | 192,979(74,505 to 407,657) |  | 900.78(896.75 to 904.82) |  | 1,443(401 to 3,904) |  | 6.73(6.39 to 7.09) |
|  | Polycystic ovarian syndrome |  | 51,045(22,970 to 92,885) |  | 246.14(244.00 to 248.29) |  | 381(128 to 899) |  | 1.84(1.66 to 2.03) |
| United Arab Emirates | Endometriosis |  | 253(123 to 470) |  | 18.47(16.03 to 21.22) |  | 2(1 to 5) |  | 0.14(0.01 to 0.66) |
|  | Unexplained infertility |  | 15,719(6,664 to 29,435) |  | 952.58(935.48 to 969.95) |  | 118(34 to 279) |  | 7.11(5.71 to 8.81) |
|  | Polycystic ovarian syndrome |  | 4,100(1,929 to 7,764) |  | 301.48(291.39 to 311.86) |  | 31(11 to 71) |  | 2.25(1.46 to 3.36) |
| Yemen | Endometriosis |  | 2,766(1,324 to 5,229) |  | 32.66(31.45 to 33.92) |  | 20(7 to 51) |  | 0.24(0.15 to 0.38) |
|  | Unexplained infertility |  | 123,183(50,953 to 258,289) |  | 1448.26(1440.14 to 1456.42) |  | 917(289 to 2,313) |  | 10.78(10.09 to 11.51) |
|  | Polycystic ovarian syndrome |  | 15,570(7,101 to 29,013) |  | 176.72(173.92 to 179.55) |  | 115(37 to 279) |  | 1.31(1.08 to 1.58) |
| Afghanistan | Endometriosis |  | 1,668(897 to 2,921) |  | 21.68(20.61 to 22.80) |  | 13(4 to 32) |  | 0.16(0.08 to 0.30) |
|  | Unexplained infertility |  | 21,752(12,715 to 34,388) |  | 294.14(290.07 to 298.26) |  | 164(58 to 366) |  | 2.22(1.88 to 2.61) |
|  | Polycystic ovarian syndrome |  | 9,100(4,972 to 15,740) |  | 109.92(107.58 to 112.32) |  | 68(24 to 152) |  | 0.82(0.63 to 1.06) |
| Bangladesh | Endometriosis |  | 7,405(4,285 to 11,855) |  | 15.80(15.44 to 16.17) |  | 56(20 to 137) |  | 0.12(0.09 to 0.15) |
|  | Unexplained infertility |  | 448,736(305,594 to 607,963) |  | 925.56(922.85 to 928.27) |  | 3,365(1,333 to 6,992) |  | 6.94(6.71 to 7.18) |
|  | Polycystic ovarian syndrome |  | 35,696(20,498 to 57,309) |  | 76.27(75.48 to 77.07) |  | 266(97 to 594) |  | 0.57(0.50 to 0.64) |
| Bhutan | Endometriosis |  | 37(18 to 72) |  | 17.41(12.26 to 24.17) |  | 0(0 to 1) |  | 0.13(0.00 to 2.41) |
|  | Unexplained infertility |  | 1,701(633 to 3,510) |  | 784.69(747.79 to 823.07) |  | 13(3 to 32) |  | 5.85(3.09 to 10.30) |
|  | Polycystic ovarian syndrome |  | 256(118 to 470) |  | 121.32(106.87 to 137.30) |  | 2(1 to 4) |  | 0.90(0.10 to 3.65) |
| India | Endometriosis |  | 67,041(32,318 to 128,088) |  | 17.51(17.38 to 17.64) |  | 501(171 to 1,199) |  | 0.13(0.12 to 0.14) |
|  | Unexplained infertility |  | 7,061,387(3,199,668 to 14,402,195) |  | 1834.96(1833.61 to 1836.32) |  | 52,628(16,382 to 128,639) |  | 13.68(13.56 to 13.79) |
|  | Polycystic ovarian syndrome |  | 531,787(267,713 to 989,701) |  | 139.07(138.69 to 139.44) |  | 3,936(1,384 to 9,100) |  | 1.03(1.00 to 1.06) |
| Nepal | Endometriosis |  | 1,850(1,090 to 3,054) |  | 19.50(18.61 to 20.41) |  | 14(5 to 37) |  | 0.15(0.08 to 0.25) |
|  | Unexplained infertility |  | 57,671(37,310 to 82,841) |  | 579.44(574.71 to 584.21) |  | 433(170 to 914) |  | 4.35(3.95 to 4.78) |
|  | Polycystic ovarian syndrome |  | 9,237(5,364 to 15,168) |  | 96.99(95.02 to 99.00) |  | 69(26 to 155) |  | 0.73(0.56 to 0.92) |
| Pakistan | Endometriosis |  | 24,284(8,127 to 48,109) |  | 38.80(38.31 to 39.29) |  | 182(49 to 477) |  | 0.29(0.25 to 0.34) |
|  | Unexplained infertility |  | 1,976,983(358,206 to 5,730,702) |  | 3123.79(3119.42 to 3128.16) |  | 14,589(2,076 to 54,156) |  | 23.06(22.68 to 23.44) |
|  | Polycystic ovarian syndrome |  | 81,795(27,892 to 160,483) |  | 129.68(128.79 to 130.58) |  | 608(159 to 1,460) |  | 0.96(0.89 to 1.04) |
| Angola | Endometriosis |  | 1,443(601 to 2,914) |  | 17.80(16.89 to 18.77) |  | 11(3 to 28) |  | 0.13(0.06 to 0.25) |
|  | Unexplained infertility |  | 89,453(38,062 to 180,202) |  | 1167.38(1159.64 to 1175.15) |  | 667(184 to 1,657) |  | 8.70(8.05 to 9.41) |
|  | Polycystic ovarian syndrome |  | 5,970(2,329 to 11,979) |  | 69.78(67.99 to 71.60) |  | 44(13 to 113) |  | 0.52(0.38 to 0.71) |
| Central African Republic | Endometriosis |  | 291(129 to 573) |  | 20.03(17.77 to 22.55) |  | 2(1 to 6) |  | 0.15(0.02 to 0.61) |
|  | Unexplained infertility |  | 35,845(15,923 to 68,689) |  | 2571.35(2544.44 to 2598.50) |  | 267(82 to 636) |  | 19.13(16.87 to 21.64) |
|  | Polycystic ovarian syndrome |  | 1,057(469 to 1,989) |  | 69.44(65.24 to 73.87) |  | 8(2 to 20) |  | 0.52(0.22 to 1.11) |
| Congo | Endometriosis |  | 152(55 to 333) |  | 10.40(8.81 to 12.21) |  | 1(0 to 3) |  | 0.08(0.00 to 0.45) |
|  | Unexplained infertility |  | 9,363(3,770 to 18,607) |  | 654.93(641.71 to 668.36) |  | 70(21 to 177) |  | 4.88(3.80 to 6.19) |
|  | Polycystic ovarian syndrome |  | 870(300 to 1,759) |  | 57.88(54.09 to 61.89) |  | 7(2 to 17) |  | 0.43(0.17 to 0.95) |
| Democratic Republic of the Congo | Endometriosis |  | 3,668(1,500 to 7,277) |  | 16.09(15.57 to 16.63) |  | 27(8 to 72) |  | 0.12(0.08 to 0.18) |
|  | Unexplained infertility |  | 144,990(56,701 to 299,072) |  | 675.59(672.06 to 679.13) |  | 1,081(281 to 2,736) |  | 5.04(4.74 to 5.36) |
|  | Polycystic ovarian syndrome |  | 14,296(5,814 to 27,975) |  | 59.77(58.78 to 60.78) |  | 106(32 to 277) |  | 0.44(0.36 to 0.54) |
| Equatorial Guinea | Endometriosis |  | 52(20 to 109) |  | 13.14(9.77 to 17.52) |  | 0(0 to 1) |  | 0.10(0.00 to 1.71) |
|  | Unexplained infertility |  | 4,296(1,804 to 8,764) |  | 1149.89(1115.40 to 1185.33) |  | 32(9 to 82) |  | 8.59(5.85 to 12.39) |
|  | Polycystic ovarian syndrome |  | 408(165 to 787) |  | 100.91(91.26 to 111.50) |  | 3(1 to 8) |  | 0.75(0.15 to 2.63) |
| Gabon | Endometriosis |  | 60(24 to 127) |  | 11.59(8.83 to 15.03) |  | 0(0 to 1) |  | 0.09(0.00 to 1.12) |
|  | Unexplained infertility |  | 7,753(3,297 to 14,668) |  | 1564.54(1529.58 to 1600.15) |  | 58(17 to 146) |  | 11.65(8.82 to 15.18) |
|  | Polycystic ovarian syndrome |  | 487(197 to 951) |  | 91.02(83.04 to 99.64) |  | 4(1 to 9) |  | 0.67(0.16 to 1.99) |
| Burundi | Endometriosis |  | 507(275 to 878) |  | 15.67(14.31 to 17.15) |  | 4(1 to 10) |  | 0.12(0.03 to 0.38) |
|  | Unexplained infertility |  | 4,383(2,312 to 7,124) |  | 140.32(136.15 to 144.62) |  | 33(12 to 75) |  | 1.05(0.72 to 1.52) |
|  | Polycystic ovarian syndrome |  | 1,203(621 to 2,057) |  | 36.08(34.03 to 38.25) |  | 9(3 to 21) |  | 0.27(0.12 to 0.58) |
| Comoros | Endometriosis |  | 33(16 to 62) |  | 16.34(11.22 to 23.10) |  | 0(0 to 1) |  | 0.12(0.00 to 2.37) |
|  | Unexplained infertility |  | 5,376(2,468 to 10,595) |  | 2704.05(2631.99 to 2777.66) |  | 40(12 to 96) |  | 20.14(14.37 to 27.55) |
|  | Polycystic ovarian syndrome |  | 220(103 to 399) |  | 107.71(93.90 to 123.08) |  | 2(1 to 4) |  | 0.80(0.07 to 3.49) |
| Djibouti | Endometriosis |  | 38(16 to 76) |  | 11.58(8.17 to 16.01) |  | 0(0 to 1) |  | 0.09(0.00 to 1.52) |
|  | Unexplained infertility |  | 5,394(2,557 to 10,179) |  | 1633.11(1589.75 to 1677.42) |  | 40(13 to 94) |  | 12.17(8.70 to 16.66) |
|  | Polycystic ovarian syndrome |  | 240(98 to 476) |  | 74.94(65.75 to 85.12) |  | 2(1 to 5) |  | 0.56(0.06 to 2.30) |
| Eritrea | Endometriosis |  | 232(95 to 467) |  | 13.27(11.60 to 15.13) |  | 2(1 to 4) |  | 0.10(0.01 to 0.45) |
|  | Unexplained infertility |  | 29,659(13,518 to 59,211) |  | 1734.72(1714.89 to 1754.74) |  | 221(71 to 523) |  | 12.93(11.27 to 14.79) |
|  | Polycystic ovarian syndrome |  | 1,068(434 to 2,035) |  | 59.71(56.16 to 63.46) |  | 8(2 to 20) |  | 0.44(0.19 to 0.93) |
| Ethiopia | Endometriosis |  | 4,282(1,762 to 8,371) |  | 14.19(13.76 to 14.63) |  | 32(10 to 82) |  | 0.11(0.07 to 0.16) |
|  | Unexplained infertility |  | 201,723(78,933 to 438,909) |  | 688.18(685.13 to 691.24) |  | 1,500(431 to 3,640) |  | 5.12(4.86 to 5.39) |
|  | Polycystic ovarian syndrome |  | 18,599(7,662 to 35,846) |  | 59.17(58.31 to 60.05) |  | 138(41 to 337) |  | 0.44(0.37 to 0.52) |
| Kenya | Endometriosis |  | 1,467(563 to 3,091) |  | 10.27(9.74 to 10.82) |  | 11(3 to 28) |  | 0.08(0.04 to 0.15) |
|  | Unexplained infertility |  | 94,112(38,007 to 197,491) |  | 680.41(676.01 to 684.82) |  | 703(202 to 1,816) |  | 5.08(4.71 to 5.48) |
|  | Polycystic ovarian syndrome |  | 8,810(3,586 to 17,428) |  | 59.42(58.16 to 60.69) |  | 65(19 to 164) |  | 0.44(0.34 to 0.57) |
| Madagascar | Endometriosis |  | 1,272(594 to 2,436) |  | 16.58(15.66 to 17.53) |  | 9(3 to 24) |  | 0.12(0.06 to 0.24) |
|  | Unexplained infertility |  | 97,362(41,254 to 200,873) |  | 1299.92(1291.64 to 1308.25) |  | 724(207 to 1,779) |  | 9.67(8.97 to 10.42) |
|  | Polycystic ovarian syndrome |  | 5,562(2,390 to 10,428) |  | 69.19(67.35 to 71.07) |  | 41(13 to 97) |  | 0.51(0.37 to 0.71) |
| Malawi | Endometriosis |  | 358(198 to 624) |  | 6.73(6.03 to 7.50) |  | 3(1 to 6) |  | 0.05(0.01 to 0.20) |
|  | Unexplained infertility |  | 6,389(3,353 to 10,953) |  | 114.46(111.63 to 117.36) |  | 48(17 to 104) |  | 0.86(0.63 to 1.17) |
|  | Polycystic ovarian syndrome |  | 2,359(1,153 to 4,261) |  | 40.90(39.21 to 42.66) |  | 17(5 to 45) |  | 0.30(0.17 to 0.52) |
| Mauritius | Endometriosis |  | 42(19 to 85) |  | 13.63(9.83 to 18.50) |  | 0(0 to 1) |  | 0.10(0.00 to 1.58) |
|  | Unexplained infertility |  | 1,947(676 to 4,225) |  | 614.56(587.44 to 642.67) |  | 15(4 to 37) |  | 4.59(2.54 to 7.74) |
|  | Polycystic ovarian syndrome |  | 1,072(490 to 2,019) |  | 350.86(330.08 to 372.65) |  | 8(3 to 18) |  | 2.61(1.12 to 5.25) |
| Mozambique | Endometriosis |  | 1,191(527 to 2,406) |  | 14.46(13.63 to 15.34) |  | 9(3 to 21) |  | 0.11(0.05 to 0.23) |
|  | Unexplained infertility |  | 112,580(48,920 to 222,636) |  | 1432.59(1423.99 to 1441.24) |  | 836(254 to 2,050) |  | 10.64(9.91 to 11.41) |
|  | Polycystic ovarian syndrome |  | 5,915(2,279 to 11,788) |  | 67.47(65.71 to 69.28) |  | 43(13 to 110) |  | 0.49(0.35 to 0.68) |
| Rwanda | Endometriosis |  | 429(170 to 910) |  | 11.61(10.54 to 12.79) |  | 3(1 to 9) |  | 0.09(0.02 to 0.28) |
|  | Unexplained infertility |  | 8,474(2,045 to 21,045) |  | 231.10(226.18 to 236.11) |  | 63(11 to 177) |  | 1.72(1.32 to 2.22) |
|  | Polycystic ovarian syndrome |  | 2,350(884 to 4,682) |  | 61.73(59.24 to 64.31) |  | 17(5 to 44) |  | 0.46(0.27 to 0.75) |
| Seychelles | Endometriosis |  | 4(2 to 8) |  | 16.81(4.44 to 44.67) |  | 0(0 to 0) |  | 0.13(0.00 to 17.97) |
|  | Unexplained infertility |  | 150(54 to 302) |  | 627.17(530.48 to 737.10) |  | 1(0 to 3) |  | 4.69(0.16 to 25.95) |
|  | Polycystic ovarian syndrome |  | 78(36 to 140) |  | 340.28(268.57 to 425.74) |  | 1(0 to 1) |  | 2.53(0.01 to 22.44) |
| Somalia | Endometriosis |  | 1,061(461 to 2,188) |  | 20.97(19.70 to 22.31) |  | 8(2 to 22) |  | 0.16(0.07 to 0.34) |
|  | Unexplained infertility |  | 42,679(18,063 to 86,780) |  | 867.64(859.26 to 876.09) |  | 318(94 to 788) |  | 6.46(5.76 to 7.24) |
|  | Polycystic ovarian syndrome |  | 2,974(1,208 to 5,946) |  | 54.50(52.51 to 56.56) |  | 22(6 to 56) |  | 0.41(0.25 to 0.64) |
| United Republic of Tanzania | Endometriosis |  | 1,702(952 to 2,890) |  | 11.07(10.54 to 11.62) |  | 13(4 to 32) |  | 0.08(0.04 to 0.15) |
|  | Unexplained infertility |  | 73,738(54,020 to 99,017) |  | 508.73(505.01 to 512.47) |  | 554(235 to 1,137) |  | 3.82(3.50 to 4.16) |
|  | Polycystic ovarian syndrome |  | 8,274(4,378 to 14,633) |  | 50.99(49.88 to 52.13) |  | 62(19 to 160) |  | 0.38(0.29 to 0.49) |
| Uganda | Endometriosis |  | 1,159(637 to 2,054) |  | 10.35(9.74 to 10.99) |  | 9(3 to 22) |  | 0.08(0.03 to 0.17) |
|  | Unexplained infertility |  | 17,078(9,463 to 28,097) |  | 151.71(149.40 to 154.05) |  | 129(46 to 291) |  | 1.14(0.95 to 1.37) |
|  | Polycystic ovarian syndrome |  | 5,881(2,902 to 10,401) |  | 48.54(47.26 to 49.86) |  | 43(14 to 108) |  | 0.36(0.26 to 0.50) |
| Zambia | Endometriosis |  | 610(258 to 1,282) |  | 11.33(10.43 to 12.30) |  | 5(1 to 12) |  | 0.08(0.02 to 0.24) |
|  | Unexplained infertility |  | 29,795(11,876 to 63,330) |  | 576.88(570.23 to 583.61) |  | 221(59 to 558) |  | 4.29(3.73 to 4.92) |
|  | Polycystic ovarian syndrome |  | 4,054(1,575 to 7,956) |  | 72.09(69.84 to 74.40) |  | 30(8 to 78) |  | 0.53(0.35 to 0.78) |
| Botswana | Endometriosis |  | 69(26 to 150) |  | 10.06(7.83 to 12.77) |  | 1(0 to 1) |  | 0.08(0.00 to 0.78) |
|  | Unexplained infertility |  | 3,537(1,339 to 7,464) |  | 502.04(485.60 to 518.92) |  | 26(7 to 67) |  | 3.74(2.45 to 5.52) |
|  | Polycystic ovarian syndrome |  | 508(193 to 1,010) |  | 74.50(68.15 to 81.31) |  | 4(1 to 10) |  | 0.55(0.14 to 1.51) |
| Lesotho | Endometriosis |  | 88(38 to 179) |  | 16.22(12.97 to 20.17) |  | 1(0 to 2) |  | 0.12(0.00 to 1.27) |
|  | Unexplained infertility |  | 5,815(2,278 to 11,823) |  | 1105.50(1076.97 to 1134.70) |  | 43(13 to 112) |  | 8.24(5.95 to 11.26) |
|  | Polycystic ovarian syndrome |  | 565(243 to 1,072) |  | 101.81(93.49 to 110.79) |  | 4(1 to 11) |  | 0.75(0.21 to 2.17) |
| Namibia | Endometriosis |  | 111(44 to 228) |  | 15.93(13.09 to 19.26) |  | 1(0 to 2) |  | 0.12(0.00 to 0.91) |
|  | Unexplained infertility |  | 3,719(1,191 to 8,577) |  | 543.20(525.81 to 561.07) |  | 28(7 to 76) |  | 4.05(2.68 to 5.94) |
|  | Polycystic ovarian syndrome |  | 602(252 to 1,153) |  | 86.15(79.38 to 93.40) |  | 4(1 to 11) |  | 0.64(0.19 to 1.68) |
| South Africa | Endometriosis |  | 2,727(1,161 to 5,349) |  | 17.54(16.89 to 18.22) |  | 20(6 to 52) |  | 0.13(0.08 to 0.20) |
|  | Unexplained infertility |  | 106,682(33,433 to 244,984) |  | 661.16(657.19 to 665.15) |  | 795(205 to 2,194) |  | 4.93(4.59 to 5.29) |
|  | Polycystic ovarian syndrome |  | 19,449(8,414 to 37,590) |  | 126.94(125.15 to 128.74) |  | 144(49 to 360) |  | 0.94(0.79 to 1.11) |
| Eswatini | Endometriosis |  | 52(22 to 103) |  | 15.39(11.46 to 20.48) |  | 0(0 to 1) |  | 0.11(0.00 to 1.96) |
|  | Unexplained infertility |  | 1,750(502 to 4,242) |  | 522.91(498.54 to 548.36) |  | 13(3 to 34) |  | 3.90(2.07 to 7.00) |
|  | Polycystic ovarian syndrome |  | 406(174 to 752) |  | 119.56(108.13 to 132.09) |  | 3(1 to 8) |  | 0.88(0.18 to 3.06) |
| Zimbabwe | Endometriosis |  | 603(235 to 1,307) |  | 14.12(13.00 to 15.31) |  | 5(1 to 12) |  | 0.11(0.03 to 0.29) |
|  | Unexplained infertility |  | 17,913(6,747 to 39,980) |  | 431.61(425.27 to 438.04) |  | 133(36 to 353) |  | 3.21(2.69 to 3.82) |
|  | Polycystic ovarian syndrome |  | 2,790(1,061 to 5,511) |  | 63.27(60.92 to 65.70) |  | 21(6 to 52) |  | 0.47(0.29 to 0.74) |
| Benin | Endometriosis |  | 447(229 to 801) |  | 12.19(11.07 to 13.42) |  | 3(1 to 8) |  | 0.09(0.02 to 0.31) |
|  | Unexplained infertility |  | 7,491(3,515 to 12,819) |  | 207.03(202.28 to 211.87) |  | 56(18 to 131) |  | 1.55(1.16 to 2.04) |
|  | Polycystic ovarian syndrome |  | 2,316(1,175 to 4,122) |  | 60.31(57.84 to 62.88) |  | 17(6 to 40) |  | 0.45(0.26 to 0.76) |
| Burkina Faso | Endometriosis |  | 955(375 to 1,996) |  | 15.81(14.80 to 16.87) |  | 7(2 to 20) |  | 0.12(0.05 to 0.27) |
|  | Unexplained infertility |  | 23,401(7,092 to 55,572) |  | 394.85(389.72 to 400.05) |  | 175(35 to 481) |  | 2.94(2.52 to 3.44) |
|  | Polycystic ovarian syndrome |  | 3,608(1,324 to 7,218) |  | 56.86(54.99 to 58.79) |  | 27(8 to 69) |  | 0.42(0.27 to 0.64) |
| Cameroon | Endometriosis |  | 1,419(617 to 2,839) |  | 16.76(15.89 to 17.68) |  | 11(3 to 27) |  | 0.13(0.06 to 0.25) |
|  | Unexplained infertility |  | 126,296(46,882 to 275,367) |  | 1523.74(1515.23 to 1532.28) |  | 942(257 to 2,517) |  | 11.37(10.64 to 12.13) |
|  | Polycystic ovarian syndrome |  | 9,340(3,975 to 17,837) |  | 105.79(103.63 to 108.00) |  | 69(20 to 171) |  | 0.78(0.61 to 1.01) |
| Cabo Verde | Endometriosis |  | 13(5 to 29) |  | 8.55(4.60 to 14.83) |  | 0(0 to 0) |  | 0.06(0.00 to 3.17) |
|  | Unexplained infertility |  | 1,024(381 to 2,231) |  | 656.57(616.88 to 698.34) |  | 8(2 to 19) |  | 4.91(2.06 to 10.17) |
|  | Polycystic ovarian syndrome |  | 100(39 to 204) |  | 65.09(52.96 to 79.38) |  | 1(0 to 2) |  | 0.48(0.00 to 3.86) |
| Chad | Endometriosis |  | 737(267 to 1,631) |  | 17.36(16.10 to 18.72) |  | 5(1 to 15) |  | 0.13(0.04 to 0.34) |
|  | Unexplained infertility |  | 16,364(5,804 to 38,070) |  | 398.90(392.64 to 405.25) |  | 122(28 to 325) |  | 2.98(2.46 to 3.60) |
|  | Polycystic ovarian syndrome |  | 1,521(485 to 3,161) |  | 33.08(31.39 to 34.86) |  | 11(3 to 29) |  | 0.24(0.12 to 0.48) |
| Côte d'Ivoire | Endometriosis |  | 940(373 to 1,961) |  | 13.09(12.26 to 13.97) |  | 7(2 to 19) |  | 0.10(0.04 to 0.22) |
|  | Unexplained infertility |  | 54,884(21,147 to 117,125) |  | 781.50(774.93 to 788.13) |  | 410(110 to 1,089) |  | 5.83(5.28 to 6.44) |
|  | Polycystic ovarian syndrome |  | 4,490(1,688 to 9,025) |  | 60.94(59.16 to 62.78) |  | 33(9 to 83) |  | 0.45(0.31 to 0.65) |
| Gambia | Endometriosis |  | 91(33 to 187) |  | 13.28(10.65 to 16.54) |  | 1(0 to 2) |  | 0.10(0.00 to 1.16) |
|  | Unexplained infertility |  | 4,553(1,621 to 9,930) |  | 689.16(668.90 to 709.99) |  | 34(8 to 87) |  | 5.13(3.52 to 7.40) |
|  | Polycystic ovarian syndrome |  | 430(168 to 839) |  | 60.37(54.70 to 66.62) |  | 3(1 to 8) |  | 0.45(0.09 to 1.63) |
| Ghana | Endometriosis |  | 911(328 to 1,919) |  | 9.23(8.64 to 9.86) |  | 7(2 to 17) |  | 0.07(0.03 to 0.15) |
|  | Unexplained infertility |  | 48,784(15,554 to 110,042) |  | 504.44(499.95 to 508.97) |  | 363(86 to 926) |  | 3.75(3.38 to 4.17) |
|  | Polycystic ovarian syndrome |  | 5,573(2,048 to 11,106) |  | 56.23(54.76 to 57.74) |  | 41(11 to 111) |  | 0.42(0.30 to 0.57) |
| Guinea | Endometriosis |  | 433(225 to 772) |  | 12.12(10.99 to 13.36) |  | 3(1 to 8) |  | 0.09(0.02 to 0.31) |
|  | Unexplained infertility |  | 21,146(13,302 to 32,779) |  | 604.02(595.81 to 612.34) |  | 159(58 to 340) |  | 4.53(3.85 to 5.33) |
|  | Polycystic ovarian syndrome |  | 1,723(875 to 3,186) |  | 45.97(43.80 to 48.26) |  | 13(4 to 32) |  | 0.34(0.18 to 0.63) |
| Guinea-Bissau | Endometriosis |  | 70(28 to 149) |  | 12.27(9.55 to 15.71) |  | 1(0 to 1) |  | 0.09(0.00 to 1.29) |
|  | Unexplained infertility |  | 3,509(1,217 to 7,866) |  | 627.22(606.43 to 648.67) |  | 26(6 to 68) |  | 4.66(3.03 to 7.05) |
|  | Polycystic ovarian syndrome |  | 333(133 to 672) |  | 56.61(50.64 to 63.25) |  | 2(1 to 6) |  | 0.42(0.07 to 1.73) |
| Liberia | Endometriosis |  | 148(55 to 316) |  | 10.08(8.51 to 11.89) |  | 1(0 to 3) |  | 0.07(0.00 to 0.47) |
|  | Unexplained infertility |  | 11,223(4,242 to 23,367) |  | 782.72(768.19 to 797.48) |  | 83(22 to 211) |  | 5.80(4.61 to 7.23) |
|  | Polycystic ovarian syndrome |  | 841(302 to 1,736) |  | 54.66(50.99 to 58.56) |  | 6(2 to 17) |  | 0.40(0.15 to 0.93) |
| Mali | Endometriosis |  | 1,093(574 to 1,951) |  | 18.52(17.41 to 19.70) |  | 8(3 to 20) |  | 0.14(0.06 to 0.30) |
|  | Unexplained infertility |  | 26,927(14,408 to 45,477) |  | 421.52(416.41 to 426.69) |  | 203(72 to 456) |  | 3.17(2.74 to 3.67) |
|  | Polycystic ovarian syndrome |  | 3,192(1,607 to 5,918) |  | 50.07(48.29 to 51.92) |  | 24(8 to 55) |  | 0.37(0.23 to 0.59) |
| Mauritania | Endometriosis |  | 185(81 to 380) |  | 16.11(13.84 to 18.69) |  | 1(0 to 4) |  | 0.12(0.01 to 0.68) |
|  | Unexplained infertility |  | 12,501(4,532 to 27,568) |  | 1114.03(1094.29 to 1134.08) |  | 93(25 to 238) |  | 8.32(6.70 to 10.28) |
|  | Polycystic ovarian syndrome |  | 1,109(470 to 2,141) |  | 91.38(85.99 to 97.07) |  | 8(2 to 20) |  | 0.68(0.29 to 1.43) |
| Niger | Endometriosis |  | 1,556(613 to 3,189) |  | 26.84(25.48 to 28.27) |  | 12(3 to 32) |  | 0.20(0.10 to 0.39) |
|  | Unexplained infertility |  | 38,566(13,783 to 88,824) |  | 682.36(675.28 to 689.50) |  | 287(74 to 747) |  | 5.08(4.49 to 5.75) |
|  | Polycystic ovarian syndrome |  | 2,775(1,023 to 5,616) |  | 43.14(41.48 to 44.87) |  | 21(5 to 55) |  | 0.32(0.19 to 0.53) |
| Nigeria | Endometriosis |  | 10,762(4,398 to 21,933) |  | 17.36(17.02 to 17.70) |  | 81(25 to 209) |  | 0.13(0.10 to 0.16) |
|  | Unexplained infertility |  | 334,529(114,231 to 740,640) |  | 563.43(561.48 to 565.38) |  | 2,486(584 to 6,613) |  | 4.19(4.02 to 4.36) |
|  | Polycystic ovarian syndrome |  | 44,905(17,170 to 88,616) |  | 68.16(67.52 to 68.82) |  | 333(102 to 827) |  | 0.51(0.45 to 0.57) |
| Sao Tome and Principe | Endometriosis |  | 5(2 to 11) |  | 8.82(2.91 to 21.62) |  | 0(0 to 0) |  | 0.07(0.00 to 8.66) |
|  | Unexplained infertility |  | 151(38 to 382) |  | 259.95(219.92 to 305.85) |  | 1(0 to 3) |  | 1.94(0.07 to 11.70) |
|  | Polycystic ovarian syndrome |  | 35(12 to 68) |  | 56.29(38.97 to 79.62) |  | 0(0 to 1) |  | 0.41(0.00 to 9.23) |
| Senegal | Endometriosis |  | 651(343 to 1,117) |  | 15.46(14.28 to 16.72) |  | 5(2 to 13) |  | 0.12(0.04 to 0.31) |
|  | Unexplained infertility |  | 24,414(14,303 to 38,501) |  | 602.20(594.58 to 609.91) |  | 183(67 to 402) |  | 4.52(3.88 to 5.24) |
|  | Polycystic ovarian syndrome |  | 3,098(1,635 to 5,460) |  | 70.73(68.23 to 73.31) |  | 23(8 to 54) |  | 0.52(0.33 to 0.81) |
| Sierra Leone | Endometriosis |  | 260(91 to 586) |  | 10.17(8.95 to 11.54) |  | 2(0 to 6) |  | 0.08(0.01 to 0.38) |
|  | Unexplained infertility |  | 15,275(5,700 to 32,881) |  | 624.50(614.44 to 634.73) |  | 114(30 to 282) |  | 4.67(3.83 to 5.67) |
|  | Polycystic ovarian syndrome |  | 1,237(426 to 2,600) |  | 46.70(44.09 to 49.46) |  | 9(2 to 24) |  | 0.34(0.16 to 0.73) |
| Togo | Endometriosis |  | 306(122 to 651) |  | 13.62(12.13 to 15.25) |  | 2(1 to 6) |  | 0.10(0.01 to 0.38) |
|  | Unexplained infertility |  | 12,864(4,162 to 29,039) |  | 577.76(567.80 to 587.88) |  | 96(23 to 257) |  | 4.30(3.48 to 5.28) |
|  | Polycystic ovarian syndrome |  | 1,418(569 to 2,741) |  | 61.70(58.52 to 65.03) |  | 11(3 to 28) |  | 0.46(0.23 to 0.87) |
| American Samoa | Endometriosis |  | 2(1 to 5) |  | 21.74(3.24 to 74.29) |  | 0(0 to 0) |  | 0.16(0.00 to 37.67) |
|  | Unexplained infertility |  | 81(31 to 165) |  | 757.71(600.96 to 943.66) |  | 1(0 to 2) |  | 5.67(0.02 to 47.98) |
|  | Polycystic ovarian syndrome |  | 29(13 to 55) |  | 266.58(178.25 to 384.57) |  | 0(0 to 1) |  | 1.97(0.00 to 41.14) |
| Bermuda | Endometriosis |  | 1(1 to 3) |  | 12.15(0.72 to 61.02) |  | 0(0 to 0) |  | 0.09(0.00 to 41.03) |
|  | Unexplained infertility |  | 157(67 to 315) |  | 1238.49(1047.62 to 1457.39) |  | 1(0 to 3) |  | 9.26(0.32 to 55.92) |
|  | Polycystic ovarian syndrome |  | 27(12 to 52) |  | 234.88(152.97 to 347.79) |  | 0(0 to 0) |  | 1.74(0.00 to 44.01) |
| Cook Islands | Endometriosis |  | 1(0 to 2) |  | 18.43(0.18 to 130.56) |  | 0(0 to 0) |  | 0.14(0.00 to 97.01) |
|  | Unexplained infertility |  | 31(11 to 64) |  | 742.76(503.15 to 1059.57) |  | 0(0 to 1) |  | 5.56(0.00 to 107.49) |
|  | Polycystic ovarian syndrome |  | 12(5 to 22) |  | 280.66(143.68 to 497.20) |  | 0(0 to 0) |  | 2.07(0.00 to 100.77) |
| Greenland | Endometriosis |  | 2(1 to 4) |  | 16.04(2.13 to 60.20) |  | 0(0 to 0) |  | 0.12(0.00 to 34.24) |
|  | Unexplained infertility |  | 50(5 to 149) |  | 369.54(274.36 to 489.54) |  | 0(0 to 1) |  | 2.76(0.00 to 38.83) |
|  | Polycystic ovarian syndrome |  | 31(14 to 57) |  | 231.80(156.73 to 332.56) |  | 0(0 to 1) |  | 1.72(0.00 to 37.11) |
| Guam | Endometriosis |  | 8(3 to 16) |  | 21.85(9.25 to 43.91) |  | 0(0 to 0) |  | 0.16(0.00 to 11.43) |
|  | Unexplained infertility |  | 255(87 to 545) |  | 729.19(642.42 to 824.58) |  | 2(0 to 5) |  | 5.43(0.61 to 20.57) |
|  | Polycystic ovarian syndrome |  | 103(44 to 191) |  | 293.85(239.91 to 356.50) |  | 1(0 to 2) |  | 2.18(0.02 to 15.16) |
| Monaco | Endometriosis |  | 1(1 to 2) |  | 17.61(0.59 to 101.17) |  | 0(0 to 0) |  | 0.13(0.00 to 71.22) |
|  | Unexplained infertility |  | 42(10 to 101) |  | 581.58(415.11 to 798.96) |  | 0(0 to 1) |  | 4.38(0.00 to 78.39) |
|  | Polycystic ovarian syndrome |  | 33(16 to 58) |  | 505.99(345.11 to 720.49) |  | 0(0 to 1) |  | 3.78(0.00 to 77.80) |
| Nauru | Endometriosis |  | 1(0 to 1) |  | 24.36(0.16 to 207.25) |  | 0(0 to 0) |  | 0.18(0.00 to 166.83) |
|  | Unexplained infertility |  | 22(8 to 47) |  | 790.41(497.25 to 1207.78) |  | 0(0 to 0) |  | 5.88(0.00 to 176.76) |
|  | Polycystic ovarian syndrome |  | 7(3 to 13) |  | 237.90(95.60 to 511.75) |  | 0(0 to 0) |  | 1.77(0.00 to 169.54) |
| Niue | Endometriosis |  | 0(0 to 0) |  | 18.83(0.00 to 1099.65) |  | 0(0 to 0) |  | 0.14(0.00 to 1062.08) |
|  | Unexplained infertility |  | 3(1 to 6) |  | 763.73(145.30 to 2344.38) |  | 0(0 to 0) |  | 5.68(0.00 to 1073.27) |
|  | Polycystic ovarian syndrome |  | 1(0 to 2) |  | 265.38(6.04 to 1551.05) |  | 0(0 to 0) |  | 1.98(0.00 to 1065.80) |
| Northern Mariana Islands | Endometriosis |  | 2(1 to 4) |  | 18.06(1.84 to 72.69) |  | 0(0 to 0) |  | 0.13(0.00 to 42.23) |
|  | Unexplained infertility |  | 77(30 to 158) |  | 771.53(608.35 to 966.64) |  | 1(0 to 1) |  | 5.76(0.01 to 52.49) |
|  | Polycystic ovarian syndrome |  | 26(12 to 48) |  | 258.03(168.46 to 380.31) |  | 0(0 to 0) |  | 1.91(0.00 to 45.55) |
| Palau | Endometriosis |  | 1(0 to 1) |  | 17.35(0.03 to 168.60) |  | 0(0 to 0) |  | 0.13(0.00 to 137.44) |
|  | Unexplained infertility |  | 25(10 to 50) |  | 760.70(488.56 to 1138.22) |  | 0(0 to 0) |  | 5.67(0.00 to 147.54) |
|  | Polycystic ovarian syndrome |  | 9(4 to 16) |  | 265.05(115.92 to 526.74) |  | 0(0 to 0) |  | 1.97(0.00 to 140.90) |
| Puerto Rico | Endometriosis |  | 82(38 to 159) |  | 11.39(9.04 to 14.19) |  | 1(0 to 2) |  | 0.08(0.00 to 0.76) |
|  | Unexplained infertility |  | 8,804(3,626 to 17,764) |  | 1212.93(1187.61 to 1238.68) |  | 66(19 to 170) |  | 9.07(7.00 to 11.58) |
|  | Polycystic ovarian syndrome |  | 1,852(842 to 3,495) |  | 263.66(251.70 to 276.05) |  | 14(5 to 34) |  | 1.96(1.06 to 3.34) |
| Saint Kitts and Nevis | Endometriosis |  | 2(1 to 3) |  | 12.09(1.25 to 49.46) |  | 0(0 to 0) |  | 0.09(0.00 to 29.51) |
|  | Unexplained infertility |  | 198(81 to 407) |  | 1275.05(1103.07 to 1467.94) |  | 1(0 to 4) |  | 9.50(0.65 to 45.18) |
|  | Polycystic ovarian syndrome |  | 33(15 to 62) |  | 220.64(151.11 to 312.70) |  | 0(0 to 1) |  | 1.64(0.00 to 32.36) |
| San Marino | Endometriosis |  | 1(1 to 2) |  | 17.36(0.53 to 99.74) |  | 0(0 to 0) |  | 0.13(0.00 to 69.63) |
|  | Unexplained infertility |  | 41(10 to 102) |  | 593.66(422.28 to 816.30) |  | 0(0 to 1) |  | 4.45(0.00 to 77.21) |
|  | Polycystic ovarian syndrome |  | 31(15 to 55) |  | 493.61(333.92 to 706.74) |  | 0(0 to 1) |  | 3.68(0.00 to 76.19) |
| Tokelau | Endometriosis |  | 0(0 to 0) |  | 26.93(0.00 to 1314.18) |  | 0(0 to 0) |  | 0.20(0.00 to 1261.13) |
|  | Unexplained infertility |  | 2(1 to 5) |  | 769.39(120.18 to 2570.72) |  | 0(0 to 0) |  | 5.74(0.00 to 1272.26) |
|  | Polycystic ovarian syndrome |  | 1(0 to 1) |  | 244.97(2.38 to 1713.98) |  | 0(0 to 0) |  | 1.83(0.00 to 1264.37) |
| Tuvalu | Endometriosis |  | 1(0 to 1) |  | 20.09(0.05 to 183.83) |  | 0(0 to 0) |  | 0.15(0.00 to 148.39) |
|  | Unexplained infertility |  | 23(9 to 48) |  | 796.33(503.18 to 1205.40) |  | 0(0 to 0) |  | 5.94(0.00 to 159.22) |
|  | Polycystic ovarian syndrome |  | 7(3 to 13) |  | 224.23(87.93 to 481.79) |  | 0(0 to 0) |  | 1.66(0.00 to 151.16) |
| United States Virgin Islands | Endometriosis |  | 2(1 to 4) |  | 13.49(1.71 to 50.50) |  | 0(0 to 0) |  | 0.10(0.00 to 28.17) |
|  | Unexplained infertility |  | 197(83 to 392) |  | 1223.00(1056.48 to 1409.77) |  | 1(0 to 4) |  | 9.17(0.61 to 43.47) |
|  | Polycystic ovarian syndrome |  | 41(19 to 75) |  | 261.13(186.13 to 357.60) |  | 0(0 to 1) |  | 1.94(0.00 to 31.56) |
| South Sudan | Endometriosis |  | 417(188 to 822) |  | 18.15(16.42 to 20.04) |  | 3(1 to 9) |  | 0.14(0.03 to 0.42) |
|  | Unexplained infertility |  | 19,451(8,379 to 39,620) |  | 860.78(848.50 to 873.20) |  | 145(40 to 359) |  | 6.43(5.41 to 7.59) |
|  | Polycystic ovarian syndrome |  | 1,470(607 to 2,882) |  | 58.17(55.15 to 61.33) |  | 11(3 to 28) |  | 0.43(0.21 to 0.82) |
| Sudan | Endometriosis |  | 2,432(1,037 to 5,021) |  | 20.67(19.85 to 21.52) |  | 18(5 to 43) |  | 0.15(0.09 to 0.25) |
|  | Unexplained infertility |  | 130,217(52,979 to 270,584) |  | 1118.24(1112.13 to 1124.38) |  | 973(285 to 2,436) |  | 8.35(7.83 to 8.90) |
|  | Polycystic ovarian syndrome |  | 19,637(7,654 to 38,973) |  | 162.73(160.44 to 165.05) |  | 145(43 to 366) |  | 1.20(1.01 to 1.42) |

**Table S7:** Prevalence and YLDs cases and age-standardised rate of secondary infertility attributable to endometriosis, PCOS and unexplained infertility for WCBA in 2021 by location.

| **location** | **Disease** |  | **Prevalence in 2021** | | |  | **YLDs (Years Lived with Disability) in 2021** | | |
| --- | --- | --- | --- | --- | --- | --- | --- | --- | --- |
|  |  |  | **Number of cases** |  | **ASR per 100,000 population** |  | **Number of cases** |  | **ASR per 100,000 population** |
| **Global** |  |  |  |  |  |  |  |  |  |
|  | Endometriosis |  | 873,543(504,747 to 1,445,036) |  | 44.41(44.31 to 44.50) |  | 4,479(1,471 to 11,124) |  | 0.23(0.22 to 0.23) |
|  | Unexplained infertility |  | 89,014,732(43,511,904 to 165,111,254) |  | 4498.61(4497.68 to 4499.55) |  | 457,227(141,175 to 1,202,203) |  | 23.11(23.04 to 23.18) |
|  | Polycystic ovarian syndrome |  | 9,012,694(5,577,809 to 13,911,090) |  | 458.30(458.00 to 458.60) |  | 45,887(15,907 to 111,166) |  | 2.33(2.31 to 2.36) |
| **GBD regions** |  |  |  |  |  |  |  |  |  |
| East Asia | Endometriosis |  | 138,816(83,194 to 223,788) |  | 38.73(38.52 to 38.94) |  | 711(240 to 1,809) |  | 0.20(0.18 to 0.21) |
|  | Unexplained infertility |  | 27,867,037(13,438,499 to 50,871,418) |  | 7597.72(7594.86 to 7600.58) |  | 143,199(44,318 to 386,101) |  | 39.06(38.85 to 39.26) |
|  | Polycystic ovarian syndrome |  | 1,726,394(1,077,580 to 2,724,958) |  | 506.10(505.32 to 506.88) |  | 8,781(3,100 to 21,486) |  | 2.58(2.52 to 2.63) |
| Southeast Asia | Endometriosis |  | 94,162(53,740 to 152,729) |  | 50.41(50.09 to 50.74) |  | 484(157 to 1,204) |  | 0.26(0.24 to 0.28) |
|  | Unexplained infertility |  | 8,763,423(4,074,079 to 16,981,866) |  | 4682.13(4679.03 to 4685.23) |  | 45,117(12,574 to 120,691) |  | 24.10(23.88 to 24.33) |
|  | Polycystic ovarian syndrome |  | 1,364,407(819,651 to 2,151,707) |  | 732.45(731.22 to 733.68) |  | 6,950(2,377 to 17,111) |  | 3.73(3.64 to 3.82) |
| Oceania | Endometriosis |  | 2,778(1,648 to 4,346) |  | 80.21(77.24 to 83.26) |  | 14(5 to 35) |  | 0.41(0.23 to 0.70) |
|  | Unexplained infertility |  | 58,628(32,079 to 92,243) |  | 1695.35(1681.61 to 1709.17) |  | 301(95 to 737) |  | 8.71(7.75 to 9.76) |
|  | Polycystic ovarian syndrome |  | 17,046(10,854 to 26,263) |  | 488.01(480.68 to 495.42) |  | 87(32 to 204) |  | 2.48(1.98 to 3.07) |
| Central Asia | Endometriosis |  | 11,260(6,502 to 18,692) |  | 44.55(43.73 to 45.39) |  | 58(19 to 138) |  | 0.23(0.17 to 0.30) |
|  | Unexplained infertility |  | 652,473(252,639 to 1,399,188) |  | 2519.52(2513.39 to 2525.66) |  | 3,354(916 to 8,760) |  | 12.96(12.52 to 13.41) |
|  | Polycystic ovarian syndrome |  | 33,898(20,098 to 54,878) |  | 136.28(134.83 to 137.75) |  | 172(57 to 422) |  | 0.69(0.59 to 0.81) |
| Central Europe | Endometriosis |  | 10,789(6,339 to 17,951) |  | 41.69(40.89 to 42.51) |  | 55(18 to 135) |  | 0.21(0.16 to 0.28) |
|  | Unexplained infertility |  | 1,160,443(496,295 to 2,284,588) |  | 4353.22(4345.16 to 4361.28) |  | 5,955(1,717 to 16,152) |  | 22.33(21.76 to 22.92) |
|  | Polycystic ovarian syndrome |  | 17,418(10,770 to 27,614) |  | 67.40(66.37 to 68.44) |  | 88(30 to 219) |  | 0.34(0.27 to 0.43) |
| Eastern Europe | Endometriosis |  | 34,672(19,825 to 57,272) |  | 68.04(67.30 to 68.79) |  | 178(58 to 425) |  | 0.35(0.30 to 0.41) |
|  | Unexplained infertility |  | 2,897,702(1,318,683 to 5,977,026) |  | 5556.98(5550.36 to 5563.60) |  | 14,905(4,469 to 42,715) |  | 28.59(28.12 to 29.07) |
|  | Polycystic ovarian syndrome |  | 36,601(21,167 to 60,246) |  | 71.03(70.28 to 71.80) |  | 186(62 to 453) |  | 0.36(0.31 to 0.42) |
| High-income Asia Pacific | Endometriosis |  | 15,969(8,564 to 26,383) |  | 37.55(36.95 to 38.15) |  | 82(24 to 202) |  | 0.19(0.15 to 0.24) |
|  | Unexplained infertility |  | 514,266(40,244 to 1,585,306) |  | 1118.21(1115.12 to 1121.31) |  | 2,713(137 to 10,878) |  | 5.89(5.67 to 6.12) |
|  | Polycystic ovarian syndrome |  | 444,727(278,560 to 668,205) |  | 1069.76(1066.53 to 1072.99) |  | 2,264(803 to 5,015) |  | 5.45(5.22 to 5.68) |
| Australasia | Endometriosis |  | 1,762(807 to 3,295) |  | 23.17(22.09 to 24.29) |  | 9(2 to 24) |  | 0.12(0.05 to 0.24) |
|  | Unexplained infertility |  | 19,018(4,047 to 88,405) |  | 240.72(237.30 to 244.18) |  | 102(12 to 507) |  | 1.29(1.05 to 1.58) |
|  | Polycystic ovarian syndrome |  | 56,166(30,061 to 95,052) |  | 732.80(726.72 to 738.93) |  | 287(91 to 702) |  | 3.75(3.33 to 4.22) |
| Western Europe | Endometriosis |  | 26,378(14,620 to 43,180) |  | 26.32(26.00 to 26.65) |  | 135(43 to 338) |  | 0.13(0.11 to 0.16) |
|  | Unexplained infertility |  | 1,833,889(477,298 to 4,354,952) |  | 1764.01(1761.44 to 1766.58) |  | 9,546(1,850 to 29,223) |  | 9.17(8.99 to 9.36) |
|  | Polycystic ovarian syndrome |  | 771,424(477,035 to 1,208,106) |  | 761.87(760.15 to 763.59) |  | 3,941(1,337 to 9,429) |  | 3.89(3.77 to 4.02) |
| Southern Latin America | Endometriosis |  | 5,465(3,178 to 8,761) |  | 30.11(29.32 to 30.92) |  | 28(9 to 72) |  | 0.15(0.10 to 0.22) |
|  | Unexplained infertility |  | 298,789(57,785 to 751,141) |  | 1641.26(1635.38 to 1647.16) |  | 1,556(247 to 4,866) |  | 8.55(8.13 to 8.98) |
|  | Polycystic ovarian syndrome |  | 77,169(44,594 to 122,127) |  | 429.64(426.61 to 432.69) |  | 393(129 to 975) |  | 2.19(1.98 to 2.42) |
| High-income North America | Endometriosis |  | 17,771(9,905 to 29,061) |  | 20.27(19.97 to 20.57) |  | 91(29 to 223) |  | 0.10(0.08 to 0.13) |
|  | Unexplained infertility |  | 1,004,531(120,618 to 2,774,628) |  | 1148.08(1145.83 to 1150.33) |  | 5,216(531 to 17,935) |  | 5.96(5.80 to 6.12) |
|  | Polycystic ovarian syndrome |  | 648,932(387,951 to 1,020,896) |  | 749.28(747.45 to 751.11) |  | 3,306(1,150 to 7,560) |  | 3.82(3.69 to 3.95) |
| Caribbean | Endometriosis |  | 4,254(2,460 to 7,111) |  | 35.15(34.11 to 36.23) |  | 22(7 to 54) |  | 0.18(0.11 to 0.27) |
|  | Unexplained infertility |  | 468,082(252,624 to 874,751) |  | 3859.05(3848.00 to 3870.12) |  | 2,401(751 to 5,996) |  | 19.80(19.02 to 20.61) |
|  | Polycystic ovarian syndrome |  | 47,030(28,255 to 75,976) |  | 390.11(386.59 to 393.65) |  | 239(82 to 578) |  | 1.98(1.74 to 2.25) |
| Andean Latin America | Endometriosis |  | 5,528(2,880 to 9,225) |  | 30.95(30.14 to 31.77) |  | 28(8 to 70) |  | 0.16(0.11 to 0.23) |
|  | Unexplained infertility |  | 160,479(14,713 to 529,544) |  | 905.90(901.47 to 910.35) |  | 838(64 to 3,141) |  | 4.73(4.41 to 5.06) |
|  | Polycystic ovarian syndrome |  | 142,130(85,529 to 223,836) |  | 799.28(795.12 to 803.45) |  | 724(252 to 1,727) |  | 4.07(3.78 to 4.38) |
| Central Latin America | Endometriosis |  | 22,144(12,547 to 36,516) |  | 32.29(31.87 to 32.72) |  | 113(36 to 276) |  | 0.17(0.14 to 0.20) |
|  | Unexplained infertility |  | 1,960,948(770,194 to 4,166,807) |  | 2856.20(2852.20 to 2860.20) |  | 10,076(2,633 to 27,273) |  | 14.67(14.39 to 14.96) |
|  | Polycystic ovarian syndrome |  | 533,543(326,889 to 831,808) |  | 780.13(778.04 to 782.23) |  | 2,715(940 to 6,633) |  | 3.97(3.82 to 4.12) |
| Tropical Latin America | Endometriosis |  | 21,913(12,027 to 36,474) |  | 35.02(34.55 to 35.49) |  | 113(36 to 281) |  | 0.18(0.15 to 0.22) |
|  | Unexplained infertility |  | 1,722,797(624,019 to 3,716,464) |  | 2632.85(2628.91 to 2636.79) |  | 8,858(2,318 to 24,492) |  | 13.53(13.25 to 13.81) |
|  | Polycystic ovarian syndrome |  | 91,507(52,196 to 151,906) |  | 146.70(145.74 to 147.66) |  | 467(158 to 1,189) |  | 0.75(0.68 to 0.82) |
| North Africa and Middle East | Endometriosis |  | 82,672(46,366 to 140,065) |  | 51.19(50.84 to 51.54) |  | 424(133 to 1,045) |  | 0.26(0.24 to 0.29) |
|  | Unexplained infertility |  | 4,555,587(2,064,411 to 8,414,525) |  | 2772.34(2769.79 to 2774.89) |  | 23,506(6,506 to 59,536) |  | 14.30(14.12 to 14.49) |
|  | Polycystic ovarian syndrome |  | 863,318(511,107 to 1,387,352) |  | 535.89(534.75 to 537.02) |  | 4,401(1,470 to 10,854) |  | 2.73(2.65 to 2.81) |
| South Asia | Endometriosis |  | 226,549(127,053 to 376,492) |  | 45.65(45.56 to 45.75) |  | 1,161(374 to 2,972) |  | 0.23(0.23 to 0.24) |
|  | Unexplained infertility |  | 26,008,800(13,238,371 to 47,482,723) |  | 5229.56(5228.55 to 5230.56) |  | 133,097(40,404 to 322,617) |  | 26.76(26.69 to 26.83) |
|  | Polycystic ovarian syndrome |  | 1,539,646(912,883 to 2,464,849) |  | 310.47(310.23 to 310.72) |  | 7,828(2,657 to 19,255) |  | 1.58(1.56 to 1.60) |
| Central Sub-Saharan Africa | Endometriosis |  | 17,817(10,132 to 29,884) |  | 58.03(57.17 to 58.90) |  | 92(29 to 225) |  | 0.30(0.24 to 0.37) |
|  | Unexplained infertility |  | 1,413,915(662,156 to 2,692,943) |  | 4900.70(4892.54 to 4908.87) |  | 7,250(2,090 to 19,082) |  | 25.13(24.55 to 25.72) |
|  | Polycystic ovarian syndrome |  | 65,727(37,942 to 107,018) |  | 207.53(205.91 to 209.15) |  | 334(108 to 828) |  | 1.05(0.94 to 1.18) |
| Eastern Sub-Saharan Africa | Endometriosis |  | 50,842(29,895 to 83,046) |  | 49.94(49.50 to 50.38) |  | 261(86 to 633) |  | 0.26(0.23 to 0.29) |
|  | Unexplained infertility |  | 3,211,607(1,741,545 to 5,569,923) |  | 3298.24(3294.58 to 3301.90) |  | 16,454(5,364 to 39,734) |  | 16.90(16.64 to 17.16) |
|  | Polycystic ovarian syndrome |  | 215,182(129,458 to 341,131) |  | 204.13(203.24 to 205.01) |  | 1,094(376 to 2,620) |  | 1.04(0.98 to 1.10) |
| Southern Sub-Saharan Africa | Endometriosis |  | 10,471(6,004 to 17,398) |  | 47.04(46.14 to 47.96) |  | 54(17 to 135) |  | 0.24(0.18 to 0.32) |
|  | Unexplained infertility |  | 643,093(185,145 to 1,538,474) |  | 2844.44(2837.48 to 2851.41) |  | 3,307(723 to 9,746) |  | 14.63(14.14 to 15.14) |
|  | Polycystic ovarian syndrome |  | 64,527(38,063 to 104,291) |  | 291.01(288.77 to 293.27) |  | 328(109 to 799) |  | 1.48(1.32 to 1.65) |
| Western Sub-Saharan Africa | Endometriosis |  | 71,532(41,799 to 116,528) |  | 63.04(62.57 to 63.51) |  | 366(116 to 889) |  | 0.32(0.29 to 0.36) |
|  | Unexplained infertility |  | 3,799,224(1,572,005 to 7,878,576) |  | 3533.82(3530.21 to 3537.43) |  | 19,474(5,194 to 48,899) |  | 18.11(17.86 to 18.37) |
|  | Polycystic ovarian syndrome |  | 255,902(149,816 to 411,424) |  | 219.51(218.64 to 220.38) |  | 1,301(427 to 3,152) |  | 1.12(1.05 to 1.18) |
| **countries and territories** |  |  |  |  |  |  |  |  |  |
| China | Endometriosis |  | 129,109(77,066 to 208,230) |  | 37.37(37.16 to 37.58) |  | 661(223 to 1,677) |  | 0.19(0.18 to 0.21) |
|  | Unexplained infertility |  | 27,145,921(13,133,928 to 49,572,551) |  | 7673.68(7670.75 to 7676.61) |  | 139,498(43,183 to 377,028) |  | 39.45(39.24 to 39.66) |
|  | Polycystic ovarian syndrome |  | 1,660,339(1,034,973 to 2,622,582) |  | 505.32(504.53 to 506.12) |  | 8,445(2,980 to 20,662) |  | 2.57(2.52 to 2.63) |
| Democratic People's Republic of Korea | Endometriosis |  | 4,239(2,504 to 6,808) |  | 62.45(60.58 to 64.37) |  | 22(7 to 55) |  | 0.32(0.20 to 0.49) |
|  | Unexplained infertility |  | 376,898(179,418 to 732,485) |  | 5619.33(5601.40 to 5637.32) |  | 1,932(574 to 4,667) |  | 28.80(27.53 to 30.12) |
|  | Polycystic ovarian syndrome |  | 20,984(12,533 to 33,317) |  | 316.07(311.79 to 320.40) |  | 106(37 to 260) |  | 1.60(1.31 to 1.95) |
| Cambodia | Endometriosis |  | 2,684(1,554 to 4,370) |  | 58.38(56.19 to 60.65) |  | 14(5 to 35) |  | 0.30(0.16 to 0.51) |
|  | Unexplained infertility |  | 193,758(85,866 to 387,173) |  | 4188.23(4169.55 to 4206.97) |  | 995(272 to 2,600) |  | 21.51(20.19 to 22.90) |
|  | Polycystic ovarian syndrome |  | 26,711(16,085 to 42,151) |  | 581.29(574.31 to 588.33) |  | 136(46 to 331) |  | 2.96(2.48 to 3.51) |
| Indonesia | Endometriosis |  | 42,385(23,793 to 70,524) |  | 54.93(54.41 to 55.46) |  | 218(72 to 554) |  | 0.28(0.25 to 0.32) |
|  | Unexplained infertility |  | 5,147,331(2,462,049 to 9,685,354) |  | 6664.79(6659.03 to 6670.55) |  | 26,470(7,832 to 69,891) |  | 34.27(33.85 to 34.68) |
|  | Polycystic ovarian syndrome |  | 600,208(358,431 to 973,417) |  | 780.56(778.58 to 782.54) |  | 3,056(1,051 to 7,432) |  | 3.97(3.83 to 4.12) |
| Lao People's Democratic Republic | Endometriosis |  | 1,083(599 to 1,816) |  | 54.75(51.52 to 58.13) |  | 6(2 to 14) |  | 0.28(0.10 to 0.65) |
|  | Unexplained infertility |  | 63,687(25,271 to 132,830) |  | 3263.42(3238.03 to 3288.96) |  | 327(85 to 872) |  | 16.78(15.00 to 18.72) |
|  | Polycystic ovarian syndrome |  | 12,538(7,322 to 20,211) |  | 632.28(621.21 to 643.51) |  | 64(22 to 161) |  | 3.22(2.48 to 4.13) |
| Malaysia | Endometriosis |  | 4,756(2,729 to 7,641) |  | 54.93(53.38 to 56.52) |  | 24(8 to 59) |  | 0.28(0.18 to 0.42) |
|  | Unexplained infertility |  | 363,333(134,149 to 772,038) |  | 4200.89(4187.22 to 4214.59) |  | 1,867(484 to 4,848) |  | 21.59(20.62 to 22.60) |
|  | Polycystic ovarian syndrome |  | 100,054(61,605 to 153,486) |  | 1156.40(1149.23 to 1163.60) |  | 509(178 to 1,223) |  | 5.88(5.38 to 6.42) |
| Maldives | Endometriosis |  | 60(36 to 96) |  | 47.29(36.00 to 61.52) |  | 0(0 to 1) |  | 0.24(0.00 to 4.78) |
|  | Unexplained infertility |  | 6,709(5,253 to 8,443) |  | 5015.59(4895.43 to 5138.40) |  | 34(13 to 78) |  | 25.65(17.75 to 36.51) |
|  | Polycystic ovarian syndrome |  | 979(611 to 1,550) |  | 771.09(722.93 to 822.01) |  | 5(2 to 12) |  | 3.89(1.23 to 10.05) |
| Myanmar | Endometriosis |  | 6,872(4,121 to 11,089) |  | 46.12(45.03 to 47.22) |  | 36(11 to 86) |  | 0.24(0.17 to 0.33) |
|  | Unexplained infertility |  | 229,704(155,488 to 312,449) |  | 1564.05(1557.66 to 1570.46) |  | 1,177(434 to 2,872) |  | 8.01(7.56 to 8.48) |
|  | Polycystic ovarian syndrome |  | 89,596(56,948 to 137,078) |  | 600.90(596.97 to 604.85) |  | 454(161 to 1,043) |  | 3.05(2.77 to 3.34) |
| Philippines | Endometriosis |  | 14,983(7,212 to 25,217) |  | 51.68(50.86 to 52.52) |  | 77(22 to 199) |  | 0.27(0.21 to 0.33) |
|  | Unexplained infertility |  | 1,739,753(487,120 to 3,904,560) |  | 6062.21(6053.19 to 6071.25) |  | 8,995(1,644 to 26,396) |  | 31.35(30.70 to 32.00) |
|  | Polycystic ovarian syndrome |  | 189,692(97,853 to 328,252) |  | 651.86(648.92 to 654.81) |  | 969(298 to 2,423) |  | 3.33(3.12 to 3.55) |
| Sri Lanka | Endometriosis |  | 2,446(1,409 to 3,968) |  | 42.85(41.17 to 44.60) |  | 12(4 to 31) |  | 0.22(0.11 to 0.38) |
|  | Unexplained infertility |  | 153,108(51,811 to 322,049) |  | 2629.88(2616.68 to 2643.14) |  | 790(189 to 2,190) |  | 13.57(12.64 to 14.55) |
|  | Polycystic ovarian syndrome |  | 45,834(27,705 to 72,126) |  | 804.84(797.46 to 812.27) |  | 234(80 to 576) |  | 4.11(3.60 to 4.67) |
| Thailand | Endometriosis |  | 6,393(3,621 to 10,535) |  | 37.85(36.91 to 38.81) |  | 33(10 to 83) |  | 0.20(0.13 to 0.28) |
|  | Unexplained infertility |  | 451,100(148,396 to 963,976) |  | 2633.66(2625.88 to 2641.47) |  | 2,324(568 to 6,438) |  | 13.55(13.00 to 14.12) |
|  | Polycystic ovarian syndrome |  | 136,182(81,571 to 218,515) |  | 810.61(806.23 to 815.01) |  | 694(236 to 1,652) |  | 4.13(3.82 to 4.46) |
| Timor-Leste | Endometriosis |  | 212(117 to 354) |  | 68.61(59.46 to 78.84) |  | 1(0 to 3) |  | 0.35(0.01 to 2.12) |
|  | Unexplained infertility |  | 6,835(2,782 to 13,683) |  | 2403.30(2345.71 to 2461.99) |  | 35(9 to 90) |  | 12.38(8.58 to 17.36) |
|  | Polycystic ovarian syndrome |  | 1,619(922 to 2,583) |  | 515.51(490.04 to 542.05) |  | 8(3 to 20) |  | 2.62(1.12 to 5.35) |
| Viet Nam | Endometriosis |  | 12,005(6,726 to 18,918) |  | 44.17(43.38 to 44.98) |  | 62(18 to 157) |  | 0.23(0.17 to 0.29) |
|  | Unexplained infertility |  | 384,451(89,661 to 926,089) |  | 1355.36(1351.07 to 1359.66) |  | 1,981(342 to 5,971) |  | 6.99(6.68 to 7.30) |
|  | Polycystic ovarian syndrome |  | 155,699(93,964 to 237,264) |  | 575.95(573.06 to 578.84) |  | 794(268 to 1,929) |  | 2.94(2.73 to 3.15) |
| Fiji | Endometriosis |  | 139(79 to 221) |  | 60.52(50.88 to 71.48) |  | 1(0 to 2) |  | 0.31(0.00 to 2.26) |
|  | Unexplained infertility |  | 11,854(5,682 to 22,188) |  | 5109.27(5017.68 to 5202.14) |  | 61(17 to 151) |  | 26.25(20.08 to 33.76) |
|  | Polycystic ovarian syndrome |  | 1,495(889 to 2,446) |  | 651.52(618.90 to 685.43) |  | 8(3 to 19) |  | 3.31(1.39 to 6.67) |
| Kiribati | Endometriosis |  | 24(14 to 40) |  | 76.27(48.90 to 114.51) |  | 0(0 to 0) |  | 0.39(0.00 to 15.11) |
|  | Unexplained infertility |  | 1,120(477 to 2,242) |  | 3556.42(3350.87 to 3772.04) |  | 6(2 to 16) |  | 18.26(6.51 to 41.85) |
|  | Polycystic ovarian syndrome |  | 176(103 to 281) |  | 550.82(472.32 to 639.55) |  | 1(0 to 2) |  | 2.81(0.05 to 19.18) |
| Marshall Islands | Endometriosis |  | 9(5 to 15) |  | 62.54(28.72 to 119.42) |  | 0(0 to 0) |  | 0.32(0.00 to 28.22) |
|  | Unexplained infertility |  | 513(227 to 1,027) |  | 3515.48(3217.66 to 3834.12) |  | 3(1 to 7) |  | 18.07(3.21 to 57.77) |
|  | Polycystic ovarian syndrome |  | 79(46 to 127) |  | 537.78(425.65 to 671.21) |  | 0(0 to 1) |  | 2.73(0.00 to 32.74) |
| Micronesia (Federated States of) | Endometriosis |  | 16(9 to 26) |  | 63.51(35.99 to 104.36) |  | 0(0 to 0) |  | 0.33(0.00 to 17.00) |
|  | Unexplained infertility |  | 835(379 to 1,678) |  | 3495.58(3261.79 to 3741.93) |  | 4(1 to 11) |  | 17.99(5.18 to 45.23) |
|  | Polycystic ovarian syndrome |  | 149(86 to 238) |  | 588.98(497.62 to 692.75) |  | 1(0 to 2) |  | 3.01(0.03 to 21.83) |
| Papua New Guinea | Endometriosis |  | 2,180(1,289 to 3,435) |  | 82.87(79.42 to 86.45) |  | 11(4 to 28) |  | 0.42(0.21 to 0.77) |
|  | Unexplained infertility |  | 28,910(14,779 to 44,471) |  | 1069.35(1057.02 to 1081.80) |  | 148(43 to 374) |  | 5.49(4.64 to 6.46) |
|  | Polycystic ovarian syndrome |  | 12,070(7,650 to 18,632) |  | 454.43(446.33 to 462.65) |  | 61(23 to 141) |  | 2.31(1.76 to 2.98) |
| Samoa | Endometriosis |  | 29(17 to 48) |  | 64.05(42.89 to 92.45) |  | 0(0 to 0) |  | 0.33(0.00 to 9.95) |
|  | Unexplained infertility |  | 1,572(664 to 3,193) |  | 3562.29(3387.46 to 3744.07) |  | 8(2 to 21) |  | 18.35(7.93 to 36.57) |
|  | Polycystic ovarian syndrome |  | 311(181 to 505) |  | 662.21(590.08 to 741.12) |  | 2(1 to 4) |  | 3.37(0.26 to 15.05) |
| Solomon Islands | Endometriosis |  | 142(80 to 238) |  | 86.59(72.91 to 102.19) |  | 1(0 to 2) |  | 0.45(0.00 to 3.36) |
|  | Unexplained infertility |  | 5,476(2,400 to 11,088) |  | 3393.86(3304.39 to 3485.22) |  | 28(7 to 73) |  | 17.48(11.62 to 25.34) |
|  | Polycystic ovarian syndrome |  | 833(486 to 1,327) |  | 496.25(462.98 to 531.38) |  | 4(1 to 10) |  | 2.53(0.72 to 6.53) |
| Tonga | Endometriosis |  | 17(10 to 28) |  | 72.74(42.67 to 116.17) |  | 0(0 to 0) |  | 0.38(0.00 to 16.96) |
|  | Unexplained infertility |  | 824(325 to 1,669) |  | 3501.00(3265.67 to 3748.94) |  | 4(1 to 11) |  | 17.98(5.13 to 45.26) |
|  | Polycystic ovarian syndrome |  | 185(109 to 292) |  | 753.72(648.50 to 871.47) |  | 1(0 to 2) |  | 3.84(0.08 to 23.24) |
| Vanuatu | Endometriosis |  | 51(29 to 84) |  | 67.17(49.88 to 88.80) |  | 0(0 to 1) |  | 0.34(0.00 to 6.37) |
|  | Unexplained infertility |  | 2,520(1,119 to 4,980) |  | 3442.80(3309.08 to 3580.71) |  | 13(3 to 33) |  | 17.74(9.41 to 30.70) |
|  | Polycystic ovarian syndrome |  | 419(243 to 690) |  | 543.92(492.73 to 599.26) |  | 2(1 to 5) |  | 2.77(0.36 to 10.32) |
| Armenia | Endometriosis |  | 270(169 to 419) |  | 35.26(31.06 to 39.94) |  | 1(0 to 3) |  | 0.18(0.01 to 1.05) |
|  | Unexplained infertility |  | 6,593(3,703 to 10,402) |  | 843.31(822.55 to 864.54) |  | 34(11 to 82) |  | 4.29(2.93 to 6.18) |
|  | Polycystic ovarian syndrome |  | 1,001(632 to 1,559) |  | 131.52(123.24 to 140.29) |  | 5(2 to 12) |  | 0.67(0.21 to 1.74) |
| Azerbaijan | Endometriosis |  | 1,182(665 to 1,989) |  | 40.23(37.95 to 42.64) |  | 6(2 to 15) |  | 0.20(0.07 to 0.49) |
|  | Unexplained infertility |  | 107,900(40,097 to 236,833) |  | 3524.36(3503.18 to 3545.65) |  | 555(142 to 1,603) |  | 18.14(16.65 to 19.75) |
|  | Polycystic ovarian syndrome |  | 3,950(2,309 to 6,466) |  | 138.05(133.70 to 142.52) |  | 20(7 to 50) |  | 0.70(0.42 to 1.12) |
| Georgia | Endometriosis |  | 356(216 to 567) |  | 44.51(39.93 to 49.55) |  | 2(1 to 4) |  | 0.23(0.02 to 1.04) |
|  | Unexplained infertility |  | 22,486(6,626 to 53,183) |  | 2688.94(2653.45 to 2724.85) |  | 116(23 to 328) |  | 13.81(11.37 to 16.69) |
|  | Polycystic ovarian syndrome |  | 1,615(980 to 2,598) |  | 202.77(192.78 to 213.20) |  | 8(3 to 21) |  | 1.04(0.45 to 2.16) |
| Kazakhstan | Endometriosis |  | 2,290(1,311 to 3,824) |  | 46.62(44.70 to 48.59) |  | 12(4 to 29) |  | 0.24(0.12 to 0.43) |
|  | Unexplained infertility |  | 143,322(49,750 to 326,572) |  | 2821.67(2806.96 to 2836.44) |  | 739(183 to 2,019) |  | 14.55(13.51 to 15.65) |
|  | Polycystic ovarian syndrome |  | 7,022(4,136 to 11,473) |  | 144.75(141.34 to 148.23) |  | 36(12 to 89) |  | 0.73(0.51 to 1.03) |
| Kyrgyzstan | Endometriosis |  | 926(525 to 1,531) |  | 52.18(48.86 to 55.67) |  | 5(1 to 12) |  | 0.27(0.08 to 0.66) |
|  | Unexplained infertility |  | 71,760(30,428 to 147,086) |  | 4033.88(4004.30 to 4063.63) |  | 369(105 to 921) |  | 20.75(18.67 to 22.99) |
|  | Polycystic ovarian syndrome |  | 2,142(1,252 to 3,550) |  | 122.64(117.48 to 127.97) |  | 11(4 to 27) |  | 0.62(0.31 to 1.13) |
| Mongolia | Endometriosis |  | 449(251 to 751) |  | 51.16(46.50 to 56.20) |  | 2(1 to 6) |  | 0.26(0.04 to 0.97) |
|  | Unexplained infertility |  | 25,803(9,462 to 56,662) |  | 2855.59(2820.67 to 2890.87) |  | 132(32 to 357) |  | 14.65(12.25 to 17.44) |
|  | Polycystic ovarian syndrome |  | 1,132(674 to 1,850) |  | 131.10(123.50 to 139.08) |  | 6(2 to 14) |  | 0.67(0.24 to 1.56) |
| Tajikistan | Endometriosis |  | 1,113(651 to 1,812) |  | 41.91(39.46 to 44.47) |  | 6(2 to 14) |  | 0.22(0.08 to 0.50) |
|  | Unexplained infertility |  | 22,641(13,733 to 32,159) |  | 808.52(798.01 to 819.16) |  | 116(42 to 266) |  | 4.13(3.41 to 4.97) |
|  | Polycystic ovarian syndrome |  | 2,285(1,351 to 3,587) |  | 87.21(83.65 to 90.90) |  | 12(4 to 28) |  | 0.44(0.22 to 0.80) |
| Turkmenistan | Endometriosis |  | 641(352 to 1,079) |  | 50.72(46.86 to 54.81) |  | 3(1 to 8) |  | 0.26(0.06 to 0.74) |
|  | Unexplained infertility |  | 38,256(15,812 to 84,305) |  | 3040.66(3010.25 to 3071.31) |  | 196(51 to 507) |  | 15.61(13.50 to 17.96) |
|  | Polycystic ovarian syndrome |  | 1,813(1,061 to 2,961) |  | 144.28(137.71 to 151.09) |  | 9(3 to 23) |  | 0.73(0.34 to 1.39) |
| Uzbekistan | Endometriosis |  | 4,034(2,281 to 6,549) |  | 43.44(42.10 to 44.80) |  | 21(6 to 51) |  | 0.22(0.14 to 0.35) |
|  | Unexplained infertility |  | 213,711(72,190 to 460,714) |  | 2256.23(2246.65 to 2265.84) |  | 1,098(271 to 3,042) |  | 11.60(10.92 to 12.31) |
|  | Polycystic ovarian syndrome |  | 12,939(7,630 to 20,588) |  | 141.87(139.42 to 144.35) |  | 66(21 to 161) |  | 0.72(0.56 to 0.92) |
| Albania | Endometriosis |  | 223(134 to 367) |  | 36.25(31.64 to 41.38) |  | 1(0 to 3) |  | 0.18(0.01 to 1.06) |
|  | Unexplained infertility |  | 9,216(6,524 to 12,282) |  | 1496.88(1466.46 to 1527.81) |  | 47(18 to 101) |  | 7.65(5.62 to 10.22) |
|  | Polycystic ovarian syndrome |  | 275(162 to 443) |  | 45.26(40.05 to 51.00) |  | 1(0 to 3) |  | 0.23(0.01 to 1.13) |
| Bosnia and Herzegovina | Endometriosis |  | 298(173 to 482) |  | 40.81(36.24 to 45.87) |  | 2(0 to 4) |  | 0.21(0.01 to 1.05) |
|  | Unexplained infertility |  | 24,647(9,133 to 53,728) |  | 3333.75(3291.83 to 3376.13) |  | 126(34 to 343) |  | 17.09(14.21 to 20.45) |
|  | Polycystic ovarian syndrome |  | 414(246 to 706) |  | 56.68(51.24 to 62.59) |  | 2(1 to 5) |  | 0.29(0.04 to 1.17) |
| Bulgaria | Endometriosis |  | 618(362 to 1,038) |  | 43.94(40.42 to 47.72) |  | 3(1 to 8) |  | 0.23(0.05 to 0.74) |
|  | Unexplained infertility |  | 49,771(18,466 to 112,171) |  | 3383.06(3352.65 to 3413.72) |  | 255(63 to 707) |  | 17.32(15.21 to 19.69) |
|  | Polycystic ovarian syndrome |  | 916(545 to 1,537) |  | 64.81(60.51 to 69.37) |  | 5(2 to 12) |  | 0.33(0.09 to 0.89) |
| Croatia | Endometriosis |  | 321(196 to 503) |  | 36.00(32.12 to 40.29) |  | 2(1 to 4) |  | 0.18(0.02 to 0.90) |
|  | Unexplained infertility |  | 31,372(11,400 to 70,323) |  | 3407.58(3369.60 to 3445.94) |  | 161(39 to 433) |  | 17.49(14.87 to 20.50) |
|  | Polycystic ovarian syndrome |  | 565(336 to 944) |  | 62.92(57.74 to 68.50) |  | 3(1 to 8) |  | 0.32(0.06 to 1.10) |
| Czechia | Endometriosis |  | 1,064(619 to 1,728) |  | 47.74(44.80 to 50.84) |  | 5(2 to 13) |  | 0.24(0.08 to 0.61) |
|  | Unexplained infertility |  | 114,110(50,510 to 226,324) |  | 4981.53(4951.94 to 5011.29) |  | 586(170 to 1,591) |  | 25.56(23.48 to 27.80) |
|  | Polycystic ovarian syndrome |  | 1,429(848 to 2,387) |  | 64.02(60.59 to 67.61) |  | 7(2 to 18) |  | 0.32(0.13 to 0.72) |
| Hungary | Endometriosis |  | 870(499 to 1,463) |  | 41.90(39.09 to 44.88) |  | 4(1 to 11) |  | 0.22(0.06 to 0.58) |
|  | Unexplained infertility |  | 70,313(27,077 to 156,795) |  | 3359.20(3333.99 to 3384.58) |  | 361(96 to 967) |  | 17.22(15.46 to 19.15) |
|  | Polycystic ovarian syndrome |  | 1,333(814 to 2,210) |  | 63.86(60.37 to 67.53) |  | 7(2 to 16) |  | 0.32(0.12 to 0.73) |
| North Macedonia | Endometriosis |  | 231(132 to 367) |  | 42.19(36.85 to 48.22) |  | 1(0 to 3) |  | 0.21(0.01 to 1.40) |
|  | Unexplained infertility |  | 18,774(6,974 to 42,078) |  | 3324.47(3276.67 to 3372.91) |  | 96(25 to 264) |  | 17.07(13.81 to 21.01) |
|  | Polycystic ovarian syndrome |  | 315(187 to 531) |  | 58.11(51.73 to 65.17) |  | 2(1 to 4) |  | 0.29(0.02 to 1.52) |
| Montenegro | Endometriosis |  | 63(36 to 107) |  | 44.09(33.80 to 56.76) |  | 0(0 to 1) |  | 0.22(0.00 to 3.69) |
|  | Unexplained infertility |  | 4,680(1,806 to 10,641) |  | 3220.95(3128.66 to 3315.49) |  | 24(6 to 67) |  | 16.56(10.58 to 24.99) |
|  | Polycystic ovarian syndrome |  | 91(54 to 153) |  | 63.53(51.02 to 78.40) |  | 0(0 to 1) |  | 0.32(0.00 to 3.86) |
| Poland | Endometriosis |  | 3,695(2,145 to 6,164) |  | 40.11(38.78 to 41.48) |  | 19(6 to 47) |  | 0.21(0.12 to 0.34) |
|  | Unexplained infertility |  | 564,600(258,285 to 1,078,624) |  | 5904.64(5888.86 to 5920.45) |  | 2,897(878 to 7,786) |  | 30.29(29.17 to 31.45) |
|  | Polycystic ovarian syndrome |  | 7,101(4,474 to 11,162) |  | 78.47(76.58 to 80.41) |  | 36(13 to 88) |  | 0.40(0.27 to 0.57) |
| Romania | Endometriosis |  | 1,698(985 to 2,845) |  | 43.22(41.13 to 45.40) |  | 9(3 to 20) |  | 0.22(0.10 to 0.44) |
|  | Unexplained infertility |  | 132,134(49,479 to 298,061) |  | 3295.97(3277.88 to 3314.14) |  | 679(176 to 1,887) |  | 16.92(15.65 to 18.28) |
|  | Polycystic ovarian syndrome |  | 2,442(1,444 to 4,054) |  | 61.29(58.81 to 63.86) |  | 12(4 to 30) |  | 0.31(0.16 to 0.56) |
| Serbia | Endometriosis |  | 785(457 to 1,317) |  | 38.66(35.97 to 41.51) |  | 4(1 to 10) |  | 0.20(0.05 to 0.54) |
|  | Unexplained infertility |  | 68,572(25,383 to 152,420) |  | 3309.83(3284.90 to 3334.92) |  | 352(93 to 967) |  | 16.97(15.23 to 18.88) |
|  | Polycystic ovarian syndrome |  | 1,194(698 to 1,991) |  | 58.69(55.35 to 62.18) |  | 6(2 to 15) |  | 0.30(0.11 to 0.68) |
| Slovakia | Endometriosis |  | 527(307 to 844) |  | 41.09(37.56 to 44.92) |  | 3(1 to 6) |  | 0.21(0.04 to 0.79) |
|  | Unexplained infertility |  | 44,584(17,021 to 100,594) |  | 3331.31(3299.86 to 3363.05) |  | 229(61 to 628) |  | 17.10(14.92 to 19.58) |
|  | Polycystic ovarian syndrome |  | 794(469 to 1,317) |  | 61.95(57.55 to 66.64) |  | 4(1 to 10) |  | 0.31(0.08 to 0.93) |
| Slovenia | Endometriosis |  | 238(141 to 393) |  | 57.16(49.91 to 65.30) |  | 1(0 to 3) |  | 0.29(0.01 to 1.83) |
|  | Unexplained infertility |  | 10,780(3,192 to 25,639) |  | 2479.94(2432.37 to 2528.35) |  | 56(11 to 163) |  | 12.79(9.59 to 16.89) |
|  | Polycystic ovarian syndrome |  | 294(178 to 485) |  | 70.16(62.09 to 79.13) |  | 1(0 to 3) |  | 0.35(0.02 to 1.92) |
| Belarus | Endometriosis |  | 1,452(825 to 2,425) |  | 64.89(61.47 to 68.48) |  | 7(2 to 18) |  | 0.33(0.13 to 0.75) |
|  | Unexplained infertility |  | 92,781(37,776 to 197,484) |  | 4011.54(3985.00 to 4038.25) |  | 478(126 to 1,388) |  | 20.68(18.82 to 22.72) |
|  | Polycystic ovarian syndrome |  | 1,583(925 to 2,670) |  | 70.35(66.78 to 74.09) |  | 8(3 to 21) |  | 0.36(0.15 to 0.78) |
| Estonia | Endometriosis |  | 180(104 to 290) |  | 62.28(53.29 to 72.56) |  | 1(0 to 2) |  | 0.32(0.00 to 2.47) |
|  | Unexplained infertility |  | 12,155(5,103 to 25,242) |  | 4116.67(4042.43 to 4192.12) |  | 63(19 to 176) |  | 21.17(16.16 to 27.49) |
|  | Polycystic ovarian syndrome |  | 232(137 to 395) |  | 80.32(70.05 to 91.88) |  | 1(0 to 3) |  | 0.41(0.01 to 2.60) |
| Latvia | Endometriosis |  | 245(141 to 402) |  | 61.45(53.77 to 70.08) |  | 1(0 to 3) |  | 0.31(0.01 to 1.96) |
|  | Unexplained infertility |  | 17,781(7,054 to 36,891) |  | 4340.67(4275.74 to 4406.49) |  | 91(26 to 266) |  | 22.33(17.90 to 27.71) |
|  | Polycystic ovarian syndrome |  | 307(179 to 516) |  | 76.78(68.16 to 86.35) |  | 2(1 to 4) |  | 0.39(0.03 to 2.07) |
| Lithuania | Endometriosis |  | 441(253 to 727) |  | 75.29(68.35 to 82.84) |  | 2(1 to 5) |  | 0.38(0.05 to 1.56) |
|  | Unexplained infertility |  | 26,998(11,552 to 57,113) |  | 4594.26(4539.36 to 4649.75) |  | 139(38 to 388) |  | 23.64(19.86 to 28.05) |
|  | Polycystic ovarian syndrome |  | 446(258 to 739) |  | 76.81(69.74 to 84.50) |  | 2(1 to 6) |  | 0.39(0.06 to 1.58) |
| Republic of Moldova | Endometriosis |  | 544(308 to 917) |  | 57.41(52.52 to 62.72) |  | 3(1 to 7) |  | 0.29(0.05 to 1.14) |
|  | Unexplained infertility |  | 41,167(17,367 to 86,677) |  | 4188.18(4146.78 to 4229.98) |  | 212(62 to 614) |  | 21.59(18.71 to 24.89) |
|  | Polycystic ovarian syndrome |  | 607(353 to 1,012) |  | 63.88(58.70 to 69.48) |  | 3(1 to 7) |  | 0.33(0.06 to 1.19) |
| Russian Federation | Endometriosis |  | 25,324(14,567 to 41,194) |  | 70.90(69.99 to 71.82) |  | 130(43 to 315) |  | 0.36(0.30 to 0.44) |
|  | Unexplained infertility |  | 2,193,912(986,615 to 4,504,209) |  | 5985.97(5977.75 to 5994.19) |  | 11,275(3,477 to 32,129) |  | 30.78(30.19 to 31.37) |
|  | Polycystic ovarian syndrome |  | 27,019(15,751 to 44,383) |  | 74.70(73.77 to 75.64) |  | 137(45 to 336) |  | 0.38(0.32 to 0.45) |
| Ukraine | Endometriosis |  | 6,486(3,623 to 10,935) |  | 60.07(58.56 to 61.62) |  | 33(10 to 83) |  | 0.31(0.21 to 0.45) |
|  | Unexplained infertility |  | 512,909(230,569 to 1,050,249) |  | 4718.16(4704.85 to 4731.50) |  | 2,646(742 to 7,848) |  | 24.33(23.39 to 25.32) |
|  | Polycystic ovarian syndrome |  | 6,406(3,656 to 10,342) |  | 58.62(57.13 to 60.15) |  | 32(10 to 79) |  | 0.30(0.20 to 0.44) |
| Brunei Darussalam | Endometriosis |  | 46(23 to 79) |  | 34.21(25.00 to 46.10) |  | 0(0 to 1) |  | 0.18(0.00 to 4.14) |
|  | Unexplained infertility |  | 873(83 to 3,199) |  | 642.99(601.03 to 687.44) |  | 5(0 to 21) |  | 3.43(1.06 to 8.90) |
|  | Polycystic ovarian syndrome |  | 952(548 to 1,564) |  | 715.54(670.71 to 762.89) |  | 5(2 to 11) |  | 3.65(1.16 to 9.25) |
| Japan | Endometriosis |  | 10,808(5,935 to 17,743) |  | 39.33(38.57 to 40.10) |  | 56(16 to 133) |  | 0.20(0.15 to 0.27) |
|  | Unexplained infertility |  | 392,965(30,193 to 1,217,868) |  | 1331.36(1327.15 to 1335.58) |  | 2,070(104 to 8,499) |  | 7.00(6.70 to 7.32) |
|  | Polycystic ovarian syndrome |  | 351,229(219,971 to 525,855) |  | 1309.96(1305.51 to 1314.43) |  | 1,789(639 to 3,954) |  | 6.67(6.36 to 7.00) |
| Republic of Korea | Endometriosis |  | 4,540(2,292 to 7,700) |  | 34.71(33.69 to 35.77) |  | 23(7 to 60) |  | 0.18(0.11 to 0.28) |
|  | Unexplained infertility |  | 103,324(8,416 to 365,935) |  | 722.04(717.60 to 726.51) |  | 548(27 to 2,352) |  | 3.83(3.51 to 4.18) |
|  | Polycystic ovarian syndrome |  | 79,375(46,924 to 125,487) |  | 620.43(616.02 to 624.87) |  | 403(138 to 921) |  | 3.15(2.84 to 3.49) |
| Singapore | Endometriosis |  | 576(282 to 961) |  | 31.57(28.92 to 34.51) |  | 3(1 to 8) |  | 0.16(0.03 to 0.77) |
|  | Unexplained infertility |  | 17,104(1,230 to 61,145) |  | 797.89(785.81 to 810.23) |  | 91(4 to 386) |  | 4.24(3.40 to 5.38) |
|  | Polycystic ovarian syndrome |  | 13,171(7,648 to 20,636) |  | 736.38(723.20 to 749.85) |  | 67(24 to 158) |  | 3.76(2.87 to 4.97) |
| Australia | Endometriosis |  | 1,355(593 to 2,593) |  | 21.22(20.10 to 22.40) |  | 7(2 to 19) |  | 0.11(0.04 to 0.24) |
|  | Unexplained infertility |  | 12,784(3,103 to 63,159) |  | 192.42(189.09 to 195.81) |  | 69(9 to 358) |  | 1.04(0.81 to 1.32) |
|  | Polycystic ovarian syndrome |  | 43,692(22,713 to 75,719) |  | 679.83(673.43 to 686.29) |  | 224(69 to 539) |  | 3.48(3.04 to 3.98) |
[truncated: 58,120 more chars]
